# TAK1 inhibition attenuates both inflammation and fibrosis in experimental pneumoconiosis

**DOI:** 10.1038/celldisc.2017.23

**Published:** 2017-07-11

**Authors:** Jie Li, Chao Liang, Zong-Kang Zhang, Xiaohua Pan, Songlin Peng, Wing-Sze Lee, Aiping Lu, Zhixiu Lin, Ge Zhang, Wing-Nang Leung, Bao-Ting Zhang

**Affiliations:** 1 School of Chinese Medicine, Faculty of Medicine, The Chinese University of Hong Kong, Sha Tin, Hong Kong SAR, China; 2 Institute of Integrated Bioinformedicine and Translational Science (IBTS), School of Chinese Medicine, Hong Kong Baptist University, Kowloon Tong, Hong Kong SAR, China; 3 Law Sau Fai Institute for Advancing Translational Medicine in Bone and Joint Diseases (TMBJ), School of Chinese Medicine, Hong Kong Baptist University, Kowloon Tong, Hong Kong SAR, China; 4 Department of Surgery, Shenzhen Bao’an District People’s Hospital, Shenzhen, China; 5 Department of Surgery, Shenzhen People’s Hospital, Jinan University School of Medicine, Shenzhen, China

**Keywords:** TAK1, inflammation, fibrosis, pneumoconiosis

## Abstract

Pneumoconiosis, caused by inhalation of mineral dusts, is a major occupational disease worldwide. Currently, there are no effective drugs owing to a lack of potential therapeutic targets during either the inflammation or fibrosis molecular events in pneumoconiosis. Here, we performed microarrays to identify aberrantly expressed genes in the above molecular events *in vitro* and found a hub gene transforming growth factor-β-activated kinase 1 (*TAK1*), which was highly expressed and activated in pneumoconiosis patients as well as silica-exposed rats with experimental pneumoconiosis. Genetic modulation of *TAK1* by CRISPR (clustered regularly interspaced short palindromic repeats)/Cas9, RNA interference and overexpression indicated the important role of *TAK1* in both inflammation and fibrosis in experimental pneumoconiosis. To achieve pharmacological *TAK1* inhibition, we virtually screened out a natural product resveratrol, which targeted *TAK1* at both N161 and A107 residues, and significantly inhibited *TAK1* activation to attenuate inflammation and fibrosis *in vitro*. Consistently, *in vivo* prevention and intervention studies showed that resveratrol could inhibit pulmonary inflammation and fibrosis in silica-exposed rats.

## Introduction

Pneumoconiosis is one of the most serious occupational diseases worldwide. It is usually caused by chronic inhalation of mineral dusts, especially silica and asbestos [1, 2]. Pneumoconiosis is characterized by the accumulation of inflammatory cells and the subsequent formation of fibrotic nodules in the lungs, leading to breathing problems, lung cancers and even death [3, 4]. However, there are no effective drugs owing to a lack of potential therapeutic targets. The most commonly used corticosteroids are broad-spectrum anti-inflammatory and anti-fibrotic agents, which always arouse severe side effects such as tuberculosis infection, osteonecrosis and gastrointestinal symptoms after long-term clinical use [5,6,7].

Development of pneumoconiosis includes inflammatory response at early stage and the consequent fibrosis at late stage [8, 9]. Typically, inhaled silica dusts induce the accumulation and activation of multiple lung cell types including macrophages, lymphocytes and epithelial cells. Then, the activated cells release inflammatory and fibrogenic mediators such as interleukin-1β (IL-1β), tumor necrosis factor-α (TNF-α), matrix metalloproteinases (MMPs) and transforming growth factor-β (TGF-β) [10,11,12,13]. In turn, lung fibroblasts are stimulated to proliferate by the released mediators and generate collagens, which surround the inhaled silica dusts and form hyalinized nodules. These multiple and constant nodules ultimately lead to progressive massive fibrosis [14, 15]. Accordingly, we hypothesized that key regulatory factors, which control both inflammation and the consequent fibrosis in pneumoconiosis, could be ideal therapeutic targets.

TGF-β-activated kinase 1 (*TAK1*, also called as *MAP3K7*) is a member of the mitogen-activated protein kinase kinase kinase (MAP3K) family [16]. It is an intracellular hub molecule that regulates nuclear factor-κB, c-Jun N-terminal kinase (JNK), p38 and TGF-β signaling, which are classical pathways involved in inflammation and fibrosis [15,16,17]. TAK1 activity is tightly regulated by multiple binding partners including TAB1, TAB2 and TAB3, as well as post-translational modifications such as phosphorylation, ubiquitination and methylation [ 17,18,19,20]. TAK1 activation correlates with phosphorylation primarily on T187 and also on T178, T184 and S192. In particular, phosphorylation of T187 is required for TAK1 kinase and increase in response to stimulation by inflammatory cytokines [21, 22]. To date, accumulating evidence demonstrates that TAK1 participates in inflammatory and fibrotic response in kidney, liver and heart disorders and cancers [23]. However, poor evidence indicates the role of TAK1 in inflammation and fibrosis in lung diseases and remodeling, especially the pneumoconiosis.

In our study, we performed microarrays to determine gene signatures in cultured alveolar macrophages with silica exposure and lung fibroblasts with TGF-β stimulation *in vitro*. We overlapped the upregulated genes and found a highly expressed and activated *TAK1* in lung specimens from pneumoconiosis patients as well as primary alveolar macrophages and lung fibroblasts isolated from silica-exposed rats with experimental pneumoconiosis. By CRISPR (clustered regularly interspaced short palindromic repeats)/Cas9-based genome-editing approach, we demonstrated that loss of *TAK1* in lung tissues could alleviate silica-induced inflammation and fibrosis in silica-exposed mice with experimental pneumoconiosis. In addition, gene modulation of *TAK1* by *TAK1* small interfering RNA (siRNA) and overexpression vector confirmed the key role of TAK1 in both inflammation and fibrosis in experimental pneumoconiosis *in vitro*. Molecular docking identified resveratrol as a small-molecule inhibitor for TAK1. Meanwhile, we also validated that both N161 and A107 residues in TAK1 were essential for resveratrol–TAK1 interaction. *In vitro* and *in vivo* data showed that resveratrol inhibited TAK1 activation, inflammation and fibrosis in experimental pneumoconiosis. Thus, the present findings not only revealed TAK1 as a potential molecular target regulating both inflammation and fibrosis in experimental pneumoconiosis, but also identified resveratrol as a TAK1 small-molecule inhibitor, which could be further developed as a promising agent for pneumoconiosis.

## Results

### TAK1 as a highly expressed and activated protein in pneumoconiosis

**Figure 1:**
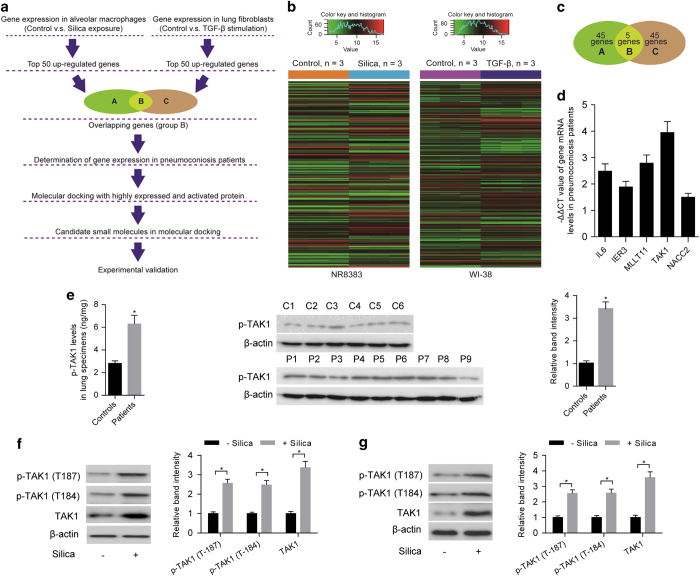
TAK1 as a highly expressed and activated protein in pneumoconiosis. (**a**) Flow chart depicting experimental design in our study. (**b**) Heatmaps showing gene signatures in alveolar macrophage NR8383 cells exposed to silica (*n*=3) or vehicle control (*n*=3) and lung fibroblast WI-38 cells stimulated with TGF-β (*n*=3) or vehicle control (*n*=3), respectively. (**c**) Five overlapping genes between top 50 upregulated genes in silica-exposed NR8383 cells and top 50 upregulated genes in TGF-β-stimulated WI-38 cells. (**d**) −ΔΔCT value of mRNA level for the five overlapping genes in lung specimens from pneumoconiosis patients (*n*=9) as determined by real-time PCR. GAPDH was used as a control gene for internal correction. Expression of the overlapping genes in control individuals (*n*=6) served as the calibrators. ΔΔCT=(CT_target_−CT_GAPDH_)_patients_−(CT_target_−CT_GAPDH_)_controls_. (**e**) The level of p-TAK1 in lung specimens from pneumoconiosis patients (*n*=9) or control individuals (*n*=6) as determined by ELISA (left) and western blot (middle: representative images; right: relative bands intensity). (**f**) Levels of p-TAK1 (T187, T184) and TAK1 were determined by western blotting (left: representative images; right: relative bands intensity) in primary alveolar macrophages isolated from rats with (*n*=5) or without silica exposure (*n*=5). (**g**) Levels of p-TAK1 (T187, T184) and TAK1 were determined by western blotting (left: representative images; right: relative bands intensity) in primary lung fibroblasts isolated from rats with (*n*=5) or without silica exposure (*n*=5). Data are presented as mean±s.d. *P<0.05. One-way analysis of variance (ANOVA) with a *post hoc* test was performed and the statistical differences between the two groups were determined by the Student’s *t*-test.

Figure 1a illustrated the overall experimental design in our study. Given that inflammation and the consequent fibrosis are two major molecular events in pneumoconiosis, hub regulatory factors simultaneously controlling the two events are ideal as potential therapeutic targets. Thus, cultured alveolar macrophage NR8383 cells were exposed to silica to induce inflammatory response and lung fibroblast WI-38 cells were stimulated with TGF-β to induce fibrotic response [24, 25]. Microarrays were used to determine gene signatures in the above two cell lines (Figure 1b). We selected top 50 upregulated genes from each gene signature of the two cell lines (Supplementary Tables S1 and S2) and obtained five overlapping genes (*IL6*, *IER3*, *MLLT11*, *TAK1* and *NACC2*) (Figure 1c and Supplementary Table S3). To confirm the clinical association of the overlapping genes with pneumoconiosis, we examined the mRNA levels of the overlapping genes in human lung specimens (Supplementary Table S4). Compared with control individuals, real-time PCR results showed that the mRNA levels of overlapping genes were higher in pneumoconiosis patients. Furthermore, *TAK1* exhibited the highest expression level and thus drew our attention for further investigation (Figure 1d). Results from enzyme-linked immunosorbent assay (ELISA) and western blotting demonstrated that level of phosphorylated TAK1 (T187, p-TAK1), was also higher in pneumoconiosis patients when compared with control individuals (Figure 1e).

To further explore the relationship between TAK1 and pneumoconiosis, Sprague–Dawley rats were exposed to silica aerosol to induce experimental pneumoconiosis [26]. After silica exposure for 20 days, primary alveolar macrophages were isolated to determine levels of TAK1, TAK1 activation (T184 and T187, p-TAK1) and inflammatory response at an early stage of experimental pneumoconiosis [26, 27]. Compared with primary alveolar macrophages isolated from rats with no silica exposure, western blotting results showed that the levels of TAK1 and p-TAK1 (T184 and T187) were higher in primary alveolar macrophages isolated from silica-exposed rats (Figure 1f). Meanwhile, increased levels of inflammatory indicators including inflammatory cytokines (IL-1β, TGF-β and TNF-α) determined by ELISA and activities of MMPs (MMP-9 and MMP-2) examined by gelatin zymography were observed in primary alveolar macrophages isolated from silica-exposed rats (Supplementary Figure S1A). We also isolated primary lung fibroblasts from rats with silica exposure for 40 days and determined the levels of TAK1, TAK1 activation (T184 and T187, p-TAK1) and fibrotic response at a late stage of experimental pneumoconiosis [26, 27]. Compared with primary lung fibroblasts isolated from rats with no silica exposure, we found that levels of TAK1 and p-TAK1 (T184 and T187) were also higher in primary lung fibroblasts isolated from silica-exposed rats (Figure 1g). Besides, elevated levels of fibrotic indicators, including collagen subtypes (collagen type I (Col I) and collagen type III (Col III)) determined by western blotting and cell proliferation rate examined by the 2-(4,5-dimethyl-2-thiazolyl)-3,5-diphenyl-, bromide (MTT) assay, were also observed in primary lung fibroblasts isolated from silica-exposed rats (Supplementary Figure S1B).

### Knockdown of TAK1 in lungs by CRISPR/Cas9 system reduced inflammation and fibrosis

**Figure 2:**
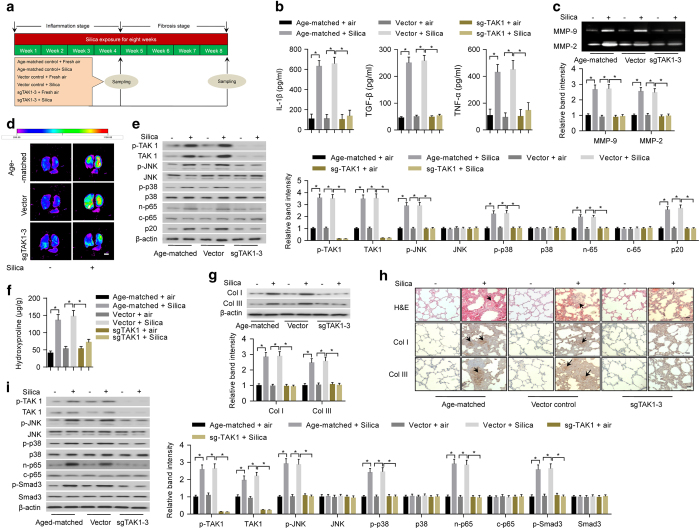
Deletion of TAK1 in lung tissues by lentiviral-based CRISPR/Cas9 system reduced inflammation and fibrosis in silica-exposed mice. (**a**) A schematic diagram illustrating the experimental design. Before induction of inflammation and fibrosis in lungs by silica exposure, the mice were infected intratracheally with lentiviral vector expressing CRISPR/Cas9 system (sgTAK1–3 and Cas9) or lentiviral vector control. (**b**) ELISA to determine silica exposure-induced levels of IL-1β (left), TGF-β (middle) and TNF-α (right) in the supernatant of BALF from age-matched mice or mice infected with specified lentiviral vectors. (**c**) Gelatin zymography (upper: representative images; bottom: relative bands intensity) to examine silica exposure-induced activities of matrix metalloproteinases (MMP-9 and MMP-2) in homogenized lung tissues from age-matched mice or mice infected with specified lentiviral vectors. (**d**) MMP-targeted NIR fluorescence imaging showing silica exposure-induced activities of matrix metalloproteinases in lung tissues from age-matched mice or mice infected with specified lentiviral vectors. Scale bar, 1 cm. (**e**) Western blotting (left: representative images; right: relative bands intensity) to examine silica exposure-induced inflammation-related downstream signaling in primary alveolar macrophages isolated from lung tissues of age-matched mice or mice infected with specified lentiviral vectors. (**f**) Hydroxyproline assay to determine silica exposure-induced total collagen levels in lung tissues from age-matched mice or mice infected with specified lentiviral vectors. (**g**) Western blotting (upper: representative images; bottom: relative bands intensity) to determine silica exposure-induced levels of Col I and Col III in lung tissue from age-matched mice or mice infected with specified lentiviral vectors. (**h**) Histological examination to determine silica exposure-induced fibrotic nodule formation (arrows indicated in H&E staining) and collagen deposition (arrows indicated in immunohistochemical staining) in lung tissue from age-matched mice or mice infected with specified lentiviral vectors. Scale bars, 50 μm. (**i**) Western blotting (left: representative images; right: relative bands intensity) to examine silica exposure-induced fibrosis-related downstream signaling in primary fibroblasts isolated from lung tissues of age-matched mice or mice infected with specified lentiviral vectors. Data are presented as mean±s.d. *P<0.05, *n*=9 per group. One-way ANOVA with a *post hoc* test was performed and the statistical differences between the two groups were determined by the Student’s *t*-test.

Since TAK1-deficient mice are embryonic lethal [28], it is impractical to delete *TAK1* in alveolar macrophages and lung fibroblasts by traditional methods to explore the role of TAK1 in both inflammation and fibrosis during development of pneumoconiosis. Recent studies have demonstrated the feasibility of using the CRISPR/Cas9 system to directly delete target genes in lung tissues [29, 30]. In this study, we used lentiviral-based CRISPR/Cas9 system to generate a lung tissue-specific *TAK1* knockdown mouse model. We constructed lentiviral vectors expressing CRISPR/Cas9 components including single-stranded RNA against *TAK1* (sgTAK1) and Cas9. Both mouse primary alveolar macrophages and lung fibroblasts incubated with lentiviral vectors expressing sgRNA-3 showed significantly increased insertion/deletion frequency and reduced TAK1 protein expression when compared with other sgRNAs (Supplementary Figure S2A and B). Furthermore, the lentiviruses were intratracheally delivered into C57BL/6 mouse lungs once a week. At 4 weeks after infection, the results consistently showed that sgTAK1–3 achieved the highest insertion/deletion frequency and *TAK1* knockdown efficiency in mouse lung tissues (Supplementary Figure S2C). Then, the mice were exposed to silica aerosol to induce experimental pneumoconiosis [27] (Figure 2a). After silica exposure for 20 days, we found that the levels of inflammatory cytokines (IL-1β, TGF-β and TNF-α) in the supernatant of bronchoalveolar lavage fluid (BALF) determined by ELISA (Figure 2b) and activities of MMPs in lung tissues examined by gelatin zymography and MMP-targeted near-infrared (NIR) fluorescence imaging (Figure 2c and d) were obviously lower in mice with periodic intratracheal injections of lentiviruses expressing sgTAK1–3 and Cas9, when compared with those in age-matched mice or mice with periodic intratracheal injections of lentiviral vector control. Furthermore, we isolated primary alveolar macrophages and evaluated inflammation-related downstream signaling by western blotting. Consistently, the levels of phosphorylated-JNK (p-JNK), phosphorylated-p38 (p-p38), nuclear p65 (n-p65) and p20 were markedly lower in primary alveolar macrophages isolated from mice with periodic intratracheal injections of lentiviruses expressing sgTAK1–3 and Cas9 (Figure 2e). After silica exposure for 40 days, we found that levels of collagens and collagen subtypes (Col I and Col III) in lung tissues determined by hydroxyproline assay and western blotting, respectively, were significantly lower in mice with periodic intratracheal injections of lentiviruses expressing sgTAK1–3 and Cas9 (Figure 2f and g). We also performed hematoxylin and eosin (H&E) staining and immunohistochemistry to examine fibrotic nodule formation and collagen deposition. We found that fibrotic nodule formation and deposition of Col I and Col III in lung tissues were obviously less in mice with periodic intratracheal injections of lentiviruses expressing sgTAK1–3 and Cas9 (Figure 2h). Further, we evaluated fibrosis-related downstream signaling in primary lung fibroblasts by western blotting. Our results showed that levels of p-JNK, p-p38, n-p65 and phosphorylated-Smad3 (p-Smad3) were markedly lower in mice with periodic intratracheal injections of lentiviruses expressing sgTAK1–3 and Cas9 (Figure 2i).

### Role of TAK1 in both inflammation and fibrosis in experimental pneumoconiosis

**Figure 3:**
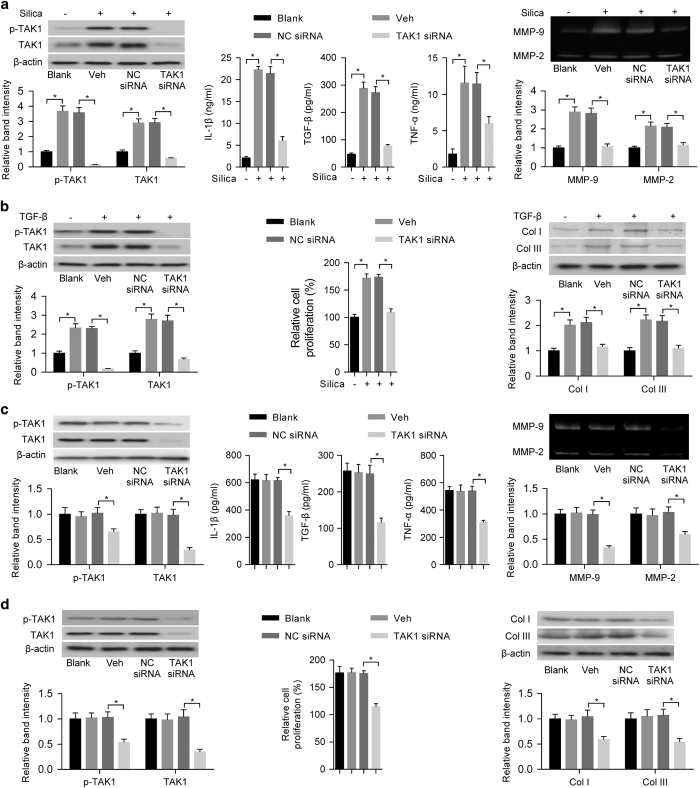
Effects of TAK1 gene silencing on inflammation and fibrosis in experimental pneumoconiosis. (**a**) Levels of TAK1 and p-TAK1 were determined by western blotting (left upper: representative images; left bottom: relative bands intensity), inflammatory cytokines (IL-1β, TGF-β and TNF-α) were examined by ELISA (middle) and MMPs (MMP-9 and MMP-2) were analyzed by gelatin zymography (right upper: representative images; right bottom: relative bands intensity) in silica-exposed NR8383 cells with prior incubation of TAK1 siRNA, NC siRNA or vehicle control (Veh: Lipofectamine 2000); *n*=4 per group. (**b**) Levels of p-TAK1 and TAK1 were determined by western blotting (left upper: representative images; left bottom: relative bands intensity), cell proliferation rate was examined by MTT assay (middle) and collagen subtypes (Col I and Col III) were determined by western blotting (right upper: representative images; right bottom: relative bands intensity) in TGF-β-stimulated WI-38 cells with prior incubation of TAK1 siRNA, NC siRNA or vehicle control (Veh: Lipofectamine 2000). *n*=4 per group. (**c**) Levels of TAK1 and p-TAK1 were determined by western blotting (left upper: representative images; left bottom: relative bands intensity), inflammatory cytokines (IL-1β, TGF-β and TNF-α) were examined by ELISA (middle) and MMPs (MMP-9 and MMP-2) were analyzed by gelatin zymography (right upper: representative images; right bottom: relative bands intensity) in primary alveolar macrophages with incubation of TAK1 siRNA, NC siRNA or vehicle control (Veh: Lipofectamine 2000). The primary alveolar macrophages were isolated from silica-exposed rats; *n*=5 per group. (**d**) Levels of p-TAK1 and TAK1 were determined by western blotting (left upper: representative images; left bottom: relative bands intensity), cell proliferation rate was examined by MTT assay (middle) and collagen subtypes (Col I and Col III) were determined by western blotting (right upper: representative images; right bottom: relative bands intensity) in primary lung fibroblasts with incubation of TAK1 siRNA, NC siRNA or vehicle control (Veh: Lipofectamine 2000). The primary lung fibroblasts were isolated from silica-exposed rats; *n*=5 per group. Data are presented as mean±s.d. *P<0.05. One-way ANOVA with a *post hoc* test was performed and the statistical differences between the two groups were determined by the Student’s *t*-test.

To further examine the role of TAK1 in both inflammation and fibrosis in experimental pneumoconiosis, we transfected cultured alveolar macrophage NR8383 cells and lung fibroblast WI-38 cells with *TAK1* siRNA or nonsense control siRNA (NC siRNA) *in vitro*. After silica exposure for NR8383 cells, there were lower levels of TAK1 and p-TAK1, supernatant inflammatory cytokines (IL-1β, TGF-β and TNF-α) and activities of MMPs (MMP-9 and MMP-2) in *TAK1* siRNA-transfected NR8383 cells when compared with vehicle- or NC siRNA-transfected NR8383 cells (Figure 3a). After TGF-β stimulation for WI-38 cells, our results showed that the levels of TAK1 and p-TAK1, collagen subtypes (Col I and Col III) and cell proliferation rate were lower in *TAK1* siRNA-transfected WI-38 cells when compared with vehicle- or NC siRNA-transfected WI-38 cells (Figure 3b). We also transfected primary alveolar macrophages or lung fibroblasts isolated from silica-exposed rats with *TAK1* siRNA or NC siRNA *in vitro*. We found that *TAK1* siRNA-transfected primary alveolar macrophages had decreased levels of TAK1 and p-TAK1, inflammatory cytokines (IL-1β, TGF-β and TNF-α) and activities of MMPs (MMP-9 and MMP-2) when compared to vehicle- or NC siRNA-transfected primary alveolar macrophages (Figure 3c). Meanwhile, we also found that *TAK1* siRNA-transfected primary lung fibroblasts had reduced levels of TAK1 and p-TAK1, collagen subtypes (Col I and Col III) and cell proliferation rate when compared with vehicle- or NC siRNA-transfected primary lung fibroblasts (Figure 3d).

We also transfected primary alveolar macrophages or lung fibroblasts isolated from silica-exposed rats with TAK1 overexpression vector or empty vector. Our results showed that primary alveolar macrophages with transfection of TAK1 overexpression vector had increased levels of TAK1 and p-TAK1, inflammatory cytokines (IL-1β, TGF-β and TNF-α) and activities of MMPs (MMP-9 and MMP-2) when compared with vehicle- or empty vector-transfected primary alveolar macrophages (Supplementary Figure S3A). Meanwhile, primary lung fibroblasts with transfection of TAK1 overexpression vector had increased levels of TAK1 and p-TAK1, collagen subtypes (Col I and Col III) and cell proliferation when compared with vehicle- or empty vector-transfected primary lung fibroblasts (Supplementary Figure S3B). All these results indicated the important role of TAK1 in both inflammation and fibrosis in experimental pneumoconiosis *in vitro*.

### *In vitro* effects of TAK1 inhibitor 5*Z*-7-oxozeaenol on inflammation and fibrosis and cytotoxicity

To examine whether TAK1 inhibition by pharmacological approach could attenuate both inflammation and fibrosis in experimental pneumoconiosis, we incubated primary alveolar macrophages and lung fibroblasts isolated from silica-exposed rats with previously identified TAK1 inhibitor 5*Z*-7-oxozeaenol [31] at concentrations of 5, 10 and 20 μM, respectively. Results from ELISA showed that 5*Z*-7-oxozeaenol effectively decreased the level of p-TAK1 and inflammatory cytokines (IL-1β and TNF-α) in primary alveolar macrophages isolated from silica-exposed rats in dose-dependent manners (Supplementary Figure S4A). Meanwhile, we also observed that 5*Z*-7-oxozeaenol reduced levels of p-TAK1 and collagen subtypes (Col I and Col III) in primary lung fibroblasts isolated from silica-exposed rats (Supplementary Figure S4B). In addition, we also examined the cytotoxicity of 5*Z*-7-oxozeaenol in primary alveolar macrophages and lung fibroblasts isolated from healthy rats. MTT assay showed that there was no detectable cytotoxicity of 5*Z*-7-oxozeaenol in primary alveolar macrophages isolated from healthy rats, whereas we found obvious cytotoxicity of 5*Z*-7-oxozeaenol in primary lung fibroblasts isolated from healthy rats (Supplementary Figure S4C), implying that 5*Z*-7-oxozeaenol could induce potential side effect during treatment of pneumoconiosis. Collectively, it is urgent to screen alternative small molecules targeting TAK1 with both high efficiency and safety.

### Resveratrol screened as a potential small-molecule inhibitor for TAK1

To screen alternative small-molecule inhibitors for TAK1, we performed TAK1-based molecular docking. We retrieved the crystal structure of TAK1 (PDB code: 2eva) from the protein data bank and defined a docking pocket around the essential autophosphorylation site T187 on the activation loop of TAK1 [16]. Molecular docking was conducted between the defined pocket in TAK1 and more than 3 000 small molecules from commercially available databases and our established compound libraries. Considering binding free-energy (calculated as a parameter for evaluation of binding affinity) [32] and drug-like criteria, we selected the top 30 small molecules as candidates (Supplementary Table S5) for experimental selection. We incubated the candidates with primary alveolar macrophages and lung fibroblasts isolated from silica-exposed rats. ELISA results showed that a natural product resveratrol was the optimal small molecule, which significantly decreased levels of p-TAK1 in both primary alveolar macrophages and lung fibroblasts in dose-dependent manners (Supplementary Figure S5A and B). Meanwhile, the MTT assay showed that resveratrol had no detectable cytotoxicity in primary alveolar macrophages isolated from healthy rats and only slight cytotoxicity of resveratrol at a concentration of 100 μM was observed in fibroblasts isolated from healthy rats (Supplementary Figure S5C). In molecular docking, resveratrol (Supplementary Figure S5D) showed reasonable binding conformations with putative pocket of TAK1 and low binding free energies (Supplementary Figure S5E).

### *In vitro* effects of resveratrol on both inflammation and fibrosis in experimental pneumoconiosis

**Figure 4:**
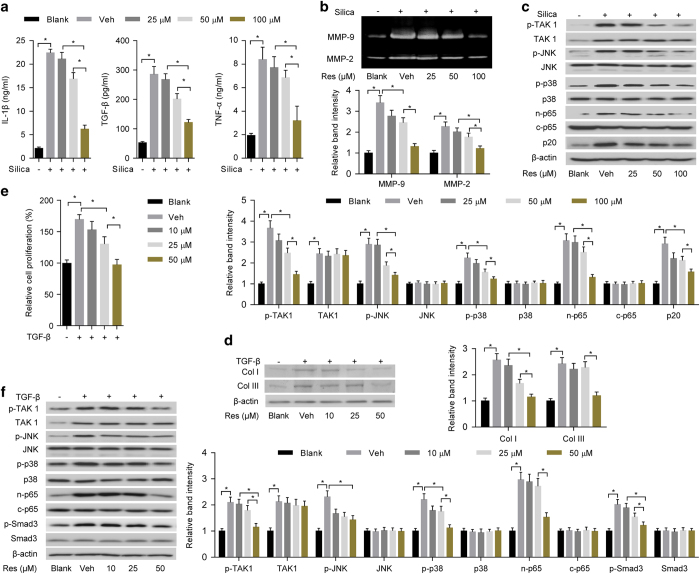
*In vitro* effects of resveratrol on inflammation and fibrosis. (**a**) ELISA to examine levels of IL-1β (left), TGF-β (middle) and TNF-α (right) in the supernatant of alveolar macrophage NR8383 cells with silica exposure and simultaneous administration of vehicle control (Veh: DMSO) or resveratrol (Res) at concentrations of 25, 50 and 100 μM, respectively. (**b**) Gelatin zymography (upper: representative images; bottom: relative bands intensity) to examine activities of matrix metalloproteinases (MMP-9 and MMP-2) in NR8383 cells with silica exposure and simultaneous administration of vehicle control (Veh: DMSO) or resveratrol (Res) at concentrations of 25, 50 and 100 μM, respectively. (**c**) Western blotting (upper: representative images; bottom: relative bands intensity) to examine TAK1 activation (p-TAK1) and inflammation-related downstream signaling in NR8383 cells with silica exposure and simultaneous administration of vehicle control (Veh: DMSO) or resveratrol (Res) at concentrations of 25, 50 and 100 μM, respectively. (**d**) Western blotting (left: representative images; right: relative bands intensity) to determine levels of Col I and Col III in lung fibroblast WI-38 cells with TGF-β-stimulation and simultaneous administration of vehicle control (Veh: DMSO) or resveratrol (Res) at concentrations of 10, 25 and 50 μM, respectively. (**e**) MTT to examine cell proliferation rate of WI-38 cells with TGF-β-stimulation and simultaneous administration of vehicle control (Veh: DMSO) or resveratrol (Res) at concentrations of 10, 25 and 50 μM, respectively. (**f**) Western blotting (left: representative images; right: relative bands intensity) to examine TAK1 activation (p-TAK1) and fibrosis-related downstream signaling in WI-38 cells with TGF-β stimulation and simultaneous administration of vehicle control (Veh: DMSO) or resveratrol (Res) at concentrations of 10, 25 and 50 μM, respectively. NR8383 and WI-38 cells without any treatment were served as blank controls. Data are presented as mean±s.d. *P<0.05, *n*=4 per group. One-way ANOVA with a *post hoc* test was performed and the statistical differences between the two groups were determined by the Student’s *t*-test.

To evaluate *in vitro* effects of resveratrol on inflammation, cultured alveolar macrophage NR8383 cells were exposed to silica and simultaneously treated with vehicle (dimethyl sulfoxide (DMSO)) or resveratrol at concentrations of 25, 50 and 100 μM, respectively. Compared with vehicle control, our results showed that resveratrol lowered inflammatory response in a dose-dependent manner, as indicated by the reduced level of inflammatory cytokines (IL-1β, TGF-β and TNF-α) and decreased activities of MMPs (MMP-9 and MMP-2) (Figure 4a and b). For TAK1 activation and inflammation-related downstream signaling, western blotting results showed that resveratrol decreased levels of p-TAK1, p-JNK, p-p38, n-p65 and p20 in a dose-dependent manner, whereas there was no obvious change of TAK1 in silica-exposed NR8383 cells treated with resveratrol (Figure 4c). To evaluate *in vitro* effects of resveratrol on fibrosis, lung fibroblast WI-38 cells were stimulated with TGF-β and simultaneously treated with vehicle (DMSO) or resveratrol at concentrations of 10, 25 and 50 μM, respectively. Compared with vehicle control, we found that resveratrol reduced levels of collagen subtypes (Col I and Col III) and cell proliferation rate in a dose-dependent manner (Figure 4d and e). For TAK1 activation and fibrosis-related downstream signaling, western blotting results showed that resveratrol decreased levels of p-TAK1, p-JNK, p-p38, n-p65 and p-Smad3 in a dose-dependent manner, whereas the level of TAK1 was not affected by resveratrol in TGF-β-stimulated WI-38 cells (Figure 4f).

To evaluate whether inhibition of TAK1 activation by resveratrol was responsible for attenuated inflammation and fibrosis in experimental pneumoconiosis, primary alveolar macrophages or lung fibroblasts isolated from silica-exposed rats were transfected with TAK1 overexpression vector or empty vector and then incubated with resveratrol or vehicle (DMSO) *in vitro*. Compared with vehicle control, our results showed that resveratrol reduced TAK1 overexpression-aggravated levels of inflammatory cytokines (IL-1β, TGF-β and TNF-α), activities of MMPs (MMP-9 and MMP-2), TAK1 activation (p-TAK1) and inflammation-related downstream signaling (p-JNK, p-p38, n-p65 and p20) in primary alveolar macrophages isolated from silica-exposed rats (Supplementary Figure S6A–C). In addition, resveratrol also decreased TAK1 overexpression-aggravated levels of collagen subtypes (Col I and Col III), cell proliferation rate, TAK1 activation (p-TAK1) and fibrosis-related downstream signaling (p-JNK, p-p38, n-p65 and p-Smad3) in primary lung fibroblasts isolated from silica-exposed rats (Supplementary Figure S6D–F).

### Validation of TAK1 as a molecular target of resveratrol and identification of key residues in TAK1

**Figure 5:**
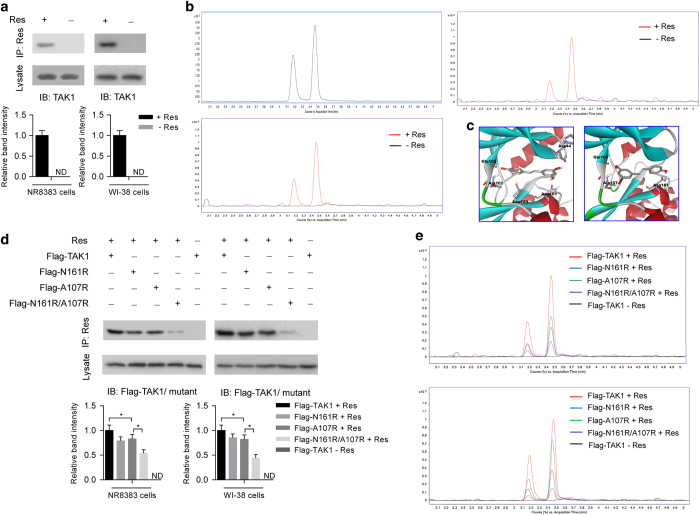
Validation of TAK1 as a molecular target of resveratrol and identification of key residues in TAK1. (**a**) Immunoprecipitation using anti-resveratrol antibody to examine the interaction between TAK1 and resveratrol (Res) in alveolar macrophage NR8383 (left) and lung fibroblasts WI-38 cells (right). TAK1 in the immunoprecipitate and lysate was examined by western blotting (upper: representative images; bottom: relative bands intensity). (**b**) Reference standard of resveratrol (left upper) and immunoprecipitation using anti-TAK1 antibody to examine the interaction between resveratrol and TAK1 in NR8383 (left bottom) and WI-38 cells (right). Resveratrol in the immunoprecipitate was examined by LC-MS/MS. There were two peaks for *trans*- and *cis*-resveratrol according to the reference standard. (**c**) Close-up view showing key residues on TAK1 in two predicted binding conformations between TAK1 and resveratrol in molecule docking. (**d**) Immunoprecipitation using anti-resveratrol antibody to examine the interaction between Flag-TAK1 wild-type or Flag-TAK1 mutants (N161R, A107R and N161R/A107R) and resveratrol in NR8383 (left) and WI-38 cells (right). The cells were transfected with pcDNA3.1-based expression vectors for Flag-TAK1 wild-type or Flag-TAK1 mutants (N161R, A107R and N161R/A107R). Flag-TAK1 or Flag-TAK1 mutants in immunoprecipitates and lysates was determined by western blotting (upper: representative images; bottom: relative bands intensity). (**e**) Immunoprecipitation using anti-Flag antibody to examine the interaction between resveratrol and Flag-TAK1 wild-type or Flag-TAK1 mutants in NR8383 cells (upper) and WI-38 cells (bottom). Resveratrol in immunoprecipitate was examined by LC-MS/MS. Experiments were performed at least three times. Res, resveratrol; ND, not detectable.

To validate putative interaction between TAK1 and resveratrol, we incubated alveolar macrophage NR8383 and lung fibroblast WI-38 cells with resveratrol or vehicle (DMSO) and then performed co-immunoprecipitation (co-IP). After co-IP with anti-resveratrol antibody, we detected the existence of TAK1 by western blotting in immunoprecipitates from NR8383 or WI-38 cells incubated with resveratrol (Figure 5a), whereas no TAK1 was detected in immunoprecipitates from NR8383 or WI-38 cells incubated with vehicle (Figure 5a). Consistently, after co-IP with anti-TAK1 antibody, we detected the existence of resveratrol by liquid chromatography-tandem mass spectrometry (LC-MS/MS) in the immunoprecipitates from NR8383 or WI-38 cells incubated with resveratrol (Figure 5b), whereas no resveratrol was detected in the immunoprecipitates from NR8383 and WI-38 cells incubated with vehicle (Figure 5b). To test whether the interaction between resveratrol and TAK1 was specific, we incubated NR8383 and WI-38 cells with a chalcone derivative (4′-hydroxychalcone), which had a similar chemical formula and molecule weight with resveratrol (Supplementary Figure S7A). After co-IP with anti-TAK1 antibody, no chalcone derivative (4′-hydroxychalcone) was detected in the immunoprecipitates from NR8383 and WI-38 cells incubated with chalcone derivative (4′-hydroxychalcone) or vehicle (Supplementary Figure S7B and C). All these results indicated that there was a specific interaction between resveratrol and TAK1 in both NR8383 and WI-38 cells.

To identify the key residues in TAK1, we analyzed the possible binding conformations between resveratrol and TAK1 in molecular docking. As shown in Figure 5c and Supplementary Figure S8A and B, amino-acid residues including Arg44 (R44), Leu163 (L163), Ala107 (A107), Asn (N161) and Glu105 (E105) in TAK1 were predicted to participate in resveratrol–TAK1 interaction from the two possible binding conformations. Thus, we chose them for further mutation studies *in vitro*. Both NR8383 and WI-38 cells were transfected with expression vectors for Flag-tagged TAK1 wild-type or mutants (TAK1-N161R, TAK1-A107R, TAK1-E105R, TAK1-R44A and TAK1-L163R) and then incubated with vehicle or resveratrol. After co-IP with anti-resveratrol antibody, we detected decreased levels of Flag-TAK1-N161R and Flag-TAK1-A107R in the immunoprecipitates from NR8383 and WI-38 cells by western blotting with anti-Flag antibody (Figure 5d), whereas there was no obvious change of Flag-TAK1-E105R, Flag-TAK1-R44A and Flag-TAK1-L163R (data not shown), when compared with Flag-TAK1 wild type. Furthermore, we also transfected NR8383 and WI-38 cells with expression vector for Flag-tagged TAK1 mutant containing both N161R and A107R (Flag-TAK1-N161R/A107R). We found that level of Flag-TAK1-N161R/A107R mutant decreased more obviously than either Flag-TAK1-N161R or Flag-TAK1-A107R mutant (Figure 5d). Consistently, after co-IP with anti-Flag antibody, we detected decreased levels of resveratrol by LC-MS/MS in the immunoprecipitates from NR8383 and WI-38 cells transfected with expression vectors for Flag-TAK1 mutants (N161R, A107R and N161R/A107R) when compared with those transfected with expression vectors for Flag-TAK1 wild type (Figure 5e). Furthermore, the level of resveratrol decreased more obviously in the immunoprecipitates from cells transfected with expression vector for Flag-TAK1-N161R/A107R mutant than those transfected with expression vector for either Flag-TAK1-N161R or Flag-TAK1-A107R mutant (Figure 5e). All these results indicated that both N161 and A107 residues in TAK1 were essential for resveratrol–TAK1 interaction.

### Prevention effects of resveratrol on inflammation and fibrosis in silica-exposed rats

**Figure 6:**
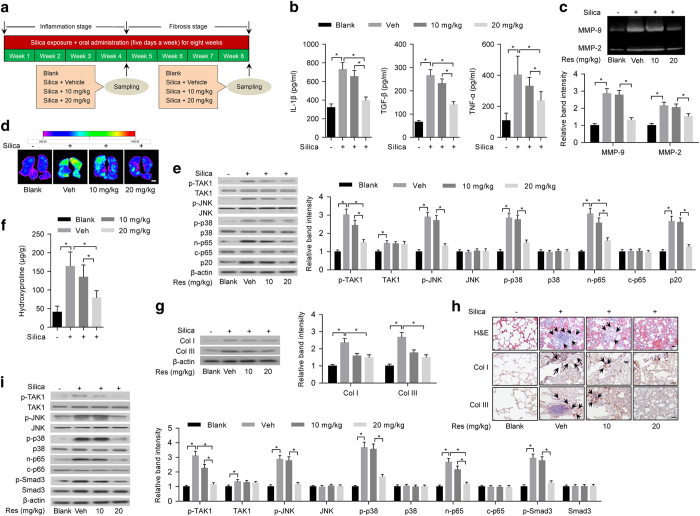
Prevention effects of resveratrol on inflammation and fibrosis in silica-exposed rats. (**a**) A schematic diagram illustrating the experimental design. Briefly, rats were exposed to silica aerosol and simultaneously administered with vehicle (Tween-80) or low-dose resveratrol (10 mg kg^−1^) or high-dose resveratrol (20 mg kg^−1^). After 20 days (4 weeks), the prevention effects of resveratrol on inflammation and related signaling pathway were examined (**b**–**e**), whereas the prevention effects of resveratrol on fibrosis and related signaling pathway were examined at 40 days (8 weeks) (**f**–**i**). (**b**) ELISA to determine levels of IL-1β (left), TGF-β (middle) and TNF-α (right) in the supernatant of BALF from rats with silica exposure and simultaneous administration of vehicle control (Veh: Tween-80), low-dose resveratrol (10 mg kg^−1^) or high-dose resveratrol (20 mg kg^−1^). (**c**) Gelatin zymography (upper: representative images; bottom: relative bands intensity) to examine activities of matrix metalloproteinases (MMP-9 and MMP-2) in homogenized lung tissues from rats with silica exposure and simultaneous administration of vehicle control (Veh: Tween-80), low-dose resveratrol (10 mg kg^−1^) or high-dose resveratrol (20 mg kg^−1^). (**d**) MMP-targeted NIR fluorescence imaging showing activities of matrix metalloproteinases in lung tissues from rats with silica exposure and simultaneous administration of vehicle control (Veh: Tween-80), low-dose resveratrol (10 mg kg^−1^) or high-dose resveratrol (20 mg kg^−1^). Scale bar, 1 cm. (**e**) Western blotting (left: representative images; right: relative bands intensity) to examine TAK1 activation (p-TAK1) and inflammation-related downstream signaling in primary alveolar macrophages isolated from lung tissues of rats with silica exposure and simultaneous administration of vehicle control (Veh: Tween-80), low-dose resveratrol (10 mg kg^−1^) or high-dose resveratrol (20 mg kg^−1^). (**f**) Hydroxyproline assay to determine total collagen levels in lung tissues from rats with silica exposure and simultaneous administration of vehicle control (Veh: Tween-80), low-dose resveratrol (10 mg kg^−1^) or high-dose resveratrol (20 mg kg^−1^). (**g**) Western blotting (left: representative images; right: relative bands intensity) to determine levels of Col I and Col III in lung tissue from rats with silica exposure and simultaneous administration of vehicle control (Veh: Tween-80), low-dose resveratrol (10 mg kg^−1^) or high-dose resveratrol (20 mg kg^−1^). (**h**) Histological examination to determine fibrotic nodule formation (arrows indicated in H&E staining) and collagen deposition (arrows indicated in immunohistochemical staining) in lung tissue from rats with silica exposure and simultaneous administration of vehicle control (Veh: Tween-80), low-dose resveratrol (10 mg kg^−1^) or high-dose resveratrol (20 mg kg^−1^). Scale bars, 50 μm. (**i**) Western blotting (left: representative images; right: relative bands intensity) to examine TAK1 activation (p-TAK1) and fibrosis-related downstream signaling in primary fibroblasts isolated from lung tissues of rats with silica exposure and simultaneous administration of vehicle control (Veh: Tween-80), low-dose resveratrol (10 mg kg^−1^) or high-dose resveratrol (20 mg kg^−1^). Rats without any treatment served as a blank control. Data are presented as mean±s.d. *P<0.05, *n*=9 per group. One-way ANOVA with a *post hoc* test was performed and the statistical differences between the two groups were determined by the Student’s *t*-test.

Sprague–Dawley rats were exposed to silica aerosol to induce experimental pneumoconiosis [33] and simultaneously administered with vehicle (Tween-80) or low-dose resveratrol (10 mg kg^−1^) or high-dose resveratrol (20 mg kg^−1^) (Figure 6a). After 20 days, we examined the prevention effects of resveratrol on inflammation in silica-exposed rats at early stage. Compared with silica-exposed rats administered with vehicle, we found that levels of inflammatory cytokines (IL-1β, TGF-β and TNF-α) in the supernatant of BALF determined by ELISA (Figure 6b) and activities of MMPs in lung tissues examined by gelatin zymography and MMP-targeted NIR fluorescence imaging (Figure 6c and d) were obviously lower in rats administered with high-dose resveratrol. Furthermore, we isolated primary alveolar macrophages and evaluated TAK1 activation (p-TAK1) and inflammation-related downstream signaling by western blotting. Consistently, the levels of p-TAK1, p-JNK, p-p38, n-p65 and p20 other than TAK1 were markedly lower in primary alveolar macrophages isolated from rats administered with high-dose resveratrol (Figure 6e). After 40 days, we examined the prevention effects of resveratrol on fibrosis in silica-exposed rats at late stage. We found that levels of collagens and collagen subtypes (Col I and Col III) in lung tissues determined by hydroxyproline assay and western blotting, respectively, were significantly lower in rats administered with high-dose resveratrol (*P*<0.05), when compared with rats treated with vehicle (Figure 6f and g). We also performed H&E staining and immunohistochemistry to examine fibrotic nodule formation and collagen deposition. Compared with rats administered with vehicle, we found that fibrotic nodule formation and deposition of Col I and Col III in lung tissues were obviously less in rats administered with high-dose resveratrol (Figure 6h). Further, we evaluated TAK1 activation (p-TAK1) and fibrosis-related downstream signaling in primary lung fibroblasts by western blotting. Our results showed that the levels of p-TAK1, p-JNK, p-p38, n-p65 and p-Smad3 other than TAK1 were markedly lower in rats administered with high-dose resveratrol, when compared with rats administered with vehicle (Figure 6i).

### Intervention effects of resveratrol on inflammation and fibrosis in silica-exposed rats

**Figure 7:**
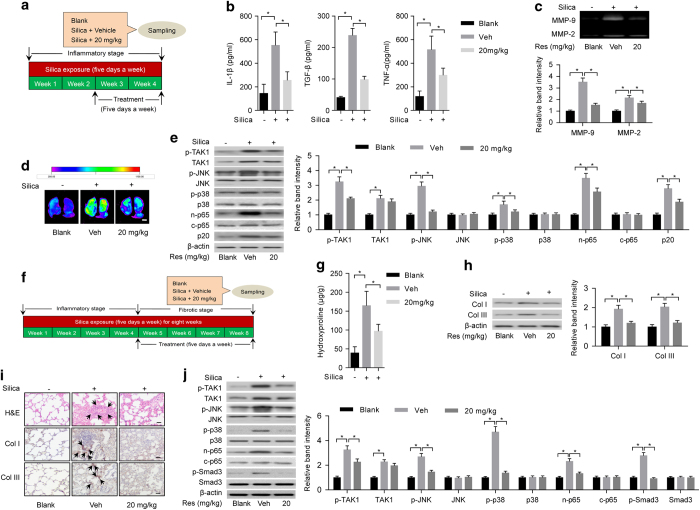
Intervention effects of resveratrol on inflammation and fibrosis in silica-exposed rats. (**a**) A schematic diagram illustrating the experimental design for the intervention study of resveratrol on inflammatory stage. Briefly, rats were exposed to silica aerosol for 10 days. Then, we administered the rats with resveratrol (20 mg kg^−1^) or vehicle (Tween-80) and simultaneously continued the silica exposure for another 10 days. (**b**) ELISA to determine levels of IL-1β (left), TGF-β (middle) and TNF-α (right) in the supernatant of BALF from silica-exposed rats with the administration of vehicle control (Veh: Tween-80) or resveratrol (20 mg kg^−1^). (**c**) Gelatin zymography (upper: representative images; bottom: relative bands intensity) to examine activities of matrix metalloproteinases (MMP-9 and MMP-2) in homogenized lung tissues from silica-exposed rats with the administration of vehicle control (Veh: Tween-80) or resveratrol (20 mg kg^−1^). (**d**) MMP-targeted NIR fluorescence imaging showing activities of matrix metalloproteinases in silica-exposed rats with the administration of vehicle control (Veh: Tween-80) or resveratrol (20 mg kg^−1^). Scale bar, 1 cm. (**e**) Western blotting (left: representative images; right: relative bands intensity) to examine TAK1 activation (p-TAK1) and inflammation-related downstream signaling in primary alveolar macrophages isolated from lung tissues of silica-exposed rats with treatment of vehicle control (Veh: Tween-80) or resveratrol (20 mg kg^−1^). (**f**) A schematic diagram illustrating the experimental design for the intervention study of resveratrol on fibrotic stage. Briefly, rats were exposed to silica aerosol for 20 days. Then, we administered the rats with 20 mg kg^−1^ resveratrol or vehicle (Tween-80) and simultaneously continued the silica exposure for another 20 days. (**g**) Hydroxyproline assay to determine levels of total collagen levels in lung tissues from silica-exposed rats with the administration of vehicle control (Veh: Tween-80) or resveratrol (20 mg kg^−1^). (**h**) Western blotting (left: representative images; right: relative bands intensity) to determine levels of Col I and Col III in lung tissue from silica-exposed rats with administration of vehicle control (Veh: Tween-80) or resveratrol (20 mg kg^−1^). (**i**) Histological examination to determine fibrotic nodule formation (arrows indicated in H&E staining) and collagen deposition (arrows indicated in immunohistochemical staining) in lung tissue from silica-exposed rats with treatment of vehicle control (Veh: Tween-80) or resveratrol (20 mg kg^−1^). Rats without any treatment served as blank controls. Scale bars, 50 μm. (**j**) Western blotting (left: representative images; right: relative bands intensity) to examine TAK1 activation (p-TAK1) and fibrosis-related downstream signaling in primary lung fibroblasts isolated from lung tissues of silica-exposed rats with administration of vehicle control (Veh: Tween-80) or resveratrol (20 mg kg^−1^). Data are presented as mean±s.d. *P<0.05, *n*=9 per group. One-way ANOVA with a *post hoc* test was performed and the statistical differences between the two groups were determined by the Student’s *t*-test.

To examine the intervention effects of resveratrol on inflammation in experimental pneumoconiosis, Sprague–Dawley rats were exposed to silica aerosol [33]. After 10 days of silica exposure, we administered the rats with resveratrol (20 mg kg^−1^) or vehicle (Tween-80) and simultaneously continued the silica exposure for another 10 days (Figure 7a). Compared with rats treated with vehicle, the levels of inflammatory cytokines (IL-1β, TGF-β and TNF-α) in the supernatant of BALF determined by ELISA (Figure 7b) and activities of MMPs in lung tissues determined by gelatin zymography and MMP-targeted NIR fluorescence imaging analysis (Figure 7c and d) decreased significantly in rats treated with resveratrol. Meanwhile, the levels of p-TAK1, p-JNK, p-p38, n-p65 and p20 other than TAK1 determined by western blotting decreased remarkably in primary alveolar macrophages isolated from rats treated with resveratrol when compared with rats treated with vehicle (Figure 7e). To evaluate the intervention effects of resveratrol on fibrosis in experimental pneumoconiosis, Sprague–Dawley rats were exposed to silica aerosol [33]. After 20 days of silica exposure, we administered the rats with 20 mg kg^−1^ resveratrol or vehicle (Tween-80) daily and simultaneously continued the silica exposure for another 20 days (Figure 7f). Compared with rats treated with vehicle, levels of total collagens (Figure 7g) and collagen subtypes (Col I and Col III) (Figure 7h) determined by hydroxyproline assay and western blotting, respectively, reduced significantly in lung tissues from rats treated with resveratrol (*P*<0.05). H&E staining and immunohistochemistry showed that there were less fibrotic nodule formation and deposition of Col I and Col III in lung tissues from rats treated with resveratrol (Figure 7i). The levels of p-TAK1, p-JNK, p-p38, n-p65 and p-Smad3 other than TAK1 determined by western blotting reduced markedly in primary lung fibroblasts isolated from rats treated with resveratrol (Figure 7j).

## Discussion

To date, TAK1 has emerged as an interesting therapeutic target in a series of disorders [17, 34, 35]. TAK1 signaling activation is essential for macrophage survival [36]. The role of TAK1 in fibrotic process has also been demonstrated in kidney fibrosis models [37]. However, association between TAK1 and pneumoconiosis was poorly understood. Our data have revealed that the levels of TAK1 and its active phosphorylated form (T187, p-TAK1) were aberrantly high in the lung specimens from pneumoconiosis patients as well as primary alveolar macrophages and fibroblasts from silica-exposed rats. After local delivery of CRISPR/Cas9 targeting *TAK1* in lungs, we found decreased inflammation and fibrosis in silica-exposed mice, which might be attributed to *TAK1* knockdown in alveolar macrophages (major source of proinflammatory cytokines and MMPs) [38] and fibroblasts (principal source of extensive extracellular matrix, such as collagens) [39] largely responsible for the above molecular events, as well as in other possible cells (lymphocytes and epithelial cells) involved in inflammatory cytokine production [40, 41]. By RNA interference and overexpression approaches, we validated the key role of TAK1 in regulating both macrophage-mediated inflammation and fibroblast-mediated fibrosis *in vitro*.

Regarding the key role of TAK1 in experimental pneumoconiosis, we speculated that pharmacologic inhibition of TAK1 might be effective in preventing both inflammation and fibrosis in pneumoconiosis. As expected, our results showed that the previously identified TAK1 small-molecule inhibitor 5*Z*-7-oxozeaenol [42, 43] markedly prevented inflammation in primary alveolar macrophages and fibrosis in lung fibroblasts isolated from silica-exposed rats, respectively. However, we also noticed that 5*Z*-7-oxozeaenol exhibited obvious cytotoxicity in primary lung fibroblasts isolated from healthy rats, as indicated by the MTT assay. These results contradicted previous studies, in which 5*Z*-7-oxozeaenol was reported to be sufficiently cytotoxic to tumor cells and less harmless to normal cells [35]. We speculated that the safety profile of 5*Z*-7-oxozeaenol was cell selective and there might be some unknown mechanisms underlying the cytotoxicity of 5*Z*-7-oxozeaenol in primary lung fibroblasts isolated from silica-exposed rats in our study.

To screen alternative small molecules for pharmacological inhibition of TAK1, we defined a docking pocket around T187 in TAK1, which was essential for autophosphorylation and activation of TAK1, and performed molecular docking. We found that resveratrol, a natural product derived from grape and Chinese herb *Polygonum cuspidatum* [44], most significantly inhibited active phosphorylated form of TAK1 (T187, p-TAK1) in both primary alveolar macrophages and lung fibroblasts isolated from silica-exposed rats. Remarkably, MTT assay showed that there was a favorable safety profile of resveratrol in primary alveolar macrophages and lung fibroblasts isolated from healthy rats, implying that resveratrol could be a promising alternative TAK1 inhibitor. In accordance with our molecular docking-based screening strategy, we validated that TAK1 was a molecular target of resveratrol. Moreover, mutation experiments showed that amino-acid residues in TAK1 including N161 and A107, which surrounded T187 of TAK1 in space conformation and were predicted in molecular docking, were essential for resveratrol–TAK1 interaction.

Consistent with the virtual screening data, our *in vitro* data demonstrated that the screened resveratrol effectively inhibited TAK1 activation, inflammatory response in cultured alveolar macrophage NR8383 cells with silica exposure and fibrotic response in cultured lung fibroblast WI-38 cells with TGF-β stimulation *in vitro*. Furthermore, resveratrol prevented TAK1 overexpression-aggravated inflammation in primary alveolar macrophages and fibrosis in primary lung fibroblasts isolated from silica-exposed rats. *In vivo* prevention and intervention experiments evidenced that resveratrol inhibited inflammation and fibrosis in silica-exposed rats with experimental pneumoconiosis, which could be explained by attenuated TAK1 activation mainly in alveolar macrophages and fibroblasts.

Although resveratrol has been extensively studied over the years, various pharmacological effects including antioxidant, anticarcinogenic, lifespan beneficial activities have been reported [45, 46]. The exact molecular mechanisms governing the therapeutic properties of resveratrol still remain elusive. Previously, sirtuin-1 (SIRT1), which is a NAD-dependent protein deacetylase [47], has been identified as a molecular target of resveratrol [48]. It has been reported that expression and activation of SIRT1 are downregulated in inflammation and fibrosis in liver and kidney diseases and resveratrol could activate SIRT1 and thus ameliorate inflammation and fibrosis [49,50,51,52]. Here, we also determined the expression of SIRT1 in lung specimens of pneumoconiosis patients, as well as primary alveolar macrophages and lung fibroblasts isolated from silica-exposed rats. Our results showed that there was no obvious change of SIRT1 expression and activation (Supplementary Figure S9), whereas TAK1 was highly expressed and activated in above lung specimens and primary cells, implying that TAK1 rather than SIRT1 was one of the specific therapeutic targets responsible for the inflammation and fibrosis in pneumoconiosis.

In conclusion, we revealed that TAK1 played a critical role in regulating both inflammation and fibrosis in pneumoconiosis and could be a potential therapeutic target for pneumoconiosis. Resveratrol identified as a newly TAK1 inhibitor was a promising agent for treatment of pneumoconiosis. Furthermore, TAK1 inhibition by resveratrol may also pave the way for treatment of other lung diseases, which are characterized with pulmonary inflammation and fibrosis.

## Materials and Methods

### Cell culture and reagents

Rat alveolar macrophage NR8383 cells (CRL-2192, ATCC, Manassas, VA, USA) were cultured at 37 °C in a humidified environment containing 5% CO_2_ in Ham’s F-12K (Kaighn’s) medium (Life Technologies, Grand Island, NY, USA), supplemented with 15% fetal bovine serum (FBS) (Life Technologies). Human lung fibroblast WI-38 cells (ATCC; no. CCL-75) were cultured at 37 °C in a humidified environment containing 5% CO_2_ in Eagle's minimum essential medium (ATCC) supplemented with 10% FBS and 100 U ml^–1^ penicillin–streptomycin (Life Technologies). Resveratrol with a purity ⩾99% (high-performance liquid chromatography (HPLC)) and 5*Z*-7-oxozeaenol with a purity ⩾98% (HPLC) were purchased from Sigma (St Louis, MO, USA). Chalcone derivative (4′-hydroxychalcone) with a purity ⩾98% (HPLC) was purchased from Santa Cruz Biotechnology (Dallas, TX, USA). Silica particles, silicon dioxide (SiO_2_), with an average diameter of 1.6 μm were purchased from US Silica MIN-U-SIL-5, US Silica, Shanghai, China. Human recombinant TGF-β (TGF-β1) was purchased from Merck Millipore (Danvers, MA, USA). Cross-species TAK1 siRNA and NC siRNA were purchased from Cell Signaling Technology (Danvers, MA, USA) and transfected into cells using the Lipofectamine 2000 (Invitrogen, Carlsbad, CA, USA), according to the manufacturer’s instructions. SIRT1 Activity Assay Kit was from Abcam (Cambridge, MA, USA).

### Microarray analysis

As there was no well-established human alveolar macrophage cell line, we chose rat alveolar macrophage NR8383 cells, which had been extensively used as *in vitro* inflammation model for pneumoconiosis [53], to induce inflammatory response. Briefly, NR8383 cells were seeded in 6-well plates with a density of 4×10^5^ cells per well and cultured overnight until 70–80% confluence. The cells were pretreated with 50 ng ml^−1^ lipopolysaccharides (Sigma) for 3 h and then incubated with 100 μg cm^−^^2^ silica particle for 12 h [24]. NR8383 cells incubated with vehicle (F-12K medium) were used as controls. Meanwhile, human lung fibroblast WI-38 cells were used to induce fibrotic response. Briefly, the WI-38 cells were seeded in 6-well plates with a density of 2×10^5^ cells per well and cultured overnight until 70–80% confluence. The cells were incubated with 10 ng ml^−1^ TGF-β for 48 h [25]. WI-38 cells incubated with vehicle (phosphate-buffered saline (PBS)) were used as controls. For microarray analysis, RNA samples were extracted from NR8383 cells incubated with vehicle (*n*=3) or silica (*n*=3) and WI-38 cells incubated with vehicle (*n*=3) or TGF-β (*n*=3), and then amplified by Agilent Quick Amp Labeling Kit (Agilent Technologies, Santa Clara, CA, USA). The Agilent GeneSpring GX V11.5.1 Software (Agilent Technologies, Santa Clara, CA, USA) was used for data normalization and analysis. Gene signatures were identified by fold-change screening. The statistical differences were determined by the Student’s *t*-test. Compared with vehicle controls, top 50 upregulated genes in silica-exposed NR8383 cells or TGF-β-stimulated WI-38 cells were selected.

### Subjects and fiberoptic bronchoscopy

We collected lung specimens from nine pneumoconiosis patients or six control individuals in collaborative hospitals. The subjects were diagnosed based on clinical signs, occupational history, X-ray exam, ILO International Classification of Radiographs of Pneumoconioses [14] and high-resolution computed tomography scan and further confirmed by pathological examination with lung biopsy specimens. Physiological limitation of pneumoconiosis patients included airflow limitation, hyperinflation, gas exchange disturbances, ventilatory muscle dysfunction, cardiovascular disturbances and reduced exercise capacity (Supplementary Table S4). Based on the above information, we concluded that the pneumoconiosis in our study was the result of exposure to silica. Lung specimens were obtained during lung biopsy [54]. Briefly, fiberoptic bronchoscopy was performed to obtain right lower lobe lung biopsy specimens, using small pinchers attached to a long cable threaded through the bronchoscope by a specialist. One to three biopsy specimens were obtained from each individual. The specimens were frozen and stored at −80 °C for further analysis by ELISA, western blotting and real-time PCR. This study was approved by the Medical Ethics Committees of Shenzhen People’s Hospital and Shenzhen Bao’an District People’s Hospital. We obtained informed consent from the subjects.

### Real-time PCR

RNeasy Mini Kit (Qiagen, Germantown, MD, USA) was used to extract total RNA using the commercialized protocol. Total RNA was reverse transcribed to cDNA using the SuperScript First-Strand Synthesis System (Thermo Fisher Scientific, Grand Island, NY, USA) according to the manufacturer’s instructions. Real-time PCR was performed using SYBR premix Ex Taq Kit (Takara, Kusatsu, Shiga, Japan) in a Real-Time PCR System (StepOnePlus; Applied Biosystems, Grand Island, NY, USA). ΔΔCT methods were used to evaluate mRNA expression of *IER3*, *MLLT11*, *TAK1*, *IL6* and *NACC2*. Glyceraldehyde 3-phosphate dehydrogenase (*GAPDH*) was used as an endogenous control for normalization. Expression of the overlapping genes in control individuals served as the calibrators. ΔΔCT=(CT_target_−CT_GAPDH_)_patients_−(CT_target_−CT_GAPDH_)_controls_. The primer pairs used in real-time PCR were synthesized in Sangon Biotech (Shanghai, China) and shown in Supplementary Table S6.

### Lentiviral vectors and sgRNA cloning

The U6-sgRNA-EFS-Cas9-2A-Cre (pSECC) lentiviral vector was constructed by assembling four parts with overlapping DNA ends using Gibson assembly [30]. Briefly, a 2.2 kb part (corresponding to the U6-Filler fragment from LentiCRISPR), a 0.3 kb part (corresponding to the EFS promoter from LentiCRISPR28), a 5.3 kb part (corresponding to a Cas9-2A-Cre fragment, which was generated by assembly PCR) and a 5.7 kb lentiviral backbone were assembled using Gibson assembly. For sgRNA cloning, the pSECC vector was digested with *Bsm*BI and ligated with *Bsm*BI-compatible annealed oligos [55]. The sgRNA sequences were designed by Feng Zhang’s laboratory at the Broad institute (sgTAK1-1: 5′-GGGACTTACTGGATTCAGGC-3′; sgTAK-2: 5′- CGGCGCTTCGATCATCTCAC-3′; sgTAK-3: 5′- GATGATCGAAGCGCCGTCGC-3′) [56]. An extra G (required for U6 transcriptional initiation) was added to the 5′ end of sgRNAs that lacked it. The pSECC lentiviral vector is available through Addgene (Cambridge, MA, USA).

### Lentiviral production and *in vivo* infection

Lentiviruses were produced by co-transfection of 293T cells with lentiviral backbone constructs and packaging vectors (delta8.2 and vesicular stomatitis virus G) using TransIT-LT1 (Mrius Bio, Madison, WI, USA). Supernatant was collected 48 and 72 h post-transfection, concentrated by ultracentrifugation at 25 000 r.p.m. for 90 min and resuspended in an appropriate volume of OptiMEM (Gibco, Grand Island, NY, USA). For *in vivo* infection, the lentiviruses (1.5×10^8^ IU per mouse) were intratracheally delivered into C57BL/6 mouse lungs [30, 57].

### SURVEYOR assay

Genomic region surrounding the CRISPR target site for each gene was PCR amplified, and products were purified using QiaQuick Spin Column (Qiagen) following the manufacturer’s protocol. A total of 400 ng of the purified PCR products were mixed with 2 μl 10x *Taq* polymerase PCR buffer (Enzymatics, Beverly, MA, USA) and ultrapure water to a final volume of 20 μl, and subjected to a reannealing process to enable heteroduplex formation. After reannealing, products were treated with SURVEYOR nuclease and SURVEYOR enhancer S (Transgenomics, Omaha, NE, USA), and analyzed on 4–20% Novex TBE polyacrylamide gels (Life Technologies). Gels were stained with SYBR Gold DNA stain (Life Technologies) and quantification was based on relative band intensities.

### Exposure of rodents to silica to induce experimental pneumoconiosis

Three-month old male C57/BL6 mice and 2-month-old male Sprague–Dawley rats were exposed to silica aerosol to induce experimental pneumoconiosis following established procedure [26, 27]. Briefly, C57/BL6 mice and Sprague–Dawley rats were housed using whole-body inhalation chambers (TIANJIN HOPE INDUSTRY & TRADE CO., LTD., Tianjin, China) and exposed to 15 mg m^−^^3^ silica or filtered-air (control). Exposures were conducted for 6 h per day (3 h x 2 times) with a 45-min interval for animal feeding, 5 days per week, for a total of 20 days (the inflammatory stage) or 40 days (the fibrotic stage). The rodents were on a 12-h light–dark schedule and were exposed during the dark cycle to coincide with their most active period.

### Isolation of primary alveolar macrophages

Primary alveolar macrophages were isolated from rodents following established protocols with minor modifications [58]. Briefly, mice and rats were killed and the lungs were carefully removed. The lung was cannulated via the trachea and BAL was performed using 10×5 ml ice-cold PBS. The lavage fluid was centrifuged at 400 *g* for 5 min and the pelleted cells were resuspended in PBS. Cell viability was determined by trypan blue exclusion. The cells were cultured in Ham’s F-12K medium supplemented with 15% FBS for 45 min at 37 °C and the non-adherent cells was washed away with PBS. The adherent cells were recovered and diluted as needed. Alveolar macrophages were identified as F4/80^+^ and CD11c^+^ cells by flow cytometry using anti-F4/80 (Abcam) and anti-CD11c antibody (Abcam) [59].

### Isolation of primary lung fibroblasts

Primary lung fibroblasts were isolated from rodents following established protocols with minor modifications [60]. Briefly, lungs were perfused through the right ventricle of the heart with PBS and then excised and homogenized with GentleMACS (Miltenyi Biotec, Bergisch Gladbach, Germany), followed by collagenase D (2 mg ml^−1^; Roche, Indianapolis, IN, USA) treatment for 1 h at 37 °C. The cell suspension was depleted of immune and endothelial cells by means of negative selection for CD45 (Miltenyi Biotec) and CD31 (BD Biosciences, San Jose, CA, USA) with magnetic cell sorting. The resulting cells were cultured in Eagle's minimum essential medium medium supplemented with 10% FBS, 100 U ml^–1^ penicillin–streptomycin, 1% l-glutamine, 1% sodium pyruvate and 1% nonessential amino acids (PAA Laboratories, Grand Island, NY, USA). Cells from passages 2 to 4 were used in our study.

### Inflammatory response in cultured alveolar macrophages

Cultured alveolar macrophage NR8383 cells were pretreated with 50 ng ml^−1^ lipopolysaccharides for 3 h and then incubated with 100 μg cm^−^^2^ silica particle to induce inflammatory response [24]. After 12 h, cell supernatants were collected and levels of inflammatory cytokines (IL-1β, TGF-β and TNF-α) were measured by ELISA Kits (IL-1β and TNF-α: R&D System, Minneapolis, MN, USA; TGF-β: eBioscience, San Diego, CA, USA). Activities of MMPs (MMP-2 and MMP-9) were determined by gelatin zymography according to established protocol. For drug administration, the cells were exposed to silica and simultaneously treated with small molecules at different concentrations. For genetic modulation of TAK1 by siRNA, the cells were incubated with siRNA for 36 h before the induction of inflammatory response by silica exposure.

### Inflammatory response in primary alveolar macrophages

Primary alveolar macrophages were cultured overnight in Ham’s F-12K medium supplemented with 15% FBS at 37 °C. Cell supernatants were collected and the levels of inflammatory cytokines (IL-1β, TGF-β and TNF-α) were measured by ELISA Kits. Activities of MMPs (MMP-2 and MMP-9) were determined by gelatin zymography according to established protocol. For drug administration, the cells were treated with small molecules at different concentrations for 12 h before the determination of inflammatory response including levels of inflammatory cytokines and activities of MMPs. For genetic modulation of TAK1, the cells were incubated with siRNA or expression vectors for 36 h before the determination of inflammatory response including levels of inflammatory cytokines and activities of MMPs.

### Fibrotic response in cultured lung fibroblasts

Cultured lung fibroblast WI-38 cells were incubated with 10 ng ml^−1^ TGF-β or PBS for 48 h. MTT was performed to evaluate cell proliferation rate according to the manufacturer’s instruction (Sigma-Aldrich). Western blotting was performed to analyze the expression levels of collagen subtypes including Col I and Col III. For drug administration, the cells were incubated with TGF-β and simultaneously treated with small molecules at different concentrations. For genetic modulation of TAK1 by siRNA, the cells were incubated with siRNA for 36 h before the induction of fibrotic response by TGF-β stimulation.

### Fibrotic response in cultured and primary lung fibroblasts

Primary lung fibroblasts were cultured in Eagle's minimum essential medium supplemented with 10% FBS and 100 U ml^–1^ penicillin–streptomycin. After 48 h, MTT was performed to evaluate the cell proliferation rate according to the manufacturer’s instruction (Sigma-Aldrich). Western blotting was performed to analyze the expression levels of collagen subtypes including Col I and Col III. For drug administration, the cells were treated with small molecules at different concentrations for 12 h before the determination of fibrotic response including cell proliferation rate and collagen subtypes. For genetic modulation of TAK1, the cells were incubated with siRNA or expression vectors for 36 h before the determination of fibrotic response including cell proliferation rate and collagen subtypes.

### TAK1-based molecular docking

Crystal structure of TAK1 (PDB code: 2eva) was retrieved from the protein data bank and a docking pocket around the essential autophosphorylation site T187 on activation loop of TAK1 was defined. More than 3 000 small molecules from commercially available database (e.g., NCI Diversity Set III library) and our established compound libraries were used for the TAK1-based molecular docking. Virtual screening parameters were prepared by autodock tools and used in AutoDock Vina. The small molecules were ranked with the consideration of binding free energies and drug-like (LipinskiScore=7) and lead-like (leadScore=5) filters.

### Interaction between TAK1 and resveratrol

A total of 5×10^6^ alveolar macrophage NR8383 cells or 1×10^6^ lung fibroblast WI-38 cells were seeded in 75 cm^2^ flasks and cultured overnight until 80–90% confluence 37 °C. The NR8383 cells were incubated with 100 μM resveratrol or chalcone derivative (4′-hydroxychalcone) and the WI-38 cells were incubated with 50 μM resveratrol or chalcone derivative (4′-hydroxychalcone). Both cells treated with DMSO were served as vehicle controls. Cells were harvested 12 h after drug administration and lysed. Immunoprecipitation followed with western blotting or LC-MS/MS was performed according to established protocol with minor modification [61]. To identify key residues in TAK1, 5×10^5^ alveolar macrophage NR8383 cells or 1×10^5^ lung fibroblast WI-38 cells were seeded in 100 cm^2^ dishes and cultured overnight until 50–60% confluence at 37 °C. Both cells were transfected with Flag-tagged TAK1 wild-type or mutants (TAK1-N161R, TAK1-A107R, TAK1-E105R, TAK1-R44A and TAK1-L163R). After 48 h, NR8383 cells were incubated with 100 μM resveratrol or DMSO and WI-38 cells were incubated with 50 μM resveratrol or DMSO. Cells were harvested 12 h after drug administration and lysed for immunoprecipitation followed with western blotting or LC-MS/MS.

### Immunoprecipitation followed with western blotting or LC-MS/MS

Cell lysates were prepared in HEPES lysis buffer (20 mM HEPES (pH 7.2), 50 mM NaCl, 0.5% Triton X-100, 1 mM NaF, 1 mM dithiothreitol) supplemented with protease inhibitor cocktail (Roche) and phosphatase inhibitors (10 mM NaF and 1 mM Na_3_VO_4_). Immunoprecipitations were performed using anti-resveratrol or anti-TAK1 (Cell Signaling Technology) or anti-Flag (Sigma) antibodies and protein A/G-agarose (Santa Cruz Biotechnology) at 4 °C. For detection of TAK1 or Flag-TAK1 wild type/mutants in both lysates and immunoprecipitates, anti-resveratrol or anti-TAK1 (Cell Signaling Technology) or anti-Flag (Sigma) primary antibodies and the appropriate secondary antibodies were used in western blotting, followed by detection with SuperSignal Chemiluminescence Kit (Pierce, Rockford, IL, USA). Anti-resveratrol antibody was generated according to previously established protocol [62]. For detection of resveratrol or chalcone derivative (4′-hydroxychalcone) after immunoprecipitations, small molecule was extracted by 90% methanol (Sigma) and examined by Agilent 6400 Ultra High-Performance Liquid Chromatograph with Tripe Qquadrupole Mass Spectrometer (Agilent Technologies, Santa Clara, CA, USA) [63]. Resveratrol with a purity ⩾99% (HPLC) (Sigma) and Chalcone derivative (4′-hydroxychalcone) with a purity 98% (HPLC) (Santa Cruz Biotechnology) were used as reference standards. Small molecules at the same retention time and same product ions in multiple reaction monitoring detection method [64] with the reference standards were identified as the target molecules.

### Construction of expression vectors

A cDNA construct containing the full-length open reading frame of TAK1 wild type was subcloned into mammalian expression vector pcDNA3.1 (Thermo Fisher Scientific) or 3x Flag-tagged mammalian expression vector pcDNA3.1 (Addgene) and verified by DNA sequencing analysis [65]. Site mutations including TAK1-N161R, TAK1-A107R, TAK1-E105R, TAK1-R44A and TAK1-L163R were made by site-directed PCR mutagenesis using QuikChange Mutagenesis Kit (Agilent Technologies) and verified by DNA sequencing analysis. The expression vectors were transfected into primary macrophages and fibroblast cells and normal culture cells by using Nucleofector Kits (Lonza, Basel, Switzerland) and Lipofectamine 2000 (Invitrogen), according to the manufacturer’s instructions, respectively.

### Animal handing

All the animals were housed in Laboratory Animal House of the Chinese University of Hong Kong with a temperature-controlled, 12 h light–dark cycle facility and food and water were available *ad libitum*. The animals were acclimatized to the laboratory conditions for at least 7 days before being used in experiments. The animals were killed by lethal dose of pentobarbital after treatment. The animal study procedures were approved by the Animal Experimentation Ethics Committee of the Chinese University of Hong Kong (Ref No.: 12/081/MIS).

### BALF analysis

Following established protocols [66], left lung of the rodents was ligated at the left main bronchus, BALF from the right lung was obtained by washing the airways three times with 5 ml of BALF buffer (composed of PBS with 5.5 mM d-glucose, pH at 7.4) through a tracheal cannula, BALF cell suspensions were centrifuged and the supernatants were collected. Levels of inflammatory cytokines including TNF-α, TGF-β and IL-1β were measured by ELISA testing.

### Histology and immunohistochemistry

The dissected left lungs were fixed in 10% formaldehyde and processed routinely for embedding in paraffin. Paraffin blocks were sectioned into 5 μm and mounted on glass slides for histological and immunohistochemical staining. H&E staining was performed on sections to examine the general morphology. Immunohistochemical staining was performed to detect Col I and Col III using anti-Col I (Abcam) and anti-Col III antibody (Abcam), respectively. The sections were counterstained with hematoxylin.

### Determination of lung collagen content

Hydroxyproline assay (Chondrex, Redmond, WA, USA) was performed to determine the collagen content in lung tissues. According to the instruction, dissected lung tissues were lysed and hydroxyproline content was quantified by spectrophotometer at 550 nm. The data were expressed as collagen per dry weight (μg g^−1^) [67].

### Gelatin zymography

Protein samples from cell supernatants or homogenized lung tissues were separated in 10% sodium dodecyl sulfate-polyacrylamide gel electrophoresis containing 1 mg ml^−1^ gelatin (Sigma) [68]. The gels were incubated in 2.5% Triton X-100 solution at room temperature with gentle agitation to remove sodium dodecyl sulfate and were soaked in reaction buffer (50 mM Tris-HCl (pH 7.5), 150 mM NaCl and 10 mM CaCl_2_) at 37 °C overnight [69]. After the reaction, the gels were stained for 1 h with staining solution (0.1% Coomassie Brilliant Blue, 30% methanol and 10% acetic acid) and were destained in the same solution, but without Coomassie Brilliant Blue. Gelatinolytic activity of MMPs were visualized as a clear band against a dark background of stained gelatin using ChemiDoc MP System (Bio-Rad, Hercules, CA, USA) and Image Lab program (Bio-Rad). The intensities of the MMPs bands were quantified and normalized to the corresponding controls.

### MMP-targeted NIR fluorescence imaging

Each mouse or rat was intravenously injected with 0.2 ml (4 nmol) MMP Sense680 Fluorescence Imaging Agent (Perkin-Elmer, Waltham, MA, USA) [70]. After 24 h, animals were killed and whole lung was dissected for *ex vivo* imaging with MS FX-Pro Imaging System (Carestream, Rochester, NY, USA). The parameters used in imaging acquisition were as following: peak emission filter, 700 nm; excitation filter, 650 nm; acquisition time, 30 s.

### ELISA testing

Levels of TNF-α and IL-1β were quantified using commercially available ELISA Kits (R&D Systems, Minneapolis, MN, USA) according to the manufacturer’s instructions. The level of TGF-β was quantified using a commercially available ELISA Kit (eBioscience, San Diego, CA, USA) according to the manufacturer’s instructions. The level of p-TAK1 was quantified using anti-p-TAK1 antibody (Elabscience, Wuhan, China) following established ELISA protocol (TECH TIP no. 65; Thermo Fisher Scientific). The level of SIRT1 was examined by a commercial ELISA Kit (Abcam).

### Western blot analysis

Proteins extracted from cells or tissues were quantified using Bradford Assay (Bio-Rad). Protein samples were separated by sodium dodecyl sulfate-polyacrylamide gel electrophoresis and transferred onto PVDF membranes (Bio-Rad). After blocking, the membranes were probed with primary antibodies and then incubated with specific horseradish peroxidase-conjugated secondary antibodies (Santa Cruz Biotechnology). Immunodetection was performed using enhanced chemiluminescence consistent with the manufacturer’s protocol (Thermo Fisher Scientific). Primary antibodies including anti-TAK1, anti-p-TAK1 (T187), anti-JNK, anti-p-JNK, anti-p38, anti-p-p38, anti-Smad3, anti-p-Smad3, anti-p65 and anti-β-actin were purchased from Cell Signaling Technology. Anti-p-TAK1 antibody (T184) was purchased from Thermo Fisher Scientific. Anti-Col I, anti-Col III and anti-SIRT1 were purchased from Abcam. Anti-p20 antibody was purchased from BioVision (Milpitas, CA, USA). The band intensities were quantified and normalized to the corresponding controls. β-Actin was used as a loading control for internal correction.

### Statistical analyses

All numerical data are expressed as the mean±s.d. One-way analysis of variance (ANOVA) with a *post hoc* test was performed and the statistical differences between the two groups were determined by the Student’s *t*-test. All statistical analyses were performed with SPSS software, version 22.0. *P*<0.05 was considered statistically significant. The analysis was performed by a contract service from Bioinformedicine (San Diego, CA, USA; http://www.bioinformedicine.com/index.php).
