# Supplementary material for: TAK1 inhibition attenuates both inflammation and fibrosis in experimental pneumoconiosis
Source: Cell Discov. 2017 Jul 11;3:17023–. doi: 10.1038/celldisc.2017.23 (PMC5504492; doi:10.1038/celldisc.2017.23)
Supplement: Supplementary information [file celldisc201723-s1.pdf]

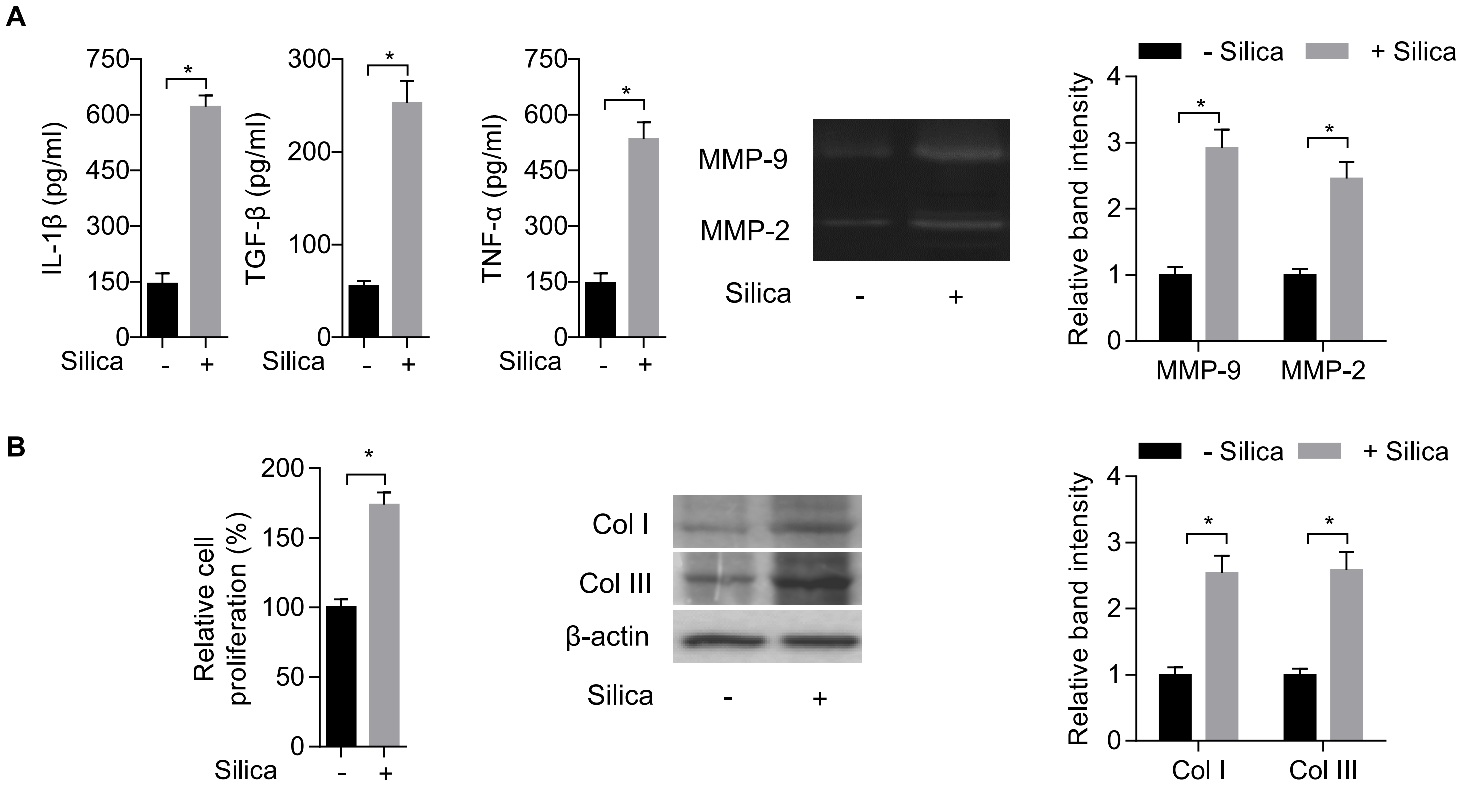
A
IL-1β (pg/ml)
Silica - +
TGF-β (pg/ml)
Silica - +
TNF-α (pg/ml)
Silica - +
MMP-9
MMP-2
Silica - +
- Silica
+ Silica
Relative band intensity
MMP-9
MMP-2
B
Relative cell proliferation (%)
Silica - +
Col I
Col III
β-actin
Silica - +
- Silica
+ Silica
Relative band intensity
Col I
Col III

Supplementary Figure S1 Inflammatory and fibrotic response in primary alveolar macrophages and fibroblasts, respectively (A) Levels of inflammatory cytokines (IL-1β, TGF-β and TNF-α) examined by ELISA (left) and MMPs (MMP-9 and MMP-2) analyzed by gelatin zymography (middle: representative images; right: relative bands intensity) in primary alveolar macrophages isolated from rats with (n = 5) or without silica-exposure (n = 5). **(B)** Cell proliferation rate examined by MTT assay (left) and collagen subtypes (Col I and Col III) determined by western blotting (middle: representative images; right: relative bands intensity) in primary lung fibroblasts isolated from rats with (n = 5) or without silica-exposure (n = 5). Data are presented as mean ± s.d. *$P < 0.05$. One-way analysis of variance (ANOVA) with a *post-hoc* test was performed and the statistical differences between the two groups were determined by the Student's *t* test.

A

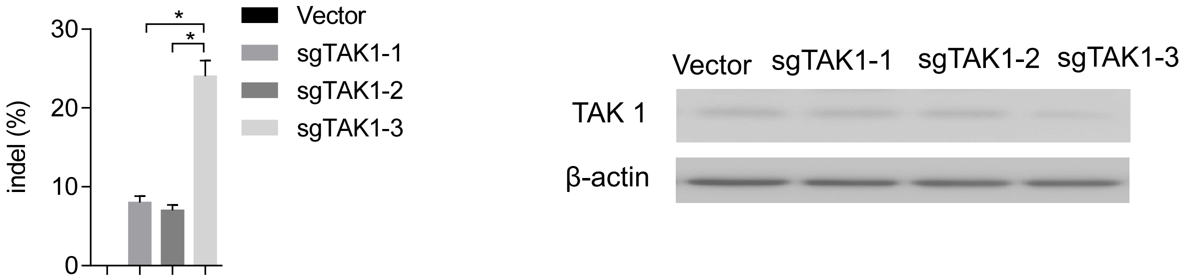

indel (%)
30
20
10
0
*
*
Vector
sgTAK1-1
sgTAK1-2
sgTAK1-3
Vector
sgTAK1-1
sgTAK1-2
sgTAK1-3
TAK 1
β-actin

B

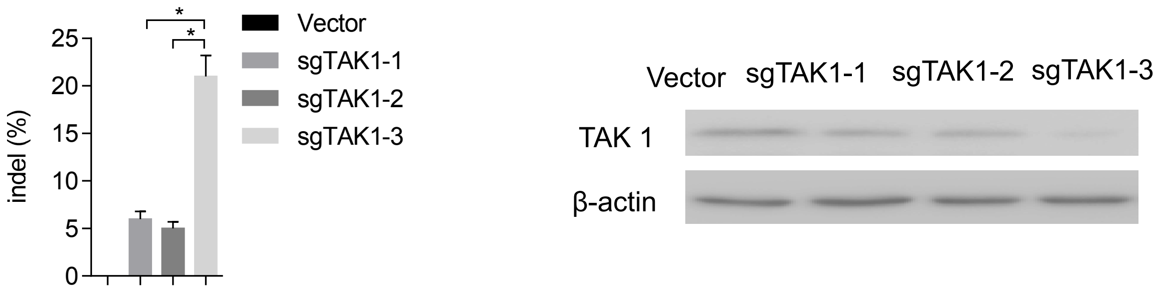

indel (%)
25
20
15
10
5
0
*
*
Vector
sgTAK1-1
sgTAK1-2
sgTAK1-3
Vector
sgTAK1-1
sgTAK1-2
sgTAK1-3
TAK 1
β-actin

C

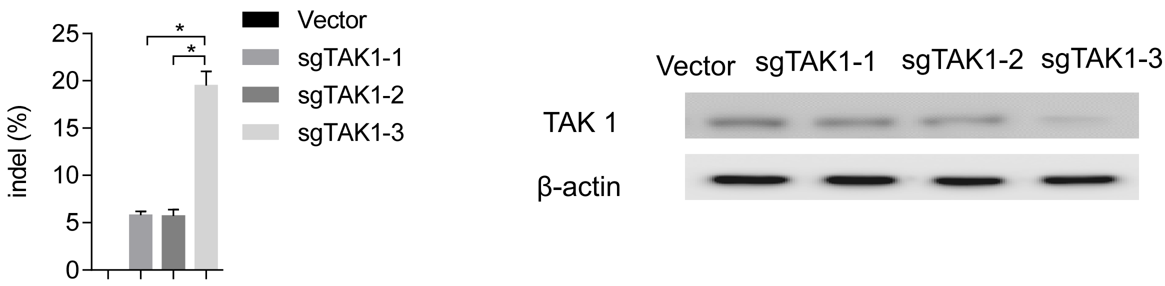

indel (%)
25
20
15
10
5
0
*
*
Vector
sgTAK1-1
sgTAK1-2
sgTAK1-3
Vector
sgTAK1-1
sgTAK1-2
sgTAK1-3
TAK 1
β-actin

Supplementary Figure S2 TAK1 gene knockdown efficiency of lentiviruses expressing CRISPR/Cas9 systems (A) SURVEYOR assay (left) and western blotting (right) to determine the TAK1 knockdown efficiency of different CRISPR/Cas9 systems in primary alveolar macrophages *in vitro*. **(B)** SURVEYOR assay (left) and western blotting (right) to determine the TAK1 knockdown efficiency of different CRISPR/Cas9 systems in primary lung fibroblasts *in vitro*. **(C)** SURVEYOR assay (left) and western blotting (right) to determine the TAK1 knockdown efficiency of different CRISPR/Cas9 systems in lung tissues *in vivo*. The lentiviruses were intratracheally delivered into C57BL/6 mouse lungs once a week. Four weeks of post-infection, TAK1 knockdown efficiency were determined by SURVEYOR and western blotting. n = 6 per group. Data are presented as mean ± s.d. $^*P < 0.05$ vs corresponding group. One-way analysis of variance (ANOVA) with a post-hoc test was performed and the statistical differences between the two groups were determined by the Student’s t test.

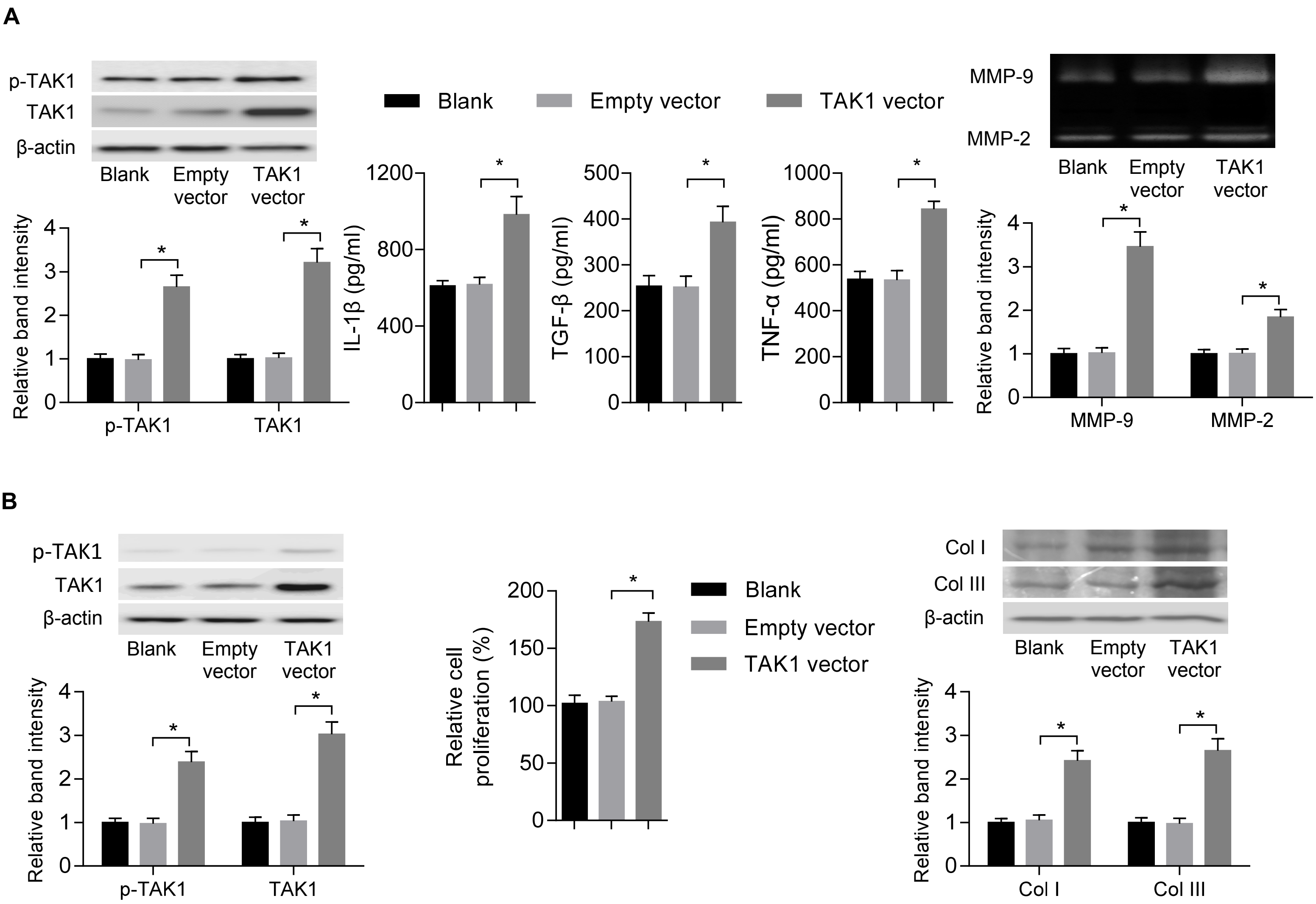
A
p-TAK1
TAK1
β-actin
Blank
Empty vector
TAK1 vector
Relative band intensity
IL-1β (pg/ml)
TGF-β (pg/ml)
TNF-α (pg/ml)
MMP-9
MMP-2
B
Relative cell proliferation (%)
Col I
Col III

Supplementary Figure S3 ***In vitro*** **effects of TAK1 overexpression on inflammation and fibrosis (A)** Levels of p-TAK1 and TAK1 determined by western blotting (left upper: representative images; left bottom: relative bands intensity), inflammatory cytokines (IL-1β, TGF-β and TNF-α) examined by ELISA (middle) and MMPs (MMP-9 and MMP-2) analyzed by gelatin zymography (right upper: representative images; right bottom: relative bands intensity) in primary alveolar macrophages with incubation of empty vector or expression vector for TAK1 (TAK1 vector). Primary alveolar macrophages were isolated from silica-exposed rats. **(B)** Levels of p-TAK1 and TAK1 determined by western blotting (left upper: representative images; left bottom: relative bands intensity), cell proliferation rate examined by MTT assay (middle) and collagen subtypes (Col I and Col III) determined by western blotting (right upper: representative images; right bottom: relative bands intensity) in primary lung fibroblasts with incubation of empty vector or expression vector for TAK1 (TAK1 vector). Primary lung fibroblasts were isolated from silica-exposed rats. Data are presented as mean ± s.d. *$P < 0.05$, n = 5 per group. One-way analysis of variance (ANOVA) with a *post-hoc* test was performed and the statistical differences between the two groups were determined by the Student's *t* test.

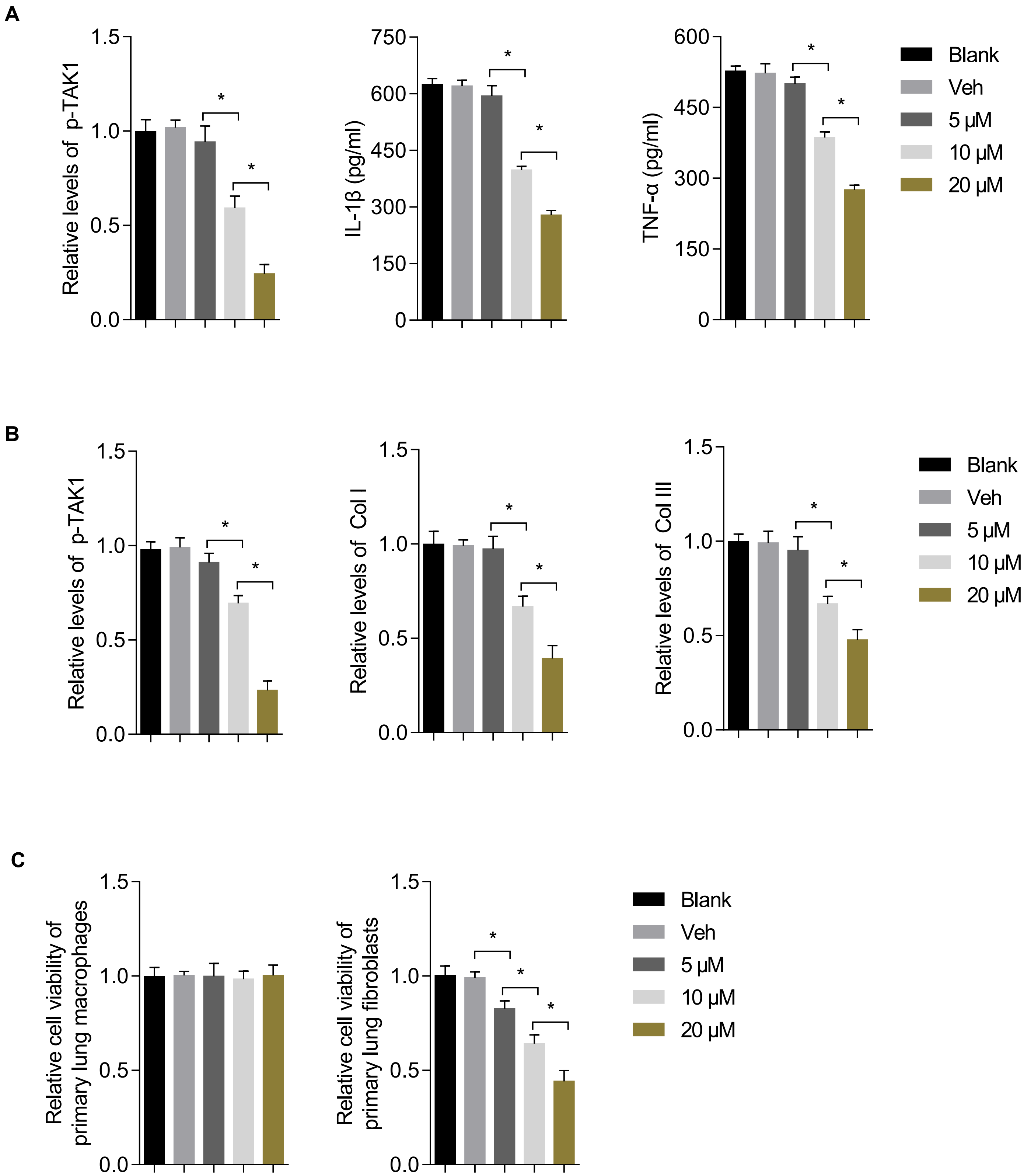
A
Relative levels of p-TAK1
IL-1β (pg/ml)
TNF-α (pg/ml)
Blank
Veh
5 μM
10 μM
20 μM
B
Relative levels of p-TAK1
Relative levels of Col I
Relative levels of Col III
Blank
Veh
5 μM
10 μM
20 μM
C
Relative cell viability of primary lung macrophages
Relative cell viability of primary lung fibroblasts
Blank
Veh
5 μM
10 μM
20 μM

Supplementary Figure S4 *In vitro* effects of TAK1 inhibitor 5Z-7-oxozeaenol on inflammation and fibrosis and cytotoxicity (A) ELISA to examine levels of p-TAK1 and supernatant inflammatory cytokines (IL-1β and TNF-α) in primary alveolar macrophages with incubation of vehicle control (Veh: DMSO) or TAK1 inhibitor 5Z-7-oxozeaenol at concentrations of 5, 10 and 20 μM, respectively. Primary alveolar macrophages were isolated from silica-exposed rats. **(B)** ELISA to examine levels of p-TAK1 and collagen subtypes (Col I and Col III) in primary lung fibroblasts with incubation of vehicle control (Veh: DMSO) or TAK1 inhibitor 5Z-7-oxozeaenol at concentrations of 5, 10 and 20 μM, respectively. Primary lung fibroblasts were isolated from silica-exposed rats. **(C)** MTT assay to examine cytotoxicity of primary alveolar macrophages and lung fibroblasts with incubation of vehicle control (Veh: DMSO) or TAK1 inhibitor 5Z-7-oxozeaenol at concentrations of 5, 10 and 20 μM, respectively. Primary alveolar macrophages and lung fibroblasts were isolated from healthy rats. Data are presented as mean ± s.d. *$P < 0.05$, n = 5 per group. One-way analysis of variance (ANOVA) with a *post-hoc* test was performed and the statistical differences between the two groups were determined by the Student's *t* test.

A

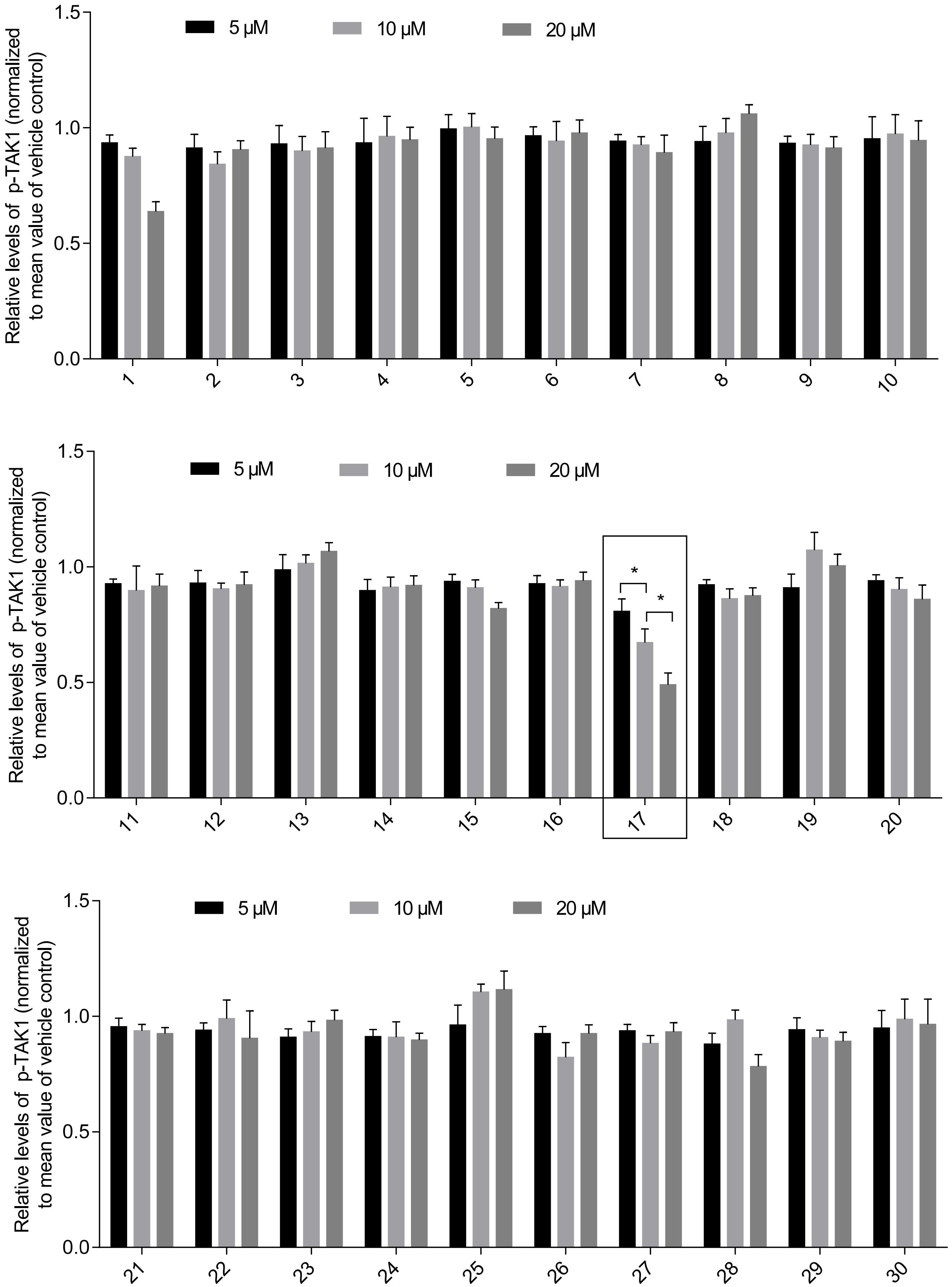

1.5
1.0
0.5
0.0
5 µM
10 µM
20 µM
Relative levels of p-TAK1 (normalized to mean value of vehicle control)
1
2
3
4
5
6
7
8
9
10
11
12
13
14
15
16
17
18
19
20
*
*
21
22
23
24
25
26
27
28
29
30

B

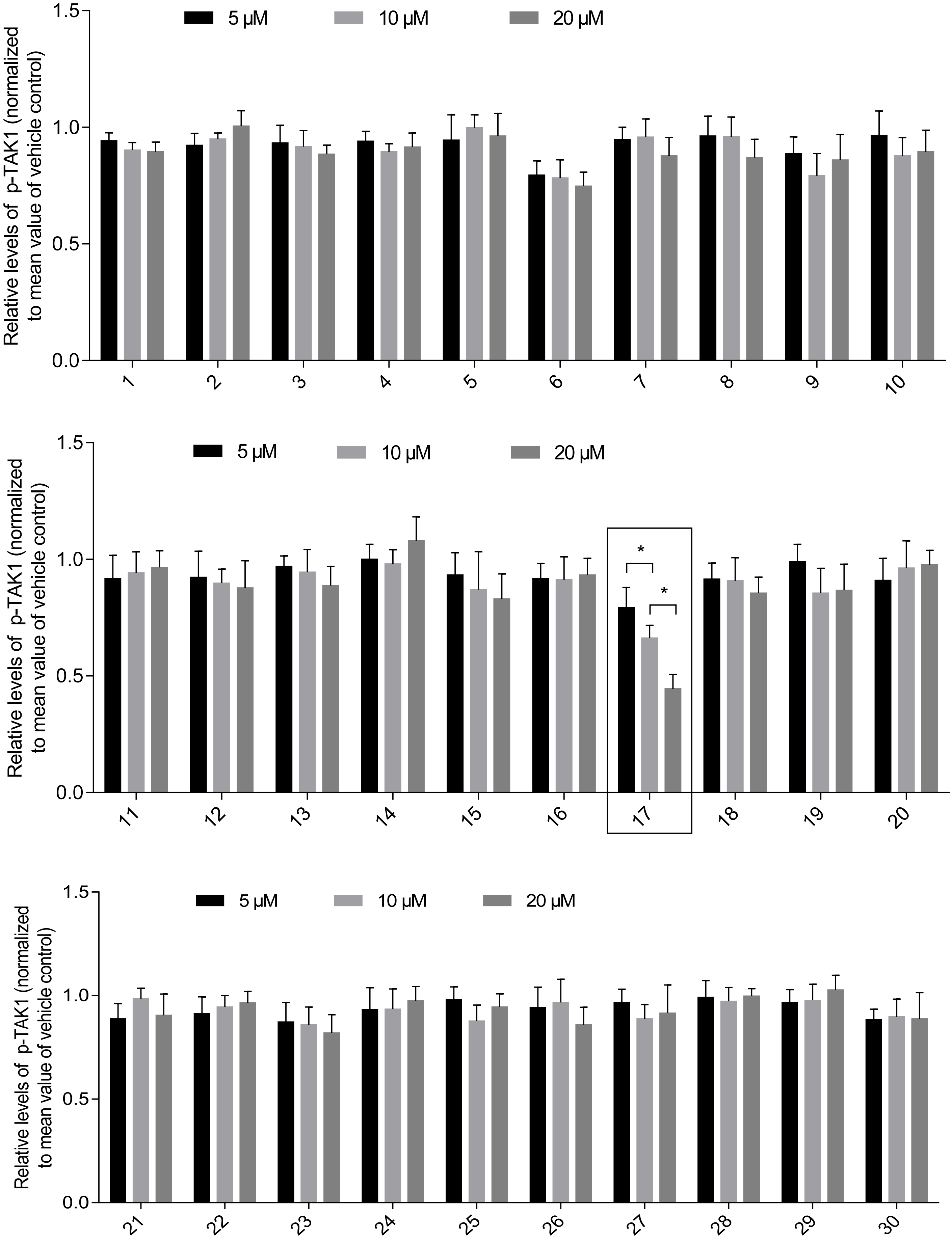
5 μM
10 μM
20 μM
Relative levels of p-TAK1 (normalized to mean value of vehicle control)
1.5
1.0
0.5
0.0
1
2
3
4
5
6
7
8
9
10
11
12
13
14
15
16
17
18
19
20
*
*
21
22
23
24
25
26
27
28
29
30

C

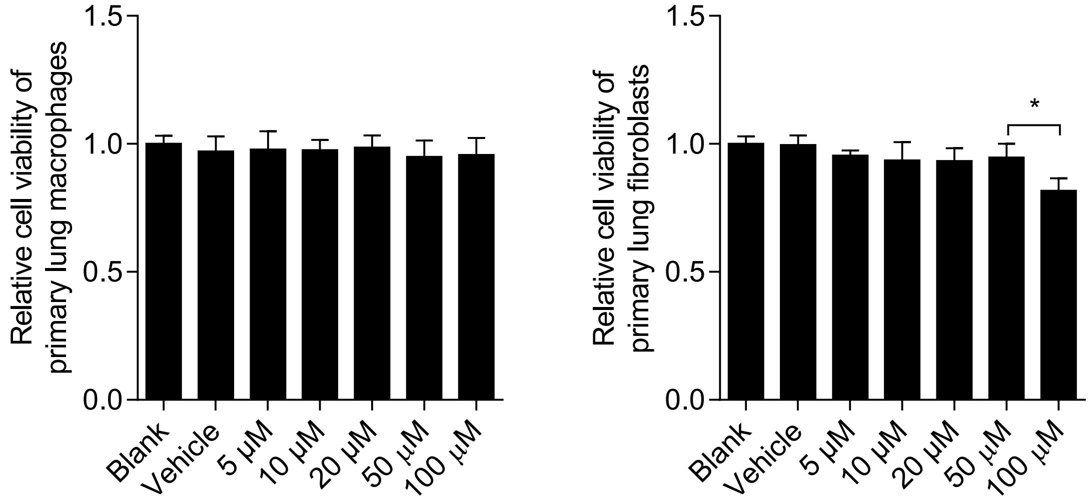
Relative cell viability of primary lung macrophages
1.5
1.0
0.5
0.0
Blank
Vehicle
5 μM
10 μM
20 μM
50 μM
100 μM
Relative cell viability of primary lung fibroblasts
*

D

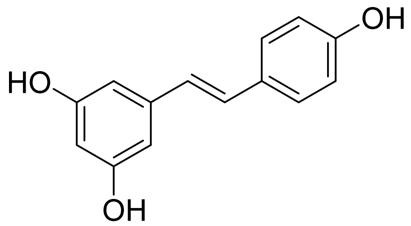
HO
OH
OH

E

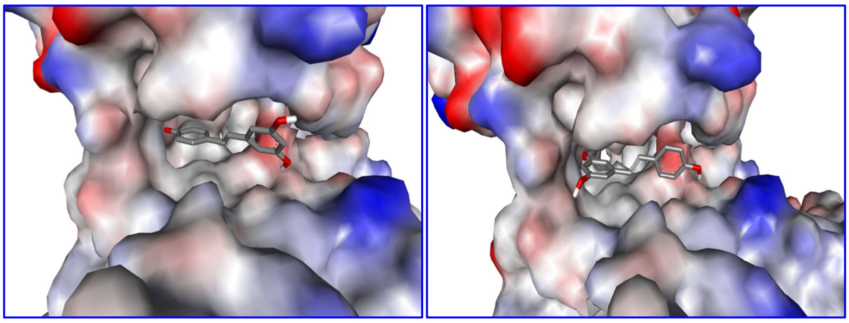

Supplementary Figure S5 Screening of TAK1 small molecule inhibitors (A) ELISA to examine levels of p-TAK1 in primary alveolar macrophages with incubation of vehicle control (DMSO) or 30 TAK1 inhibitor candidates at concentrations of 5, 10 and 20 μM, respectively. Primary alveolar macrophages were isolated from silica-exposed rats. **(B)** ELISA to examine levels of p-TAK1 in primary lung fibroblasts with incubation of vehicle control (DMSO) or 30 TAK1 inhibitor candidates at concentrations of 5, 10 and 20 μM, respectively. Primary lung fibroblasts were isolated from silica-exposed rats. **(C)** MTT assay to examine cytotoxicity of primary alveolar macrophages and lung fibroblasts with incubation of vehicle control (DMSO) or resveratrol at concentrations of 5, 10 and 20 μM, respectively. Primary alveolar macrophages and lung fibroblasts were isolated from healthy rats. **(D)** Formula of resveratrol. **(E)** Close-up view showing the predicted two conformations between TAK1 and resveratrol with low binding free energies (left: -6.46; right: -6.03) in molecular docking. Data are presented as mean ± s.d. $^*P < 0.05$, n = 5 per group. One-way analysis of variance (ANOVA) with a *post-hoc* test was performed and the statistical differences between the two groups were determined by the Student's *t* test.

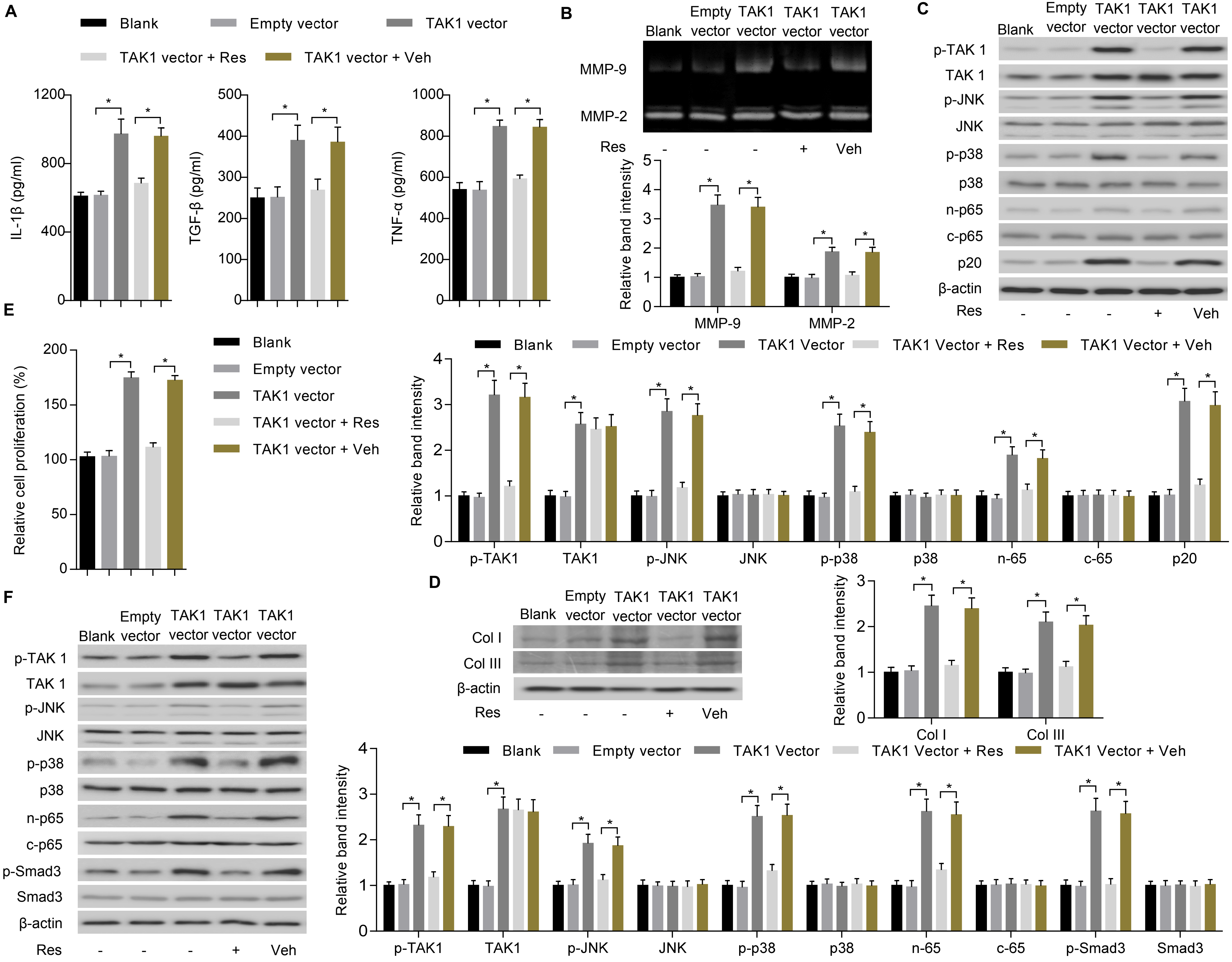
A
Blank
Empty vector
TAK1 vector
TAK1 vector + Res
TAK1 vector + Veh
IL-1β (pg/ml)
TGF-β (pg/ml)
TNF-α (pg/ml)
B
Blank
Empty vector
TAK1 vector
TAK1 vector
TAK1 vector
MMP-9
MMP-2
Res
-
-
-
+
Veh
Relative band intensity
MMP-9
MMP-2
C
Blank
Empty vector
TAK1 vector
TAK1 vector
TAK1 vector
p-TAK 1
TAK 1
p-JNK
JNK
p-p38
p38
n-p65
c-p65
p20
β-actin
Res
-
-
-
+
Veh
E
Relative cell proliferation (%)
Blank
Empty vector
TAK1 vector
TAK1 vector + Res
TAK1 vector + Veh
Blank
Empty vector
TAK1 Vector
TAK1 Vector + Res
TAK1 Vector + Veh
Relative band intensity
p-TAK1
TAK1
p-JNK
JNK
p-p38
p38
n-65
c-65
p20
D
Blank
Empty vector
TAK1 vector
TAK1 vector
TAK1 vector
Col I
Col III
β-actin
Res
-
-
-
+
Veh
Relative band intensity
Col I
Col III
F
Blank
Empty vector
TAK1 vector
TAK1 vector
TAK1 vector
p-TAK 1
TAK 1
p-JNK
JNK
p-p38
p38
n-p65
c-p65
p-Smad3
Smad3
β-actin
Res
-
-
-
+
Veh
Blank
Empty vector
TAK1 Vector
TAK1 Vector + Res
TAK1 Vector + Veh
Relative band intensity
p-TAK1
TAK1
p-JNK
JNK
p-p38
p38
n-65
c-65
p-Smad3
Smad3

Supplementary Figure S6 *In vitro* effects of resveratrol on TAK1 overexpression-aggravated inflammation and fibrosis (A) ELISA to examine levels of IL-1β (left), TGF-β (middle) and TNF-α (right) in supernatant of TAK1-overexpressed primary alveolar macrophages with treatment of 100 μM resveratrol (Res) or vehicle control (Veh). **(B)** Gelatin zymography (upper: representative images; bottom: relative bands intensity) to examine activities of matrix metalloproteinases (MMP-9 and MMP-2) in TAK1-overexpressed primary alveolar macrophages with treatment of 100 μM resveratrol (Res) or vehicle control (Veh). **(C)** Western blotting (upper: representative images; bottom: relative bands intensity) to examine TAK1 activation (p-TAK1) and inflammation-related downstream signaling in TAK1-overexpressed primary alveolar macrophages with treatment of 100 μM resveratrol (Res) or vehicle control (Veh). **(D)** Western blotting (left: representative images; right: relative bands intensity) to determine levels of Col I and Col III in TAK1-overexpressed primary lung fibroblasts with treatment of 50 μM resveratrol (Res) or vehicle control (Veh). **(E)** MTT assay to examine cell proliferation rate of TAK1-overexpressed primary lung fibroblasts with treatment of 50 μM resveratrol (Res) or vehicle control (Veh). **(F)** Western blotting (left: representative images; right: relative bands intensity) to examine TAK1 activation (p-TAK1) and fibrosis-related downstream signaling in TAK1-overexpressed primary lung fibroblast with treatment of 50 μM resveratrol (Res) or vehicle control (Veh). Primary alveolar macrophages and lung fibroblasts were isolated from silica-exposed rats. Vector: pcDNA3.1 empty vector; Vector-TAK1: pcDNA3.1-based expression vector for TAK1. Data are presented as mean ± s.d. *$P < 0.05$, n = 4 per group. One-way analysis of variance (ANOVA) with a *post-hoc* test was performed and the statistical differences between the two groups were determined by the Student's *t* test.

A

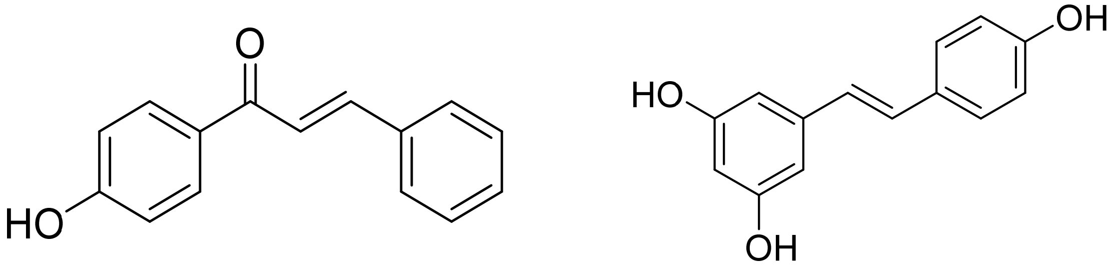
O
HO
HO
OH
OH

B

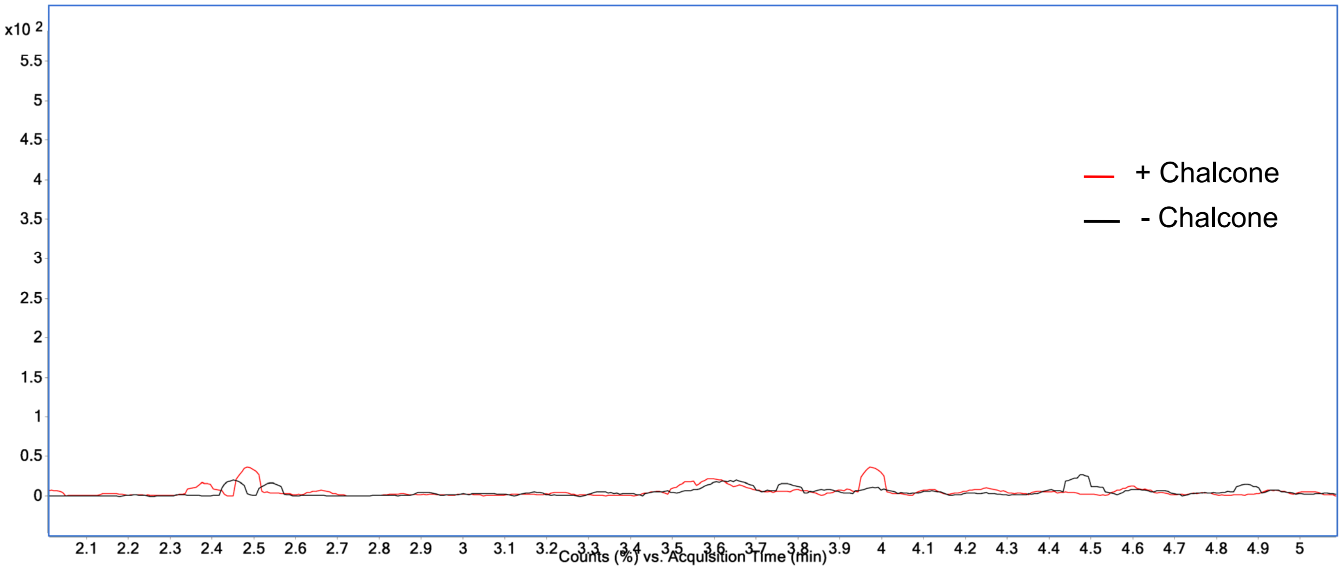
x10 2
5.5
5
4.5
4
3.5
3
2.5
2
1.5
1
0.5
0
+ Chalcone
- Chalcone
2.1 2.2 2.3 2.4 2.5 2.6 2.7 2.8 2.9 3 3.1 3.2 3.3 3.4 3.5 3.6 3.7 3.8 3.9 4 4.1 4.2 4.3 4.4 4.5 4.6 4.7 4.8 4.9 5
Counts (%) vs. Acquisition Time (min)

C

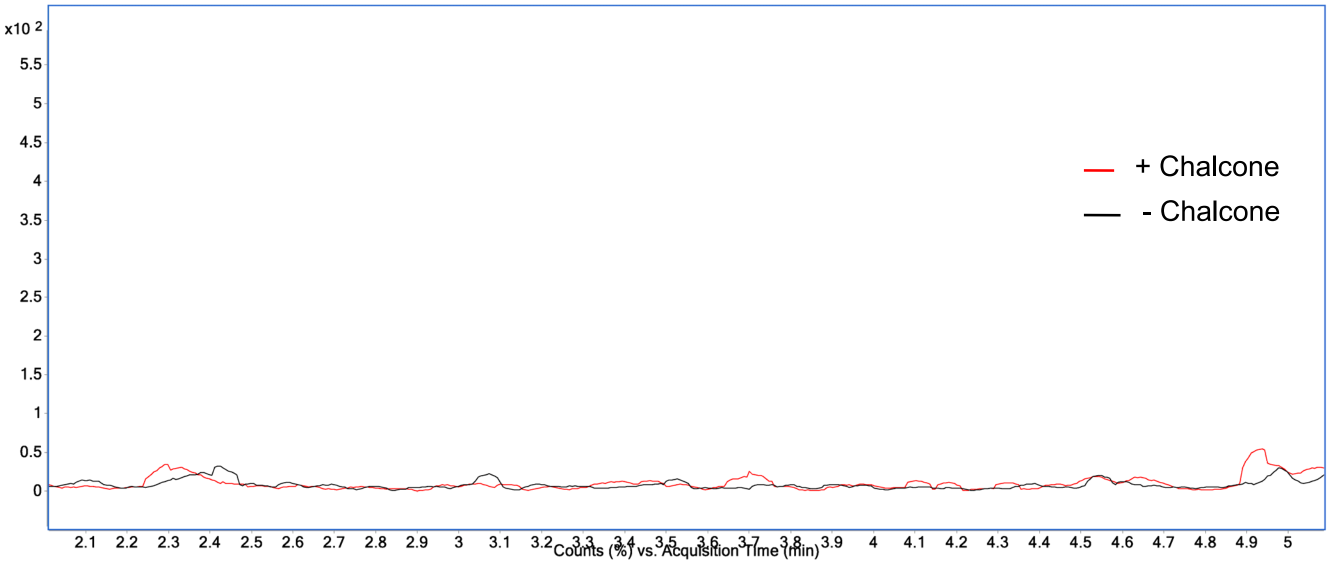
x10 2
5.5
5
4.5
4
3.5
3
2.5
2
1.5
1
0.5
0
+ Chalcone
- Chalcone
2.1 2.2 2.3 2.4 2.5 2.6 2.7 2.8 2.9 3 3.1 3.2 3.3 3.4 3.5 3.6 3.7 3.8 3.9 4 4.1 4.2 4.3 4.4 4.5 4.6 4.7 4.8 4.9 5
Counts (%) vs. Acquisition Time (min)

Supplementary Figure S7 Binding assay of chalcone derivative with TAK1 by immunoprecipitation followed with LC-MS/MS (A) Formula of chalcone derivative (4'-hydroxychalcone) (left) and resveratrol (right). **(B)** Immunoprecipitation using anti-TAK1 antibody to examine the interaction between chalcone derivative (4'-hydroxychalcone) and TAK1 in NR8383 cells. **(C)** Immunoprecipitation using anti-TAK1 antibody to examine the interaction between chalcone derivative (4'-hydroxychalcone) and TAK1 in WI-38 cells. Chalcone derivative (4'-hydroxychalcone) in immunoprecipitate was examined by LC-MS/MS. Chalcone: chalcone derivative (4'-hydroxychalcone). Experiments were performed three times.

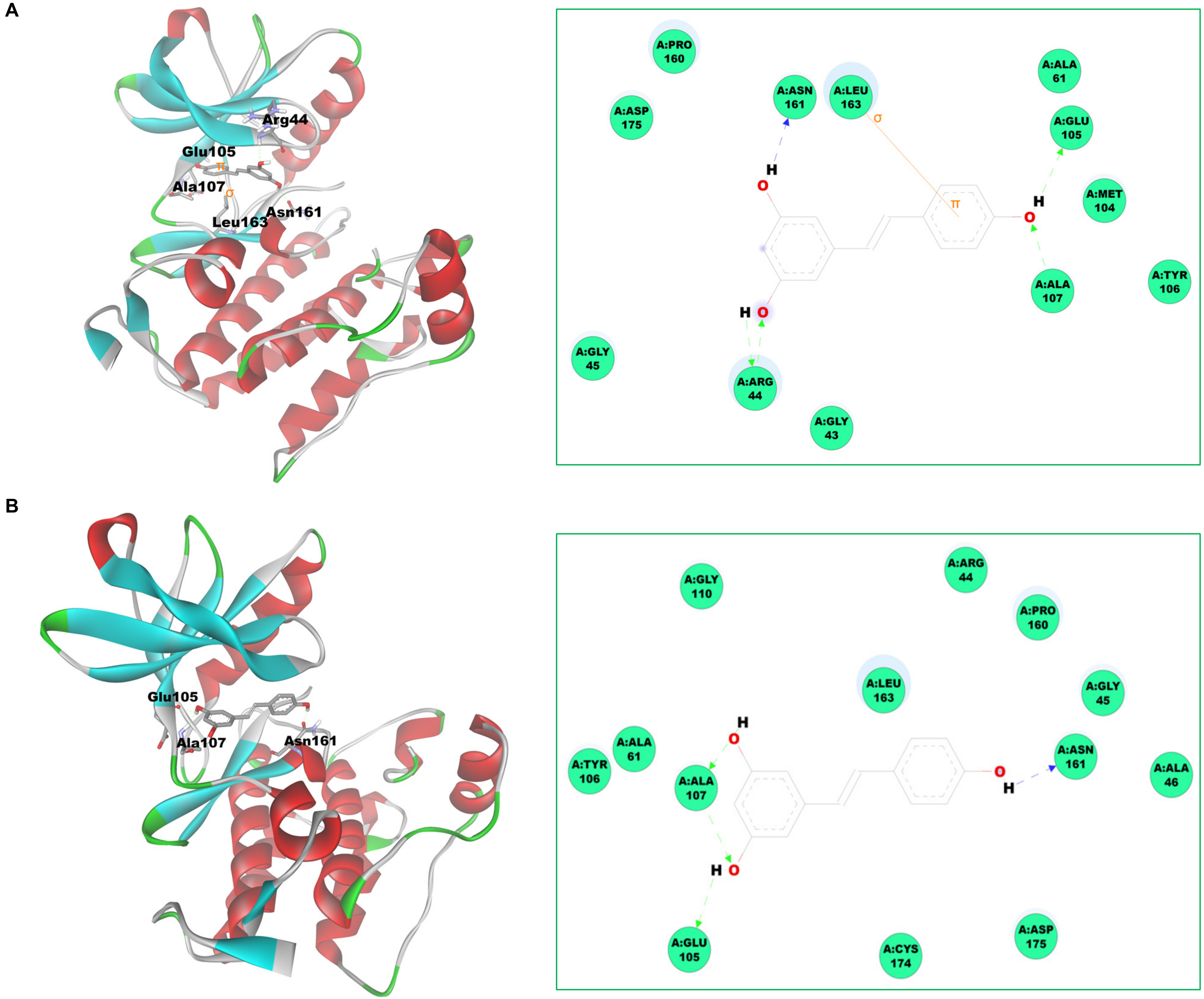
A
Arg44
Glu105
Ala107
Leu163
Asn161
A:PRO 160
A:ASP 175
A:ASN 161
A:LEU 163
A:ALA 61
A:GLU 105
A:MET 104
A:ALA 107
A:TYR 106
A:GLY 45
A:ARG 44
A:GLY 43
B
Glu105
Ala107
Asn161
A:GLY 110
A:ARG 44
A:PRO 160
A:LEU 163
A:GLY 45
A:ALA 61
A:TYR 106
A:ALA 107
A:ASN 161
A:ALA 46
A:GLU 105
A:CYS 174
A:ASP 175

Supplementary Figure S8 Key residues involved in interaction between TAK1 and resveratrol determined by molecular docking (A) 3D (left) and 2D (right) models showing key residues in one predicted binding confirmation between resveratrol and TAK1. **(B)** 3D (left) and 2D (right) models showing key residues in another predicted binding confirmation between resveratrol (in opposite orientation) and TAK1.

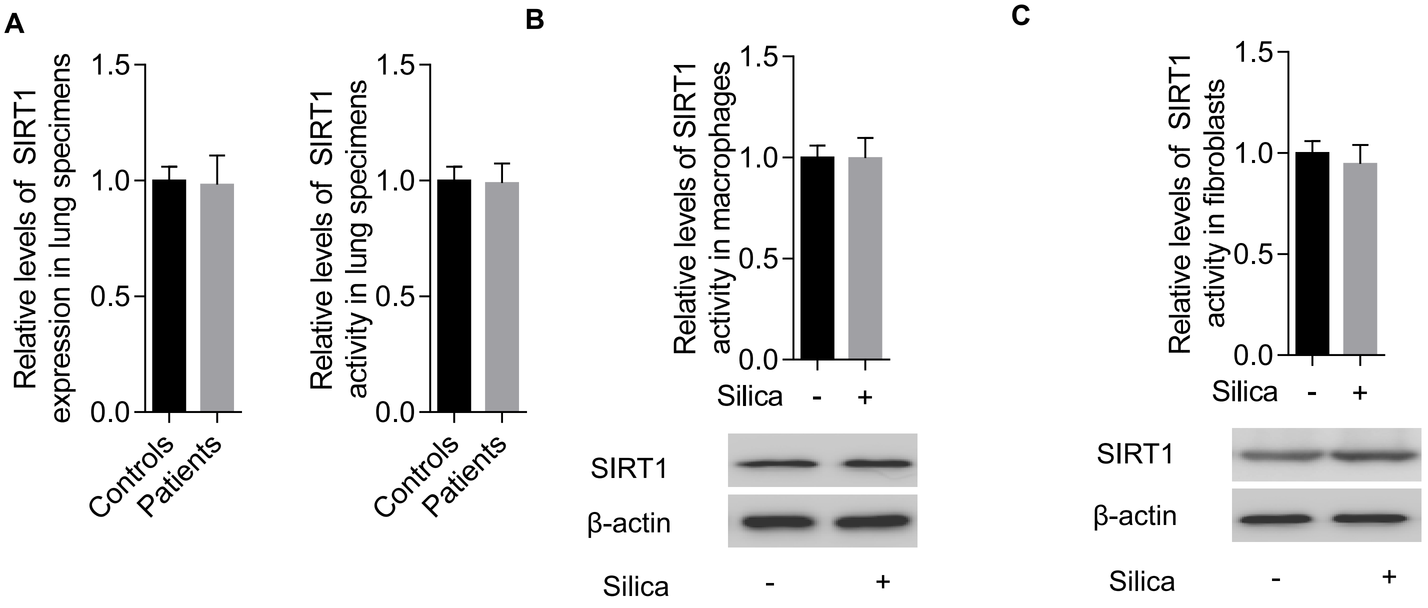
A
Relative levels of SIRT1 expression in lung specimens
Relative levels of SIRT1 activity in lung specimens
Controls
Patients
B
Relative levels of SIRT1 activity in macrophages
Silica - +
SIRT1
β-actin
Silica - +
C
Relative levels of SIRT1 activity in fibroblasts
Silica - +
SIRT1
β-actin
Silica - +

Supplementary Figure S9 Expression and activity of Sirtuin 1 (SIRT1) in pneumoconiosis (A) Relative levels of SIRT1 expression and activity in lung specimens from pneumoconiosis patients (n = 9) or control individuals (n = 6). Levels of SIRT1 expression determined by Enzyme-linked immunosorbent assay (ELISA) and SIRT1 activity determined by a commercial kit in lung specimens from pneumoconiosis patients were normalized to their mean value in lung specimens from control individuals, respectively. **(B)** Levels of SIRT1 expression determined by western blotting and SIRT1 activity determined by a commercial kit in primary alveolar macrophages isolated from rats with (n = 5) or without silica-exposure (n = 5). **(C)** Levels of SIRT1 expression determined by western blotting and SIRT1 activity determined by a commercial kit in primary lung fibroblasts isolated from rats with (n = 5) or without silica-exposure (n = 5). Level of SIRT1 activity in primary alveolar macrophages or fibroblasts isolated from rats with silica-exposure was normalized to that in primary alveolar macrophages or fibroblasts isolated from rats without silica-exposure. Data are presented as mean ± s.d. Student's *t* test was performed to determine the statistical differences between the two groups.

Supplementary Table S1 Aberrantly expressed genes in silica-exposed NR8383 cells

No.	Probe Name	Gene Symbol	Absolute Fold Change ([Silica treatment] vs [Control])	P-value	Regulation
1	A_44_P334736	Edn1	60.68303972	6.6798E-08	up
2	A_44_P371339	Il6	60.16219865	5.5793E-07	up
3	A_44_P557487	Cpb2	50.06123456	1.64355E-05	up
4	A_64_P007508	Csf3	49.95123641	2.89846E-05	up
5	A_64_P021723	TAK1	45.51287314	2.57853E-05	up
6	A_42_P687186	Sdc4	14.26187652	2.99942E-06	up
7	A_44_P132610	Slc20a1	12.16127651	4.17553E-07	up
8	A_64_P182654	Birc2	11.22487543	2.86193E-05	up
9	A_64_P149139	Rgs3	10.92127687	0.003793919	up
10	A_42_P515405	Ier3	9.512439871	0.001663364	up
11	A_44_P335446	Dusp2	9.434234578	1.02073E-05	up
12	A_64_P126236	Dbn1	9.161265789	0.001724112	up
13	A_64_P160673	Tor1aip2	9.091898794	7.97497E-07	up
14	A_64_P031456	Top1	8.562365789	2.73136E-06	up
15	A_64_P064020	Fgfr1	8.064676516	0.026801377	up
16	A_42_P597242	RGD1303142	7.932368651	6.77845E-09	up
17	A_44_P357470	Bmpr1a	7.914657891	0.000148037	up
18	A_64_P015607	Chka	7.873651908	3.3146E-07	up
19	A_44_P137802	Smpd1	7.860875761	2.18743E-05	up
20	A_42_P806899	Kdm6b	7.292109876	0.002907056	up
21	A_64_P031506	Tubb3	6.983788065	0.000205973	up
22	A_44_P527556	Taf7	6.801876891	0.000735284	up
23	A_44_P245775	RGD1305014	6.712312873	4.63359E-06	up
24	A_64_P014717	Mllt11	6.634218761	3.70859E-05	up
25	A_64_P057566	Kcnn4	6.470986124	0.004089239	up
26	A_64_P005193	Kb21	6.412876912	0.001489889	up
27	A_42_P768978	Nrp1	6.230976514	0.003907606	up
28	A_64_P101792	Efnb3	6.133276891	0.003333402	up
29	A_44_P1043157	Cxcl9	6.122198712	0.00235347	up
30	A_44_P696669	Ehd1	6.081254081	1.29105E-05	up
31	A_42_P648200	Pak1ip1	6.021298684	1.62095E-05	up
32	A_64_P035212	Polr1e	5.980898763	0.000431815	up
33	A_64_P163169	Begain	5.961876901	0.046328757	up
34	A_42_P727584	Mapk6	5.950876543	1.57208E-05	up
35	A_44_P224326	Rnf38	5.892098762	4.50227E-05	up
36	A_64_P024356	Sh2b2	5.664198764	0.000091118	up
37	A_64_P021800	Kpna4	5.632098751	2.65593E-05	up
38	A_44_P490206	Eif1b	5.621280762	4.4908E-06	up
39	A_64_P163728	Rai12	5.550876891	3.14817E-06	up
40	A_42_P536564	Etf1	5.501876808	8.28609E-09	up
41	A_44_P454646	Fam69a	5.491987678	4.31565E-05	up
42	A_44_P1041610	Atp6v0b	5.463239071	0.000351243	up
43	A_64_P165015	Shcbp1l	5.384098765	0.001385853	up
44	A_64_P063463	Sgpl1	5.362387609	2.39151E-05	up
45	A_64_P069646	Nacc2	5.281276871	0.000150739	up
46	A_43_P10498	Bag2	5.271238709	0.00025402	up
47	A_64_P075515	Celf1	5.264098717	0.000356279	up

48	A_64_P152091	Tmem194b	5.161409871	0.003354354	up
49	A_64_P077723	Nptn	5.150187687	0.000129056	up
50	A_44_P1048663	Ccnl1	5.124098722	4.32592E-05	up
51	A_64_P025583	RGD1311343	4.853039716	7.89563E-08	up
52	A_64_P143547	Fabp9	4.843998215	7.05483E-07	up
53	A_64_P148976	Rnase10	4.841031452	5.26522E-07	up
54	A_44_P556319	Fgf18	4.812011821	8.6575E-07	up
55	A_44_P192000	Sbsn	4.812335747	0.000476613	up
56	A_44_P298465	Gcg	4.802509053	1.99E-06	up
57	A_42_P473398	Cxcl1	4.800970928	6.92E-06	up
58	A_64_P072903	Astl	4.799264525	8.73E-05	up
59	A_44_P370052	Ldhc	4.747993412	1.06E-07	up
60	A_44_P478144	LOC290595	4.74119479	5.75E-07	up
61	A_64_P034244	Olr1	4.698946472	4.12E-07	up
62	A_43_P14872	Sdc1	4.692590385	1.30E-06	up
63	A_44_P314969	Slc6a3	4.68347793	2.54E-06	up
64	A_64_P011489	Gdf15	4.638006901	9.49E-06	up
65	A_64_P155311	Prss22	4.637451092	9.75E-05	up
66	A_64_P061895	Plscr2	4.632283874	0.000468106	up
67	A_43_P15447	Bglap	4.62976821	0.000102703	up
68	A_64_P081203	Dmkn	4.624265291	5.08E-06	up
69	A_64_P136820	Gas2l3	4.621279453	1.05E-05	up
70	A_64_P142320	Zfp458	4.620909602	4.30E-07	up
71	A_64_P025833	Oas1h	4.587594999	2.10E-05	up
72	A_64_P139802	Prph	4.578599791	5.55E-06	up
74	A_64_P131151	Opn4	4.564504109	2.92E-07	up
75	A_44_P456172	Mterfd3	4.560754457	5.89E-05	up
76	A_64_P073303	Plac1l	4.55376655	2.75E-06	up
79	A_64_P005471	Fam71f2	4.530810652	6.78E-07	up
80	A_64_P140437	Cxcl2	4.528945986	3.05E-05	up
81	A_42_P559831	Ces1c	4.509807853	7.37E-06	up
82	A_44_P318318	Mmp3	4.508283788	1.56E-06	up
83	A_64_P080682	LOC691952	4.492513586	1.61E-05	up
84	A_42_P529780	Spdya	4.48815562	2.03E-07	up
85	A_64_P012198	Agrp	4.485710352	3.06E-07	up
86	A_64_P079474	LOC691670	4.484512515	1.67E-05	up
88	A_44_P536089	Klre1	4.481827016	0.001092583	up
90	A_44_P1035040	Ccrl2	4.474679647	2.16E-07	up
91	A_64_P127986	Fam126b	4.471466291	5.21E-05	up
92	A_44_P483992	LOC678910	4.470124149	8.42E-05	up
93	A_44_P448030	RGD1307805	4.458755324	6.96E-07	up
94	A_64_P109899	Lgals4	4.449780586	8.80E-05	up
95	A_64_P108887	Cdk5r1	4.435589947	0.000618088	up
96	A_44_P392201	LOC682105	4.425890731	0.000139551	up
97	A_44_P540992	Ereg	4.399858824	3.42E-06	up
98	A_64_P028190	Irg1	4.370067812	4.67E-08	up
99	A_64_P123118	Fosl1	4.366533878	0.007693739	up
100	A_64_P046125	Tmem88	4.360958717	6.50E-06	up
101	A_44_P233080	Egr1	4.355929084	7.44E-07	up
102	A_64_P087543	Foxi1	4.33692652	0.006282403	up
103	A_44_P142837	Xirp1	4.333905412	6.99E-05	up

104	A_43_P15198	Rap1gap2	4.302001417	2.29E-05	up
105	A_64_P054302	Olr556	4.294146566	0.036175178	up
106	A_44_P469584	Cd14	4.289083591	1.38E-05	up
107	A_64_P008033	Fam83f	4.281966573	0.009857523	up
108	A_64_P100516	Aoc3	4.272848406	0.000433918	up
109	A_64_P089170	Cxadrl1	4.261899776	0.000665369	up
110	A_64_P055930	Tmbim1	4.259516465	2.00E-05	up
111	A_43_P11985	Ccl20	4.256743029	0.00018013	up
113	A_64_P157116	Wee2	4.255990312	0.000148664	up
114	A_64_P115060	Ccl5	4.247426846	1.55E-05	up
115	A_42_P638620	Lcn2	4.210451948	5.83E-06	up
116	A_64_P046552	Sdcbp2	4.20700053	3.90E-05	up
117	A_64_P015460	Ccl4	4.20041702	4.87E-06	up
118	A_43_P18469	Sytl3	4.194658903	9.70E-05	up
119	A_64_P023922	LOC680810	4.185192553	0.00040148	up
120	A_64_P135102	Tas2r140	4.164044924	1.07E-06	up
121	A_64_P066073	Rap1gap	4.161570853	0.00026908	up
122	A_42_P758222	Arg1	4.159088546	1.93E-05	up
123	A_64_P151795	LOC689618	4.137745752	2.83E-06	up
124	A_64_P077990	Cphx	4.124487888	0.0025625	up
126	A_42_P750340	RGD1563319	4.115763449	5.72E-05	up
127	A_64_P123169	Mrp63	4.114781622	8.67E-05	up
128	A_44_P462661	Il1rn	4.111062865	6.91E-07	up
129	A_64_P129895	Tnfsf15	4.110043985	0.000259579	up
130	A_44_P429006	Cers1	4.106353039	0.000328608	up
131	A_64_P150047	Rab20	4.093518835	1.23E-06	up
132	A_64_P248290	Liph	4.091120507	6.02E-05	up
133	A_42_P517554	Polg2	4.08254843	2.68E-07	up
134	A_43_P11616	Atf3	4.04754859	1.52E-07	up
135	A_64_P029486	Tnnc2	4.027859798	0.000142738	up
136	A_64_P007568	LOC683581	4.017193162	1.89E-05	up
137	A_64_P162306	Ftsj2	4.016131013	1.02E-05	up
139	A_44_P253393	Tm7sf4	4.014950312	1.31E-08	up
140	A_64_P065736	Slc7a2	4.014348868	1.54E-06	up
141	A_43_P11040	Tppp3	4.001324592	2.07E-06	up
142	A_64_P153159	Ebi3	3.997060288	5.59E-06	up
143	A_64_P113179	Stk32a	3.984957488	0.026949936	up
144	A_44_P1022002	Ccl7	3.979867866	2.72E-07	up
145	A_44_P457599	Foxj1	3.97892903	6.78E-08	up
146	A_44_P245845	RGD1563302	3.975224485	0.010719018	up
147	A_44_P269457	Gpr83	3.971048552	8.40E-07	up
148	A_64_P063518	Slc25a37	3.967423302	7.85E-07	up
149	A_64_P131021	Elovl3	3.956899711	0.00014373	up
150	A_64_P034541	RGD1561909	3.95473493	1.10257E-05	up
151	A_42_P693964	Cyp26b1	3.952957927	0.001258744	up
152	A_44_P899293	Snai1	3.951713897	5.90243E-06	up
153	A_64_P142492	Nos2	3.944941756	0.002450803	up
154	A_43_P23060	Hsfy2	3.941100666	0.028295842	up
155	A_64_P021141	Rgs1	3.934978628	2.75376E-06	up
156	A_64_P076832	RGD1311447	3.927654971	4.94198E-05	up
157	A_64_P023719	Nlrp6	3.926655417	8.49009E-05	up

158	A_44_P182881	Trim69	3.922654669	2.14252E-06	up
159	A_64_P150904	Rgc32	3.914682222	9.0851E-06	up
160	A_44_P791176	Cyth4	3.91004506	2.88469E-06	up
162	A_44_P765013	Rnf186	3.90940938	0.001225794	up
163	A_64_P134410	Nfia	3.909258086	0.004306577	up
165	A_64_P007943	Il7r	3.889543926	8.99641E-07	up
166	A_64_P108845	Spint2	3.885859306	3.63744E-06	up
167	A_64_P038634	RGD1310081	3.884848487	4.26965E-07	up
168	A_44_P501112	Mmp9	3.875897271	0.000010762	up
170	A_64_P067065	Cd207	3.87163979	2.26799E-06	up
171	A_44_P1046118	Tctex1d2	3.867700279	3.61509E-06	up
172	A_64_P034414	Tnf	3.865895124	0.000180076	up
174	A_44_P423651	Prss12	3.859349859	0.001996	up
175	A_43_P12786	Fabp4	3.857088726	4.88204E-09	up
176	A_64_P100783	Cnnm1	3.856202998	0.024120471	up
177	A_64_P072219	Rtp3	3.853841309	8.41859E-07	up
178	A_44_P411097	Apoa1	3.847716308	3.25302E-05	up
179	A_64_P083229	LOC688635	3.847712486	0.000257313	up
180	A_44_P382831	Epm2aip1	3.844043246	5.27783E-06	up
182	A_64_P103025	Il17f	3.841482283	8.04502E-05	up
183	A_44_P277264	Skil	3.833213089	3.11504E-05	up
184	A_44_P435596	Zfp36	3.829812246	5.10928E-05	up
185	A_64_P137784	Plvap	3.827914926	2.64099E-05	up
187	A_64_P069386	RGD1562871	3.824918568	0.001365641	up
189	A_44_P105448	Col4a5	3.822234431	0.018441418	up
190	A_64_P140144	LOC679989	3.82200412	0.023305509	up
191	A_64_P158198	RGD1561239	3.806993219	5.93296E-06	up
192	A_44_P408173	Kif2b	3.8031107	0.00040975	up
193	A_44_P294838	Il1a	3.801447412	2.38307E-06	up
194	A_44_P271511	Adam2	3.799067468	0.000236821	up
195	A_64_P109143	Gkn1	3.793639703	0.026604053	up
197	A_64_P122489	Hdc	3.789749	0.000146495	up
198	A_64_P054923	LOC680029	3.788811854	0.000129572	up
199	A_44_P409820	Faslg	3.788723878	8.01412E-05	up
200	A_64_P060303	Lsr	3.788545304	6.43442E-05	up
201	A_64_P058015	Gimap1	3.785889054	0.0379662	up
202	A_64_P025639	Rec8	3.775764192	0.00141192	up
205	A_44_P109523	Olr1627	3.775223612	0.000316822	up
206	A_44_P883087	Rgs13	3.76945252	5.40627E-08	up
207	A_64_P151468	Spint3	3.764386601	0.000508778	up
208	A_64_P054251	Pax3	3.764187084	0.000382004	up
209	A_64_P185041	LOC494527	3.763551639	1.36743E-07	up
210	A_44_P1034950	Adora2a	3.761476299	2.20823E-05	up
211	A_42_P695401	Ccl2	3.751417505	2.27647E-05	up
212	A_64_P081063	LOC363746	3.74017861	0.000119967	up
214	A_64_P097857	LOC10023321:	3.739989794	0.01358707	up
215	A_64_P053353	Jun	3.734308197	4.90317E-06	up
217	A_44_P511275	LOC685904	3.732788583	0.000685145	up
218	A_64_P082341	Slc5a3	3.730978983	0.000448684	up
219	A_44_P808807	Lrrc23	3.726726211	0.010260291	up
220	A_64_P078894	Il23a	3.723897422	0.000114621	up

221	A_44_P1047467	Igf2bp2	3.722445345	4.19E-05	up
222	A_64_P045122	Mesp1	3.721487096	1.77E-05	up
223	A_44_P762292	Serpinf2	3.716696797	5.04E-05	up
224	A_64_P061662	Entpd2	3.714440973	0.00022721	up
225	A_64_P148816	RGD1561777	3.708285103	0.005571893	up
226	A_64_P043411	Lrrc26	3.701704332	0.001800527	up
227	A_42_P765736	Agpat9	3.695492639	0.00018355	up
228	A_64_P081040	Gzmn	3.690980204	0.000559433	up
229	A_43_P16529	Gadd45b	3.678845273	0.00075509	up
230	A_44_P520929	C1qtnf1	3.672127539	3.03E-05	up
231	A_64_P267691	Trpm3	3.668087024	0.048238612	up
232	A_64_P025808	Oas1e	3.663291147	3.03E-05	up
233	A_64_P029805	Junb	3.662457454	1.03E-05	up
234	A_44_P667270	RGD1559875	3.662322063	2.80E-05	up
235	A_64_P161635	RGD1310507	3.660291803	4.58E-05	up
236	A_64_P000824	Tecpr1	3.655439396	0.001402386	up
237	A_64_P092668	Zfp275	3.649506355	0.004994644	up
238	A_64_P003784	Casp14	3.642734883	9.32E-05	up
239	A_64_P014080	Htr3b	3.642721416	0.002754551	up
240	A_64_P139497	Olr1262	3.641033463	0.032315705	up
241	A_64_P295167	Trpc3	3.637970893	0.002776655	up
242	A_42_P683837	Serpinb6b	3.63433985	0.000748796	up
243	A_44_P436310	Ctnna3	3.632483723	0.017976701	up
244	A_44_P175495	Cxcl11	3.617872335	9.53E-05	up
245	A_44_P513747	Eea1	3.609439111	1.06596E-05	up
246	A_64_P062899	Bcar1	3.604413931	0.005421746	up
247	A_42_P623913	Sox18	3.602761875	0.030224555	up
248	A_43_P10581	Mafk	3.589442284	0.00171437	up
249	A_64_P013654	Pde6b	3.587113763	0.000164141	up
250	A_64_P136362	RGD1306000	3.575705866	0.020389539	up
251	A_42_P824489	Tgm2	3.573116946	1.76E-05	up
252	A_42_P506402	Slc25a25	3.570676253	2.79034E-06	up
253	A_64_P056707	Scg2	3.566697675	3.24979E-05	up
254	A_43_P16166	Cd69	3.565812638	7.79504E-07	up
255	A_64_P021631	Nxnl2	3.562412552	0.000482789	up
256	A_42_P610788	Ampd3	3.561237535	8.74644E-06	up
257	A_64_P164255	Hivep3	3.557545808	0.001072673	up
258	A_44_P371635	Tnfaip6	3.557477996	7.89E-06	up
259	A_64_P079930	Tcf15	3.556374286	0.000971982	up
260	A_64_P010653	Cyb5r1	3.541604553	0.000760108	up
261	A_64_P036875	Lalba	3.534639709	0.013709839	up
262	A_64_P058336	Ucn	3.525943442	0.000140141	up
263	A_64_P052017	Cxcl3	3.520151693	1.14968E-06	up
264	A_64_P125472	LOC301893	3.51662297	0.000122451	up
265	A_44_P332780	Ptprj	3.516327553	0.000156646	up
266	A_64_P044776	Itih4	3.515910225	0.001523485	up
267	A_44_P886690	Lpar2	3.514313269	0.000318459	up
268	A_44_P154138	Rxfp2	3.513580615	0.008103979	up
269	A_64_P080371	Vom2r73	3.51172255	3.21E-06	up
270	A_44_P639832	Dhh	3.509599972	0.000519705	up
271	A_42_P473594	Egr2	3.507887142	3.38E-07	up

272	A_44_P518764	LOC363060	3.506119414	1.45E-06	up
273	A_42_P543654	Fam176a	3.503890443	0.000699283	up
274	A_64_P153951	Ror2	3.496373559	0.034777175	up
275	A_42_P625157	Zfp503	3.493777822	3.96E-05	up
276	A_44_P116261	Gapdhs	3.482590877	0.037732045	up
277	A_44_P536613	Ch25h	3.474022505	0.000310579	up
278	A_43_P12619	Nr4a3	3.472078582	0.016913009	up
279	A_43_P21037	Snta1	3.471445688	1.01E-05	up
280	A_43_P15253	Icam1	3.462148324	1.02E-05	up
281	A_64_P146692	Slc22a1	3.445127397	0.000220123	up
282	A_64_P145973	RGD1307722	3.443432189	0.021764524	up
283	A_64_P118789	Olr1468	3.439450279	0.049133663	up
284	A_43_P17221	Myoz2	3.433593698	0.00072043	up
285	A_64_P021795	Kpna5	3.428852473	0.040160789	up
286	A_44_P454065	Jag1	3.424021672	1.00107E-05	up
287	A_42_P739860	Dusp5	3.423143882	7.53577E-06	up
288	A_42_P574345	LOC100360737	3.418205974	0.000210956	up
289	A_64_P097842	Marcksl1	3.416183173	3.58657E-08	up
290	A_64_P034744	Acrbp	3.410501743	5.55873E-07	up
291	A_64_P023306	Dot1l	3.409288056	7.89354E-05	up
292	A_64_P019345	Gk5	3.407309344	0.016407045	up
293	A_44_P219796	Nckap1	3.404637817	3.83419E-07	up
294	A_64_P041712	Zic2	3.396901614	0.042489713	up
295	A_64_P046140	Mrgprb13	3.393343944	0.003148274	up
296	A_44_P211069	RGD1306119	3.392311535	2.15972E-06	up
297	A_64_P027707	LOC685046	3.388774253	0.008144563	up
298	A_44_P175807	Rlim	3.387038377	9.32652E-06	up
299	A_44_P620927	Clec4e	3.383857464	4.73885E-08	up
300	A_44_P821368	RGD1560958	3.381022694	0.001911572	up
301	A_64_P113301	LOC681198	3.378405888	1.21651E-05	up
302	A_44_P354078	Hist1h1a	3.369545951	0.000616996	up
303	A_64_P011116	LOC681936	3.369118097	7.63985E-06	up
304	A_64_P021775	LOC286992	3.368846436	0.033759561	up
305	A_43_P22825	Cabyr	3.367101007	0.001224378	up
306	A_44_P102369	Socs1	3.361997535	0.041596445	up
307	A_64_P104481	Spag7	3.359508934	0.000753383	up
308	A_44_P776423	Opalin	3.357611555	0.005777135	up
309	A_64_P092026	Bhlha15	3.349681132	0.000354705	up
310	A_64_P055017	Olr500	3.346234768	0.000110884	up
311	A_64_P059775	Stmn3	3.344098479	0.000537192	up
312	A_44_P349854	Gna13	3.328783248	0.002160614	up
313	A_64_P049131	LOC680716	3.323408575	4.3729E-06	up
314	A_44_P590748	Ifih1	3.305498057	2.80569E-05	up
315	A_64_P051533	LOC690463	3.304833677	0.002245037	up
316	A_43_P14911	Il1b	3.304166758	3.46203E-05	up
317	A_64_P105362	RGD1308818	3.30387415	0.00127958	up
318	A_64_P062890	Oaz3	3.291934582	0.000276093	up
319	A_44_P1038028	Tnfrsf12a	3.287228324	3.87098E-05	up
320	A_44_P330828	MGC105649	3.277173377	6.84431E-07	up
321	A_64_P139408	Evc2	3.276834175	0.002010639	up
322	A_43_P11527	Adm	3.263714816	0.000551368	up

323	A_44_P1004840	Tnfsf9	3.254108452	6.19919E-05	up
324	A_44_P470430	Fap	3.243652646	2.82895E-05	up
325	A_64_P067005	Rnf19b	3.240108669	0.04358081	up
326	A_44_P487530	Tmem56	3.23986021	0.009867796	up
327	A_64_P170328	RGD1563231	3.238836183	0.001007188	up
328	A_64_P132511	Mcc	3.234578367	0.002911299	up
329	A_64_P031123	Slc1a2	3.23399123	2.14567E-05	up
330	A_64_P047027	Rhpn2	3.233316868	0.001259395	up
331	A_43_P19852	Fam71a	3.231110224	0.000117885	up
332	A_44_P325629	Olr1425	3.225174156	0.039641289	up
333	A_64_P126772	Flywch1	3.224275826	0.002197157	up
334	A_64_P043644	Slamf6	3.222808949	1.58572E-05	up
335	A_44_P312089	Neurl3	3.221074435	0.000324367	up
336	A_44_P1004757	Serpinb10	3.220762917	0.00321962	up
337	A_64_P042219	Syt7	3.212438348	0.000158284	up
338	A_64_P111594	Dnah10	3.206738327	5.57112E-05	up
339	A_64_P129293	RGD1306565	3.205449026	0.005001139	up
340	A_64_P125973	Tnfrsf1b	3.204412035	0.000021808	up
341	A_64_P118367	Lrrd1	3.20259648	0.011725132	up
342	A_64_P024540	Guca2a	3.200111855	0.000259189	up
343	A_64_P151126	Cep152	3.197764954	5.83976E-06	up
344	A_44_P144257	Fzd5	3.195930865	8.60906E-05	up
345	A_44_P1037806	Hpx	3.195599629	0.001326436	up
346	A_64_P014504	Il27	3.192398608	0.000260909	up
347	A_44_P635178	Fam129b	3.188162872	3.69544E-05	up
348	A_44_P368065	Adprhl1	3.181341105	0.009214306	up
349	A_64_P024798	Ubox5	3.177857867	0.00042803	up
350	A_64_P130967	LOC683410	3.172570008	0.000637777	up
351	A_44_P1008751	Fgd6	3.165076419	1.14516E-05	up
352	A_64_P004705	Akap2	3.164355963	2.60815E-05	up
353	A_64_P120808	Krt80	3.162742132	0.004707422	up
354	A_64_P087184	Vwa2-ps1	3.162249792	0.033201503	up
355	A_42_P764365	Mpp4	3.157108188	0.000263419	up
356	A_64_P003972	Slc22a4	3.155336057	1.33269E-05	up
357	A_64_P040300	LOC10036614[illegible]	3.152487391	0.006960085	up
358	A_64_P011005	Eid3	3.150832729	3.95635E-06	up
359	A_64_P162331	Zfp46	3.150454921	0.000299315	up
360	A_42_P839964	Plin2	3.149108212	6.4411E-07	up
361	A_42_P488904	Slc25a44	3.147292868	2.46109E-06	up
362	A_42_P574859	Hamp	3.145674075	1.30604E-06	up
363	A_44_P552514	Ccr7	3.144018413	3.05232E-05	up
364	A_64_P033591	LOC10036512[illegible]	3.143659435	0.005288612	up
365	A_44_P515305	Olr120	3.140578143	0.038071195	up
366	A_43_P11472	Hmox1	3.127567165	8.63879E-06	up
367	A_42_P610317	Zfp142	3.127327914	5.75245E-06	up
368	A_44_P504221	Ccnt1	3.118909447	0.046008695	up
369	A_43_P19763	Tlr2	3.118547644	1.66994E-05	up
370	A_42_P485932	Sulf2	3.100736863	0.000033691	up
371	A_44_P404861	Mmp10	3.096942148	9.69909E-05	up
372	A_64_P165666	Siglec5	3.082892677	1.08717E-06	up
373	A_44_P1023538	C3	3.078644532	0.003792053	up

374	A_64_P064983	RGD1560455	3.072521984	0.000213265	up
375	A_44_P771864	Cdc14a	3.070317106	0.000203421	up
376	A_42_P541884	Irak2	3.06659455	3.62677E-06	up
377	A_64_P012939	Dapk2	3.065421655	0.003784632	up
378	A_42_P576446	Slc7a11	3.062266341	4.35735E-06	up
379	A_64_P072808	Mtnr1a	3.06210248	0.021931545	up
380	A_64_P127823	Ggct	3.06166153	0.004825268	up
381	A_64_P056743	LOC10036605	3.061641723	0.005035859	up
382	A_43_P11660	Pspn	3.059932793	0.004415331	up
383	A_64_P035817	Whsc1l1	3.059241501	9.52111E-05	up
384	A_64_P142111	Gxylt1	3.057553409	0.000457275	up
385	A_42_P817417	PVR	3.056512216	4.8689E-06	up
386	A_64_P078393	Aff4	3.049772512	7.71264E-06	up
387	A_44_P342426	Irf4	3.043840791	0.016865818	up
388	A_64_P079149	LOC685371	3.043400782	0.002300283	up
389	A_64_P125715	Serinc4	3.041440611	0.000219686	up
390	A_64_P049085	LOC10036421	3.038563272	0.007838295	up
391	A_64_P130407	Megf11	3.038364807	0.043912712	up
392	A_64_P134265	Rmrp	3.031736846	0.001007009	up
393	A_44_P321532	Apon	3.024134907	0.000132205	up
394	A_64_P143862	LOC678958	3.023544893	0.037652057	up
395	A_64_P054949	Vom2r72	3.019986708	0.002211933	up
396	A_44_P561879	Mthfd2l	3.019225124	0.000121219	up
397	A_64_P026374	Flt3	3.016882901	0.026506798	up
398	A_64_P046358	LOC688507	3.012113221	0.000806592	up
399	A_42_P730320	RGD1307937	3.008366979	3.92619E-05	up
400	A_42_P621872	Gcgr	3.003406531	2.39032E-06	up
401	A_43_P19284	Bcar3	3.001810005	0.010909233	up
402	A_44_P1039128	Cxcl10	2.992793629	0.000368142	up
403	A_64_P059151	Lhx9	2.988633524	0.005465241	up
404	A_44_P1014659	Plek2	2.983946701	0.028353418	up
405	A_64_P095810	Hormad2	2.978544541	0.048680418	up
406	A_64_P036390	Col4a3bp	2.975756407	8.52337E-08	up
407	A_64_P038478	Gcnt2	2.97473269	0.000486245	up
408	A_44_P186860	Nfkbib	2.970103213	1.15702E-05	up
409	A_44_P236820	LOC685608	2.96885987	0.0425452	up
410	A_44_P151482	Il22ra2	2.96792767	0.000592217	up
411	A_44_P245616	Nid1	2.963500567	0.000695122	up
412	A_64_P131915	LOC685685	2.96338273	0.045298795	up
413	A_44_P212964	Plac8	2.962373878	0.00033657	up
414	A_64_P040563	RGD1308305	2.961784486	0.011610894	up
415	A_64_P042183	Pim1	2.952983646	1.11479E-05	up
416	A_42_P596050	Cdhr1	2.950072343	3.69643E-05	up
417	A_64_P022447	Slc4a11	2.948703303	0.001012756	up
418	A_42_P682216	Tmem206	2.945294439	4.20425E-05	up
419	A_64_P056618	Mitf	2.944453724	0.000122609	up
420	A_64_P038887	Cdca7l	2.930461737	4.54162E-05	up
421	A_64_P006618	Fam71b	2.925887705	0.00019594	up
422	A_44_P372261	Slpi	2.922207585	7.26475E-05	up
423	A_64_P078108	Ets2	2.916998141	0.001107504	up
424	A_64_P021149	LOC686141	2.914665252	1.08148E-06	up

425	A_64_P024052	Dusp16	2.907110883	2.24453E-05	up
426	A_64_P016190	Rictor	2.906747524	1.02005E-05	up
427	A_64_P157229	Zfp334	2.905037996	0.009239691	up
428	A_42_P619110	Eaf1	2.898056992	1.14314E-07	up
429	A_64_P016013	Hepacam2	2.898012531	0.035995684	up
430	A_42_P535644	Adamts1	2.890723793	6.45653E-06	up
431	A_42_P457692	Zfand2a	2.889225216	2.81385E-05	up
432	A_44_P121446	Tmed5	2.886718508	1.44401E-07	up
433	A_42_P607012	RGD1305572	2.881956714	0.000083651	up
434	A_64_P116899	Cd247	2.876534885	0.013857557	up
435	A_44_P388783	Clec4d	2.873938786	0.000127262	up
436	A_44_P400250	Olr531	2.870727053	0.012494814	up
437	A_44_P337177	Olr816	2.850567703	0.011807153	up
438	A_64_P031781	LOC680578	2.845130183	0.008447716	up
439	A_64_P018781	Calcb	2.844439705	0.035307993	up
440	A_64_P074544	Ankrd61	2.843141036	1.64522E-06	up
441	A_64_P113950	Zc3hav1l	2.840375104	0.033844341	up
442	A_44_P281510	Tlr7	2.838017529	0.000161576	up
443	A_44_P807058	Emp1	2.835827478	9.30003E-05	up
444	A_64_P048606	Rabgef1	2.831570925	7.33677E-05	up
445	A_44_P279083	Abcb1a	2.828001071	0.000398383	up
446	A_43_P15536	Ifnb1	2.825538147	0.00012736	up
447	A_64_P061786	Mcf2l	2.823830839	0.003696314	up
448	A_44_P127597	Dnajb9	2.823356422	3.21578E-05	up
449	A_44_P206985	Gnrh1	2.823034644	6.12364E-07	up
450	A_64_P145917	Nus1	2.819866442	0.000261718	up
451	A_43_P10008	Aig1	2.817994248	0.006020091	up
452	A_44_P832655	LOC687118	2.817977645	0.000111474	up
453	A_44_P110788	Itgad	2.817010159	0.000244526	up
454	A_42_P612977	Cyb561	2.815199182	0.00254533	up
455	A_42_P794120	Stra6	2.81415716	0.00056731	up
456	A_64_P057643	Wdr25l	2.81176658	0.026695841	up
457	A_64_P110574	LOC365723	2.81022829	8.32229E-05	up
458	A_64_P019581	Olr1531	2.809133134	0.035863989	up
459	A_64_P077447	Tctn1	2.806709144	0.005952444	up
460	A_42_P684538	Slc2a1	2.794761426	9.55173E-06	up
461	A_64_P098053	Tm4sf19	2.793540882	0.000132137	up
462	A_64_P018510	Dcp1a	2.792396936	0.011608314	up
463	A_64_P149829	Fam154a	2.790840677	0.001812531	up
464	A_44_P323053	Usp53	2.789443506	0.000462262	up
465	A_64_P002156	Rgs2	2.788267867	5.93288E-06	up
466	A_64_P048637	Cnfn	2.787009847	0.00448148	up
467	A_43_P12384	Birc3	2.785390047	0.000975683	up
468	A_42_P457895	Supv3l1	2.7829497	5.66828E-06	up
469	A_64_P050199	Sema3c	2.782461258	0.002116789	up
470	A_64_P069216	Gramd1c	2.780987641	0.002678696	up
471	A_64_P126906	RGD1308644	2.772013596	0.000872232	up
472	A_44_P366157	RGD1311993	2.768956904	0.000210129	up
473	A_64_P108674	RGD1307554	2.768371006	6.09563E-06	up
474	A_64_P232281	Vom2r5	2.767806079	3.90717E-05	up
475	A_44_P492025	Oasl	2.766135572	0.000859503	up

476	A_44_P222124	Tssk3	2.765960077	0.001625338	up
477	A_64_P105221	Unc5cl	2.763630681	0.001269555	up
478	A_64_P162950	Ccr1l1	2.760126075	0.000535839	up
479	A_44_P1027971	Gpr84	2.759540833	0.00014749	up
480	A_42_P570241	Nampt	2.755722914	6.74348E-07	up
481	A_44_P1001317	Obfc2a	2.749857261	0.001037819	up
482	A_64_P073979	Mcoln3	2.746718382	1.01621E-06	up
483	A_42_P671348	Hhatl	2.745063647	0.000604092	up
484	A_64_P078185	Hbz	2.737811273	0.025960799	up
485	A_64_P070208	Gdap1l1	2.736882503	0.006637112	up
486	A_44_P318553	Met	2.736167847	0.000254298	up
487	A_42_P524525	Otp	2.734884046	0.00160104	up
488	A_43_P16449	Pelo	2.733238083	0.000010416	up
489	A_64_P026389	Olr837	2.732038852	0.044259865	up
490	A_42_P685145	Cmpk2	2.729265225	1.95132E-05	up
491	A_64_P086969	Slc16a1	2.725195381	0.000407491	up
492	A_64_P082067	Ifit3	2.718272645	0.0006047	up
493	A_64_P041802	Klhl21	2.716600588	0.000128737	up
494	A_64_P010887	Sat1	2.714618884	1.48703E-06	up
495	A_64_P120479	Itgax	2.710020942	0.000056746	up
496	A_64_P083149	Kctd4	2.70821104	8.1153E-06	up
497	A_64_P001612	Trim35	2.70791227	6.11373E-05	up
498	A_64_P011929	Il34	2.706682751	0.023750741	up
499	A_64_P072451	LOC298109	2.704913452	0.001021495	up
500	A_64_P060695	Hic1	2.704807647	0.022122911	up
501	A_64_P085695	Rpl36a	2.702611239	0.045412789	up
502	A_64_P097078	Pdp2	2.701739355	1.32479E-05	up
503	A_64_P093234	Col12a1	2.697194698	0.000140902	up
504	A_43_P15474	Spef2	2.689981032	6.52096E-05	up
505	A_64_P153502	Armc9	2.685897346	1.87022E-06	up
506	A_64_P022248	Ccdc62	2.685704665	6.28871E-05	up
507	A_44_P348812	Hist1h1t	2.682452207	0.006802504	up
508	A_64_P073400	Olr922	2.681611362	0.000533208	up
509	A_44_P351211	Phlda1	2.679823529	0.033704961	up
510	A_43_P12241	Prl7a3	2.67937343	0.000786279	up
511	A_64_P063229	Icam5	2.678325493	5.97047E-07	up
512	A_64_P045646	Dppa3l1	2.677965423	0.014593751	up
513	A_64_P057535	RGD1304770	2.677935538	0.043928472	up
514	A_64_P102433	Slc11a2	2.675995271	1.17643E-05	up
515	A_64_P054538	Clic4	2.666703082	1.31468E-05	up
516	A_44_P443500	RGD1309808	2.664967366	0.001931552	up
517	A_64_P147174	LOC10036591[illegible]	2.662950294	4.89732E-07	up
518	A_44_P194803	Baalc	2.662267122	0.041204633	up
519	A_64_P074665	Olr1366	2.662042492	0.014975261	up
520	A_64_P161546	Aadacl3	2.659212066	0.00048357	up
521	A_64_P269447	Nr3c1	2.653522288	0.000042377	up
522	A_64_P038625	LOC686860	2.651746137	1.00069E-05	up
523	A_64_P072387	Zfp295	2.647509939	3.14606E-05	up
524	A_64_P010648	Ccl12	2.643163495	1.74322E-06	up
525	A_64_P125676	Sptbn4	2.641934868	0.003729938	up
526	A_42_P667148	Plscr1	2.637133609	5.24816E-06	up

527	A_64_P014687	Shbg	2.636531804	0.008320502	up
528	A_64_P097527	Pdhx	2.636319213	3.7681E-06	up
529	A_64_P130523	Niacr1	2.634851036	7.51375E-06	up
530	A_44_P121280	Kcne1	2.627035325	0.000710995	up
531	A_64_P116924	LOC498705	2.622562097	0.000316744	up
532	A_42_P542985	Cbln2	2.616862279	0.001236443	up
533	A_64_P116069	Stard8	2.610317265	0.044889913	up
534	A_44_P837227	LOC687994	2.604097863	2.08761E-05	up
535	A_44_P273355	Cxcr1	2.602355215	5.23736E-06	up
536	A_64_P138216	Gch1	2.601863181	8.42552E-06	up
537	A_64_P054074	Tgm3	2.599043175	0.010251111	up
538	A_64_P136046	Optn	2.598212927	5.44359E-06	up
539	A_64_P071139	Map3k2	2.597007175	2.69111E-05	up
540	A_44_P222936	Slc36a3	2.594985301	0.04444844	up
541	A_64_P114258	Alg10	2.594317647	0.000309021	up
542	A_42_P688320	Gbp5	2.590497157	0.000600949	up
543	A_44_P175530	Plek	2.590408575	8.46923E-05	up
544	A_44_P791030	Cecr6	2.590320296	0.04022686	up
545	A_64_P070053	Cd200	2.586889087	5.11525E-05	up
546	A_64_P114700	Slx1b	2.585950214	0.019433731	up
547	A_44_P321075	Rasal2	2.584500235	0.000428225	up
548	A_64_P102405	RGD1566149	2.584273926	9.68073E-06	up
549	A_64_P002422	LOC683713	2.580451771	1.13674E-06	up
550	A_42_P694928	Arl5c	2.57983566	0.010906518	up
551	A_44_P867688	Ttc23l	2.576972247	0.026380649	up
552	A_44_P437966	Apcs	2.575568481	0.004894746	up
553	A_43_P11044	G0s2	2.573838146	8.74331E-06	up
554	A_64_P105933	Defb52	2.573267789	0.000585655	up
555	A_64_P056693	Pom121l2	2.572301647	0.033781224	up
556	A_64_P049331	LOC683156	2.570848628	0.000371527	up
557	A_42_P775217	Ly6g6c	2.570401747	2.98608E-05	up
558	A_64_P125521	Hps5	2.568460701	0.000004311	up
559	A_64_P139173	Cd40	2.566766395	6.85075E-05	up
560	A_64_P039737	Scx	2.564501769	0.001937213	up
561	A_43_P12743	Atp7a	2.562459081	0.000157672	up
562	A_64_P136679	Rab7b	2.561959495	0.000325262	up
563	A_44_P837093	RGD1308023	2.561572101	0.000638424	up
564	A_64_P061677	Camp	2.560661407	6.50415E-05	up
565	A_64_P280765	Cd44	2.556314902	0.00082698	up
566	A_44_P1009709	Arl9	2.554286524	0.00138155	up
567	A_64_P014291	Dkkl1	2.549264379	0.034183662	up
568	A_64_P017916	LOC681371	2.548825019	0.000107155	up
569	A_44_P374638	Hsd17b2	2.547539649	0.000392865	up
570	A_64_P051822	Ly6g5c	2.54532786	0.019657771	up
571	A_64_P156448	LOC685157	2.543747609	0.000740744	up
572	A_64_P094561	LOC498201	2.540686436	5.79513E-05	up
573	A_44_P435422	Mef2a	2.536959403	0.00022338	up
574	A_64_P056712	Gpr137b	2.534539181	5.36857E-06	up
575	A_64_P116418	Slc25a33	2.533974255	2.80303E-05	up
576	A_64_P087169	Wtip	2.528458851	0.000762772	up
577	A_64_P115706	C1qtnf6	2.527564427	0.03746945	up

578	A_64_P019555	Sele	2.527277937	0.044884594	up
579	A_43_P13508	Arl5b	2.524930306	0.000163236	up
580	A_44_P226315	Slc16a10	2.51979203	0.020978144	up
581	A_64_P003789	LOC498480	2.518877567	0.014060658	up
582	A_44_P1013314	Isg15	2.518527644	0.000118725	up
583	A_44_P281540	Mcu	2.518312466	0.004302341	up
584	A_64_P160313	Nolc1	2.516585941	3.33973E-05	up
585	A_44_P822989	Rab11fip1	2.515066884	0.001453847	up
586	A_64_P138086	Pcdh20	2.514261429	0.000246945	up
587	A_44_P281733	Nlrp3	2.50973841	1.71086E-05	up
588	A_64_P110234	Serpina3k	2.505500224	0.01654111	up
589	A_64_P007633	C4bpb	2.503315507	7.82244E-05	up
590	A_64_P003589	Sts	2.50304258	3.72009E-05	up
591	A_64_P296046	Rhoc	2.502000828	0.000050528	up
592	A_44_P200018	Celsr3	2.50162695	0.040046669	up
593	A_44_P187195	C1galt1	2.496773875	8.25821E-05	up
594	A_64_P130475	RGD1310495	2.495783977	0.000310916	up
595	A_64_P017761	Morn1	2.49461717	0.022995483	up
596	A_64_P067831	Etv3l	2.49228243	6.75058E-05	up
597	A_64_P147334	Fkbp1b	2.491316532	0.032308903	up
598	A_44_P360772	Tagln	2.490694195	0.000642602	up
599	A_64_P378583	Vom2r71	2.482174599	0.000150577	up
600	A_42_P803673	LOC360228	2.481069588	3.81543E-05	up
601	A_42_P682589	Nr4a1	2.47785483	0.000172193	up
602	A_44_P161823	RGD1309049	2.47177513	0.002174335	up
603	A_44_P1026139	RGD1564927	2.471418846	0.001099207	up
604	A_42_P754654	RGD1562378	2.470006777	1.66735E-05	up
605	A_44_P130856	LOC10036080	2.469614742	0.03156543	up
606	A_64_P034952	Vom2r50	2.466414313	0.001035606	up
607	A_64_P044573	Gucy1a3	2.463756482	0.000828675	up
608	A_64_P088392	Hbb-b1	2.460015377	0.043684268	up
609	A_64_P215165	Spag9	2.451166299	0.000495139	up
610	A_44_P560961	Ell	2.448662035	4.80351E-05	up
611	A_64_P109610	Cflar	2.448211901	0.001359541	up
612	A_64_P131543	Olr140	2.445086888	0.017011254	up
613	A_64_P020938	Ppp1r15a	2.441127191	8.20745E-05	up
614	A_44_P104985	Serpinb2	2.43984613	0.007500102	up
615	A_64_P003997	Slco1a4	2.439113117	0.014127801	up
616	A_44_P284129	Csrnp1	2.433752566	0.011123426	up
617	A_44_P744940	Iqcf1	2.433484019	0.023783996	up
618	A_64_P155501	Fam155b	2.433400019	0.000327121	up
619	A_44_P792481	Mcts2	2.432720147	0.001808209	up
620	A_44_P494130	Nfkb1	2.432430076	1.48706E-05	up
621	A_64_P088587	Stk33	2.430597769	0.00050502	up
622	A_44_P289378	Cpt1b	2.430594119	2.51559E-05	up
623	A_64_P037245	Shisa2	2.429801288	0.018856645	up
624	A_64_P002621	Ptpn12	2.429572134	0.009355575	up
625	A_64_P103493	RGD1305704	2.429045195	0.000361762	up
626	A_64_P041040	Grip2	2.42866864	0.044653231	up
627	A_64_P158940	Wsb1	2.426493508	4.66312E-05	up
628	A_64_P035062	Rims3	2.423986785	0.002623198	up

629	A_64_P079260	Ttc36	2.423884576	0.000724816	up
630	A_44_P168159	Cd55	2.423769099	1.94371E-05	up
631	A_44_P725710	Zbtb2	2.423111009	0.004018767	up
632	A_44_P349089	Ifna1	2.421429501	0.010651304	up
633	A_64_P074940	Fgd4	2.416112559	0.001903693	up
634	A_64_P149230	Ccdc154	2.415393373	1.71405E-05	up
635	A_44_P811606	Ripk2	2.414931611	4.57058E-05	up
636	A_64_P113316	Zfyve1	2.413320737	5.09866E-05	up
637	A_44_P215157	Lin7b	2.41067389	0.029655113	up
638	A_64_P141050	RGD1563120	2.410539939	0.006004391	up
639	A_64_P140636	LOC688390	2.409712226	0.01224845	up
640	A_43_P12175	Xiap	2.4088371	2.64146E-06	up
641	A_64_P099963	Steap2	2.408290343	0.046652596	up
642	A_43_P21719	Mpzl2	2.407918117	0.000449832	up
643	A_64_P062766	Atp6v0e2	2.40629302	0.000681025	up
644	A_64_P232257	Vom2r65	2.40163349	0.000219793	up
645	A_64_P316652	Cacna1a	2.398654442	0.000108314	up
646	A_64_P121981	Htra4	2.397863886	0.000229836	up
647	A_64_P020269	Hist1h2bh	2.396916193	0.00014661	up
648	A_64_P007472	Zfp592	2.394860298	0.000286782	up
649	A_44_P138800	Gcap14	2.394832078	0.000102154	up
650	A_64_P020238	Hist1h2bf	2.393634708	2.37825E-05	up
651	A_64_P068648	Foxj3	2.392585476	0.000534173	up
652	A_64_P029377	Hrh4	2.392148358	0.01007296	up
653	A_64_P113414	Kcnd3	2.391853896	0.000492507	up
654	A_64_P158345	Ddx60	2.390044312	0.000718223	up
655	A_64_P084896	Serpinb8	2.389436122	0.030556274	up
656	A_64_P064254	RGD1562534	2.389093528	0.002207605	up
657	A_64_P007030	N4bp1	2.388043307	2.82402E-05	up
658	A_44_P478319	Lonrf3	2.387484996	1.36855E-05	up
659	A_44_P339818	Ralgds	2.386321293	0.003228829	up
660	A_64_P127634	Ercc6	2.38396221	0.015508906	up
661	A_44_P262593	Ankrd2	2.383166529	0.001231009	up
662	A_42_P730229	Myo1d	2.38293869	0.015259495	up
663	A_64_P031460	Vom2r3	2.381387398	0.000173854	up
664	A_44_P991335	Capns2	2.380497697	0.001518055	up
665	A_64_P051229	RGD1565025	2.380430432	0.00464101	up
666	A_64_P286599	Vwf	2.379951983	0.010702635	up
667	A_64_P119675	Jund	2.378796734	0.000230431	up
668	A_64_P005029	Pcp4l1	2.372791552	0.022014237	up
669	A_44_P146821	Hivep2	2.368778005	1.45333E-05	up
670	A_64_P042278	Crem	2.368637353	0.000250744	up
671	A_64_P060485	LOC686683	2.367648639	0.027757606	up
672	A_64_P020741	Rsph1	2.364685304	0.000500361	up
673	A_42_P638494	Nppb	2.36072414	0.011255025	up
674	A_64_P041507	Slamf8	2.360472486	0.000437175	up
675	A_64_P155925	Ckm	2.357972403	0.004832955	up
676	A_44_P135224	Plk3	2.357279727	0.000140691	up
677	A_64_P057211	Tex101	2.355998031	0.038109324	up
678	A_64_P041481	Mep1b	2.355267298	0.022090467	up
679	A_44_P262078	Gfi1	2.354856259	0.001915547	up

680	A_64_P128219	Egr3	2.353911369	0.019135097	up
681	A_64_P161180	Rnf224	2.353104406	0.004946837	up
682	A_64_P137941	Nodal	2.352890204	0.000382847	up
683	A_42_P555795	Nfat5	2.352632917	2.60959E-06	up
684	A_64_P138008	Adra2b	2.351049911	0.029147701	up
685	A_43_P15587	Map3k8	2.35000474	1.21996E-05	up
686	A_44_P441252	Slc5a10	2.349673011	0.003201672	up
687	A_64_P071218	RGD1564031	2.349197325	0.000302918	up
688	A_44_P283812	Fam46c	2.345247963	1.06357E-06	up
689	A_64_P062980	Gzmb	2.340923843	1.31287E-05	up
690	A_43_P12927	Dusp1	2.340844661	6.50395E-05	up
691	A_64_P082037	LOC679127	2.340334587	0.001519028	up
692	A_44_P1053032	Rnf150	2.213508428	0.049471765	up
693	A_43_P20143	Ceacam20	2.213129952	0.024385162	up
694	A_42_P614984	Ucp1	2.21141762	0.000309253	up
695	A_64_P002126	Cacna1d	2.207783492	0.003680373	up
696	A_44_P147328	RGD1560248	2.20777885	0.000394165	up
697	A_44_P386799	Nudt9	2.205260877	6.97049E-05	up
698	A_44_P287089	Gmfb	2.204529323	7.22233E-06	up
699	A_64_P086021	RGD1561465	2.203857027	0.000255893	up
700	A_64_P025915	Gramd2	2.202778858	0.012170768	up
701	A_64_P334603	Tcrb	2.196839138	0.000570175	up
702	A_64_P085767	Utp15	2.194599478	9.90025E-07	up
703	A_64_P012291	Pdzd7	2.194213892	0.026152464	up
704	A_64_P033121	Rasa4	2.193822292	2.87817E-05	up
705	A_44_P555271	Mmp12	2.19257572	1.57091E-06	up
706	A_44_P809374	Clcf1	2.19120784	4.07172E-06	up
707	A_64_P084995	Tnfrsf26	2.190882329	1.30696E-05	up
708	A_44_P1036481	Prrc2c	2.186337818	0.00563102	up
709	A_44_P448710	Slc44a1	2.18205006	0.002833277	up
710	A_44_P464196	Cd97	2.180650405	0.000596932	up
711	A_44_P878617	Slc41a2	2.179419978	8.22446E-05	up
712	A_64_P105417	Fcrla	2.17889096	3.75532E-05	up
713	A_64_P039888	Abcb1b	2.178637799	3.69326E-05	up
714	A_64_P111893	Apol9a	2.178239114	6.79371E-05	up
715	A_64_P110103	Sod2	2.17027571	2.12174E-05	up
716	A_64_P101817	Sec24a	2.167962797	2.77807E-06	up
717	A_43_P16141	Nphs2	2.167774866	0.002626293	up
718	A_64_P126757	Bcl6b	2.166856579	5.87025E-05	up
719	A_44_P468141	Plaur	2.16665783	0.000493341	up
720	A_42_P800859	Rapgef2	2.165165542	2.02479E-05	up
721	A_43_P11538	Hk1	2.16302599	0.002467376	up
722	A_64_P139519	Tnfsf8	2.160674674	0.001206199	up
723	A_44_P128564	Stam	2.160291006	0.001782933	up
724	A_44_P545867	Unc119b	2.157887321	0.000538184	up
725	A_44_P445972	Med13	2.154598916	0.000021533	up
726	A_64_P210110	Schip1	2.153380851	0.041838514	up
727	A_64_P031740	MGC116197	2.153132744	0.045821784	up
728	A_43_P23363	Spata22	2.152904264	4.99816E-05	up
729	A_64_P124791	Timm8a1	2.150562659	1.48405E-05	up
730	A_64_P061061	Lmbrd2	2.149789943	0.000954481	up

731	A_44_P414996	Gfpt1	2.148626424	0.000317981	up
732	A_44_P1054213	Hspa5	2.148398322	0.000240902	up
733	A_43_P14700	LOC691125	2.145279614	0.000311709	up
734	A_43_P11685	Id2	2.14507313	0.001327988	up
735	A_64_P036676	Klf6	2.143904536	0.004596205	up
736	A_44_P307978	Cited2	2.143164615	0.000266547	up
737	A_64_P055099	Clec2dl1	2.142416287	1.33299E-05	up
738	A_64_P101185	Slc45a1	2.141672525	0.034532888	up
739	A_44_P638191	Otud1	2.135898119	0.001352982	up
740	A_64_P147564	LOC689800	2.131700368	1.37854E-05	up
741	A_64_P150525	Nup98	2.129896511	0.000134251	up
742	A_42_P533865	Anks4b	2.129163442	0.000514301	up
743	A_64_P053790	Adra1a	2.128293818	0.040376848	up
744	A_64_P110932	Rps6ka2	2.128163314	0.033324949	up
745	A_43_P12767	Pdlim5	2.127304175	0.000168155	up
746	A_64_P118992	Pnck	2.126537555	0.019641536	up
747	A_64_P076342	AA926063	2.12341349	9.09519E-05	up
748	A_64_P054048	Pgm3	2.119156889	0.003723216	up
749	A_43_P19848	RGD1309870	2.11857387	0.000277569	up
750	A_44_P1049055	Hspa4l	2.116009836	6.27141E-05	up
751	A_44_P191285	RGD1564171	2.114258092	5.52597E-06	up
752	A_64_P145092	Olr1240	2.114062262	0.015552232	up
753	A_64_P084243	Clcn5	2.113836121	0.02188998	up
754	A_44_P450758	Qtrt1	2.113626803	0.000297818	up
755	A_64_P013157	RGD1564463	2.113396314	1.51254E-05	up
756	A_64_P067654	Aldh3a1	2.112306078	0.031398455	up
757	A_44_P459858	Dysfip1	2.112138441	0.001813262	up
758	A_44_P356829	RGD1306484	2.110108574	0.013208357	up
759	A_44_P192134	Mapk8	2.110047778	0.001648716	up
760	A_64_P037050	Mkx	2.108725636	0.004222089	up
761	A_64_P021094	LOC689103	2.10867901	0.000306527	up
762	A_44_P101700	Stx11	2.105812132	7.99319E-05	up
763	A_64_P089662	LOC10036480(	2.105386836	0.000198251	up
764	A_64_P098464	Slc9a7	2.104929625	0.000107038	up
765	A_64_P006097	Gjc2	2.102186743	0.001003303	up
766	A_64_P056846	Gclc	2.100746868	2.5863E-06	up
767	A_64_P057847	Alox12b	2.099437248	0.004595181	up
768	A_64_P016911	Frmd4a	2.09872213	0.00031693	up
769	A_64_P064088	Hdhd2	2.097750357	0.007236244	up
770	A_64_P081967	Fam53c	2.096388742	0.040050623	up
771	A_43_P15356	Mgea5	2.094174507	0.00027355	up
772	A_44_P238246	Ilf3	2.094083786	2.59272E-05	up
773	A_44_P1033194	Slc7a6os	2.094080641	0.000141783	up
774	A_64_P110609	LOC691298	2.092172103	0.002577622	up
775	A_64_P053896	Alkbh7	2.091401908	0.000694314	up
776	A_64_P032574	Hk2	2.090249368	0.004732291	up
777	A_64_P138543	Bmp2k	2.088833258	2.48458E-05	up
778	A_43_P15993	Itgam	2.086879067	3.28618E-06	up
779	A_44_P537673	Zbtb43	2.086555796	4.32898E-05	up
780	A_42_P599062	Plg	2.082562799	0.001277662	up
781	A_44_P503471	Itga7	2.082535179	0.000156007	up

782	A_64_P001089	Adipoq	2.082414265	0.003756412	up
783	A_64_P049432	LOC10036119$	2.081485247	7.48254E-06	up
784	A_44_P1050015	Vcpip1	2.080541405	0.001228651	up
785	A_64_P158913	Irak3	2.079964156	0.000140931	up
786	A_42_P756706	Riok3	2.077464753	8.40322E-06	up
787	A_44_P142817	Fem1b	2.077178455	0.000805504	up
788	A_64_P136036	Ankk1	2.075652118	0.012171549	up
789	A_42_P639027	Mmp14	2.075370434	0.000990033	up
790	A_64_P019408	LOC685030	2.075068075	2.86377E-05	up
791	A_44_P536275	Adamts4	2.073476505	9.40508E-05	up
792	A_64_P031481	Slc26a9	2.071444165	0.001244083	up
793	A_42_P540950	Ier2	2.06712505	9.48974E-05	up
794	A_44_P216395	Gadd45a	2.067039082	0.000946033	up
795	A_64_P123050	Lta	2.065048979	1.41184E-06	up
796	A_44_P180240	Tmem185b	2.064187705	4.39747E-05	up
797	A_64_P084851	Rcan2	2.062027919	0.00033291	up
798	A_64_P039112	Max	2.06138384	0.000291256	up
799	A_42_P553444	Pcsk5	2.060003802	6.70752E-06	up
800	A_44_P196824	Serpinb9	2.058946486	0.004672242	up
801	A_64_P042402	LOC691931	2.058171688	0.000376709	up
802	A_64_P125506	Psgb1	2.056004848	0.002249262	up
803	A_64_P000904	Kcna3	2.053158398	0.012229234	up
804	A_64_P058923	Tes	2.051694129	6.55065E-05	up
805	A_64_P018633	Muc2	2.049075155	0.001655464	up
806	A_44_P112371	Nkrf	2.048569066	0.000745923	up
807	A_44_P1004376	Ins2	2.047414065	0.031408105	up
808	A_44_P498972	Eya4	2.047053678	0.006804486	up
809	A_44_P178252	Ccl21	2.043955943	0.00099355	up
810	A_64_P103238	Zfp52	2.043653582	3.32065E-05	up
811	A_64_P019160	Etnk1	2.039140713	0.000189502	up
812	A_44_P436040	Rassf6	2.03900908	0.000215907	up
813	A_44_P1008245	Casc1	2.038718143	0.000975923	up
814	A_64_P232269	Vom2r4	2.036525671	2.69968E-05	up
815	A_44_P263015	Sntb1	2.035006543	2.85745E-05	up
816	A_64_P058615	Uncx	2.034936581	0.000103813	up
817	A_64_P072268	Ahi1	2.034268344	9.9631E-06	up
818	A_64_P098006	Fitm1	2.033709337	0.020666192	up
819	A_64_P027487	LOC690137	2.032629965	0.000146739	up
820	A_64_P065837	RGD1561557	2.032550222	0.010746869	up
821	A_43_P12143	Clip2	2.032486355	2.43439E-05	up
822	A_44_P519620	Litaf	2.031637723	1.85871E-05	up
823	A_64_P061716	LOC10036384	2.029263672	0.013757913	up
824	A_43_P12209	Stat5b	2.025917839	0.000195424	up
825	A_42_P518855	Bcl2a1d	2.02444133	3.01792E-06	up
826	A_64_P080479	Pdgfb	2.022187633	0.000555594	up
827	A_64_P037577	Lmtk2	2.020768889	3.59038E-05	up
828	A_64_P038342	Snrpn	2.018843391	0.010907087	up
829	A_64_P141251	LOC681152	2.018785085	0.000273545	up
830	A_44_P993370	Fermt2	2.017338246	1.47303E-05	up
831	A_44_P320752	Rasl11a	2.017197161	0.000471368	up
832	A_44_P879764	B4galt1	2.01710311	0.000125274	up

833	A_44_P360972	Vom2r70	2.016110761	4.48936E-05	up
834	A_64_P093452	RGD1305464	2.013741966	0.000015335	up
835	A_44_P380575	Slc7a5	2.01352958	3.93462E-06	up
836	A_44_P461085	Gtf2h1	2.012472403	0.00224901	up
837	A_44_P351723	Cldn10	2.010952912	0.001006121	up
838	A_44_P605194	Cwc25	2.01023686	0.000232958	up
839	A_43_P19889	Angptl6	2.008877789	0.01380705	up
840	A_64_P020834	LOC680039	2.005753943	0.003005326	up
841	A_44_P395572	Nr1d2	2.005650648	0.000386338	up
842	A_64_P113019	Hsd17b13	2.005487536	0.001572687	up
843	A_44_P1012606	Npap60	2.004829664	1.56409E-05	up
844	A_64_P157059	Usp18	2.003562711	0.001086656	up
845	A_64_P048191	Pcnxl2	2.00061732	0.037725751	up
846	A_44_P544256	Erc1	20.37195457	5.71842E-06	down
847	A_43_P22351	Slc46a3	15.79307397	4.26834E-06	down
848	A_44_P272070	Ntng2	13.91610277	0.000342188	down
849	A_44_P491942	Lrat	11.8408666	0.001289639	down
850	A_64_P133967	Abcd2	11.07752159	1.55517E-06	down
851	A_43_P18802	Pask	11.05870643	6.61644E-06	down
852	A_42_P833264	Zfp278	10.67769692	0.000318626	down
853	A_64_P043406	Lrrc56	10.54475302	5.14539E-05	down
854	A_44_P480830	Zfp426	10.53710126	4.24506E-06	down
855	A_44_P794669	Mllt4	10.4498737	0.013365767	down
856	A_44_P236738	Pole2	9.124418803	2.70275E-05	down
857	A_44_P397423	Tmcc3	8.659040679	1.56943E-06	down
858	A_64_P125245	Gpr34	8.450483567	0.000142631	down
859	A_64_P025108	Cebpa	6.765024692	0.002623186	down
860	A_44_P294650	Brca1	6.755390806	0.000225082	down
861	A_43_P22402	Fbxo5	6.740801459	0.004594025	down
862	A_64_P098031	Tfap4	6.655240646	8.21836E-05	down
863	A_44_P732517	Zadh2	6.55077017	1.45159E-05	down
864	A_44_P211061	Etv1	6.296350265	2.77721E-05	down
865	A_42_P454311	Dixdc1	6.193593792	1.62623E-07	down
866	A_64_P072755	Olr434	6.180269141	0.001070225	down
867	A_44_P238556	Mcpt2	6.021726984	0.020609007	down
868	A_64_P164804	Gpr18	5.977241499	4.0531E-06	down
869	A_42_P596942	Filip1	5.865068535	5.27974E-07	down
870	A_44_P231935	Rad51	5.851946311	0.001986443	down
871	A_44_P248358	Tk2	5.83642038	0.005073134	down
872	A_64_P001259	Ntng1	5.770459481	0.025725385	down
873	A_42_P677662	Ttc30b	5.746877246	0.001008966	down
874	A_64_P163754	Sec14l4	5.628899148	0.027427852	down
875	A_44_P215253	N4bp2l1	5.536743674	0.000961267	down
876	A_44_P170018	LOC304558	5.519719165	0.000465731	down
877	A_44_P170368	Fmo4	5.484425356	4.48862E-05	down
878	A_64_P138291	Cenpk	5.478696934	0.000145922	down
879	A_44_P102876	Fam173b	5.476989318	0.0094182	down
880	A_44_P422080	Alms1	5.394979833	0.000809954	down
881	A_64_P136056	Olr883	5.319539892	0.000935934	down
882	A_64_P021686	Epor	5.300459133	7.31064E-05	down
883	A_64_P029726	Add3	5.292816853	9.52055E-05	down

884	A_64_P160175	Enam	5.200980877	0.008579321	down
885	A_44_P606174	Bcl2l14	5.136519303	0.000160411	down
886	A_44_P443479	Mns1	5.132854979	2.02079E-05	down
887	A_64_P166489	Napepld	5.116330165	2.62411E-05	down
888	A_44_P276338	Mcm6	5.068019703	0.000181065	down
889	A_64_P126896	Ly49i9	5.05331682	0.000068132	down
890	A_44_P223446	Mcm2	5.037101376	0.000548641	down
891	A_64_P068624	Trim45	5.005579729	0.00016723	down
892	A_64_P069071	Lpar6	5.002347681	5.28797E-06	down
893	A_64_P133421	Eid2b	4.990603198	0.013553476	down
894	A_42_P459431	Bphl	4.928404373	1.92727E-06	down
895	A_43_P12191	Pdcd4	4.903476623	0.000650343	down
896	A_64_P017329	Syk	4.822743895	0.000362879	down
897	A_64_P111214	Pik3ca	4.793500003	0.004137928	down
898	A_42_P670580	Vipr1	4.750788563	0.00003351	down
899	A_64_P097764	Dtwd2	4.699013133	0.000605368	down
900	A_44_P389462	Zmym3	4.689105375	1.05843E-05	down
901	A_43_P12819	Pola2	4.646922766	0.000027004	down
902	A_44_P492013	Map3k4	4.623366827	0.000021935	down
903	A_64_P127312	LOC691166	4.558697985	0.001440102	down
904	A_64_P038352	Appl2	4.532055724	0.001704744	down
905	A_64_P163160	LOC686506	4.529490047	0.003111538	down
906	A_42_P586883	Skp2	4.515147443	0.00072121	down
907	A_42_P789204	Ypel3	4.512651394	1.50798E-05	down
908	A_64_P041937	Robo3	4.488092883	0.016517513	down
909	A_64_P065492	RGD1565222	4.485496751	1.70474E-05	down
910	A_64_P127337	Per3	4.485013621	0.00037017	down
911	A_64_P059227	Nudt12	4.413732513	4.35764E-05	down
912	A_44_P196856	Cir1	4.397718318	0.001942352	down
913	A_64_P088427	Hdac8	4.376906504	0.001092538	down
914	A_42_P623374	Tnrc6b	4.368926293	0.000027893	down
915	A_64_P016578	Rassf7	4.361899208	0.002303862	down
916	A_44_P405790	Cnpy3	4.326395831	0.001034597	down
917	A_64_P102315	LOC691777	4.320128824	0.001301309	down
918	A_44_P412647	Gins2	4.31733278	4.90329E-05	down
919	A_64_P019798	RGD1306227	4.314302095	3.81077E-06	down
920	A_44_P1008591	Tmem218	4.312015408	7.67244E-05	down
921	A_44_P532473	Traip	4.293811229	7.79496E-06	down
922	A_44_P172140	Hfe	4.284198521	0.002413491	down
923	A_44_P267501	Cep57l1	4.277870076	0.000073818	down
924	A_64_P001710	Mblac2	4.259381047	0.000179487	down
925	A_44_P273885	Mettl20	4.248547516	3.22905E-06	down
926	A_64_P073653	Zfp395	4.244748665	6.80347E-06	down
927	A_64_P132841	Gtf2i	4.244704728	0.00044797	down
928	A_43_P16635	Lrrc4b	4.225428933	0.006833686	down
929	A_44_P203665	Ccng2	4.218243955	0.000229009	down
930	A_64_P112734	RGD1562342	4.211428774	0.000052415	down
931	A_44_P438718	Stil	4.19678823	0.001053421	down
932	A_64_P006002	Ppp1r3b	4.182121376	0.002611659	down
933	A_43_P19276	Cep68	4.17475249	3.78906E-07	down
934	A_44_P194531	Ubac1	4.10477772	1.81045E-05	down

935	A_44_P551169	B4galt4	4.102586541	2.76291E-06	down
936	A_44_P295051	Tmem9	4.096268831	0.017917057	down
937	A_64_P027742	Ezh1	4.062755093	0.00748139	down
938	A_43_P20515	Zfand1	4.05814946	0.001542987	down
939	A_44_P517591	Eef2k	4.040430358	0.003266711	down
940	A_44_P325911	Sirt4	4.005221972	0.000155748	down
941	A_64_P036621	Ears2	3.982669509	0.020058797	down
942	A_44_P256457	Pan2	3.96196417	0.00051346	down
943	A_44_P300093	Ttc15	3.956258882	0.000820023	down
944	A_64_P023541	Atg16l1	3.955509856	0.018765726	down
945	A_64_P157244	Sfxn2	3.92696961	0.000760751	down
946	A_64_P040061	Gper	3.92635658	3.59472E-05	down
947	A_44_P299721	Rrm1	3.921561611	0.003397356	down
948	A_44_P100443	Ung	3.914917304	0.00080489	down
949	A_44_P252441	St3gal2	3.900780809	0.000143852	down
950	A_43_P22136	Nadkd1	3.888517056	3.03766E-05	down
951	A_64_P033082	Polr2e	3.8760642	0.001200043	down
952	A_44_P176051	Icam2	3.871053196	0.000668831	down
953	A_44_P271062	Tmem70	3.870677386	2.39723E-06	down
954	A_44_P222004	Il15	3.867443012	0.000012811	down
955	A_64_P165327	RGD1562550	3.860779428	0.034010873	down
956	A_44_P321669	Map3k3	3.853048466	0.00027034	down
957	A_44_P945723	Zc3h12d	3.842630794	9.65046E-06	down
958	A_64_P072748	Cage1	3.842335866	0.000100207	down
959	A_64_P032018	Mettl8	3.839285286	0.003868221	down
960	A_42_P772157	Il6r	3.801015566	0.000152655	down
961	A_64_P153134	LOC688310	3.797544841	9.24486E-06	down
962	A_64_P091948	Man2a2	3.794604257	0.023308022	down
963	A_44_P183638	Lilrb3	3.789672122	0.000106038	down
964	A_64_P124459	Olr1584	3.775886502	3.16523E-05	down
965	A_44_P1011038	Fbxo31	3.766187953	2.95899E-06	down
966	A_64_P107836	LOC683603	3.764987478	3.07965E-05	down
967	A_64_P001971	LOC690217	3.747456851	0.000788701	down
968	A_64_P001147	Zfp280d	3.730311652	0.003712673	down
969	A_42_P837441	Fam53b	3.726383096	6.15109E-05	down
970	A_44_P554679	Mtss1	3.716732006	5.13394E-06	down
971	A_43_P13378	Bmf	3.716068339	9.84458E-06	down
972	A_64_P152124	Txndc3	3.715603525	3.46632E-05	down
973	A_44_P105749	Rimbp3	3.712607585	2.65041E-07	down
974	A_64_P026883	Ick	3.710492866	2.63207E-05	down
975	A_42_P555701	Inpp5f	3.705827343	1.00671E-05	down
976	A_64_P061602	Cbx3	3.676735684	0.000220456	down
977	A_64_P133816	LOC311352	3.676434461	0.00026897	down
978	A_64_P113725	Slc1a3	3.674019627	1.97677E-05	down
979	A_64_P140612	Ccdc34	3.667701089	4.83457E-07	down
980	A_44_P107552	Tomm40b	3.66535703	0.000233186	down
981	A_64_P029775	RGD1304879	3.65941026	0.00536293	down
982	A_64_P026032	Olr219	3.658203334	0.002146421	down
983	A_44_P342750	Xrcc6bp1	3.649353652	0.00016458	down
984	A_43_P19787	LOC679811	3.649098009	0.006659237	down
985	A_64_P299808	Hmgn5	3.648601023	0.02731565	down

986	A_44_P304009	Fam111a	3.639008279	1.00403E-05	down
987	A_42_P688442	Usf1	3.632267615	0.000724185	down
988	A_44_P426608	Wdhd1	3.627293455	1.73127E-05	down
989	A_44_P555399	Fignl1	3.616045005	0.000115794	down
990	A_64_P032643	Hells	3.602361589	9.56372E-05	down
991	A_64_P038232	LOC10036617:	3.589009727	1.57792E-06	down
992	A_42_P480224	Gpd1	3.586208994	4.10859E-05	down
993	A_44_P226597	Pycard	3.58365834	9.64541E-05	down
994	A_64_P397198	Mms22l	3.574415879	0.019640662	down
995	A_64_P312700	Arhgef7	3.572922366	0.003059455	down
996	A_64_P050079	LOC10018898	3.570905941	0.000713876	down
997	A_42_P522582	Usp43	3.562326622	9.03122E-05	down
998	A_42_P472375	Pdk2	3.559834902	5.01408E-05	down
999	A_44_P686987	Gpr160	3.549838769	0.000736082	down
1000	A_64_P126291	Pex26	3.544186035	0.000475178	down
1001	A_44_P637827	Fam117b	3.53992714	1.95048E-05	down
1002	A_44_P1019923	Casp2	3.52597277	1.15324E-05	down
1003	A_44_P959077	Rad51c	3.522614091	3.22767E-05	down
1004	A_64_P000121	Rcbtb1	3.509592674	0.00167827	down
1005	A_64_P151046	Trub2	3.48930522	0.005097051	down
1006	A_64_P121058	Smcr7	3.487478686	1.15334E-06	down
1007	A_64_P095368	Ctps	3.482669814	0.005021264	down
1008	A_64_P065676	Hipk2	3.476595773	0.000432734	down
1009	A_42_P669870	Gtpbp3	3.460596655	2.38548E-05	down
1010	A_44_P289862	Abhd6	3.459030177	0.001421542	down
1011	A_42_P555426	Fgfbp3	3.453930053	1.08056E-06	down
1012	A_44_P326259	Iffo1	3.453027443	0.001749172	down
1013	A_64_P047206	Deptor	3.446266015	0.000205906	down
1014	A_44_P139042	Zfp579	3.434911194	0.000180211	down
1015	A_43_P20954	Wdr96	3.42721904	0.000499339	down
1016	A_44_P246311	Rmnd5b	3.4238061	0.000173823	down
1017	A_64_P099307	Tcf7l2	3.422169773	2.31928E-06	down
1018	A_64_P297053	Ush2a	3.41548755	0.013457129	down
1019	A_64_P308666	Ttc21b	3.400724103	0.002548598	down
1020	A_42_P583546	Lims2	3.397854714	0.000344854	down
1021	A_64_P097193	Topbp1	3.397752656	3.06896E-05	down
1022	A_64_P117086	Abcc10	3.390789599	1.67751E-05	down
1023	A_64_P029831	Irf2bpl	3.381983607	4.42088E-07	down
1024	A_44_P977318	Tfcp2l1	3.380701878	8.13858E-05	down
1025	A_42_P485589	Map2k5	3.380414833	0.000415991	down
1026	A_44_P265058	Gmnn	3.379465532	0.000138231	down
1027	A_64_P038107	Apobec1	3.37290262	5.07262E-05	down
1028	A_44_P1050144	Plk1s1	3.340202958	5.92559E-05	down
1029	A_64_P159170	LOC689629	3.339547034	0.025042356	down
1030	A_44_P416751	Rpa1	3.336449421	0.007791807	down
1031	A_44_P156262	Myod1	3.336191647	5.08656E-06	down
1032	A_44_P1044030	Klhdc9	3.333991054	0.000651401	down
1033	A_42_P707010	Zfp426l	3.33156375	1.78281E-05	down
1034	A_44_P1007561	Vars2	3.327794855	2.09357E-05	down
1035	A_64_P033214	Top2a	3.325979016	0.001718781	down
1036	A_64_P023401	Fut4	3.32348544	0.000161459	down

1037	A_64_P032579	Rab27a	3.319839128	1.02281E-05	down
1038	A_44_P680622	Clspn	3.318125223	0.000221863	down
1039	A_64_P069051	P2ry1	3.317671168	9.07003E-07	down
1040	A_64_P094362	Cabin1	3.309329859	0.00012894	down
1041	A_64_P140275	Cdan1	3.300885662	0.000019093	down
1042	A_42_P664472	Rasa3	3.29457682	3.79944E-06	down
1043	A_44_P148383	RGD1564952	3.288662214	6.1142E-06	down
1044	A_44_P491824	Camk2g	3.273939662	0.001262371	down
1045	A_44_P250654	Habp4	3.267759859	0.000193266	down
1046	A_64_P005254	LOC689656	3.267105404	0.000223909	down
1047	A_43_P11817	Ghr	3.260278407	1.07868E-05	down
1048	A_44_P217866	LOC310902	3.260133026	0.000125055	down
1049	A_64_P116331	Eif2c4	3.259995335	0.038813118	down
1050	A_44_P1041312	Fes	3.25916336	5.35599E-07	down
1051	A_43_P21333	Mtmr4	3.256111869	0.000200616	down
1052	A_44_P1047628	Pars2	3.254603137	9.06499E-07	down
1053	A_44_P243826	Adck5	3.250251448	0.038168851	down
1054	A_44_P227616	Engase	3.250119131	2.27629E-05	down
1055	A_42_P772136	Fam60a	3.249869454	0.00066013	down
1056	A_42_P643272	Gemin2	3.249018969	3.61571E-06	down
1057	A_44_P279116	Dyrk4	3.234060273	5.33892E-05	down
1058	A_64_P072204	LOC691979	3.234023958	4.65358E-05	down
1059	A_44_P243004	Amigo2	3.225636271	0.002102386	down
1060	A_64_P125853	Kbtbd3	3.218659805	2.48462E-05	down
1061	A_43_P15508	Rbl2	3.201835081	3.15368E-07	down
1062	A_64_P153639	Cnnm3	3.199861584	1.95316E-05	down
1063	A_44_P534154	Cnpy4	3.196906463	0.001884393	down
1064	A_44_P387246	Sin3a	3.1814668	0.001341419	down
1065	A_44_P455101	RGD1561102	3.175965484	0.006505568	down
1066	A_43_P12090	Pold1	3.170781945	2.42959E-06	down
1067	A_44_P377156	Cntnap1	3.168939903	1.6103E-06	down
1068	A_44_P419810	Lrr1	3.168649972	1.40814E-06	down
1069	A_44_P494591	Sall1	3.167103979	0.005278463	down
1070	A_44_P623236	Rbak	3.161016429	8.61838E-05	down
1071	A_44_P480573	Cytip	3.1604157	5.71662E-05	down
1072	A_44_P748197	Efcab11	3.15970711	0.000714282	down
1073	A_44_P227121	Txndc16	3.154997364	0.000021624	down
1074	A_44_P335703	Toag1	3.152990449	0.000650749	down
1075	A_43_P11186	Aifm2	3.146416885	0.000126249	down
1076	A_64_P160663	Ylpm1	3.144005119	4.76446E-06	down
1077	A_64_P138641	Zfp472	3.128450188	0.043992745	down
1078	A_64_P037631	Ar	3.126784519	0.007996956	down
1079	A_42_P573118	RGD1309594	3.125935116	0.000673907	down
1080	A_44_P683157	Zfp133	3.125923994	0.00062305	down
1081	A_44_P792784	Htr2c	3.125260757	0.014428672	down
1082	A_44_P634770	Cxxc5	3.114797126	0.000254819	down
1083	A_44_P243847	RGD1308299	3.113892199	6.04778E-05	down
1084	A_44_P1058932	Akr1b7	3.11039463	0.000812715	down
1085	A_64_P236773	Nudt6	3.105503585	4.33455E-05	down
1086	A_44_P217604	Msh2	3.098799403	0.000236898	down
1087	A_64_P126265	Rbm20	3.095978745	0.024932811	down

1088	A_43_P10757	Fam122b	3.093610001	1.97803E-06	down
1089	A_44_P497033	Was	3.09102188	0.002557418	down
1090	A_44_P637482	Slc25a42	3.089604777	0.000677549	down
1091	A_42_P459212	Tnfrsf21	3.088425079	0.000246802	down
1092	A_64_P084188	Nod1	3.086898614	0.000372236	down
1093	A_44_P100207	Cnp	3.084676649	0.006399936	down
1094	A_64_P024138	Sbk1	3.083445471	0.000118815	down
1095	A_64_P012631	Rbl1	3.080039956	6.24342E-06	down
1096	A_44_P930446	She	3.079888665	9.83286E-05	down
1097	A_44_P295118	Fam53a	3.078931491	0.000289367	down
1098	A_64_P273084	RGD1566085	3.078780611	0.00291871	down
1099	A_44_P1018447	Micall2	3.07638913	8.31952E-05	down
1100	A_44_P489273	Sucnr1	3.074398619	1.22374E-05	down
1101	A_43_P16005	Prkd3	3.072955908	0.002412017	down
1102	A_44_P523198	Akap8l	3.070496446	0.000566509	down
1103	A_44_P310792	Cog8	3.067818574	8.88148E-05	down
1104	A_44_P323430	Nme7	3.065799324	0.001595632	down
1105	A_44_P415003	Rpusd3	3.061346826	1.82583E-06	down
1106	A_44_P244040	Gtpbp5	3.060574882	0.004758864	down
1107	A_44_P435059	Hmgb3	3.05712823	6.3411E-06	down
1108	A_44_P325268	Scfd2	3.056166196	5.54579E-07	down
1109	A_64_P056537	Dscc1	3.055264326	0.000348718	down
1110	A_44_P325782	Hsd3b7	3.038224337	0.010793841	down
1111	A_64_P068162	Zfp161	3.034832936	0.002547215	down
1112	A_64_P041763	Lrrcc1	3.026606883	0.00347531	down
1113	A_64_P077940	LOC366300	3.025753584	0.000292608	down
1114	A_44_P714007	Cidec	3.025294731	5.93931E-06	down
1115	A_44_P149293	Trim23	3.023959673	0.002102047	down
1116	A_43_P15924	Pola1	3.023226214	0.001345818	down
1117	A_64_P102708	Ddi2	3.020983002	6.04987E-05	down
1118	A_64_P122589	Lman2l	3.005719888	0.000101175	down
1119	A_43_P22126	Ptcd1	3.003825211	0.001025746	down
1120	A_44_P294753	RGD621098	2.993692553	6.32025E-05	down
1121	A_44_P208049	Sh2b1	2.993168782	0.007243137	down
1122	A_64_P021591	Tpm1	2.989164582	1.82321E-05	down
1123	A_44_P168602	Rbm43	2.974451662	0.002159753	down
1124	A_64_P071515	Fkbp9	2.973871616	0.000395273	down
1125	A_42_P809596	Mfsd9	2.971336985	1.90944E-05	down
1126	A_44_P400922	Rad52	2.968463416	4.24237E-06	down
1127	A_44_P187120	LOC10036190	2.963648742	0.000116715	down
1128	A_44_P487778	RGD1309138	2.961542931	8.95594E-05	down
1129	A_44_P867745	Ggnbp1	2.95294346	0.008494699	down
1130	A_44_P128007	Slco2b1	2.949028299	0.000354886	down
1131	A_64_P002814	Grhl3	2.946056775	1.38406E-05	down
1132	A_44_P355850	Coro6	2.945630221	0.000473038	down
1133	A_64_P258485	Mapk14	2.940649831	1.05265E-05	down
1134	A_64_P035724	Fancb	2.939814653	0.000237399	down
1135	A_44_P606344	Bend6	2.932429654	0.000199702	down
1136	A_44_P424552	Plscr4	2.931234319	0.008572854	down
1137	A_64_P058251	RGD1566386	2.925949494	1.09408E-05	down
1138	A_43_P21489	Zmynd10	2.924196512	0.005091129	down

1139	A_64_P031516	LOC362863	2.901905354	0.010261715	down
1140	A_44_P267128	Tmpo	2.900201153	7.16139E-05	down
1141	A_44_P501408	Phemx	2.897658076	0.000397571	down
1142	A_64_P032986	Lpgat1	2.896829285	0.004679542	down
1143	A_64_P049648	RGD1562963	2.895753436	0.029801132	down
1144	A_44_P100197	Zfp799	2.892509705	0.004052403	down
1145	A_44_P667524	RGD1565059	2.89053091	6.10009E-05	down
1146	A_44_P274762	Cpped1	2.883827626	0.000513887	down
1147	A_44_P497553	Gcnt1	2.88310684	0.000386207	down
1148	A_64_P020456	Rad51b	2.880834336	0.005452602	down
1149	A_44_P302405	Tia1	2.878747261	7.47917E-05	down
1150	A_44_P996410	Hspb6	2.868857946	0.000871429	down
1151	A_64_P008777	Ccdc28a	2.867238339	2.15379E-05	down
1152	A_44_P337064	Slc36a2	2.866944812	3.44031E-06	down
1153	A_44_P501898	Chtf18	2.866116329	0.004676183	down
1154	A_64_P166440	LOC685909	2.862945089	6.74855E-06	down
1155	A_42_P713907	Sgk196	2.852190018	0.002343017	down
1156	A_44_P1037410	Ophn1	2.849766734	0.010578922	down
1157	A_64_P082461	Zfp748	2.848926167	5.53609E-05	down
1158	A_42_P629599	Haus1	2.848876536	5.62755E-05	down
1159	A_44_P997444	Tube1	2.846357222	0.003018659	down
1160	A_43_P17311	Tmem129	2.833354661	0.000438819	down
1161	A_64_P138636	Nr2f1	2.832668415	1.11011E-05	down
1162	A_43_P18024	Ikbip	2.831720748	0.004891982	down
1163	A_44_P405139	Arhgef11	2.827225584	0.000469478	down
1164	A_44_P550191	Slc2a8	2.827173065	0.001237571	down
1165	A_64_P021440	Slc10a6	2.824274078	0.000342069	down
1166	A_44_P342166	Plcg1	2.821657668	0.000683498	down
1167	A_44_P477568	Sdccag8	2.819375819	0.001597112	down
1168	A_42_P818546	Fhod1	2.817948671	0.000152829	down
1169	A_64_P152014	Lfng	2.814806598	1.75403E-05	down
1170	A_44_P1018246	Xpa	2.814491647	8.23761E-05	down
1171	A_44_P594610	Cdca8	2.813356803	0.001268804	down
1172	A_64_P140936	Zfp39	2.813148478	1.75678E-06	down
1173	A_44_P956502	Pik3cd	2.813118124	6.71478E-06	down
1174	A_42_P535085	Ndufv3	2.812844631	0.000101854	down
1175	A_64_P154811	LOC499980	2.811771972	1.00893E-06	down
1176	A_44_P635769	Mks1	2.809655406	0.000604946	down
1177	A_44_P457218	Ddx28	2.809363555	1.55769E-05	down
1178	A_64_P160111	LOC685003	2.803452579	3.19416E-05	down
1179	A_44_P255954	Kctd2	2.800848987	0.002190294	down
1180	A_44_P913369	Slc47a2	2.794837688	0.000532854	down
1181	A_42_P614692	Oplah	2.787688768	0.031928248	down
1182	A_44_P494439	Cnnm2	2.784735879	0.00080099	down
1183	A_44_P497193	Senp8	2.784363626	0.005136583	down
1184	A_64_P058619	Wdr24	2.782706979	7.87363E-06	down
1185	A_64_P049411	LOC679229	2.781455839	0.00076989	down
1186	A_64_P078263	Iglon5	2.781091735	0.027154405	down
1187	A_64_P087109	Ankrd10	2.773047509	3.65407E-05	down
1188	A_64_P088447	Abhd8	2.773029569	0.000101497	down
1189	A_44_P369847	Keap1	2.770774945	0.005726068	down

1190	A_64_P245720	Zinki	2.769511124	0.000289196	down
1191	A_44_P259125	Nkiras1	2.766692935	5.92451E-06	down
1192	A_44_P363647	Ezh2	2.765113436	0.000204539	down
1193	A_64_P004057	Olr220	2.759555689	0.000692689	down
1194	A_44_P357089	Fbxl20	2.759392598	0.003221464	down
1195	A_64_P014774	Mcm5	2.759319152	6.64217E-07	down
1196	A_44_P513853	Nxn	2.75867729	0.001534851	down
1197	A_44_P246381	Dgcr6	2.756744827	0.001086737	down
1198	A_64_P018977	Tex13b	2.756704828	0.008704428	down
1199	A_64_P155216	Kif9	2.756604067	0.000211455	down
1200	A_64_P114378	Rere	2.755275663	5.03641E-05	down
1201	A_44_P274491	Large	2.75362888	0.040258826	down
1202	A_64_P091318	RGD1309823	2.752466618	7.51346E-05	down
1203	A_44_P473234	Zfp688	2.749563871	0.000274499	down
1204	A_64_P138470	Ifi204	2.746510549	2.99055E-05	down
1205	A_64_P021428	Slc16a6	2.736179353	0.000187937	down
1206	A_43_P17761	Etaa1	2.734561673	0.009969202	down
1207	A_43_P21231	Suv39h1	2.734294554	0.000340092	down
1208	A_64_P134124	Xylt2	2.732667634	3.20131E-05	down
1209	A_44_P293008	Ing2	2.729266928	0.00051952	down
1210	A_42_P826191	Pecam1	2.727446693	0.000185111	down
1211	A_64_P043033	Cbr3	2.721264582	2.40037E-05	down
1212	A_43_P23215	Smpdl3b	2.720686009	2.55276E-05	down
1213	A_64_P141737	RGD1566325	2.719916571	0.000791003	down
1214	A_64_P078827	Aer61	2.719493982	0.004763969	down
1215	A_42_P641107	Rpa2	2.716671264	1.84847E-07	down
1216	A_44_P466700	Lpin1	2.716385934	0.000139	down
1217	A_42_P713844	Setd6	2.712981795	0.000364913	down
1218	A_42_P492882	Nckipsd	2.712669588	6.22897E-05	down
1219	A_42_P599116	Phldb3	2.711764886	0.00092627	down
1220	A_64_P030010	Klhdc2	2.709505358	8.29776E-06	down
1221	A_44_P1040187	Mri1	2.708541508	0.000168903	down
1222	A_44_P115886	RGD1309534	2.706552426	8.69327E-06	down
1223	A_64_P032833	Aspa	2.70646738	3.00423E-05	down
1224	A_64_P044752	Trank1	2.702917542	0.010119936	down
1225	A_44_P149563	Zfp646	2.701276025	0.002408357	down
1226	A_64_P000604	Tradd	2.697247357	0.000591956	down
1227	A_42_P496322	Ncoa7	2.696310606	0.001994836	down
1228	A_64_P054719	Ube2v1	2.693555329	0.019563469	down
1229	A_44_P407543	Mblac1	2.687571804	6.59444E-06	down
1230	A_64_P087915	Abtb1	2.680470641	0.000135665	down
1231	A_64_P048136	Brca2	2.677371807	0.00091681	down
1232	A_44_P1048727	Mppe1	2.676645355	0.000115782	down
1233	A_64_P097704	Plcb2	2.676521608	0.00232703	down
1234	A_44_P367653	Mrpl16	2.676094508	5.40979E-06	down
1235	A_44_P173138	Ehbp1l1	2.674957556	0.014276194	down
1236	A_44_P187640	Dnajc30	2.67448368	1.32351E-05	down
1237	A_44_P358160	Ear11	2.673553723	6.81689E-07	down
1238	A_43_P13267	Rab3il1	2.672928167	0.001160935	down
1239	A_42_P656476	Zer1	2.672229654	4.67748E-08	down
1240	A_44_P213214	Npepl1	2.667249715	0.000659786	down

1241	A_64_P113458	LOC691692	2.666294551	0.000366943	down
1242	A_42_P531971	Hist3h2a	2.665691758	1.12337E-06	down
1243	A_44_P326515	Chmp6	2.660658285	0.006568812	down
1244	A_44_P111662	Ly49s3	2.660098682	2.70909E-05	down
1245	A_44_P1024065	Hdac5	2.65696643	0.00021772	down
1246	A_64_P026828	Abhd14a	2.655506327	3.68006E-06	down
1247	A_43_P18690	Pds5a	2.653536389	0.025910243	down
1248	A_42_P592157	Pbx2	2.652944817	0.000237933	down
1249	A_44_P558163	Lipt1	2.651899006	1.70341E-05	down
1250	A_64_P055077	Ccdc61	2.650277023	9.42748E-06	down
1251	A_44_P117234	Mcat	2.650087142	0.000235921	down
1252	A_43_P15141	LOC682988	2.649771581	3.28739E-05	down
1253	A_44_P356596	RGD1304592	2.648099626	0.008014094	down
1254	A_44_P135082	P2rx1	2.645201638	0.045220785	down
1255	A_64_P011474	Gtf3c5	2.643386288	0.000621511	down
1256	A_42_P723173	Id1	2.643298829	0.000498808	down
1257	A_64_P108314	Slc25a40	2.642589254	0.000245893	down
1258	A_43_P15727	Lrp4	2.641707498	0.008868379	down
1259	A_44_P123788	LOC293589	2.64155601	4.17671E-05	down
1260	A_64_P229432	Pparg	2.641128203	9.20987E-06	down
1261	A_44_P1017913	Dars2	2.639726877	5.85009E-06	down
1262	A_42_P601920	Cpt2	2.639632648	1.92603E-05	down
1263	A_64_P035152	Osbpl7	2.63317426	9.38373E-05	down
1264	A_42_P562202	Atpaf1	2.632263593	7.07905E-06	down
1265	A_44_P272210	Ccne2	2.628122458	0.000492824	down
1266	A_43_P16541	Toe1	2.624628623	7.23747E-05	down
1267	A_44_P204207	Smyd4	2.624507039	0.017522979	down
1268	A_64_P143507	Alkbh2	2.624307301	1.50415E-05	down
1269	A_44_P293302	Cep57	2.624007542	1.0333E-06	down
1270	A_44_P382482	Atp6v1c2	2.623620889	0.001316854	down
1271	A_64_P019886	Sfi1	2.621846094	0.000655399	down
1272	A_44_P200846	Sfxn5	2.620854204	0.004392347	down
1273	A_64_P066404	Dok4	2.62066298	0.000413318	down
1274	A_64_P159578	Phactr1	2.620181711	0.000120279	down
1275	A_42_P561329	Anapc1	2.61479915	2.03431E-05	down
1276	A_44_P184484	Orc1	2.613336074	2.20294E-06	down
1277	A_64_P055984	Hadha	2.612110207	0.007547574	down
1278	A_43_P20713	Klhl20	2.609205003	0.000354599	down
1279	A_43_P10931	Gpr68	2.602365918	9.85611E-05	down
1280	A_64_P042899	Exosc2	2.601221825	0.000372285	down
1281	A_44_P165999	Adck3	2.601066288	1.13319E-06	down
1282	A_44_P321686	Fam55c	2.597877493	0.010064999	down
1283	A_44_P477555	Ns5atp9	2.596411108	3.89417E-06	down
1284	A_64_P022155	Slc2a5	2.595728453	6.32254E-05	down
1285	A_44_P241409	Vgll4	2.594943871	0.00021417	down
1286	A_64_P121291	Pop5	2.593595453	6.56291E-05	down
1287	A_64_P071193	Nmral1	2.591760127	6.87941E-05	down
1288	A_64_P000381	LOC500392	2.588862962	0.002976775	down
1289	A_44_P363549	Nsun6	2.586853764	1.37975E-06	down
1290	A_44_P264124	Neurl4	2.586229791	0.000017324	down
1291	A_64_P246030	Pfkfb1	2.586147092	0.000500397	down

1292	A_44_P550454	LOC687516	2.585736922	0.007118602	down
1293	A_43_P20930	Thumpd2	2.584747226	3.14219E-06	down
1294	A_64_P051802	Rab1b	2.583629026	0.000393387	down
1295	A_42_P723540	Dyrk3	2.583577211	7.41993E-06	down
1296	A_44_P435664	Rnf166	2.583494836	4.84398E-05	down
1297	A_42_P608768	Bbs12	2.577929958	1.36241E-05	down
1298	A_64_P133167	Duoxa2	2.576270477	0.000493056	down
1299	A_64_P072504	Tpra1	2.576045305	5.84789E-06	down
1300	A_64_P107841	LOC685069	2.57415751	6.65703E-06	down
1301	A_64_P079523	Ahnak2	2.566608648	0.000017405	down
1302	A_64_P014837	Pcdhga5	2.566024598	0.026568859	down
1303	A_64_P025083	Stx16	2.564340193	3.25415E-06	down
1304	A_64_P162276	Pcyox1	2.564058776	2.50932E-05	down
1305	A_44_P1049865	Crot	2.562978424	2.18247E-05	down
1306	A_44_P369144	Wdfy2	2.56287065	0.000294538	down
1307	A_44_P1011464	Rhobtb1	2.561829568	1.74045E-05	down
1308	A_44_P107184	Rbbp9	2.561121329	1.49941E-05	down
1309	A_64_P077613	Mrpl2	2.557754269	0.000203655	down
1310	A_64_P151764	Fam35a	2.557057318	0.001185285	down
1311	A_44_P276435	Nat1	2.556728556	4.10898E-06	down
1312	A_64_P155721	Taok2	2.55496926	0.000549556	down
1313	A_44_P203305	Olr1637	2.552357942	8.04563E-05	down
1314	A_64_P004702	Akap3	2.551842579	0.000108279	down
1315	A_64_P049828	Dbp	2.551791874	0.000034162	down
1316	A_44_P328512	Zswim2	2.551354849	0.001347148	down
1317	A_42_P701779	Rps6ka5	2.551028824	0.000053626	down
1318	A_44_P403532	RGD1564019	2.545714445	0.00062335	down
1319	A_44_P491393	Nudt7	2.545251703	0.000101908	down
1320	A_64_P065071	LOC690326	2.545215478	0.017258281	down
1321	A_64_P089927	Sirt7	2.543926873	0.011380908	down
1322	A_44_P378631	Decr2	2.542833147	5.19672E-05	down
1323	A_64_P045206	Rmnd1	2.542359181	0.000416618	down
1324	A_44_P1009948	RGD1304587	2.539057148	0.000713091	down
1325	A_43_P20639	Spsb2	2.53804468	0.003881946	down
1326	A_44_P452186	Epm2a	2.532731953	0.002296196	down
1327	A_64_P047097	Ptprn2	2.532505964	0.009476323	down
1328	A_64_P136178	LOC689091	2.531122385	0.016868818	down
1329	A_44_P545314	Vps4a	2.527732506	0.044950347	down
1330	A_64_P139457	LOC499124	2.527680644	0.000672708	down
1331	A_64_P051816	RGD1560978	2.516294591	0.009224113	down
1332	A_64_P335246	Fuk	2.515149809	0.000048272	down
1333	A_42_P475260	Elac1	2.515081993	0.011238692	down
1334	A_44_P548581	Fus	2.51025629	0.022631536	down
1335	A_43_P19047	Golph3l	2.510078818	3.73308E-05	down
1336	A_64_P090799	LOC686151	2.509599303	0.000817022	down
1337	A_64_P011089	Pnpo	2.509089327	0.000159856	down
1338	A_43_P13144	Cdkn2c	2.507621608	2.09403E-05	down
1339	A_64_P131370	Calr4	2.500558228	0.007152481	down
1340	A_64_P133081	LOC498154	2.497591732	0.000951704	down
1341	A_64_P138493	Cerk	2.497303216	3.04293E-06	down
1342	A_43_P17424	Chaf1b	2.497244016	0.000159607	down

1343	A_44_P524314	Dcun1d4	2.495767081	1.49963E-06	down
1344	A_64_P004337	Dtl	2.495664786	0.000500408	down
1345	A_64_P105681	LOC363326	2.495085465	0.00053221	down
1346	A_44_P316490	RGD1311612	2.493239947	6.03958E-05	down
1347	A_64_P115137	Plin3	2.486521835	0.007481656	down
1348	A_43_P16992	Auh	2.483711492	0.002919772	down
1349	A_64_P008216	Ppp1r9b	2.483123069	0.014997162	down
1350	A_64_P065902	Zfp157	2.48191774	0.000249083	down
1351	A_43_P11305	Tmem86a	2.481284222	4.62695E-05	down
1352	A_42_P618538	Lta4h	2.480810149	0.000132243	down
1353	A_44_P546180	Cdc40	2.4804556	0.006077818	down
1354	A_64_P001666	Zc3h6	2.479575808	1.33842E-05	down
1355	A_44_P468193	Mxd3	2.479535132	1.96072E-05	down
1356	A_42_P493008	Nrbp	2.4789607	0.003879306	down
1357	A_64_P051617	RGD1308026	2.478935842	8.11229E-05	down
1358	A_44_P344730	Arhgap29	2.477916661	0.000663248	down
1359	A_44_P379805	Ankrd42	2.477053336	0.000308985	down
1360	A_44_P533786	Aurkb	2.475732143	0.000353747	down
1361	A_64_P044410	LOC498122	2.474958555	0.000144666	down
1362	A_64_P100246	Llgl1	2.47491481	0.00045502	down
1363	A_64_P102255	Yeats2	2.474541664	0.011110538	down
1364	A_43_P17271	RGD1307569	2.472933881	1.18347E-06	down
1365	A_64_P078852	RGD1560151	2.471624478	4.50555E-05	down
1366	A_44_P514987	Tshz1	2.469611889	0.005279228	down
1367	A_43_P20684	Tceanc2	2.467890072	0.003233699	down
1368	A_42_P733209	Dgat2	2.467042096	4.66872E-06	down
1369	A_44_P653701	Mybl2	2.466842773	0.000079338	down
1370	A_64_P152883	RGD1563986	2.465799792	0.010374696	down
1371	A_44_P746666	Zfp667	2.46563549	0.000377524	down
1372	A_44_P380132	P2ry10	2.465101983	0.000136563	down
1373	A_44_P515852	Tpx2	2.46186804	8.54943E-05	down
1374	A_64_P142922	Cabp1	2.459577135	0.003671171	down
1375	A_64_P015079	Nek2	2.458735538	0.005065731	down
1376	A_44_P1031638	Nsrp1	2.458507347	0.005186684	down
1377	A_64_P118907	B3galt6	2.458338987	0.004829647	down
1378	A_64_P070043	Rin3	2.452787812	0.000137013	down
1379	A_64_P044137	Lrrc24	2.450817401	0.011110835	down
1380	A_64_P044505	Pxmp2	2.450218939	0.000902496	down
1381	A_64_P036788	Pxk	2.449077565	0.00337029	down
1382	A_44_P437945	Bbc3	2.449054139	0.000114961	down
1383	A_44_P529859	Ing1	2.445359089	0.001558633	down
1384	A_44_P466169	Rnase17	2.445046044	4.36719E-05	down
1385	A_64_P014632	Gosr1	2.444996444	0.011676955	down
1386	A_44_P309266	Pbk	2.443708891	5.55435E-05	down
1387	A_44_P1011595	Phf7	2.441262334	5.30593E-05	down
1388	A_44_P480954	Abhd15	2.440213312	0.001544052	down
1389	A_44_P425848	Rassf3	2.440061201	0.000098791	down
1390	A_44_P190229	Fam58b	2.439800187	0.003961719	down
1391	A_44_P1021476	Zfp637	2.439370112	7.53126E-06	down
1392	A_42_P794052	LOC691221	2.436681633	0.000603227	down
1393	A_44_P411771	LOC680262	2.436192047	0.000035862	down

1394	A_64_P109814	Hscb	2.435944899	8.67846E-05	down
1395	A_44_P150471	Gpr88	2.435425075	6.99471E-05	down
1396	A_64_P015886	Slc35e3	2.43503476	9.4193E-06	down
1397	A_44_P757980	Eif4ebp2	2.425620189	5.56737E-05	down
1398	A_64_P028345	Ift81	2.421607251	0.000493564	down
1399	A_43_P18844	Mtap	2.421241303	0.000562996	down
1400	A_64_P152888	Nphp4	2.420966025	0.003746517	down
1401	A_44_P876363	Vav2	2.420959984	0.000425131	down
1402	A_64_P112896	Klk1c6	2.41945784	0.044545227	down
1403	A_44_P102661	Spata7	2.417973556	0.000154546	down
1404	A_44_P387308	Adamtsl4	2.414522376	4.34314E-05	down
1405	A_44_P308673	Commd5	2.413017703	0.000376383	down
1406	A_64_P101046	Suv420h2	2.412562527	0.000622852	down
1407	A_64_P140081	Tsc22d4	2.409448391	0.018759192	down
1408	A_64_P142166	Polq	2.40822908	0.015228918	down
1409	A_43_P17160	Ppil2	2.406022888	0.005301794	down
1410	A_64_P081501	Mcm7	2.405768795	1.83292E-05	down
1411	A_64_P105278	Lphn2	2.404816256	0.000331459	down
1412	A_64_P138513	Sfxn1	2.404745414	0.045455277	down
1413	A_44_P469040	Fam118a	2.403232842	0.000529037	down
1414	A_44_P185351	RGD1565752	2.402069566	0.000145139	down
1415	A_42_P634149	Cryba2	2.400739221	0.042204827	down
1416	A_64_P017796	Pex5	2.399091918	0.001675007	down
1417	A_44_P478298	Agap3	2.398264035	0.006349133	down
1418	A_64_P223344	Sgk3	2.395874213	0.001186185	down
1419	A_64_P150156	Caskin1	2.390625979	0.000335786	down
1420	A_42_P515876	Rnpep	2.389704392	0.000198621	down
1421	A_43_P11613	Anp32a	2.389548252	0.0009314	down
1422	A_44_P158216	Pik3r2	2.387483893	1.35631E-06	down
1423	A_64_P098544	Spic	2.385329112	0.001696751	down
1424	A_64_P000001	Rnf187	2.383259422	0.001546539	down
1425	A_43_P12678	Pias3	2.382888368	2.33009E-05	down
1426	A_64_P085467	Tbccd1	2.382492215	0.001274064	down
1427	A_43_P12631	Capn10	2.382340565	0.000504798	down
1428	A_44_P447585	LOC10031087[illegible]	2.38207725	1.16245E-06	down
1429	A_43_P19656	Mgat4b	2.381003817	0.004396555	down
1430	A_64_P011291	Zfp426l2	2.380934392	1.82906E-05	down
1431	A_64_P055669	Pcdha1	2.380464202	0.047419152	down
1432	A_44_P438090	Plcb3	2.376389523	0.010275381	down
1433	A_64_P129063	Hirip3	2.375201375	3.34546E-05	down
1434	A_64_P089145	Bin2	2.374261048	0.003172842	down
1435	A_44_P157652	Aes	2.372321655	0.001292557	down
1436	A_42_P776018	Stard4	2.371707892	2.50862E-07	down
1437	A_64_P081546	Plag1	2.371096972	0.003559416	down
1438	A_64_P077806	Lipt2	2.369023156	7.37146E-06	down
1439	A_43_P16688	Katnb1	2.364289008	0.018038579	down
1440	A_64_P042480	Sgol2	2.361375434	0.000222277	down
1441	A_64_P048384	Wdr6	2.361327968	1.66389E-08	down
1442	A_64_P133355	Ppapdc3	2.361085578	0.000022212	down
1443	A_42_P482623	Dstyk	2.359869476	0.000245504	down
1444	A_44_P170396	Timm44	2.358656453	0.005301224	down

1445	A_44_P473314	RGD1309522	2.358325737	0.000177986	down
1446	A_44_P119177	Parp2	2.357055561	1.38564E-05	down
1447	A_43_P18603	Nek4	2.356432899	4.49203E-06	down
1448	A_43_P10405	Klhdc5	2.354226508	0.001218554	down
1449	A_44_P429066	Ankrd16	2.349168395	1.71238E-06	down
1450	A_44_P536749	Metap1d	2.344124561	0.000263912	down
1451	A_64_P129618	Mdm1	2.343772814	0.000670875	down
1452	A_44_P140416	Ccdc77	2.343069476	0.02148969	down
1453	A_44_P1022237	Rab26	2.342849314	2.14609E-06	down
1454	A_44_P340375	RGD1564209	2.341766829	0.000149295	down
1455	A_42_P766913	Ccdc28b	2.339888309	0.000273753	down
1456	A_44_P286226	Hnrnph3	2.339107285	0.025470088	down
1457	A_64_P235760	Trpc2	2.339053186	1.66737E-05	down
1458	A_64_P146532	Fbxo25	2.338171361	9.89292E-06	down
1459	A_64_P160255	Zfp709l2	2.337277826	1.59967E-05	down
1460	A_44_P944998	LOC690344	2.337144605	0.005044257	down
1461	A_64_P029720	Pex10	2.33557596	0.0012598	down
1462	A_44_P1057272	Ehmt2	2.334271169	0.000446696	down
1463	A_44_P424658	Timmdc1	2.329167941	0.002504308	down
1464	A_44_P445694	Wdr91	2.328677413	0.000202201	down
1465	A_64_P041842	Zfp386	2.327851455	1.71376E-05	down
1466	A_64_P101301	Clec4a3	2.324432718	1.49577E-05	down
1467	A_64_P047701	Ticam2	2.321418931	0.001550495	down
1468	A_44_P274650	Zfp691	2.320451269	0.043429534	down
1469	A_64_P062084	Birc6	2.318704277	0.024351309	down
1470	A_64_P110654	Slc25a26	2.318290191	0.000430251	down
1471	A_44_P470824	Trip13	2.31770101	5.32551E-06	down
1472	A_44_P442780	Kcnab1	2.315542655	0.000888954	down
1473	A_44_P322860	Camkk1	2.312888044	0.010041478	down
1474	A_44_P550697	Ankrd32	2.312452075	1.92231E-05	down
1475	A_64_P076470	Pcdhb17	2.30820293	0.001750707	down
1476	A_44_P178240	Rrs1	2.306103078	0.018650776	down
1477	A_64_P079731	Zfyve21	2.304818534	0.000461992	down
1478	A_64_P074574	Pddc1	2.304616981	0.034285376	down
1479	A_43_P18203	Sf3a3	2.302025104	0.011615501	down
1480	A_64_P088680	LOC680377	2.301455105	0.00341706	down
1481	A_64_P152993	Sipa1l1	2.300215188	0.000280703	down
1482	A_44_P268215	Maz	2.299957921	0.00625324	down
1483	A_44_P477650	Adap2	2.294066162	0.001375761	down
1484	A_44_P149267	Slc38a6	2.288837648	0.000554076	down
1485	A_44_P384677	Rtn4ip1	2.288552096	0.001444269	down
1486	A_44_P1002141	Tsen34	2.28754586	0.00010059	down
1487	A_44_P396320	Ercc6l	2.287309723	0.000399572	down
1488	A_64_P064028	Fgfr2	2.287181464	0.022052094	down
1489	A_44_P520829	Pik3r4	2.285985628	5.41966E-07	down
1490	A_44_P346848	Tmem177	2.285345781	0.000337141	down
1491	A_44_P1015388	Ankrd24	2.284347289	3.07258E-06	down
1492	A_44_P176501	Rrp15	2.283332668	0.015193856	down
1493	A_44_P245795	Ccdc88b	2.283260867	0.004850902	down
1494	A_64_P080316	Nr2f6	2.282514196	6.83608E-05	down
1495	A_64_P050734	Tdrkh	2.28158816	0.011585779	down

1496	A_44_P900001	Arhgef15	2.276344852	0.019069814	down
1497	A_44_P353836	RGD1306962	2.275983712	5.34261E-05	down
1498	A_64_P018412	LOC684439	2.275521525	4.48776E-06	down
1499	A_44_P315886	Magi3	2.273150703	4.06057E-06	down
1500	A_44_P434088	Rnf145	2.271557986	0.000951447	down
1501	A_64_P094541	Pkd1	2.270980209	0.004792053	down
1502	A_64_P117914	Phf14	2.270724533	0.00041895	down
1503	A_44_P506273	Neil3	2.270435155	0.00017526	down
1504	A_44_P479359	Mvk	2.269651827	9.21817E-06	down
1505	A_44_P333209	Rnf113a1	2.268240946	0.010442824	down
1506	A_64_P091008	RGD1306271	2.268123242	0.044631949	down
1507	A_44_P1019654	Ercc5	2.26789524	7.52198E-05	down
1508	A_44_P998128	Brd3	2.26686111	0.01131203	down
1509	A_42_P811308	Phf21a	2.266599351	0.000128664	down
1510	A_44_P115048	Terf2ip	2.262615647	1.35048E-05	down
1511	A_44_P242958	Ttpa	2.26247973	0.000204187	down
1512	A_64_P090408	Dip2a	2.261944033	0.003679451	down
1513	A_44_P443976	St3gal3	2.259559582	0.002361002	down
1514	A_64_P026003	Sec61gl	2.257175612	0.008743375	down
1515	A_64_P042991	Dnaja3	2.256975566	0.00946777	down
1516	A_64_P050660	Tnfaip8l2	2.255246037	5.43151E-07	down
1517	A_42_P591245	Gcdh	2.254679963	0.000106334	down
1518	A_42_P456701	Mtfmt	2.254335386	6.78473E-05	down
1519	A_44_P140275	Alpk1	2.253367728	0.002063908	down
1520	A_64_P064321	Scrn3	2.252790466	0.000683518	down
1521	A_44_P182257	Stx17	2.251594194	0.008924853	down
1522	A_42_P832422	Mllt3	2.250456845	2.21407E-05	down
1523	A_44_P227604	Eme1	2.250303253	0.005235366	down
1524	A_44_P607486	Foxn3	2.249572193	3.56032E-06	down
1525	A_64_P045779	Plcl2	2.248719374	0.030242633	down
1526	A_44_P623426	Rad51ap1	2.248485115	0.000122423	down
1527	A_64_P031417	Clecsf6	2.248247711	2.39535E-05	down
1528	A_42_P565275	Aspm	2.247454123	1.71653E-07	down
1529	A_64_P080144	Btbd6	2.247056292	5.70678E-06	down
1530	A_64_P109457	Kif2c	2.244233311	1.30671E-05	down
1531	A_64_P041476	LOC498368	2.241953828	2.24696E-06	down
1532	A_44_P558652	RGD1565983	2.241886281	0.000970105	down
1533	A_44_P144943	Wdr83	2.241461884	0.00065544	down
1534	A_44_P191941	Pex6	2.241432468	0.007396054	down
1535	A_64_P042455	Bloc1s3	2.240046737	5.43883E-07	down
1536	A_44_P482595	F11r	2.240023188	0.000187272	down
1537	A_44_P105377	Cenpb	2.238729001	0.00071284	down
1538	A_42_P458494	Acsf2	2.238602742	0.00491409	down
1539	A_64_P162589	Slc12a8	2.238317303	0.000075965	down
1540	A_42_P691249	Plekhm1	2.236400646	5.01994E-05	down
1541	A_44_P1021489	Palb2	2.232858302	0.000139688	down
1542	A_43_P19743	Krtcap3	2.226848176	0.019797572	down
1543	A_44_P435016	Nudt2	2.226631783	7.0216E-06	down
1544	A_44_P1013405	Acy3	2.226489796	8.63778E-05	down
1545	A_64_P092728	Uxs1	2.225649893	0.008236089	down
1546	A_64_P009047	Nt5c3l	2.225276333	0.000128464	down

1547	A_42_P605985	Ddit4	2.225205742	0.004667035	down
1548	A_43_P12875	Bbs2	2.225181989	0.000768388	down
1549	A_44_P102652	Ikbkap	2.225172375	4.21598E-05	down
1550	A_64_P164736	LOC10035993(	2.225075773	0.002634582	down
1551	A_64_P159722	Bub1	2.225044824	8.77089E-05	down
1552	A_42_P763553	Opa3	2.222670872	0.009778823	down
1553	A_42_P679092	H6pd	2.221531503	2.09526E-05	down
1554	A_64_P265262	Med20	2.219765742	0.019051309	down
1555	A_64_P047445	Tnfaip8	2.218489923	0.000476187	down
1556	A_44_P713838	Tyw3	2.218092658	0.003233804	down
1557	A_44_P140314	Cdc25c	2.21717411	0.02288434	down
1558	A_64_P154711	Olr907	2.214708105	0.001036587	down
1559	A_64_P031383	Adam15	2.213125094	0.003228225	down
1560	A_64_P147659	Tp53i13	2.212987088	0.003700282	down
1561	A_64_P149819	Fam134a	2.212434381	6.26532E-07	down
1562	A_42_P834903	Bckdk	2.211775567	5.06979E-05	down
1563	A_44_P490117	Mbip	2.21140781	5.55063E-06	down
1564	A_64_P116288	Lztfl1	2.211097025	0.000173388	down
1565	A_64_P067868	B3gnt9	2.210858359	0.018724149	down
1566	A_64_P066271	Mxi1	2.209798259	2.4371E-06	down
1567	A_64_P018582	Pura	2.208877635	0.004700072	down
1568	A_64_P130074	Pot1b	2.207294711	1.19801E-06	down
1569	A_43_P16070	Axl	2.206581448	0.001025485	down
1570	A_64_P022730	RGD1306282	2.203512428	0.004872833	down
1571	A_64_P132497	LOC685634	2.202349091	0.000079861	down
1572	A_44_P132733	Olr339	2.201721005	0.037660028	down
1573	A_43_P15304	Grb2	2.201237533	0.00137388	down
1574	A_64_P112816	Gen1	2.200483011	0.002202017	down
1575	A_44_P853289	RGD1359158	2.200096544	0.000254934	down
1576	A_64_P092906	Rnf8	2.199610228	1.27299E-05	down
1577	A_44_P372181	Dbt	2.198222875	1.56702E-06	down
1578	A_64_P119199	RGD1561270	2.198022417	0.000420273	down
1579	A_64_P362701	RGD1563714	2.197537981	0.001106916	down
1580	A_64_P033939	Mcm4	2.197218128	0.000743432	down
1581	A_44_P458878	Tcf19	2.197205284	1.92917E-07	down
1582	A_64_P023421	Hebp2	2.196126412	2.49379E-05	down
1583	A_64_P123804	Clec4a2	2.194645874	9.05726E-06	down
1584	A_44_P391706	Abcb8	2.192762002	0.004280978	down
1585	A_44_P548726	Zfp414	2.189962147	0.010092967	down
1586	A_44_P328815	Smarcal1	2.189945045	0.000840247	down
1587	A_44_P504274	D2hgdh	2.188597314	0.001147769	down
1588	A_64_P088305	RGD1565129	2.188307026	0.0166545	down
1589	A_64_P123346	Xdh	2.187945294	1.25502E-06	down
1590	A_64_P025481	Ddhd2	2.187394799	0.005427603	down
1591	A_44_P431198	Pycr2	2.187250715	0.00033466	down
1592	A_44_P419265	Zscan12	2.18696384	1.01861E-06	down
1593	A_44_P281086	Siva1	2.186290586	2.79282E-05	down
1594	A_64_P059545	Mlycd	2.185273723	2.61852E-06	down
1595	A_44_P337991	Acot3	2.185250497	0.000161746	down
1596	A_43_P18505	Rnf135	2.183955308	0.000319555	down
1597	A_43_P10136	Amz2	2.183713113	2.98746E-06	down

1598	A_42_P560781	Vamp1	2.183395676	0.002077061	down
1599	A_42_P795269	Hspc159	2.18129516	1.19036E-07	down
1600	A_64_P066841	Batf3	2.181055981	0.002812458	down
1601	A_64_P157126	Tstd2	2.180836278	0.002282309	down
1602	A_64_P152403	E4f1	2.180809371	0.001326945	down
1603	A_64_P111179	Vom1r100	2.179503268	0.005187263	down
1604	A_44_P342966	Rwdd3	2.179229442	0.001752387	down
1605	A_44_P304190	Tifa	2.179033939	3.39619E-05	down
1606	A_64_P111300	LOC678880	2.178571808	1.30178E-05	down
1607	A_43_P21295	Cdc7	2.177444027	0.018032941	down
1608	A_44_P199469	Gsdmd	2.176778331	6.67277E-06	down
1609	A_44_P338068	Ccdc134	2.174723473	0.001648441	down
1610	A_44_P114023	Mis12	2.174267129	0.003142602	down
1611	A_64_P033416	Tbc1d17	2.174169874	0.002530157	down
1612	A_64_P036820	Fam109b	2.173274638	0.000163393	down
1613	A_64_P151415	Fancl	2.170694453	7.70024E-07	down
1614	A_44_P323678	Depdc1b	2.170122225	0.000689499	down
1615	A_42_P663350	Shpk	2.168975114	1.19992E-05	down
1616	A_44_P995316	Sgpp1	2.167304957	3.13094E-05	down
1617	A_42_P771346	Acvrl1	2.166895129	0.000209004	down
1618	A_64_P001036	Acsf3	2.166502798	0.000107677	down
1619	A_44_P852017	Gpr146	2.165425493	0.000126976	down
1620	A_64_P129805	Rfc2	2.163374354	0.000872588	down
1621	A_64_P100933	Pomt2	2.163174875	0.002148576	down
1622	A_44_P947801	Gli4	2.162971666	0.000171283	down
1623	A_64_P104988	Tonsl	2.161961952	0.000452298	down
1624	A_64_P162860	LOC686961	2.16180321	0.006300467	down
1625	A_44_P520190	Exoc2	2.161667705	0.001086018	down
1626	A_44_P349539	Elmo2	2.16153061	0.001961888	down
1627	A_44_P998015	Cdc42ep3	2.160844616	6.33823E-06	down
1628	A_44_P548842	Inpp5k	2.160801181	9.29614E-05	down
1629	A_64_P123266	Dab2ip	2.159644476	3.33779E-05	down
1630	A_44_P332896	Kctd14	2.157003823	0.011234957	down
1631	A_44_P1038655	Ttc25	2.155509469	0.001144612	down
1632	A_64_P050229	LOC686765	2.154654324	0.000157975	down
1633	A_64_P050903	Prelid2	2.153879192	0.005001462	down
1634	A_42_P788040	Galnt4	2.152525855	0.000308245	down
1635	A_64_P129770	Tada2a	2.152043341	0.000245274	down
1636	A_43_P15533	Cebpe	2.151194542	5.44149E-05	down
1637	A_44_P318444	Bco2	2.150761075	0.011041273	down
1638	A_44_P508935	Ptplad2	2.14857544	0.00016971	down
1639	A_64_P150187	Pcbp4	2.146136443	0.01367322	down
1640	A_64_P017786	Specc1	2.145912721	0.004499059	down
1641	A_42_P749435	Ap1s1	2.144176499	0.001952478	down
1642	A_64_P085053	Scly	2.14177184	2.17871E-05	down
1643	A_64_P166385	Kifc1	2.141104188	0.000233672	down
1644	A_64_P078664	Slc20a2	2.140577743	0.001575556	down
1645	A_44_P387344	Atad5	2.140553905	0.01398032	down
1646	A_64_P107439	Camsap1	2.140196952	3.29154E-05	down
1647	A_64_P151448	Fancf	2.139166187	0.000929933	down
1648	A_44_P541692	Hexim2	2.139161838	0.000184538	down

1649	A_44_P389019	RT1-CE10	2.138640861	0.037733017	down
1650	A_44_P530547	Prom2	2.138225782	6.61935E-05	down
1651	A_43_P11329	Zfp574	2.136163391	0.037525713	down
1652	A_64_P032479	Sp4	2.135902413	0.001242509	down
1653	A_43_P10919	Exosc8	2.135440302	0.00175447	down
1654	A_64_P129157	Ttf1	2.134292636	0.005244037	down
1655	A_44_P161797	Fam116b	2.133409531	0.000892164	down
1656	A_44_P395538	Rabep2	2.132669882	4.91655E-05	down
1657	A_42_P704583	LOC690349	2.131855027	5.37774E-05	down
1658	A_44_P336608	RGD1565496	2.131154225	3.70193E-05	down
1659	A_44_P276562	Pm20d1	2.129867034	0.021257889	down
1660	A_43_P15577	Rab11b	2.128913747	0.006198003	down
1661	A_44_P910533	Chaf1a	2.128062467	0.000479905	down
1662	A_44_P237994	Abcg1	2.126370508	0.001456207	down
1663	A_64_P138565	Narf	2.126257021	4.58972E-08	down
1664	A_44_P297428	Ncapg	2.125221677	0.016693602	down
1665	A_64_P122178	Zfp40	2.123304872	0.00348595	down
1666	A_64_P035041	Zfp111	2.122823022	0.001492266	down
1667	A_42_P621628	Fam63a	2.122757397	0.002365304	down
1668	A_64_P070108	Trim39	2.122517821	0.007490784	down
1669	A_44_P107634	Haus4	2.121798027	1.82965E-05	down
1670	A_64_P011849	Fam3a	2.121685961	9.61412E-05	down
1671	A_44_P282433	Akt1	2.121052014	0.002770137	down
1672	A_44_P139730	Palm	2.119797862	2.95745E-06	down
1673	A_64_P007439	Kcnc3	2.119481392	0.000213649	down
1674	A_44_P423662	Sh3bp5	2.119159484	1.42308E-05	down
1675	A_64_P004725	Akap7	2.119106752	0.000269429	down
1676	A_44_P470884	Prc1	2.117812843	0.045681519	down
1677	A_44_P388755	Kif15	2.116378989	3.29145E-05	down
1678	A_64_P113493	Nsf	2.116376984	7.99299E-05	down
1679	A_44_P466614	Lactb2	2.116128153	1.31305E-06	down
1680	A_64_P023250	Ascl2	2.114515448	0.033723658	down
1681	A_42_P499282	Msrb2	2.114309629	4.61254E-05	down
1682	A_64_P152818	Camta2	2.113496271	0.007585756	down
1683	A_44_P337094	Prkag1	2.112763574	0.000654755	down
1684	A_43_P17786	Setmar	2.111432071	1.96343E-05	down
1685	A_64_P022511	Casc5	2.110068255	0.000353901	down
1686	A_64_P146762	LOC686264	2.109300731	0.026772522	down
1687	A_44_P1034155	LOC686234	2.109242054	0.000396243	down
1688	A_64_P029636	Gtpbp10	2.107783955	7.80689E-05	down
1689	A_44_P807091	Cd2bp2	2.107239703	0.000999604	down
1690	A_64_P100225	LOC679038	2.106805893	0.000670444	down
1691	A_64_P010613	Asf1a	2.106725723	0.001091883	down
1692	A_64_P055748	Rhbdf1	2.106553661	0.006222985	down
1693	A_64_P047688	Igbp1	2.105992064	0.002009789	down
1694	A_44_P1033023	Parp1	2.105367379	2.48622E-05	down
1695	A_64_P273080	Pdxk	2.103488552	0.000832456	down
1696	A_64_P138776	Scarb1	2.102461089	0.00755192	down
1697	A_44_P471233	Sash3	2.102096111	0.048831274	down
1698	A_64_P136382	Acvr1	2.101996888	0.000060304	down
1699	A_42_P537971	Pard6b	2.101751205	6.63175E-06	down

1700	A_44_P344928	RGD1563375	2.100608977	0.011987527	down
1701	A_44_P307837	Gfer	2.100501331	0.000299608	down
1702	A_44_P990937	Smarca4	2.100097876	0.040197849	down
1703	A_43_P12346	Mertk	2.100022133	0.000241173	down
1704	A_43_P10088	Rnaseh2a	2.099036858	0.000218659	down
1705	A_44_P538970	Ptpdc1	2.09827611	0.048259774	down
1706	A_43_P15701	Rab13	2.095914696	0.000454561	down
1707	A_64_P061212	Zfp703	2.095874551	0.031991834	down
1708	A_64_P128590	Slc16a14	2.095174201	0.015714982	down
1709	A_64_P143966	Pigh	2.094851484	0.000710227	down
1710	A_44_P543957	Wdr18	2.093312887	0.004428249	down
1711	A_64_P120529	Npat	2.093160105	0.003042399	down
1712	A_64_P115142	Accs	2.092739153	0.000199939	down
1713	A_44_P522369	Pld2	2.092066919	0.000281036	down
1714	A_42_P735730	Dapp1	2.09044246	0.000313814	down
1715	A_44_P360804	Mpg	2.090168813	6.32012E-06	down
1716	A_64_P059032	Susd3	2.087869441	7.83499E-06	down
1717	A_44_P530637	Gmppa	2.086506044	0.010224487	down
1718	A_64_P011869	Fam3d	2.085346084	0.000215659	down
1719	A_64_P044668	LOC691849	2.085331871	5.56381E-05	down
1720	A_64_P075647	Lmnb1	2.084988028	0.006371173	down
1721	A_44_P132039	Yipf2	2.084496573	0.003495951	down
1722	A_64_P088247	Prrt1	2.083614237	3.2937E-06	down
1723	A_43_P20107	Rfx3	2.082447609	0.000309889	down
1724	A_64_P036049	Me2	2.082425957	0.0010494	down
1725	A_43_P21660	Sun1	2.082409406	0.002082568	down
1726	A_64_P016761	Zdhhc9	2.081790898	0.018609532	down
1727	A_64_P088034	Tmem186	2.080715717	0.008229999	down
1728	A_44_P943756	Ccdc82	2.080383883	7.32639E-05	down
1729	A_44_P335734	Pigq	2.080338557	0.000139997	down
1730	A_64_P153576	LOC500684	2.079288627	0.009024479	down
1731	A_64_P070818	Cnpy2	2.079059432	9.61079E-05	down
1732	A_64_P099293	Slc4a5	2.077382819	0.013400088	down
1733	A_43_P22110	Nfatc2ip	2.076941479	0.038338719	down
1734	A_64_P118352	Msl3l2	2.076405719	6.47163E-06	down
1735	A_44_P711020	Znrf2	2.0725305	0.006021552	down
1736	A_42_P555140	Izumo4	2.071778689	0.00018138	down
1737	A_44_P381069	Glul	2.070925517	0.000394588	down
1738	A_64_P102176	Myo18a	2.070578644	0.013210257	down
1739	A_43_P16804	Pole3	2.070407717	0.004307088	down
1740	A_44_P197086	Rpusd4	2.070302145	0.000127052	down
1741	A_64_P246797	Hnrnpa3	2.070114356	0.028304681	down
1742	A_43_P17861	Tfdp2	2.069527664	9.0449E-06	down
1743	A_64_P016241	LOC684993	2.068555023	8.58419E-06	down
1744	A_44_P157078	RGD1305089	2.067635962	0.015075994	down
1745	A_44_P131012	Orc5	2.06756932	3.98959E-05	down
1746	A_44_P1006090	Spg20	2.067413115	0.034923179	down
1747	A_42_P573232	Ube2k	2.066808373	0.000051514	down
1748	A_43_P11268	Cdkn1b	2.065748089	0.000567002	down
1749	A_44_P102061	Zc3h7b	2.065166356	0.001140838	down
1750	A_44_P490159	Fam118b	2.063791797	0.000233633	down

1751	A_44_P1009603	Gtse1	2.062771379	0.000843241	down
1752	A_44_P929695	Cdk19	2.062566546	0.000499033	down
1753	A_44_P762828	LOC497978	2.061927157	0.004446319	down
1754	A_42_P535608	Asf1b	2.061882804	0.000016361	down
1755	A_44_P365947	LOC606294	2.061408606	0.000125284	down
1756	A_42_P810736	Timeless	2.059022602	0.000688797	down
1757	A_44_P548420	Pmvk	2.058687712	3.28856E-05	down
1758	A_64_P135202	Snrnp35	2.056944213	0.004359643	down
1759	A_64_P050705	Arhgef3	2.056442928	0.017734349	down
1760	A_44_P730677	RGD1311517	2.056011499	0.00254996	down
1761	A_64_P006728	Zfp524	2.055960433	0.014188902	down
1762	A_64_P042761	Tcte4	2.055910508	0.00044369	down
1763	A_44_P996124	Tlr4	2.054536556	1.32366E-05	down
1764	A_44_P301805	Ryk	2.054312178	2.64831E-06	down
1765	A_44_P187418	RGD1563825	2.054280709	0.003270575	down
1766	A_42_P715210	Abcg3l3	2.05425954	7.21977E-05	down
1767	A_44_P140514	Cenpa	2.054140837	0.00023205	down
1768	A_43_P23014	Lpxn	2.054044115	0.00047192	down
1769	A_64_P133973	Abcd1	2.053612999	0.00067825	down
1770	A_44_P368032	Polr3a	2.053062812	0.009144989	down
1771	A_43_P12540	Dgkz	2.053006222	0.012529596	down
1772	A_44_P700241	Usp46	2.052717604	3.77581E-05	down
1773	A_64_P063998	Ttc4	2.050996458	0.014519068	down
1774	A_64_P062545	Tmem132a	2.049754934	0.005710602	down
1775	A_44_P504374	Mettl13	2.048269287	0.001073209	down
1776	A_42_P661069	Zbtb5	2.047803094	2.02816E-05	down
1777	A_44_P321419	RGD1561997	2.047714334	0.000265622	down
1778	A_44_P471426	Traf3ip1	2.047226792	0.002835462	down
1779	A_44_P165692	RGD1308874	2.046800371	0.004005769	down
1780	A_42_P843603	Tk1	2.044944469	4.43228E-05	down
1781	A_44_P220575	Irak4	2.044843171	0.012910783	down
1782	A_64_P033049	RGD1564791	2.044767012	0.002824467	down
1783	A_64_P107106	Hmgb2	2.044696903	0.00286174	down
1784	A_42_P821898	Efcab7	2.044401422	1.97885E-05	down
1785	A_64_P087151	RGD1560492	2.041913663	5.74587E-05	down
1786	A_44_P159271	Prr15	2.041824639	0.002167457	down
1787	A_64_P008229	Evi2a	2.039501404	8.61605E-06	down
1788	A_42_P726293	Coq9	2.03941682	7.20367E-05	down
1789	A_44_P105680	Zfp496	2.037759511	0.005210809	down
1790	A_44_P415901	Mrpl15	2.037567283	7.09134E-06	down
1791	A_44_P417658	Tfpt	2.037333226	0.014179621	down
1792	A_44_P403005	Trub1	2.036734412	0.000535425	down
1793	A_43_P15261	Nr3c2	2.036675919	0.000770945	down
1794	A_64_P127267	Dguok	2.036601899	0.0003573	down
1795	A_64_P017821	Rap2b	2.03606549	0.011768806	down
1796	A_64_P009375	Hdgf	2.036059187	0.015581565	down
1797	A_44_P426550	Prss53	2.035763496	0.000575468	down
1798	A_44_P548812	Telo2	2.035258391	0.000265301	down
1799	A_64_P017243	Tbc1d8	2.034358354	0.000522338	down
1800	A_64_P028958	Stard7	2.034120059	0.000878981	down
1801	A_44_P881262	Ppp2r5e	2.033020224	3.64698E-05	down

1802	A_42_P668682	Dusp6	2.032752638	0.000130933	down
1803	A_44_P409518	Gmpr	2.032530263	0.000312864	down
1804	A_44_P541587	Fbxo9	2.031260588	0.000279922	down
1805	A_64_P156438	Zfp346	2.031225953	0.000410668	down
1806	A_44_P297645	Zswim3	2.030966909	0.006780795	down
1807	A_42_P590239	Crebl2	2.029756503	7.74047E-06	down
1808	A_43_P14655	Ecsit	2.027359824	0.00027141	down
1809	A_64_P098892	RGD1563482	2.026064355	0.00095968	down
1810	A_64_P080593	LOC679825	2.025862278	0.00045388	down
1811	A_44_P294687	Cdk5	2.025528335	0.000247382	down
1812	A_44_P321622	Itfg3	2.024453913	0.000348054	down
1813	A_64_P015479	Rfc5	2.023459304	0.000310312	down
1814	A_42_P493824	Dok1	2.023263189	6.79901E-05	down
1815	A_64_P044151	Lrrc14	2.022996607	0.000375118	down
1816	A_44_P544340	Obfc2b	2.021288846	0.001218443	down
1817	A_44_P191055	Pogz	2.020209858	0.000820732	down
1818	A_64_P020706	Hist2h2be	2.020138724	0.009971884	down
1819	A_44_P276227	Usp19	2.019945451	0.005956777	down
1820	A_44_P260134	Srcrb4d	2.019503808	0.02533422	down
1821	A_64_P016518	Ffar2	2.019017945	0.013693361	down
1822	A_44_P649046	Lrrc20	2.018936217	3.14425E-05	down
1823	A_44_P527185	Gtf3c1	2.017469412	0.00427955	down
1824	A_64_P136279	Senp7	2.017406298	6.11187E-05	down
1825	A_42_P605265	Chek2	2.016972014	0.000311094	down
1826	A_64_P097188	Zfp94	2.016824664	3.58292E-06	down
1827	A_44_P200636	Cenpv	2.016059196	2.15183E-05	down
1828	A_64_P004279	RGD1561472	2.016038234	0.000695219	down
1829	A_64_P016896	LOC685233	2.014476068	0.000220665	down
1830	A_44_P189026	B3galnt2	2.014317824	0.000884322	down
1831	A_42_P664274	RGD1311558	2.013817342	0.000532872	down
1832	A_64_P151071	Hmgcs1	2.013415371	3.78836E-07	down
1833	A_44_P945669	Dsn1	2.013179948	0.000260837	down
1834	A_44_P516635	Arl2	2.011754649	0.007580706	down
1835	A_44_P885147	Def8	2.011527786	0.000386769	down
1836	A_44_P210892	Nanp	2.011493905	0.000156459	down
1837	A_44_P995874	LOC10036459[illegible]	2.011424612	0.000183082	down
1838	A_42_P841704	Sh3tc1	2.010168353	9.93455E-05	down
1839	A_64_P096647	Tmprss5	2.009531464	0.040042719	down
1840	A_44_P368249	Spag8	2.008929264	0.003375749	down
1841	A_44_P254973	Trmt11	2.00859129	0.001112911	down
1842	A_44_P449727	Ttc12	2.008424181	0.00872153	down
1843	A_64_P178339	Trip11	2.00811645	0.014993453	down
1844	A_64_P057261	Synj2bp	2.007889209	2.71599E-05	down
1845	A_42_P662683	Slc41a1	2.00776349	4.00286E-06	down
1846	A_64_P065200	MGC112715	2.006601314	6.27113E-05	down
1847	A_64_P327151	Rxrb	2.005864288	0.000529758	down
1848	A_44_P506064	Aldh1l2	2.004949316	1.56443E-06	down
1849	A_43_P17531	Muted	2.003360147	0.000240653	down
1850	A_43_P12665	Mmp24	2.002347819	0.000101992	down
1851	A_44_P374741	Dnmt3a	2.001861273	0.000579465	down
1852	A_42_P603461	Isoc1	2.001474821	5.97428E-06	down

1853	A_64_P128160	Nisch	2.00083158	0.006475721	down
1854	A_64_P091891	Zfp217	2.000646534	0.003911037	down

Supplementary Table S2 Aberrantly expressed genes in TGF-β-stimulated WI-38 cells

No.	Probe Name	Gene Symbol	Absolute Fold Change ([TGF-β treatment] vs [Control])	P-value	Regulation
1	A_24_P62659	TSPAN2	400.1120877	1.44E-08	up
2	A_24_P264943	COMP	276.9847261	1.33E-08	up
3	A_23_P206920	MYH11	188.7076231	0.000000325	up
4	A_33_P3369178	PRG4	88.71321276	0.0000668	up
5	A_23_P154338	EFHD1	61.66401872	0.00000012	up
6	A_23_P155848	DKK2	58.26125762	0.0000101	up
7	A_23_P115261	AGT	53.51421876	1.16E-08	up
8	A_23_P132378	CELSR1	53.26237691	0.0000259	up
9	A_23_P135990	SLCO2A1	52.90122874	0.0000335	up
10	A_24_P414803	PLN	48.36087123	0.002850672	up
11	A_32_P151544	KRT18	44.39129732	0.0000004	up
12	A_23_P137573	LEFTY2	43.93421138	0.000000134	up
13	A_23_P46936	EGR2	38.21076123	0.000000723	up
14	A_24_P140608	HBEGF	37.70198725	0.00000581	up
15	A_33_P3396214	KREMEN2	35.82087121	7.37E-08	up
16	A_23_P396858	FZD8	34.69098624	6.37E-08	up
17	A_33_P3224324	NOX4	34.16238797	0.00000105	up
18	A_23_P1320	MYOZ1	33.59018762	2.39E-08	up
19	A_23_P106389	SEMA7A	30.80971821	0.00000018	up
20	A_23_P34700	TNNT2	30.78328064	0.00000767	up
21	A_24_P304423	IGF1	29.38018761	0.000000259	up
22	A_23_P145761	ARL4A	28.65309812	0.00000139	up
23	A_23_P21706	CTPS	28.25017681	0.000000131	up
24	A_23_P42257	IER3	23.37127692	0.000102452	up
25	A_23_P23346	MLLT11	21.76087912	0.0000382	up
26	A_23_P500773	TAK1	21.65287691	0.0000165	up
27	A_23_P71037	IL6	21.11421081	0.000013	up
28	A_23_P348257	NUAK1	20.88087341	0.00000158	up
29	A_23_P39766	GLS	20.24398762	0.0000398	up
30	A_33_P3249354	CCDC99	19.76156721	0.00000848	up
31	A_23_P201731	TRAF5	19.57087132	0.00000214	up
32	A_23_P325040	TMPO	19.31129725	0.000016	up
33	A_23_P208310	CD3EAP	18.83276971	0.0000072	up
34	A_23_P113825	NACC2	17.46176092	0.0000087	up
35	A_23_P58321	CCNA2	16.77328797	0.00000143	up
36	A_24_P419132	CENPI	16.68421981	0.023995268	up
37	A_24_P56270	DYRK2	11.90198879	0.0000284	up
38	A_23_P81121	EXOSC9	11.02087981	0.0000339	up
39	A_23_P55518	SMAD7	10.96368213	0.001205765	up
40	A_23_P100868	MYO19	10.35098798	0.0000311	up
41	A_23_P252306	ID1	10.34219771	0.00000083	up
42	A_23_P24903	P2RY2	9.972987781	0.000384152	up
43	A_23_P150693	FJX1	9.812987794	0.0000144	up
44	A_23_P205789	GABPB1	8.453287901	0.0000132	up
45	A_33_P3292829	SCYL2	8.124876912	0.000521042	up
46	A_23_P62840	YRDC	6.980986124	0.000104682	up
47	A_33_P3319041	HMGB3	6.951098761	0.0000161	up

48	A_24_P71373	SLC9A1	6.844567814	0.006387341	up
49	A_33_P3374210	MKI67	6.692176992	0.000709776	up
50	A_23_P259413	CDV3	5.518987985	0.0000349	up
51	A_23_P26024	C15orf48	5.508702303	0.00000162	up
52	A_24_P365975	COL8A2	5.490428188	0.000000265	up
53	A_23_P206022	ITGA11	5.452399476	0.000000112	up
54	A_33_P3268783	AMZ1	5.433218248	0.00000107	up
55	A_23_P163567	SMPD3	5.427664075	0.0000206	up
56	A_33_P3252286	CRLF1	5.398971122	0.00000016	up
57	A_32_P98227	LDB3	5.379901156	8.24E-08	up
58	A_23_P406025	PRUNE2	5.360139446	1.39E-08	up
59	A_24_P130363	C18orf1	5.358925278	0.009319466	up
60	A_23_P57277	C21orf7	5.350269882	0.000000764	up
61	A_33_P3418541	ACTG2	5.340493882	0.000000464	up
62	A_33_P3215640	PI16	5.334934284	0.004311506	up
63	A_33_P3373046	ZNF772	5.324709309	7.97E-08	up
64	A_23_P102681	MGC4294	5.322472904	8.49E-08	up
65	A_23_P126836	TNFSF4	5.319958162	0.00000121	up
66	A_33_P3313283	CILP2	5.312280526	0.000006	up
67	A_33_P3336686	CLIC3	5.307815659	0.000000154	up
68	A_24_P329487	FAM84B	5.303997149	0.000000153	up
69	A_23_P151895	CILP	5.298402093	0.000000531	up
70	A_24_P69095	ENC1	5.296832913	2.33E-09	up
71	A_24_P227927	IL21R	5.294277197	0.00000105	up
72	A_24_P6903	ACTBL2	5.277368163	0.00000129	up
73	A_23_P157914	MAMDC2	5.265108761	0.000000102	up
74	A_33_P3345534	KRT14	5.245363182	0.000808355	up
75	A_23_P49499	ST6GALNAC2	5.234530705	0.000000908	up
76	A_33_P3358208	PADI1	5.209029714	8.12E-09	up
77	A_24_P935794	FAM150A	5.202675284	3.81E-08	up
78	A_23_P25030	HSD17B6	5.201161613	4.49E-08	up
79	A_23_P215634	IGFBP3	5.193705341	1.59E-11	up
80	A_23_P167367	PITX2	5.193012387	1.23E-09	up
81	A_23_P53663	PAWR	5.169436405	0.000000607	up
82	A_24_P787897	XYLT1	5.163866368	5.27E-08	up
83	A_23_P65240	COL4A1	5.150010951	0.00000125	up
84	A_23_P203558	HBB	5.138883692	0.036476756	up
85	A_23_P167159	SCRG1	5.138058086	0.00000131	up
86	A_24_P331704	KRT80	5.136936832	9.83E-08	up
87	A_23_P421306	SYT12	5.128238763	0.0000304	up
88	A_24_P268676	BHLHE40	5.107907267	0.00000987	up
89	A_23_P139418	GALNTL4	5.106694425	0.00000293	up
90	A_23_P394395	JPH2	5.102377002	0.000000148	up
91	A_33_P3357818	HTR1D	5.100527055	0.001647136	up
92	A_23_P204286	MGP	5.095359129	0.00000002	up
93	A_23_P151778	CMA1	5.092372073	0.0000923	up
94	A_23_P128084	ITGA7	5.088040661	0.000000524	up
95	A_33_P3347291	INMT	5.084021154	0.00000272	up
96	A_23_P125233	CNN1	5.080700188	0.000000541	up
97	A_33_P3259339	COL11A1	5.073310055	0.000000939	up
98	A_23_P127721	P2RX3	5.059888755	0.0000549	up

99	A_33_P3249818	CEACAM18	5.04236388	0.000821821	up
100	A_23_P38732	CDH2	5.034301776	0.000000082	up
101	A_33_P3277674	FBXL22	5.032630694	0.00000398	up
102	A_24_P104407	SYNM	5.021187736	0.00000194	up
103	A_24_P413126	PMEPA1	5.014463195	0.000000256	up
104	A_33_P3738458	TNS1	5.009687342	0.00000305	up
105	A_33_P3310929	ADAM12	5.00510615	0.00000191	up
106	A_32_P51237	KANK4	5.000000249	0.000046	up
107	A_23_P13740	NAV3	4.985309362	0.0000012	up
108	A_33_P3402654	FLJ43390	4.936928895	5.43E-08	up
109	A_24_P22800	PSG11	4.913823369	0.00000274	up
110	A_24_P158089	SERPINE1	4.899785863	0.000337067	up
111	A_23_P146946	CST6	4.895764516	0.000000118	up
112	A_24_P251969	FGF1	4.892406118	5.95E-08	up
113	A_33_P3335915	SYNE1	4.888774519	0.00000915	up
114	A_24_P371628	ANKH	4.887656847	9.28E-08	up
115	A_23_P43107	TM7SF4	4.882532566	0.000181024	up
116	A_33_P3353791	ITGA1	4.876484147	0.00000048	up
117	A_23_P122924	INHBA	4.85938296	0.00000611	up
118	A_23_P106617	WFDC1	4.85773234	1.53E-10	up
119	A_23_P119095	PPP1R13L	4.856281327	0.00000198	up
120	A_23_P45424	ITGB1BP2	4.841743549	0.0000102	up
121	A_33_P3306624	HCRT	4.840571314	0.0000312	up
122	A_33_P3239347	NKX3-1	4.834552759	0.00000106	up
123	A_33_P3365735	THBS2	4.834473117	0.0000203	up
124	A_33_P3342957	STK17B	4.834179243	6.62E-08	up
125	A_23_P140928	TMC7	4.831147833	0.000163208	up
126	A_33_P3879161	PIK3AP1	4.82467022	0.004597394	up
127	A_23_P372834	AQP1	4.82114649	6.25E-08	up
128	A_33_P3268532	PCK2	4.818059578	8.68E-08	up
129	A_33_P3399534	RNU105B	4.800677427	0.0000923	up
130	A_24_P64233	ALDH1B1	4.797807142	3.73E-08	up
131	A_32_P226205	ZFHX2	4.785340998	0.0000084	up
132	A_32_P192823	PRPS1L1	4.780013082	0.000000218	up
133	A_24_P931443	GPR68	4.779350588	0.00000034	up
134	A_23_P39931	DYSF	4.778619842	0.0000229	up
135	A_23_P40295	C20orf103	4.771404509	0.000000479	up
136	A_33_P3235043	TRIM67	4.767581717	0.0000513	up
137	A_23_P430658	HEYL	4.730644668	0.00000095	up
138	A_33_P3299934	LOC10028925[illegible]	4.73054914	0.000257715	up
139	A_23_P161507	MTL5	4.712770955	0.0000423	up
140	A_33_P3399980	TYRP1	4.708659826	0.0000114	up
141	A_24_P253251	SLC7A1	4.702039961	1.07E-08	up
142	A_32_P62863	SCHIP1	4.692945263	7.81E-08	up
143	A_23_P205370	ASB2	4.645262207	0.000000858	up
144	A_23_P56347	PSG3	4.630503583	0.001082196	up
145	A_33_P3255194	OSBPL6	4.622276619	0.0000164	up
146	A_24_P226970	ZNF365	4.618489218	0.000000913	up
147	A_33_P3415052	NIPAL4	4.612691051	0.000132427	up
148	A_23_P48109	NINJ2	4.601749442	0.00000438	up
149	A_23_P161352	PTPLA	4.596035686	0.000000172	up

150	A_23_P158851	PCDH10	4.592514681	0.000000659	up
151	A_33_P3332547	IQCJ-SCHIP1	4.589786034	1.47E-08	up
152	A_23_P26457	HBA2	4.582705787	0.048873677	up
153	A_24_P914625	BEND4	4.573675994	0.000138083	up
154	A_24_P117029	LDLR	4.570737606	0.0000058	up
155	A_33_P3299510	SCXA	4.566682745	0.000615309	up
156	A_33_P3409159	SLC22A23	4.557413161	0.00000791	up
157	A_23_P2283	TAC3	4.549595135	0.000000195	up
158	A_33_P3421913	CADM1	4.547459009	0.00000392	up
159	A_24_P239606	GADD45B	4.518551398	0.00000112	up
160	A_24_P54390	RASGRP3	4.515920118	0.0000041	up
161	A_24_P926960	MEGF6	4.513954887	0.00000339	up
162	A_33_P3211238	VWCE	4.51045612	0.00000295	up
163	A_33_P3376971	CHAC1	4.507500755	6.73E-08	up
164	A_33_P3323564	LOC646324	4.498114857	0.00000177	up
165	A_23_P138194	NCF2	4.472030931	0.000013	up
166	A_33_P3406899	TRAK1	4.466418283	0.00000126	up
167	A_23_P57784	CLDN1	4.46442147	0.00000105	up
168	A_23_P427136	TSSK1B	4.455746327	0.0000715	up
169	A_23_P209347	ANKRD44	4.447008411	0.000000486	up
170	A_23_P432013	ZPLD1	4.442622836	0.00000235	up
171	A_23_P36888	FAM113B	4.441013633	0.00000336	up
172	A_33_P3392525	ARL4D	4.430231331	0.000000843	up
173	A_23_P374082	ADAM19	4.420981917	8.55E-08	up
174	A_33_P3269166	NALCN	4.42008598	0.00000575	up
175	A_23_P105251	GLI1	4.416602519	1.68E-08	up
176	A_24_P88850	MRAS	4.409411133	1.08E-08	up
177	A_24_P389916	LRRC32	4.408572239	0.00000826	up
178	A_33_P3229156	SLC17A9	4.407512517	0.00000162	up
179	A_33_P3401647	PPP1R14A	4.390044306	0.0000848	up
180	A_24_P246293	FDXACB1	4.383258206	0.000000924	up
181	A_23_P103703	HSPB7	4.383017077	0.000118617	up
182	A_33_P3318272	OR5M11	4.381269924	0.019839364	up
183	A_33_P3246418	MDFI	4.375304222	0.000000233	up
184	A_23_P131676	CXCR7	4.374691451	0.000000854	up
185	A_33_P3243093	RGS5	4.371270126	0.0000022	up
186	A_23_P62999	EXTL1	4.368147784	0.0000179	up
187	A_23_P168610	TSPAN13	4.36318708	0.000000306	up
188	A_24_P275073	ADAMTS14	4.361639703	0.00000425	up
189	A_23_P94552	TMEM2	4.357191938	0.0000056	up
190	A_32_P105549	ANXA8L2	4.307788442	0.000168238	up
191	A_23_P155979	EGF	4.301526225	0.00000819	up
192	A_23_P12554	PKD2L1	4.297266152	0.001498593	up
193	A_33_P3225512	OAS2	4.296071785	0.00044649	up
194	A_33_P3441639	LOC145694	4.28354467	0.00000127	up
195	A_23_P304524	DCLK2	4.282441286	0.00000252	up
196	A_33_P3331746	ST6GAL2	4.279374682	0.000144159	up
197	A_33_P3272231	MFSD2A	4.270021498	0.000000888	up
198	A_23_P107744	S1PR5	4.266520559	0.000000144	up
199	A_33_P3395581	TRIM53P	4.263157039	0.016502484	up
200	A_23_P150053	ACTA2	4.259795679	5.65E-09	up

201	A_33_P3254380	SLC9A7P1	4.249931731	0.00000347	up
202	A_23_P113777	ITGBL1	4.24680196	0.00000029	up
203	A_23_P66881	RGS9	4.243905897	0.00000132	up
204	A_33_P3851023	NRXN3	4.228610464	0.000018	up
205	A_23_P78750	SLC17A7	4.218522121	0.000199891	up
206	A_33_P3398143	ITGB2	4.190933414	0.0000125	up
207	A_32_P14610	PDLIM5	4.188876935	0.000000046	up
208	A_23_P59738	MYL7	4.171986048	0.000000581	up
209	A_24_P34155	RUNX1	4.166022891	0.00000572	up
210	A_23_P333228	4-Mar	4.162034719	1.91E-08	up
211	A_24_P335620	SLC7A5	4.156657852	0.0000104	up
212	A_24_P191312	SLC1A4	4.153030307	0.00000279	up
213	A_23_P126103	CTH	4.145139534	0.0000074	up
214	A_23_P350005	TRIML2	4.143734015	0.000100539	up
215	A_23_P29773	LAMP3	4.138037349	0.007954962	up
216	A_23_P122216	LOX	4.13641136	5.67E-08	up
217	A_23_P374844	GAL	4.135553218	0.000031	up
218	A_23_P127584	NNMT	4.12529417	0.000000272	up
219	A_23_P426305	AOC3	4.113509475	0.000000208	up
220	A_33_P3703501	GALNT10	4.111762115	0.00000788	up
221	A_33_P3312509	ADAMTSL2	4.107837181	0.00000996	up
222	A_24_P71468	QPCT	4.095226745	0.00000171	up
223	A_23_P64567	PPME1	4.093691353	0.0000291	up
224	A_33_P3417650	KLK10	4.093224417	0.00938347	up
225	A_33_P3411260	LOC10028888	4.091669828	0.002355253	up
226	A_33_P3268181	LIMS2	4.087479703	0.008257101	up
227	A_23_P41629	ADAMTS16	4.087070134	0.000831393	up
228	A_23_P87013	TAGLN	4.08332387	0.00000878	up
229	A_23_P210690	TRIB3	4.072810434	0.00000511	up
230	A_24_P413470	TP73	4.067366516	0.005526173	up
231	A_33_P3278590	AKAP14	4.065949603	0.002848303	up
232	A_33_P3335966	TPM1	4.037987188	3.77E-09	up
233	A_23_P256470	NPY	4.031413219	0.037472966	up
234	A_33_P3258279	NTM	4.031219947	2.72E-08	up
235	A_33_P3387861	CENPN	4.029501397	0.000000943	up
236	A_23_P95764	PRPS1	4.022977679	0.00000416	up
237	A_32_P101031	LYPD1	4.022348825	0.000000375	up
238	A_23_P7727	HAPLN1	4.014411197	0.0000181	up
239	A_23_P22350	GRAMD3	4.014312695	0.000000645	up
240	A_23_P141802	SERPINB7	3.993632279	0.0000229	up
241	A_23_P58390	C4orf32	3.991317372	0.000000461	up
242	A_23_P11843	LRRN2	3.990478636	0.0000125	up
243	A_33_P3235880	TBC1D28	3.981568192	0.011212092	up
244	A_33_P3377786	LOC649887	3.969411048	0.00000741	up
245	A_23_P162171	MCAM	3.963222598	0.0000507	up
246	A_23_P139500	BHLHE41	3.955810089	0.000000019	up
247	A_23_P42397	PRSS35	3.945652497	1.26E-08	up
248	A_24_P96961	SPSB1	3.942255733	0.00000257	up
249	A_23_P206733	CES1	3.935963749	0.0000435	up
250	A_33_P3436732	DSCR10	3.926117001	0.0000121	up
251	A_33_P3313145	ITIH3	3.91060124	1.01E-09	up

252	A_23_P431933	CAMKK1	3.89505311	0.000000673	up
253	A_23_P379020	GNRHR2	3.889519213	0.004551633	up
254	A_23_P214300	GSTA2	3.878553584	0.001739253	up
255	A_33_P3270311	HECW2	3.878327764	0.000391994	up
256	A_23_P352799	NPW	3.877318726	0.000521803	up
257	A_23_P110430	MSX1	3.877191517	4.02E-08	up
258	A_23_P120794	SLC7A4	3.876380704	0.00000165	up
259	A_24_P186943	ELN	3.86906223	0.013314967	up
260	A_23_P211445	LIMK2	3.863648011	0.000000773	up
261	A_23_P110184	SC4MOL	3.856483219	0.00003	up
262	A_23_P169909	DGKI	3.85543764	0.00001	up
263	A_32_P190303	LONRF2	3.854837825	0.0000102	up
264	A_33_P3374365	LOC10012970[illegible]	3.852462375	0.000110185	up
265	A_33_P3381666	ABLIM2	3.852364375	0.002255999	up
266	A_23_P369485	MGC45800	3.849916796	0.000174262	up
267	A_24_P151032	MYL4	3.845984904	0.00028083	up
268	A_33_P3262094	OR8G5	3.843482235	0.000995775	up
269	A_23_P112220	INSL4	3.835110997	0.00002	up
270	A_24_P74896	GPR144	3.831476437	0.0000257	up
271	A_24_P347378	ALOX5AP	3.823427011	0.00000686	up
272	A_23_P4899	NTF4	3.82304867	0.000000313	up
273	A_33_P3421821	PLA2G6	3.781703706	0.028709258	up
274	A_33_P3318796	FSTL3	3.780195985	0.000168912	up
275	A_33_P3285565	CLDN3	3.77598404	0.0000201	up
276	A_23_P67151	OLFM2	3.775381844	0.000415063	up
277	A_33_P3363188	FLJ43315	3.772467138	0.0000027	up
278	A_24_P500422	LOC730101	3.762611496	0.00000309	up
279	A_23_P25566	GPR183	3.761100873	0.0000285	up
280	A_24_P74932	PLP2	3.75985146	0.000000687	up
281	A_23_P91850	IL20RB	3.748680578	0.0000136	up
282	A_23_P68740	AIRE	3.746729957	0.000442258	up
283	A_33_P3338494	RTCD1	3.746377123	0.000155035	up
284	A_33_P3343316	SH3BGRL2	3.74239974	0.00000111	up
285	A_33_P3413905	ADM2	3.73879983	0.00000268	up
286	A_23_P151075	ARHGDIB	3.734474292	0.00000305	up
287	A_33_P3418125	GLIPR1	3.726557964	0.00000222	up
288	A_23_P124642	RASGRP1	3.706155635	0.00000436	up
289	A_33_P3219090	INSIG1	3.705877432	0.0000057	up
290	A_33_P3269403	FOXI1	3.704663912	0.047222343	up
291	A_33_P3286953	ADAMTS6	3.702862214	0.0000194	up
292	A_23_P152838	CCL5	3.686177476	0.040324379	up
293	A_23_P8497	GHRHR	3.685283228	0.038980059	up
294	A_33_P3318581	PLOD2	3.684349697	0.0000157	up
295	A_23_P376088	LIME1	3.683183647	0.000161159	up
296	A_33_P3254590	HNRNPKP3	3.679377408	0.041408905	up
297	A_33_P3260634	PLCB4	3.67743328	0.000000391	up
298	A_23_P140760	GPR97	3.669853147	0.0000288	up
299	A_23_P151970	FEM1B	3.668391631	0.000000989	up
300	A_23_P96158	KRT17	3.654541289	0.000329855	up
301	A_33_P3243887	IL11	3.65324523	0.0000207	up
302	A_33_P3229477	MPP7	3.648508547	0.000650703	up

303	A_23_P69030	COL8A1	3.639368743	0.0000252	up
304	A_23_P75430	C11orf75	3.636134755	0.00000583	up
305	A_23_P120316	MTHFD2	3.622104911	8.59E-08	up
306	A_32_P207169	C1orf133	3.618215909	0.00000492	up
307	A_23_P92730	HSPB3	3.615086915	0.000155739	up
308	A_23_P166616	AGTR1	3.605134039	0.0000177	up
309	A_23_P50919	SERPINE2	3.602874336	0.00000297	up
310	A_33_P3606685	LOC283403	3.598654337	0.001398583	up
311	A_23_P379475	DHCR24	3.593607889	0.0000018	up
312	A_33_P3227934	LOC10013043(	3.580168692	0.031895882	up
313	A_32_P157945	DSP	3.571420482	2.18E-08	up
314	A_33_P3227400	COL4A4	3.567697262	0.000732604	up
315	A_24_P178300	PRR21	3.560828616	0.0000639	up
316	A_33_P3372257	HOMER1	3.559831694	7.15E-08	up
317	A_23_P160159	SLC2A5	3.555840634	0.00000479	up
318	A_23_P135061	CORO2A	3.553912102	0.0000144	up
319	A_24_P357169	EPPK1	3.547518399	0.000634139	up
320	A_33_P3300332	LOC10050622:	3.545739711	0.000290436	up
321	A_23_P250212	SGK223	3.545154904	0.00000132	up
322	A_23_P131074	THEG	3.544736366	0.00000632	up
323	A_23_P153676	TLE2	3.538033643	6.89E-08	up
324	A_33_P3413741	OXTR	3.532357431	0.0000365	up
325	A_23_P99515	C13orf33	3.526816057	0.000000821	up
326	A_23_P113701	PDGFA	3.52561474	0.00000248	up
327	A_33_P3708413	MFAP5	3.525236383	0.00000349	up
328	A_23_P401472	CHRM3	3.524202931	0.0000148	up
329	A_33_P3271276	PSG5	3.523024074	0.00000548	up
330	A_23_P371824	TUFT1	3.520267025	0.00000411	up
331	A_23_P389102	MYO1D	3.519227386	3.32E-08	up
332	A_32_P163858	SCD	3.518992242	0.0000649	up
333	A_23_P160466	SLC19A2	3.518023939	0.00000204	up
334	A_24_P5750	KLK2	3.51670666	0.0000111	up
335	A_23_P165201	PRODH2	3.508828657	0.043673482	up
336	A_23_P209978	VSNL1	3.50420538	0.000427563	up
337	A_33_P3246448	KCNE4	3.489851464	0.0000175	up
338	A_23_P401606	EDIL3	3.489815744	0.0000415	up
339	A_23_P153022	KRTAP2-4	3.486075141	0.0000256	up
340	A_23_P81717	FRMD1	3.480946562	0.020879845	up
341	A_33_P3335147	MURC	3.473565575	0.0000187	up
342	A_32_P4626	LOC10050588(	3.468854031	0.00000166	up
343	A_23_P147245	OSBPL10	3.466983971	0.00000702	up
344	A_24_P93855	FOXP4	3.463799525	0.000423907	up
345	A_32_P103291	SMYD3	3.454014964	0.00000285	up
346	A_24_P348989	LILRA1	3.451614396	0.0000162	up
347	A_23_P136355	HHAT	3.449000489	0.0000081	up
348	A_23_P433798	PODNL1	3.448374271	0.000155528	up
349	A_23_P115190	NGF	3.447711365	0.000000113	up
350	A_24_P334130	FN1	3.447576903	0.0000501	up
351	A_32_P108655	AK4	3.435991816	0.0000789	up
352	A_33_P3408305	CERS3	3.433542925	0.0000403	up
353	A_24_P59220	POTEF	3.431039258	0.0000963	up

354	A_23_P121665	SORCS2	3.424835829	0.0000278	up
355	A_23_P114008	TM4SF20	3.421372851	0.0000352	up
356	A_23_P210581	KCNG1	3.417561579	0.00000368	up
357	A_23_P128008	CCDC81	3.413846838	0.0000748	up
358	A_33_P3378835	SLC9A3R1	3.408684091	0.000000737	up
359	A_23_P27795	SPINT2	3.407576392	0.000000016	up
360	A_23_P303155	TMEM87B	3.392699376	0.00000566	up
361	A_23_P254507	HOPX	3.392193968	0.0000002	up
362	A_33_P3349646	PCDH7	3.38552702	0.00000328	up
363	A_23_P204375	LPAR5	3.384574954	0.000041	up
364	A_23_P162589	VDR	3.384192967	0.00000358	up
365	A_23_P115444	TNFSF18	3.382475927	0.0000501	up
366	A_23_P207632	ATP2A3	3.381699188	0.000369919	up
367	A_33_P3391275	LOC284749	3.377290627	0.004280913	up
368	A_23_P318904	SERTAD4	3.37611879	3.48E-08	up
369	A_33_P3812815	PKD1	3.371410735	0.000165912	up
370	A_33_P3256272	KRTAP10-5	3.371178457	0.005677133	up
371	A_23_P31984	ACTL7A	3.368366216	0.0000426	up
372	A_23_P426021	SEL1L3	3.368263332	0.0000105	up
373	A_23_P201386	DDAH1	3.36784638	0.00000181	up
374	A_33_P3418576	SERAC1	3.363266655	0.0000012	up
375	A_33_P3289596	EFR3B	3.363197029	0.000000373	up
376	A_23_P27734	NPAS1	3.361568465	0.000776569	up
377	A_24_P354689	SPOCK1	3.358379348	0.000000262	up
378	A_23_P44724	CSRP2	3.357672066	0.0000002	up
379	A_23_P145694	ASNS	3.357219037	8.08E-09	up
380	A_23_P128728	ARG2	3.354261888	0.0000031	up
381	A_24_P395814	CGB	3.35245926	0.00000365	up
382	A_23_P214821	EDN1	3.352091895	0.00000235	up
383	A_24_P366526	SYNGR2	3.350598454	0.00000167	up
384	A_23_P205428	FOXG1	3.348196655	0.039763887	up
385	A_23_P47704	UCP2	3.34749059	0.00010504	up
386	A_23_P214144	COL10A1	3.344978644	0.005325068	up
387	A_23_P2431	C3AR1	3.343595212	0.000149637	up
388	A_33_P3277110	SLC5A3	3.341452811	0.000000119	up
389	A_23_P35456	SH3PXD2A	3.339316488	8.5E-09	up
390	A_23_P52336	UNC5B	3.338738265	0.0000339	up
391	A_33_P3311770	ZNF789	3.33728871	0.00000168	up
392	A_23_P143559	CLTCL1	3.336248535	0.0000118	up
393	A_33_P3218960	CACNA1H	3.330970322	0.000000492	up
394	A_24_P234415	STAC	3.324548367	0.000113351	up
395	A_33_P3402635	C16orf3	3.322969382	0.000091	up
396	A_33_P3250745	DCAF8L1	3.318894262	0.000124998	up
397	A_24_P298027	AXIN2	3.315902377	0.033985412	up
398	A_33_P3369844	CD24	3.315695603	0.000164371	up
399	A_23_P165624	TNFAIP6	3.310711888	0.000000353	up
400	A_23_P344884	CARNS1	3.307594785	0.0000115	up
401	A_32_P61684	PAG1	3.307346806	9.25E-08	up
402	A_33_P3221960	IL18RAP	3.299268062	0.0000139	up
403	A_23_P36624	TAS2R7	3.298447175	0.000102479	up
404	A_33_P3225507	OR10G2	3.296448022	0.000930533	up

405	A_23_P59950	SLC39A14	3.295186757	0.000000814	up
406	A_33_P3331916	LOC10050708	3.294118985	0.0000518	up
407	A_33_P3369098	MYL10	3.29279516	7.47E-08	up
408	A_24_P349274	OR4X2	3.291990639	0.042384902	up
409	A_23_P157865	TNC	3.289230397	0.00000158	up
410	A_33_P3235761	LOC284023	3.284813587	0.001617374	up
411	A_33_P3409477	UBASH3B	3.283060873	0.0000308	up
412	A_33_P3318530	LOC441204	3.282358457	0.011167721	up
413	A_33_P3308456	HOXB13-AS1	3.280446441	0.000000657	up
414	A_23_P85922	BMP8A	3.279589697	0.001836755	up
415	A_33_P3382565	KIF26B	3.279560145	0.00000601	up
416	A_24_P299318	FAM101B	3.275618482	1.97E-08	up
417	A_23_P55586	CDH20	3.275131802	0.003515318	up
418	A_23_P94795	TEAD4	3.273083331	0.000344154	up
419	A_24_P348806	PLEKHA7	3.27226964	0.0000131	up
420	A_33_P3282634	ALDH1L2	3.27212002	0.0000449	up
421	A_23_P107173	MEOX1	3.272045931	0.000152521	up
422	A_23_P131348	THUMPD2	3.27136711	0.00000203	up
423	A_33_P3331426	FLJ37786	3.262964595	0.0000326	up
424	A_23_P116942	LAG3	3.260991845	0.0000117	up
425	A_23_P131050	ACSBG2	3.259541025	0.000527067	up
426	A_24_P318967	PDXK	3.253246936	0.000000558	up
427	A_23_P256473	SEMA3C	3.252211764	0.0000025	up
428	A_33_P3728698	FLJ45248	3.250550347	0.0000447	up
429	A_23_P73012	C9orf3	3.242526204	0.00000833	up
430	A_23_P384635	DZIP1L	3.242172234	0.00000109	up
431	A_23_P49674	ARHGEF15	3.239270618	0.000531038	up
432	A_33_P3709173	PHTF2	3.238914908	0.000333798	up
433	A_33_P3398331	MMP24	3.237839262	0.000000513	up
434	A_33_P3278220	RABEPK	3.234099354	0.000255561	up
435	A_33_P3260964	LOC731779	3.233866972	0.000000791	up
436	A_23_P90722	PTPRN	3.233005586	0.004246937	up
437	A_33_P3237729	PDLIM4	3.227361087	0.00000629	up
438	A_23_P24444	DHCR7	3.225276992	0.0000076	up
439	A_33_P3404749	FMN1	3.223812118	0.00243644	up
440	A_33_P3386414	DCAF4L2	3.223724225	0.000101686	up
441	A_33_P3255509	FCHO1	3.22276308	0.000265359	up
442	A_24_P126139	RAB9B	3.220172559	0.000028	up
443	A_33_P3340615	PON1	3.219884637	0.0000323	up
444	A_23_P215419	ICA1	3.219147094	0.000267965	up
445	A_24_P298723	TTTY20	3.218823565	0.000152561	up
446	A_24_P827037	LRRC15	3.215230868	0.00000419	up
447	A_23_P320897	DENND1B	3.213813773	0.00000059	up
448	A_33_P3274935	C17orf28	3.207565368	7.96E-08	up
449	A_33_P3373364	CLIC4	3.202283124	0.0000222	up
450	A_24_P99046	STK38L	3.200866928	0.00000286	up
451	A_33_P3296772	C14orf82	3.198056292	0.00000545	up
452	A_33_P3407299	ANP32E	3.192953037	0.00000697	up
453	A_33_P3462960	DNAJC3	3.19217704	0.00000635	up
454	A_32_P156851	RCAN2	3.189881137	0.00000214	up
455	A_23_P259692	PSAT1	3.18957013	0.000000232	up

456	A_24_P104512	EVPL	3.18869181	0.000193707	up
457	A_24_P142495	KRTAP1-3	3.18854778	0.00000164	up
458	A_23_P500433	CARD9	3.188280734	1.04E-08	up
459	A_33_P3218980	ENTPD1	3.184292506	0.000124258	up
460	A_23_P46429	CYR61	3.184235414	0.00000018	up
461	A_24_P237583	WFDC8	3.179893541	0.009603609	up
462	A_33_P3401008	TMEM150B	3.175320022	0.0000134	up
463	A_23_P406385	FBXL16	3.175107122	0.000012	up
464	A_23_P19291	TUBB2A	3.163226071	0.00000186	up
465	A_24_P340941	LOC10030697	3.157773952	0.005724057	up
466	A_33_P3460043	C8orf56	3.157425732	0.0000273	up
467	A_33_P3230990	SCUBE1	3.157174423	0.000000328	up
468	A_33_P3739260	CAP2	3.155211759	0.0000605	up
469	A_24_P95822	NPTN	3.15291476	0.0000669	up
470	A_23_P62890	GBP1	3.151127945	0.000000135	up
471	A_23_P122174	XRCC4	3.150874516	0.000000409	up
472	A_33_P3333627	PHACTR1	3.143452217	0.0000224	up
473	A_23_P116512	PRR5L	3.140185966	0.000000788	up
474	A_23_P107116	RNF112	3.136083922	0.000000695	up
475	A_23_P100074	AVEN	3.133203916	0.00000153	up
476	A_24_P306304	PRAMEF16	3.128337863	0.03013309	up
477	A_33_P3270863	XDH	3.121955557	0.000954071	up
478	A_33_P3705884	LOC148189	3.120836046	0.00000295	up
479	A_23_P258381	SPSB4	3.120834099	0.0000303	up
480	A_33_P3332081	KHDRBS3	3.120798406	0.000000403	up
481	A_33_P3298024	ABCC3	3.119040819	0.00000136	up
482	A_23_P327519	STARD4	3.116915405	0.0000162	up
483	A_23_P354027	KCTD11	3.1144281	0.000137896	up
484	A_32_P225355	CPEB2	3.11111883	0.00000921	up
485	A_33_P3302428	TNRC6C	3.111046374	0.0000507	up
486	A_23_P25994	LGMN	3.104722157	0.0000266	up
487	A_33_P3218178	MCART6	3.102856979	0.000026	up
488	A_33_P3367293	MT1IP	3.102355468	0.019893049	up
489	A_24_P52921	BCAT1	3.100955881	0.000000335	up
490	A_23_P429998	FOSB	3.1005069	0.00000326	up
491	A_23_P26522	AQP8	3.09942709	0.0000237	up
492	A_23_P408376	HSPA12A	3.098188665	0.000000488	up
493	A_33_P3608210	LOC554202	3.096809561	1.98E-08	up
494	A_23_P301053	PAIP2B	3.096230405	0.000220198	up
495	A_32_P471485	RTKN2	3.092723806	0.002016628	up
496	A_23_P255876	DNAI1	3.088247189	0.005902692	up
497	A_33_P3217559	DLK1	3.087924188	4.69E-09	up
498	A_23_P1819	OR8B8	3.087260668	2.22E-08	up
499	A_23_P487	UCK2	3.086422502	0.0000156	up
500	A_33_P3362008	NPPB	3.084598964	0.0000253	up
501	A_33_P3370714	PGC	3.083078094	0.004201376	up
502	A_23_P107963	FUT1	3.080105997	0.000000123	up
503	A_33_P3279629	UCN2	3.078838586	0.00000353	up
504	A_23_P112187	FIBCD1	3.077150274	0.000969737	up
505	A_23_P121011	CSRNP1	3.076803339	0.000317126	up
506	A_33_P3233947	FAM36A	3.072154063	0.0000017	up

507	A_23_P399001	CXXC5	3.070741353	0.0000748	up
508	A_23_P21976	CSPG4	3.070537736	0.0000679	up
509	A_23_P331700	SRRM3	3.064933559	0.000271704	up
510	A_23_P383227	S100A1	3.064495671	0.000292429	up
511	A_33_P3818959	SAMD11	3.061159958	0.00000913	up
512	A_33_P3267814	MICAL3	3.060584358	0.0000079	up
513	A_23_P69738	RASL11B	3.058862661	0.00000286	up
514	A_32_P473302	FLJ35024	3.058586336	0.00000323	up
515	A_33_P3254811	C3orf70	3.054345432	0.0000116	up
516	A_23_P164912	LIN7B	3.052537249	0.00000226	up
517	A_33_P3394380	AKAP5	3.050743599	0.000512062	up
518	A_33_P3233700	LOC10012867	3.046584312	0.0000247	up
519	A_32_P88231	LOC10050569	3.044666409	0.0000514	up
520	A_24_P167642	GCH1	3.043093792	0.0000772	up
521	A_23_P303891	LCE1C	3.040259213	0.000341482	up
522	A_23_P110403	PDLIM3	3.038043092	0.00000246	up
523	A_33_P3350758	RASAL2	3.035837915	0.0000769	up
524	A_23_P19650	VIP	3.035474177	0.0000082	up
525	A_23_P24129	DKK1	3.033800041	5.98E-08	up
526	A_23_P388379	ATRNL1	3.032902599	0.004282539	up
527	A_24_P213643	TSPAN10	3.024184168	0.02127846	up
528	A_23_P146134	DUSP26	3.022751262	0.0000011	up
529	A_23_P416666	GJA10	3.018343849	0.000965769	up
530	A_23_P314101	SUSD2	3.016387271	0.0000535	up
531	A_33_P3369158	KIF3C	3.00780909	0.00000621	up
532	A_33_P3230264	GPC3	3.001309293	0.016143343	up
533	A_33_P3222139	SREBF1	3.000254741	0.000215158	up
534	A_24_P185854	DMD	2.997959028	2.79E-08	up
535	A_24_P131222	ATP13A2	2.996813285	0.00032862	up
536	A_23_P7313	SPP1	2.995717675	0.00000022	up
537	A_33_P3336700	SHROOM3	2.995073346	0.0000097	up
538	A_33_P3307960	AADACL3	2.992209868	0.0000165	up
539	A_33_P3391375	LANCL3	2.991763775	0.0000253	up
540	A_23_P413923	DMRTA1	2.991672048	0.009090189	up
541	A_33_P3395028	LOC152225	2.990030429	0.002580945	up
542	A_23_P254079	STBD1	2.989812131	0.0000336	up
543	A_23_P146284	SQLE	2.984020955	0.0000133	up
544	A_33_P3380383	TIFAB	2.983580012	0.002798356	up
545	A_33_P3267081	ZNF771	2.983548233	0.000000777	up
546	A_23_P344555	NEDD9	2.981349134	0.00000796	up
547	A_23_P35444	INA	2.980704451	0.00000752	up
548	A_23_P121716	ANXA3	2.979367661	0.00000328	up
549	A_33_P3422802	ULBP1	2.97886732	0.00000147	up
550	A_23_P168403	KCNH2	2.978425418	0.00000966	up
551	A_33_P3356857	KIF9	2.973908652	0.000954057	up
552	A_23_P8196	ME1	2.972663784	0.00000314	up
553	A_33_P3406493	GABBR2	2.968987872	0.0000446	up
554	A_33_P3286846	LOC653550	2.967573303	0.031550213	up
555	A_23_P148990	HMCN1	2.966935438	0.000000565	up
556	A_24_P215240	ENKUR	2.966137888	0.006347386	up
557	A_32_P131640	SLITRK4	2.965966974	0.0000814	up

558	A_24_P3005	SCN9A	2.964886752	0.000000947	up
559	A_23_P208009	SEC11C	2.964269121	2.91E-08	up
560	A_23_P54291	DUOX1	2.963786791	0.001923157	up
561	A_23_P98580	FADS2	2.962139668	0.0000575	up
562	A_23_P153930	ACVR2A	2.958022104	0.000000181	up
563	A_32_P210202	E2F7	2.954538913	0.00000473	up
564	A_33_P3266489	OR13H1	2.950698879	0.000000361	up
565	A_33_P3788618	ACER2	2.949540393	0.017367854	up
566	A_33_P3278868	HEATR5A	2.944941209	0.00000741	up
567	A_33_P3273629	CCDC144A	2.944799004	0.000613163	up
568	A_33_P3229370	ID4	2.943328701	0.00000396	up
569	A_33_P3388948	SNX30	2.942903699	0.00000115	up
570	A_33_P3246613	CCDC78	2.940907687	0.000142676	up
571	A_23_P156218	GZMK	2.939934134	0.00000335	up
572	A_33_P3233150	ZSWIM4	2.938206242	0.00000732	up
573	A_23_P114839	FHL3	2.936737806	0.0000318	up
574	A_24_P810290	PPAPDC1A	2.932646135	0.0000111	up
575	A_24_P182620	CELSR2	2.929187748	0.00000173	up
576	A_32_P42574	C1orf198	2.928518213	0.000000516	up
577	A_24_P317907	SORBS1	2.925843155	0.0000202	up
578	A_32_P76720	NT5DC3	2.925678686	0.00000535	up
579	A_23_P211428	SMTN	2.925576683	0.000000725	up
580	A_23_P83818	COL5A1	2.916956085	0.002111124	up
581	A_33_P3412463	C10orf81	2.91683983	0.045665455	up
582	A_24_P129417	BMP1	2.915044687	0.001390594	up
583	A_24_P522631	TMEM201	2.914398182	0.000716972	up
584	A_33_P3228305	ARHGAP26	2.91344754	0.00000145	up
585	A_23_P33664	ELSPBP1	2.912492834	0.000501072	up
586	A_23_P203947	DDX11	2.911088434	0.00000583	up
587	A_33_P3243702	KLHL30	2.90979416	0.0000732	up
588	A_24_P250922	PTGS2	2.905685715	0.00000736	up
589	A_32_P140489	GDF6	2.902591943	0.000000283	up
590	A_23_P133386	RASGRF2	2.902491616	0.00000199	up
591	A_33_P3372288	ZNF81	2.899565443	0.004904303	up
592	A_23_P11032	SLC9A7	2.899414442	0.000611295	up
593	A_33_P3299160	LOC440131	2.899360113	0.002366629	up
594	A_32_P207124	CT47A11	2.898528491	0.000000329	up
595	A_23_P93531	TAAR1	2.89755223	0.000979777	up
596	A_24_P336577	C1orf183	2.895286402	0.0000545	up
597	A_23_P216812	CDKN2B	2.892686746	0.046165091	up
598	A_23_P371266	DNM3	2.890325552	0.00012685	up
599	A_33_P3278313	MSRB3	2.889899189	0.000000412	up
600	A_33_P3423979	PALLD	2.889304455	0.007121089	up
601	A_23_P316381	ACOX3	2.881141467	0.00000103	up
602	A_33_P3420235	ARPC5	2.876679909	0.0000104	up
603	A_23_P347048	SGPP1	2.874912804	0.000123068	up
604	A_33_P3343467	FLJ35390	2.869230092	0.002720473	up
605	A_23_P334751	B3GALNT2	2.868742414	0.00000623	up
606	A_24_P144784	HILS1	2.868403534	0.00000368	up
607	A_33_P3238548	C3orf36	2.867592452	0.000129822	up
608	A_33_P3310784	TM6SF1	2.867046361	0.00000259	up

609	A_33_P3307495	STRA6	2.866542164	0.000399811	up
610	A_23_P200579	CELA3B	2.866444475	0.04472812	up
611	A_24_P100368	DYNLT3	2.866011702	0.000161102	up
612	A_33_P3358601	IFITM10	2.863049605	0.000015	up
613	A_24_P270033	MPZL3	2.86293186	0.00065933	up
614	A_23_P214011	CDH6	2.857279563	0.0000139	up
615	A_23_P35148	TAF13	2.855482085	0.000000151	up
616	A_33_P3313622	MIR17HG	2.855286804	0.033182548	up
617	A_33_P3230528	MPRIP	2.852297898	0.000000196	up
618	A_24_P942773	SLMAP	2.852255062	0.000000314	up
619	A_33_P3214665	MAP2	2.84854823	0.00000865	up
620	A_23_P356616	ABTB2	2.843011629	0.00000895	up
621	A_33_P3670415	NAT8L	2.840295631	0.000161337	up
622	A_24_P134392	HSPA13	2.838051954	0.000000507	up
623	A_23_P161424	PLXDC2	2.835322483	0.00000201	up
624	A_24_P282547	CFHR4	2.82932721	0.000165989	up
625	A_23_P368645	AGPS	2.828615341	0.00000212	up
626	A_23_P59099	OR11A1	2.828171681	0.000259153	up
627	A_23_P133694	SLC29A1	2.827471208	0.00000682	up
628	A_23_P125475	GABRQ	2.826871687	0.027876374	up
629	A_23_P144959	VCAN	2.825248758	0.000000604	up
630	A_33_P3261862	OR2A42	2.823171295	0.000000102	up
631	A_23_P359214	LOC643650	2.822062618	0.000000506	up
632	A_33_P3303542	SSC5D	2.820318964	0.000000558	up
633	A_33_P3344482	FERMT2	2.818534383	0.00000082	up
634	A_32_P159445	IQSEC2	2.81677013	0.000296658	up
635	A_23_P58009	C3orf52	2.816103451	0.000283751	up
636	A_23_P416314	HRASLS5	2.811934521	0.000249546	up
637	A_23_P210358	LIMS1	2.810230303	0.000344009	up
638	A_23_P375524	LCE1D	2.810086746	0.001733699	up
639	A_33_P3239185	SYT7	2.809631647	0.000016	up
640	A_24_P79054	TGFB1	2.809231791	0.002013339	up
641	A_33_P3388651	ABLIM1	2.808725561	0.0000229	up
642	A_33_P3265222	KIAA1324	2.806531658	0.0000884	up
643	A_33_P3604591	SNORA78	2.804793647	0.002796093	up
644	A_23_P47614	PHLDA2	2.803373557	0.0000953	up
645	A_23_P23850	DAB1	2.802901022	0.020293471	up
646	A_23_P150741	C2CD3	2.800758002	0.00000316	up
647	A_23_P371865	CDYL2	2.796560096	0.00000645	up
648	A_33_P3235531	CD1D	2.794801849	0.000454224	up
649	A_23_P111724	RUNDC3B	2.789565319	0.00000805	up
650	A_23_P126649	PGBD5	2.788327459	0.000445277	up
651	A_24_P210569	SCG3	2.786518696	0.030406041	up
652	A_32_P104063	CRNDE	2.785724461	0.00000933	up
653	A_23_P103864	TTC13	2.785377111	0.0000227	up
654	A_33_P3229241	HIST2H2BF	2.785306964	0.0000118	up
655	A_23_P120435	WFDC3	2.783928194	0.00000554	up
656	A_24_P122137	LIF	2.781983121	0.00000819	up
657	A_23_P65618	TGM1	2.781439772	0.0000157	up
658	A_23_P3038	GPX2	2.780842366	0.018681593	up
659	A_23_P22224	EIF4EBP1	2.776566276	0.00000236	up

660	A_24_P9346	SYNGAP1	2.776553253	0.0000173	up
661	A_24_P135753	PTPLB	2.774191668	0.00000119	up
662	A_23_P389897	NGFR	2.769731256	0.00000139	up
663	A_33_P3309799	TRIM10	2.7680129	0.000434733	up
664	A_33_P3239278	OR4D10	2.767822961	0.000071	up
665	A_23_P156880	ENPP1	2.767452906	0.0000144	up
666	A_33_P3340862	TMEM88B	2.766687437	0.000333861	up
667	A_33_P3274332	UBE2J1	2.766096203	0.0000215	up
668	A_23_P88099	MCF2L	2.762382947	0.008373486	up
669	A_23_P65129	SPRYD3	2.761325704	0.00000176	up
670	A_33_P3410599	FAM46A	2.753161677	0.0000198	up
671	A_24_P46130	ACPP	2.751634724	0.0000135	up
672	A_33_P3369760	GLIPR2	2.748956035	0.00000292	up
673	A_33_P3280521	MFAP3L	2.748851873	0.00000326	up
674	A_33_P3293391	LOC642826	2.748238098	0.0000179	up
675	A_23_P7535	HRH2	2.745866718	0.035024734	up
676	A_33_P3219870	PNPLA2	2.745033965	0.00000216	up
677	A_24_P406754	LOXL4	2.744992422	0.000396448	up
678	A_33_P3297978	MYO1E	2.744248257	9.19E-08	up
679	A_33_P3420466	MATN3	2.743378343	0.0000127	up
680	A_23_P401675	MARVELD2	2.743031139	0.0000305	up
681	A_33_P3313421	UHRF1BP1	2.743026703	0.0000152	up
682	A_23_P392470	NR3C2	2.742462322	0.0000135	up
683	A_33_P3250055	MAPK12	2.740807175	0.000651086	up
684	A_23_P419239	ETNK1	2.740461436	0.00000453	up
685	A_33_P3252974	SCRT2	2.738990509	0.02224433	up
686	A_33_P3332382	LOC10012940	2.738116821	0.00000281	up
687	A_23_P206501	CLEC18B	2.734981675	0.0000409	up
688	A_23_P258088	PACSIN1	2.734390014	0.00000041	up
689	A_23_P301572	FAM184B	2.730522289	0.000000721	up
690	A_23_P216549	RUSC2	2.727714719	0.001314298	up
691	A_23_P128174	RAB3IP	2.724402133	0.0000468	up
692	A_23_P424900	C1orf88	2.724245588	0.00000272	up
693	A_23_P43164	SULF1	2.723480681	0.000000614	up
694	A_33_P3254460	DLK2	2.721758381	0.00000334	up
695	A_24_P141707	INHBE	2.719384277	0.00000114	up
696	A_33_P3306654	LOC10013155	2.718638511	0.000883657	up
697	A_23_P150583	SCGB1A1	2.714961362	0.000873174	up
698	A_24_P703830	NANOS3	2.714426337	0.000000219	up
699	A_33_P3363245	NXPH4	2.713579169	0.0000578	up
700	A_33_P3394312	OR2A2	2.709778946	0.0000179	up
701	A_33_P3259775	DOCK5	2.706758485	0.001405155	up
702	A_33_P3280811	TTC16	2.705909021	0.004666535	up
703	A_23_P131596	PRADC1	2.705469105	0.000000361	up
704	A_33_P3227793	CGREF1	2.704547683	0.000751992	up
705	A_33_P3398998	C2orf50	2.704455202	0.000182222	up
706	A_33_P3293474	OPN3	2.702964692	0.006517494	up
707	A_33_P3369834	REEP6	2.702413924	0.000752578	up
708	A_23_P76460	MYF6	2.700694588	0.000253742	up
709	A_32_P313405	LAMA1	2.699034778	0.0000181	up
710	A_23_P92261	ECE2	2.695805666	0.000000813	up

711	A_23_P49338	TNFRSF12A	2.691789883	0.000104402	up
712	A_23_P250694	TSGA13	2.691725949	0.005880978	up
713	A_33_P3327097	MTMR1	2.687279224	0.000069	up
714	A_33_P3294986	LIPE	2.687222288	0.00000375	up
715	A_23_P19592	PGM3	2.687062727	0.00000399	up
716	A_23_P204980	UGGT2	2.684646994	0.000027	up
717	A_23_P259521	WDR41	2.683445957	0.00000642	up
718	A_23_P25674	CKB	2.675583092	0.000000098	up
719	A_33_P3292896	SFXN5	2.674614253	0.005806584	up
720	A_23_P63825	GOT1	2.67115248	0.00000139	up
721	A_23_P31921	ASS1	2.670806889	0.003754564	up
722	A_23_P167129	HHIP	2.668140799	0.004647351	up
723	A_23_P87500	ORMDL2	2.666998229	0.00000357	up
724	A_33_P3419790	C6orf105	2.665198524	0.022352864	up
725	A_24_P363896	COL27A1	2.664806294	0.000499132	up
726	A_33_P3306207	KLRG2	2.662980135	0.000335463	up
727	A_33_P3292402	LRRIQ3	2.661001518	0.000197862	up
728	A_32_P315395	MGC16275	2.660483581	0.000239851	up
729	A_33_P3277579	ZFYVE28	2.660113863	0.003775313	up
730	A_24_P160202	PANX2	2.660097576	0.021110349	up
731	A_33_P3257330	DCBLD1	2.659167214	0.009201801	up
732	A_33_P3245248	TERC	2.658827781	0.002110343	up
733	A_23_P161218	ANKRD1	2.658285085	0.001718041	up
734	A_33_P3221177	ZDHHC20	2.658186018	0.000406174	up
735	A_23_P304450	GATA6	2.657425966	0.000000163	up
736	A_24_P683011	LOC10012798[illegible]	2.65597457	0.000000377	up
737	A_23_P351215	SKIL	2.65422192	0.000019	up
738	A_32_P118372	INTU	2.653588135	0.000000284	up
739	A_33_P3220149	MAML1	2.65283326	0.000142899	up
740	A_23_P315212	NTSR2	2.652317832	0.00000114	up
741	A_24_P914940	MEF2BNB	2.651311351	0.0000467	up
742	A_23_P137532	PLOD1	2.646205249	0.000406563	up
743	A_33_P3277097	LY6G6E	2.642697694	0.000693911	up
744	A_23_P405129	LTBP2	2.637669915	0.00000777	up
745	A_24_P684186	EMB	2.637631217	0.00000399	up
746	A_33_P3805090	FNIP2	2.637031248	0.0000303	up
747	A_33_P3424057	PEG3	2.635925508	0.002786363	up
748	A_33_P3244753	DRP2	2.635247564	0.000000389	up
749	A_23_P252201	EAF2	2.633819235	0.0000413	up
750	A_23_P170857	IL1RAP	2.627662525	0.0000102	up
751	A_23_P31135	ACAT2	2.626482854	0.0000509	up
752	A_33_P3337267	NRARP	2.626186609	0.002500074	up
753	A_23_P250478	PDK3	2.625605926	0.00015882	up
754	A_33_P3415895	NRBF2	2.62265705	0.000000128	up
755	A_23_P64404	FADS3	2.621589258	0.012084396	up
756	A_32_P148345	ANXA2	2.62030485	0.0000298	up
757	A_23_P353005	RNF217	2.619735818	0.00000189	up
758	A_23_P368886	CHSY3	2.619160011	0.00000249	up
759	A_23_P120103	KCNS3	2.618862715	0.00321226	up
760	A_33_P3314276	ASPHD1	2.61754136	0.0000935	up
761	A_23_P436158	TLL2	2.617489954	0.04830929	up

762	A_23_P94319	KBTBD11	2.617175191	0.0000291	up
763	A_24_P79403	PF4	2.615365599	0.000409081	up
764	A_23_P81441	C5orf20	2.612326942	0.000000357	up
765	A_23_P250274	LRRC8A	2.611831816	0.0000276	up
766	A_33_P3347707	NOVA2	2.607838208	0.043952518	up
767	A_33_P3258772	LOC10050681	2.606605645	0.012051632	up
768	A_24_P238118	PP12613	2.603426722	0.000147742	up
769	A_33_P3462422	THEMIS	2.601560335	0.000173406	up
770	A_33_P3799692	LOC338620	2.600228064	0.00000221	up
771	A_33_P3336287	SEC61A2	2.599968841	0.019840937	up
772	A_33_P3408244	SPRNP1	2.59951882	0.000916883	up
773	A_23_P4036	TEX2	2.598693524	0.000114134	up
774	A_23_P217208	SLC35A2	2.597053378	0.0000063	up
775	A_23_P25913	DNAL1	2.5934342	0.0000451	up
776	A_33_P3272539	PLEKHG5	2.59070282	0.0000383	up
777	A_33_P3249489	WDR89	2.588241913	0.00000039	up
778	A_23_P153571	IGFL2	2.587200447	0.000529757	up
779	A_24_P216361	PRAME	2.586365079	0.039257074	up
780	A_23_P307502	C9orf53	2.58546164	0.007553768	up
781	A_23_P160503	GLRX2	2.584815368	0.000000179	up
782	A_23_P167040	PDIA5	2.584590405	0.00000232	up
783	A_33_P3282434	SLC16A1	2.582850428	0.00000652	up
784	A_23_P160460	UAP1	2.582527121	0.000000637	up
785	A_33_P3283669	ATP1A3	2.580368601	0.000000766	up
786	A_23_P122068	C1QTNF3	2.579981999	0.00000989	up
787	A_33_P3422113	ZSCAN12P1	2.577447247	0.002381933	up
788	A_23_P46470	ERRFI1	2.575678038	0.00000108	up
789	A_23_P410110	C10orf25	2.575502428	0.000260157	up
790	A_33_P3275435	LOC10021654	2.573901421	0.00000379	up
791	A_33_P3329344	FASN	2.573433734	2.13E-08	up
792	A_33_P3395758	C14orf28	2.57224786	0.0000107	up
793	A_23_P352389	C17orf46	2.570680534	0.000000115	up
794	A_33_P3399248	UFM1	2.570250548	0.0000568	up
795	A_23_P204158	RNFT2	2.569855368	0.0000792	up
796	A_33_P3387621	RHPN2	2.568482065	0.00000223	up
797	A_32_P78816	PSPH	2.567990146	0.0000368	up
798	A_33_P3367201	MMAB	2.566795454	0.0000224	up
799	A_32_P377880	GDNF	2.565789532	0.0000612	up
800	A_32_P208120	CAMK1D	2.564724746	0.00000561	up
801	A_23_P74330	MGC4473	2.561196659	0.00000239	up
802	A_23_P28598	DLX2	2.56000152	0.000000469	up
803	A_33_P3281572	CMAHP	2.559685449	0.0000437	up
804	A_33_P3242923	LOC283553	2.557596485	0.00000379	up
805	A_23_P115022	TMEM125	2.556387373	0.001970626	up
806	A_23_P30495	HMGCR	2.555794783	0.0000456	up
807	A_23_P42695	GGCT	2.554786384	0.00000507	up
808	A_23_P205031	COL4A2	2.55456073	0.000000823	up
809	A_33_P3426943	LOC151484	2.549874072	0.028885868	up
810	A_32_P121085	DOK3	2.547172149	0.0000363	up
811	A_23_P16944	SDC1	2.546703199	8.45E-08	up
812	A_24_P253003	WNT11	2.542762058	0.000167543	up

813	A_33_P3270203	DIS3L2	2.542716116	0.004669954	up
814	A_33_P3213707	PDCD1LG2	2.541968231	0.00000214	up
815	A_33_P3309491	PTPRU	2.541726678	0.000000238	up
816	A_23_P15174	MT1F	2.536912335	0.0000117	up
817	A_24_P314477	TUBB2B	2.536455998	0.000000489	up
818	A_32_P3476	RPRML	2.534920203	0.002544185	up
819	A_23_P65157	COX17	2.534260215	0.0000254	up
820	A_33_P3372212	PSG10P	2.531489207	0.0000604	up
821	A_33_P3413038	PLXNB3	2.531343279	0.000000861	up
822	A_23_P153628	YIF1B	2.53030413	0.000694607	up
823	A_33_P3211734	SIKE1	2.530240757	0.0000772	up
824	A_32_P452655	LGALS9C	2.52839757	0.0000162	up
825	A_23_P15844	BRIP1	2.527308302	0.0000708	up
826	A_33_P3329839	CDK2AP2	2.524465859	0.00000454	up
827	A_23_P125717	NAP1L3	2.524211272	0.00000501	up
828	A_33_P3280405	OR7E37P	2.524188527	0.00000431	up
829	A_33_P3322859	HES6	2.523581186	0.000228934	up
830	A_24_P187970	PADI2	2.523163916	0.013516634	up
831	A_24_P149036	DPYSL3	2.521857051	0.00000851	up
832	A_23_P205074	SLC46A3	2.519853453	0.00000486	up
833	A_23_P132175	RTN4R	2.51950386	0.0000435	up
834	A_33_P3331188	ARHGAP23	2.519462355	0.000224029	up
835	A_23_P259442	CPE	2.518083575	0.0000224	up
836	A_33_P3412767	SYDE2	2.517971872	0.000123424	up
837	A_33_P3303772	SLC6A3	2.517277037	0.039096045	up
838	A_23_P70688	LY86	2.51723458	0.000000159	up
839	A_33_P3394699	SCN2B	2.514071185	0.000460145	up
840	A_33_P3264926	SAMD4A	2.51032821	9.63E-08	up
841	A_23_P29067	TMPRSS2	2.510158505	0.0000871	up
842	A_33_P3382412	ZNF468	2.509489077	0.0000303	up
843	A_33_P3209581	IQSEC3	2.509414398	0.005197671	up
844	A_23_P1029	MFAP2	2.508665817	0.0000217	up
845	A_24_P925040	CAV2	2.508418097	0.0000013	up
846	A_33_P3283061	LOC10012834	2.507483777	0.000400173	up
847	A_33_P3380751	ST8SIA1	2.503726775	0.000441627	up
848	A_24_P934126	LOC10012803	2.502906966	0.00054733	up
849	A_33_P3422030	FXYD5	2.502688612	0.016376791	up
850	A_33_P3252598	DEFB136	2.502446108	0.000000668	up
851	A_23_P210465	PI3	2.502400721	0.000000299	up
852	A_33_P3364268	LBH	2.501132232	0.0000189	up
853	A_23_P154986	GGT1	2.500648821	0.0000254	up
854	A_24_P152649	LOC644189	2.49835069	0.0000442	up
855	A_24_P154948	GARS	2.497637321	0.0000102	up
856	A_23_P118392	RASD1	2.496371189	0.00000486	up
857	A_33_P3382125	LOC728675	2.494550945	0.0000117	up
858	A_23_P151506	PLEK2	2.494504664	0.00084111	up
859	A_33_P3228722	TBCD	2.493968367	0.006775761	up
860	A_24_P462899	CENPW	2.490313147	0.00000496	up
861	A_33_P3402404	SCN3B	2.487222662	0.000133453	up
862	A_33_P3284939	TMEM189	2.486308127	0.0000266	up
863	A_33_P3398912	SLC2A6	2.484629378	0.00000213	up

864	A_33_P3239134	POM121L4P	2.48220683	9.76E-08	up
865	A_24_P118196	GXYLT2	2.481710792	0.0000129	up
866	A_23_P110791	CSF1R	2.481283534	0.000000238	up
867	A_33_P3362371	RTN3	2.480575153	0.0011174	up
868	A_23_P47247	TCP11L1	2.47915453	0.000000803	up
869	A_32_P25437	SLC12A2	2.478716315	0.0000166	up
870	A_23_P38649	MC2R	2.478016682	0.0000473	up
871	A_23_P137381	ID3	2.477978952	0.000398836	up
872	A_24_P373152	CFL2	2.475253755	0.00000057	up
873	A_23_P418031	IFFO2	2.474832126	0.00000829	up
874	A_23_P167812	RBM24	2.474083743	0.00000068	up
875	A_33_P3267482	KIAA1804	2.47373587	0.000657169	up
876	A_32_P157391	FOLH1B	2.472604794	0.000111381	up
877	A_23_P353744	LARP1B	2.471172007	0.00000433	up
878	A_33_P3280385	COL6A3	2.470831223	0.000028	up
879	A_23_P211561	MEI1	2.470286832	0.0000936	up
880	A_33_P3419419	PIK3C2B	2.469843393	0.000259306	up
881	A_33_P3259821	DOCK9	2.467856772	0.00000217	up
882	A_32_P73796	PPM1E	2.467428592	0.0000178	up
883	A_24_P165450	TTLL7	2.466995983	0.0000551	up
884	A_33_P3217213	PDLIM7	2.464715852	0.000911084	up
885	A_24_P193295	RAB15	2.463957833	0.0000178	up
886	A_24_P943095	SPECC1	2.463686636	0.00000103	up
887	A_33_P3422265	LOC729305	2.462239048	0.001349635	up
888	A_23_P322704	FAM177A1	2.461537071	0.000000984	up
889	A_33_P3330991	LOC10013423	2.458664869	0.000252962	up
890	A_23_P85903	TLR5	2.458171661	0.011339656	up
891	A_33_P3216714	DNAJC6	2.456333073	0.000182972	up
892	A_33_P3331307	CDC42EP3	2.455042727	0.000753023	up
893	A_33_P3274009	LOC10013296	2.454886799	0.0000876	up
894	A_24_P323114	ANXA2P3	2.454812214	0.00000101	up
895	A_33_P3289356	CD58	2.454657378	0.000000886	up
896	A_32_P123514	PABPC4L	2.453067275	0.00000695	up
897	A_23_P138655	CYP26A1	2.452269777	0.000802942	up
898	A_23_P6413	SELM	2.451806856	0.000001	up
899	A_33_P3282181	ARHGAP4	2.446876579	0.002994104	up
900	A_24_P915196	C9orf91	2.446737507	0.0000667	up
901	A_33_P3296352	PPM1B	2.445952974	0.033454856	up
902	A_33_P3266928	LMTK3	2.445869166	0.000589537	up
903	A_23_P415643	ZNF48	2.445835598	0.000000203	up
904	A_33_P3305250	PSMD5	2.445823222	0.001979121	up
905	A_33_P3383205	OK/SW-CL.58	2.445807173	0.000137573	up
906	A_33_P3259522	CDCP2	2.445595269	0.000181759	up
907	A_23_P253586	DOPEY2	2.445074911	0.0000278	up
908	A_24_P161036	ACOT1	2.442270502	0.00000339	up
909	A_33_P3282489	GCNT1	2.4413492	0.00000605	up
910	A_32_P197340	LOC285141	2.440470027	0.0000401	up
911	A_23_P64860	SELPLG	2.440433602	0.000108781	up
912	A_33_P3237096	INPP5F	2.440365657	0.00000806	up
913	A_32_P6015	MNX1	2.440088826	0.000113017	up
914	A_32_P9382	MZT1	2.438596956	0.00000631	up

915	A_23_P161698	MMP3	2.434922746	0.000108401	up
916	A_23_P388168	RAB3B	2.432971634	0.0000835	up
917	A_33_P3359012	DUSP8	2.430326257	0.0000406	up
918	A_33_P3229863	LOC10012871	2.428306114	0.00000631	up
919	A_23_P95790	ITLN1	2.427744223	0.000505056	up
920	A_33_P3241393	SLC4A5	2.425428919	0.000000227	up
921	A_23_P210425	MYL9	2.424001682	0.03606866	up
922	A_33_P3877739	SMCR2	2.423358199	0.000222999	up
923	A_23_P310972	PCDHGB1	2.422726418	0.000311617	up
924	A_23_P75529	PKNOX2	2.422060105	0.00000971	up
925	A_24_P134195	MYADM	2.42171149	0.019160778	up
926	A_24_P206047	SLC25A4	2.420534963	0.0000566	up
927	A_23_P253350	C8orf4	2.420252664	0.0000043	up
928	A_23_P80839	MAP6D1	2.419944287	0.0000364	up
929	A_33_P3671647	LOC441025	2.41958815	0.0000103	up
930	A_33_P3329549	FBRS	2.417447681	0.024487306	up
931	A_33_P3280801	LMO7	2.41690539	0.00000977	up
932	A_24_P42066	GGT3P	2.416549701	0.00000305	up
933	A_33_P3602006	ADAT2	2.414010582	0.001407732	up
934	A_23_P204579	TDG	2.413714153	0.0000202	up
935	A_23_P10858	ANKLE2	2.412585604	0.0000113	up
936	A_33_P3383071	LOC10012843	2.412517097	0.000850681	up
937	A_33_P3308105	GGH	2.412482817	0.0000229	up
938	A_23_P353574	NEK7	2.412163447	0.000537639	up
939	A_23_P387031	COL23A1	2.41111534	0.011362539	up
940	A_23_P315122	EMX1	2.410929225	0.00532742	up
941	A_23_P253389	SLC4A2	2.4103439	0.024755157	up
942	A_33_P3369317	DNAJB5	2.410307868	0.002693827	up
943	A_23_P204801	SLC41A2	2.409721914	0.00000155	up
944	A_23_P160940	ABCA4	2.409494153	0.0000711	up
945	A_33_P3218832	RIMS1	2.409332489	0.0000889	up
946	A_33_P3220570	UBTD1	2.40783578	0.00000092	up
947	A_23_P136405	PDCD1	2.405301092	0.009939543	up
948	A_23_P150198	LOC440040	2.403972068	0.007789069	up
949	A_33_P3489737	NLN	2.403432301	1.37E-08	up
950	A_23_P44836	NT5DC2	2.402030828	0.000314377	up
951	A_23_P75811	SLC3A2	2.401730598	0.001023541	up
952	A_23_P111092	OR2H1	2.401095524	0.000152766	up
953	A_33_P3213119	HAS2-AS1	2.400673047	0.0000248	up
954	A_24_P416961	ARVCF	2.399438496	0.000129022	up
955	A_33_P3388865	LRRC10	2.399089867	0.021181312	up
956	A_23_P119102	VASP	2.398811288	0.0000585	up
957	A_23_P92727	RAI14	2.398690466	0.00000783	up
958	A_33_P3211739	CREB3L2	2.395400132	0.0000157	up
959	A_33_P3222069	SPHK1	2.394695411	0.00000629	up
960	A_23_P218597	NPAS2	2.394142791	0.0000396	up
961	A_24_P200000	STEAP3	2.393318442	0.001508995	up
962	A_23_P413815	VKORC1L1	2.391364145	0.000000285	up
963	A_24_P230282	VCX2	2.390602393	0.016611647	up
964	A_33_P3387691	SCML4	2.389885169	0.024563826	up
965	A_33_P3417459	SCARNA9L	2.389047271	0.000567189	up

966	A_23_P371787	KIAA0247	2.388134624	0.000011	up
967	A_24_P80138	PDCL3	2.38764387	0.000323299	up
968	A_33_P3850216	TRIM55	2.386355533	0.0000997	up
969	A_23_P318284	GPD1L	2.385096933	0.00000084	up
970	A_23_P258944	DNAJB9	2.382487536	0.000000393	up
971	A_23_P404685	LCE1A	2.382394728	0.000368025	up
972	A_33_P3235335	MTMR3	2.38182431	0.01074812	up
973	A_23_P349406	RIMKLA	2.380498412	0.0387936	up
974	A_33_P3230698	KIF1A	2.379978048	0.00000539	up
975	A_33_P3356701	ZDHHC12	2.379810172	0.000000774	up
976	A_24_P937405	PRSS23	2.378730231	0.0000141	up
977	A_33_P3287710	LOC10012829:	2.376793887	0.0000269	up
978	A_24_P272290	C6orf145	2.376588237	0.000225095	up
979	A_23_P30363	P4HA2	2.375545544	0.00000915	up
980	A_33_P3257150	CDC42	2.374923155	0.000107573	up
981	A_23_P40847	CHST2	2.373722028	0.00000736	up
982	A_24_P117942	TOMM20L	2.373661152	0.012568881	up
983	A_23_P82478	PUS7	2.372381237	0.0000876	up
984	A_32_P110872	A2LD1	2.372241905	0.00000619	up
985	A_33_P3249976	JAM2	2.36941893	0.0000093	up
986	A_33_P3214035	ADORA2A	2.368571134	0.019669782	up
987	A_23_P408768	DOT1L	2.367476601	0.0000887	up
988	A_23_P165840	ODC1	2.365593198	0.0000162	up
989	A_33_P3326588	TNFRSF10D	2.365171613	1.52E-08	up
990	A_33_P3393766	C17orf96	2.364230723	0.0000491	up
991	A_33_P3421351	TRAF3IP3	2.363434419	0.028052506	up
992	A_33_P3215953	MPZL1	2.360927381	0.00000703	up
993	A_33_P3283833	FOXS1	2.36070374	0.000121917	up
994	A_33_P3224423	POLM	2.359897993	0.0000337	up
995	A_32_P194264	CHAC2	2.358559778	0.011422589	up
996	A_33_P3321462	OR5B21	2.356586221	0.007836349	up
997	A_33_P3335935	HN1L	2.356146643	0.0000109	up
998	A_23_P106544	C16orf61	2.355246619	0.0000017	up
999	A_23_P166400	RASL10A	2.354705279	0.000000509	up
1000	A_23_P43476	VLDLR	2.350923999	0.000136441	up
1001	A_33_P3243008	KCNU1	2.350326741	0.0000206	up
1002	A_23_P126388	SH3BGRL3	2.350154603	0.00000178	up
1003	A_23_P257111	FBP1	2.349972705	0.008230729	up
1004	A_32_P24376	LOC730755	2.349608951	0.001248052	up
1005	A_24_P332647	SSH1	2.349325044	0.00000691	up
1006	A_23_P58877	GOPC	2.348982014	0.00000423	up
1007	A_23_P252913	BBS7	2.347603341	0.000004	up
1008	A_24_P103886	IDI1	2.347326185	0.000000561	up
1009	A_23_P154065	TUBA4A	2.345824906	0.00000661	up
1010	A_23_P14515	ACOT4	2.344107013	0.0000248	up
1011	A_23_P166336	TMEM191A	2.343683193	0.0000765	up
1012	A_33_P3342628	HES4	2.343470228	0.0000362	up
1013	A_33_P3327608	EML6	2.34304008	0.012948908	up
1014	A_33_P3229953	EEF1A2	2.3428714	0.001284546	up
1015	A_23_P166306	CBS	2.342241227	0.000000186	up
1016	A_33_P3340490	FLJ46906	2.341556203	0.00000369	up

1017	A_33_P3412548	LOC10013442;	2.340798905	0.001954982	up
1018	A_23_P144123	SLC22A13	2.339676608	0.00000709	up
1019	A_24_P76666	CSNK2A1	2.338381413	0.00000708	up
1020	A_33_P3386062	LOC283585	2.335506025	0.0000905	up
1021	A_23_P431388	SPOCD1	2.335089694	0.0000901	up
1022	A_23_P208698	GYS1	2.33367825	0.0000121	up
1023	A_33_P3327270	TMED7-TICAM	2.33216242	0.000360132	up
1024	A_23_P171336	NXF3	2.331904382	0.0000285	up
1025	A_33_P3273125	FAM106CP	2.331851151	0.038492593	up
1026	A_23_P42897	MGAM	2.329245975	0.007497294	up
1027	A_33_P3421243	AFP	2.329017802	0.000357908	up
1028	A_23_P139648	IAPP	2.329006501	0.001082031	up
1029	A_24_P297537	MAMSTR	2.328696299	0.0000214	up
1030	A_32_P148796	UBXN2B	2.328177952	0.000000376	up
1031	A_24_P40094	ATCAY	2.328139921	0.001098225	up
1032	A_23_P71649	MUSK	2.327467464	0.043453429	up
1033	A_33_P3421571	RAPH1	2.327443802	0.000200128	up
1034	A_33_P3390335	RSU1	2.324889262	0.0000887	up
1035	A_33_P3329795	SLC22A20	2.322783461	0.019374232	up
1036	A_23_P363647	DDX26B	2.322728989	0.00000156	up
1037	A_24_P125839	C21orf91	2.317146617	0.0000723	up
1038	A_23_P363769	KRT86	2.316875198	0.0000138	up
1039	A_33_P3237634	TSC22D3	2.316869738	0.000123078	up
1040	A_23_P106145	ERO1L	2.313163111	0.0000056	up
1041	A_32_P87013	IL8	2.312028102	0.000374338	up
1042	A_23_P125829	PGK1	2.311937398	0.000000313	up
1043	A_33_P3288219	FLJ45684	2.311638548	3.75E-08	up
1044	A_23_P31376	LRRN3	2.311011331	0.000415277	up
1045	A_23_P9614	NDUFA4L2	2.31080897	0.0238882	up
1046	A_33_P3554053	NCRNA00106	2.309652383	0.023997577	up
1047	A_33_P3410925	KLF1	2.30863703	0.000233198	up
1048	A_33_P3419180	LOC283547	2.307976445	0.0000906	up
1049	A_24_P237586	ANKRD37	2.306278916	0.0000766	up
1050	A_33_P3420635	OR4D6	2.30461171	0.000572183	up
1051	A_23_P67381	SULT2A1	2.304509955	0.002959954	up
1052	A_33_P3257703	C9orf131	2.303432785	0.021050298	up
1053	A_23_P119593	EPHX3	2.301972023	0.000742642	up
1054	A_23_P319617	CHST7	2.301896552	0.000000277	up
1055	A_32_P140898	FOXN2	2.301854271	0.000351313	up
1056	A_33_P3352687	LOC10013316	2.301509397	0.000148168	up
1057	A_33_P3269844	LRRC26	2.298550453	0.000505263	up
1058	A_23_P162547	MYL2	2.297289489	0.045895983	up
1059	A_24_P62615	CAP1	2.29447502	0.00000296	up
1060	A_33_P3789382	LOC84989	2.293919875	0.000101192	up
1061	A_33_P3229012	LOC652586	2.293899735	0.000608397	up
1062	A_24_P112447	ENTPD7	2.292997899	0.000065	up
1063	A_23_P53345	ARNTL2	2.290734991	0.0000127	up
1064	A_23_P162579	HSPB8	2.290399351	0.000138021	up
1065	A_33_P3331085	SEC24A	2.289665211	0.0000387	up
1066	A_33_P3280400	MGC72080	2.289409124	0.0000858	up
1067	A_33_P3258013	UBE3B	2.288641777	0.000157924	up

1068	A_33_P3251322	MTMR14	2.288436616	0.020414543	up
1069	A_33_P3415087	CLCN5	2.288369995	0.000504381	up
1070	A_32_P58407	KCND3	2.287380963	0.0000049	up
1071	A_23_P407565	CX3CR1	2.284700676	0.00000128	up
1072	A_23_P428260	STEAP2	2.282811918	0.0000323	up
1073	A_33_P3794213	LOC338653	2.281012996	0.00000163	up
1074	A_33_P3702364	SNX24	2.280996501	0.000296609	up
1075	A_33_P3248424	FAM105B	2.279342673	0.0000123	up
1076	A_33_P3417222	CD72	2.279226288	0.000977909	up
1077	A_23_P422831	FAM189A2	2.278466775	0.000769294	up
1078	A_24_P925062	MXRA7	2.27805856	0.0000296	up
1079	A_33_P3447441	LOC202025	2.277772615	0.00000282	up
1080	A_23_P82334	SLC25A13	2.277029528	0.0000027	up
1081	A_24_P55225	RSPH9	2.274808868	0.000935273	up
1082	A_33_P3282566	TMEM104	2.273554624	0.0000388	up
1083	A_23_P51646	PLK3	2.270476965	0.0000496	up
1084	A_33_P3241884	SDC3	2.269260134	0.000686401	up
1085	A_33_P3314515	NCRNA00119	2.269109137	0.000700437	up
1086	A_23_P15369	CD300LB	2.26737031	0.0000966	up
1087	A_23_P372848	P2RX1	2.26693077	0.0000111	up
1088	A_33_P3286196	KRTAP2-2	2.266630669	0.0000226	up
1089	A_23_P357811	MBNL1	2.266288088	0.00000582	up
1090	A_33_P3324086	CCDC90A	2.266243162	0.000000513	up
1091	A_23_P85941	ALX3	2.26606158	0.00000893	up
1092	A_33_P3390950	LOC10012833	2.265515718	0.001147477	up
1093	A_24_P213161	NLRP2	2.264778042	0.0000708	up
1094	A_33_P3344618	TFEB	2.263640205	0.0000151	up
1095	A_23_P401774	ELMOD1	2.263570122	0.000994297	up
1096	A_23_P89343	SNX11	2.263552864	0.000000652	up
1097	A_33_P3316800	AHR	2.263526191	0.00000112	up
1098	A_33_P3341365	RNF216	2.262397347	0.0000198	up
1099	A_33_P3310455	LOC644248	2.261482084	0.000656748	up
1100	A_23_P150852	ESYT1	2.260474328	0.0000343	up
1101	A_23_P409623	PPFIBP2	2.259327274	0.00000571	up
1102	A_33_P3247204	LOC619207	2.259257168	0.0000599	up
1103	A_33_P3339070	LOC10021600	2.258165983	0.0000491	up
1104	A_32_P31771	KIAA1715	2.258069671	0.00000376	up
1105	A_33_P3357753	LOC728190	2.257424181	0.00000653	up
1106	A_24_P67681	LOC10050867	2.256740342	0.0000336	up
1107	A_23_P115726	SLC16A9	2.256432935	0.000973947	up
1108	A_23_P171077	EBP	2.254632818	0.00000271	up
1109	A_23_P36464	C12orf11	2.253050005	0.00000165	up
1110	A_23_P78099	VTN	2.251083648	0.00000733	up
1111	A_23_P60742	MYLPF	2.248221739	0.00000357	up
1112	A_24_P1054	TONSL	2.247562498	0.010670512	up
1113	A_33_P3305620	TIPARP	2.245771118	0.000156051	up
1114	A_23_P368338	BET3L	2.244835403	0.008799836	up
1115	A_33_P3345936	CCDC9	2.244708074	0.001163419	up
1116	A_33_P3325275	NRSN2	2.244058055	0.000854435	up
1117	A_33_P3279162	NBPF6	2.242335265	0.000495727	up
1118	A_23_P383278	PYCRL	2.241336662	0.002877646	up

1119	A_32_P158083	LHFPL3	2.241200936	0.000970766	up
1120	A_23_P141021	LPCAT2	2.240450212	0.000100647	up
1121	A_33_P3376681	GPR139	2.239557954	0.021981794	up
1122	A_33_P3352073	LOC401093	2.238640138	0.00000639	up
1123	A_32_P181103	GGCX	2.21653058	0.00000713	up
1124	A_33_P3415191	ATP8B1	2.214321954	0.00000472	up
1125	A_32_P183970	C15orf62	2.21423626	0.003588436	up
1126	A_32_P170547	CT45A5	2.213744414	0.000264983	up
1127	A_33_P3368560	AHSA2	2.212453295	0.000104362	up
1128	A_23_P117873	CHRM5	2.21235975	0.0000396	up
1129	A_33_P3246248	LOC729879	2.21175717	0.000381133	up
1130	A_33_P3338152	HIF3A	2.211735298	0.00023741	up
1131	A_33_P3365750	EML5	2.210727491	0.001609077	up
1132	A_33_P3231252	NHLH2	2.210712474	0.000000787	up
1133	A_23_P42997	CPSF4	2.210522369	1.93E-08	up
1134	A_33_P3316878	CHPF	2.209310717	0.000000222	up
1135	A_33_P3216664	LOC151174	2.209163455	0.000263041	up
1136	A_23_P215525	OSBPL3	2.208943472	0.00000927	up
1137	A_33_P3405848	TPO	2.208404379	0.00000193	up
1138	A_23_P308042	C6orf195	2.208392592	0.004343382	up
1139	A_33_P3366120	FLNA	2.207288183	0.004108924	up
1140	A_33_P3252196	EZH2	2.204004241	0.0000521	up
1141	A_33_P3218356	TMEM167B	2.202855609	0.0000249	up
1142	A_23_P502312	CD97	2.201982241	0.0000199	up
1143	A_23_P72157	MFSD7	2.200725947	0.0000138	up
1144	A_23_P91636	POM121L9P	2.200025583	0.0000418	up
1145	A_24_P212811	ANKRD34A	2.19890905	0.000224439	up
1146	A_33_P3392580	AK2	2.195750299	0.0000184	up
1147	A_23_P166421	TBC1D10A	2.195000802	0.000018	up
1148	A_23_P254816	TCF15	2.194058866	0.000115813	up
1149	A_23_P108785	ACTR3	2.194036206	0.000000246	up
1150	A_33_P3418803	C4orf44	2.193271889	0.009701783	up
1151	A_33_P3286754	SEC14L2	2.191860832	0.000107427	up
1152	A_33_P3255304	GGT5	2.190064207	0.00000122	up
1153	A_24_P362904	PFKFB4	2.189770993	0.0000354	up
1154	A_32_P32391	OR7E156P	2.189081246	0.0000283	up
1155	A_23_P406616	FLJ36031	2.188705227	0.000172025	up
1156	A_23_P63190	NRAS	2.188548466	0.00000242	up
1157	A_23_P201918	ABCB10	2.186628804	0.000000135	up
1158	A_24_P750305	LOC643837	2.185904441	0.0000555	up
1159	A_23_P7896	DUSP22	2.185663949	0.0000787	up
1160	A_33_P3629247	ANKMY1	2.185192081	0.0000522	up
1161	A_32_P163805	NCRNA00301	2.185176783	0.008652028	up
1162	A_32_P416161	XKRX	2.185009673	0.000395132	up
1163	A_32_P175739	HK2	2.1849289	0.000807275	up
1164	A_33_P3215123	RAP1GDS1	2.184303661	0.0000205	up
1165	A_33_P3341586	SIL1	2.184277721	0.0000567	up
1166	A_33_P3233906	RAMP1	2.183174779	0.000518961	up
1167	A_33_P3361758	C15orf33	2.183068904	0.000342076	up
1168	A_24_P258846	NFATC1	2.182665424	0.000128859	up
1169	A_33_P3306287	METTL16	2.181796684	0.0000101	up

1170	A_24_P557479	XAF1	2.1798079	0.0000216	up
1171	A_23_P127579	PTS	2.178356935	0.000000465	up
1172	A_24_P769672	C12orf73	2.17799705	0.0000165	up
1173	A_23_P122863	GRB10	2.177872757	0.00000583	up
1174	A_33_P3415843	CRAT	2.1767645	0.0000386	up
1175	A_33_P3311503	SMR3B	2.176622675	0.00000773	up
1176	A_33_P3314466	IMMT	2.175826971	0.000000247	up
1177	A_33_P3209351	IARS	2.175451721	0.000025	up
1178	A_33_P3383331	SLC35A3	2.175278821	0.000000403	up
1179	A_33_P3416231	HOXA9	2.175221275	0.000081	up
1180	A_23_P171132	EDA2R	2.174978792	0.0000877	up
1181	A_33_P3365845	NUPL1	2.174831808	0.004882189	up
1182	A_24_P348083	C18orf23	2.173607326	0.000651994	up
1183	A_24_P913227	CDC23	2.172998131	0.000356592	up
1184	A_33_P3248602	DUX4	2.171324475	0.036137308	up
1185	A_23_P29769	WWTR1	2.170969764	0.00000451	up
1186	A_23_P6119	SEC23B	2.169256773	0.000000936	up
1187	A_33_P3672756	LOC284561	2.169245997	0.00197723	up
1188	A_33_P3419785	BNIP3	2.168799221	0.00000099	up
1189	A_23_P93524	SAMD3	2.168795713	0.0000756	up
1190	A_23_P376557	MMP25	2.167324487	0.0000575	up
1191	A_33_P3252394	GADD45G	2.167281172	0.0000713	up
1192	A_33_P3272823	MAG	2.167135108	0.006164622	up
1193	A_23_P101013	TMC6	2.166299927	0.000107772	up
1194	A_33_P3407414	LOC92249	2.166105884	0.000000679	up
1195	A_23_P80773	SRPRB	2.16514213	0.00000321	up
1196	A_23_P134058	GFOD1	2.164641984	0.000001	up
1197	A_33_P3702055	SLC11A1	2.164095151	0.000154336	up
1198	A_33_P3415859	NLRC3	2.164044751	0.000669454	up
1199	A_23_P78762	HSD17B14	2.163749421	0.002828015	up
1200	A_24_P929369	AP4E1	2.163148636	6.45E-08	up
1201	A_24_P326491	MKX	2.162239502	0.000079	up
1202	A_24_P307289	TMEM95	2.16119922	0.004454017	up
1203	A_32_P226646	LOC10012978	2.160800232	0.0000151	up
1204	A_33_P3385762	OR52W1	2.16054498	0.000156348	up
1205	A_23_P48088	CD27	2.16007609	0.031269693	up
1206	A_33_P3271530	PGAP1	2.159928167	0.0000773	up
1207	A_23_P346291	GPR173	2.159742778	5.59E-09	up
1208	A_33_P3364884	PDHB	2.158898125	0.00000192	up
1209	A_33_P3333488	HAGH	2.15843438	9.54E-08	up
1210	A_33_P3253807	CEBPG	2.158211621	0.0000148	up
1211	A_24_P910688	LOC10050904	2.158096186	0.00000889	up
1212	A_23_P117851	CPLX3	2.154454554	0.0000181	up
1213	A_32_P134007	XKR4	2.154049445	0.025870408	up
1214	A_33_P3417502	WNT3A	2.153670089	0.0000151	up
1215	A_33_P3347622	MOBKL1A	2.153315675	0.000245302	up
1216	A_33_P3304878	WDFY4	2.150140448	0.023802787	up
1217	A_33_P3797897	LOC494558	2.149283808	0.024230129	up
1218	A_24_P69053	ODZ3	2.14927487	0.008731335	up
1219	A_33_P3275600	PLA2G4E	2.148552058	0.028786974	up
1220	A_33_P3415678	MKL1	2.14802681	0.00050696	up

1221	A_33_P3287646	HSPB1	2.14788388	0.00000182	up
1222	A_24_P942481	GPR180	2.147809442	0.0000043	up
1223	A_33_P3307133	GRIK2	2.145519579	0.007085435	up
1224	A_33_P3221761	MLL4	2.14541092	0.009837579	up
1225	A_33_P3409580	LOC10012972	2.144912804	0.012760801	up
1226	A_23_P373119	HMGB3P1	2.143548213	0.0000059	up
1227	A_23_P87363	ART1	2.143447379	0.0000611	up
1228	A_23_P18641	SNX25	2.14234476	0.0000252	up
1229	A_24_P204244	ANXA2P1	2.141042351	0.00000247	up
1230	A_33_P3337977	FIG4	2.140393916	0.00028897	up
1231	A_33_P3373298	STAG3L4	2.140371563	0.000129946	up
1232	A_33_P3305433	LOC10013138	2.140020228	0.036546071	up
1233	A_24_P56130	MYL6	2.139307894	0.0000145	up
1234	A_33_P3388067	ZNF735	2.138701542	0.000734715	up
1235	A_24_P253723	C17orf91	2.138610374	0.00000318	up
1236	A_23_P51397	ENAH	2.138336301	0.0000048	up
1237	A_33_P3267844	OR51Q1	2.138145849	0.038465424	up
1238	A_23_P132936	SPCS3	2.137930567	0.000028	up
1239	A_23_P71241	SEC61G	2.136228393	0.000133435	up
1240	A_33_P3464555	LOC283070	2.13601217	0.0000192	up
1241	A_24_P330303	FRMD6	2.135883019	0.000000352	up
1242	A_33_P3419696	FGF2	2.135072411	0.00000065	up
1243	A_33_P3340565	PRSS53	2.134896555	0.00000113	up
1244	A_33_P3436316	ASXL1	2.134796424	0.0000072	up
1245	A_23_P96209	REEP4	2.13472091	0.000252733	up
1246	A_33_P3374778	FLJ36644	2.133082847	0.001269596	up
1247	A_33_P3354137	MAP4	2.131247635	0.005512742	up
1248	A_23_P1102	ACTA1	2.130654496	0.0000341	up
1249	A_33_P3302260	C11orf9	2.130625944	0.00194067	up
1250	A_32_P212897	LOC643406	2.13051666	0.0000263	up
1251	A_23_P368195	LSM11	2.129761972	0.0000284	up
1252	A_23_P23029	BSND	2.128525637	0.044090799	up
1253	A_33_P3357149	FAM35A	2.12817777	1.83E-09	up
1254	A_23_P4572	MYL12A	2.12771856	0.000000768	up
1255	A_33_P3364060	HR	2.127145914	0.0000188	up
1256	A_33_P3303810	LAD1	2.12711446	0.000300974	up
1257	A_24_P337657	SRF	2.126941323	0.001677774	up
1258	A_24_P335305	OAS3	2.125709326	0.012971881	up
1259	A_33_P3260575	CERCAM	2.125703433	0.000000466	up
1260	A_23_P50907	ITGAV	2.125606434	9.66E-08	up
1261	A_33_P3381255	CLEC2A	2.124208383	0.00000565	up
1262	A_33_P3383696	SPEG	2.123137244	0.00000892	up
1263	A_33_P3255499	MCART1	2.12299499	0.00068315	up
1264	A_23_P159956	MID2	2.121948977	0.000201494	up
1265	A_33_P3245163	MYC	2.121751112	0.000907268	up
1266	A_33_P3209960	RASGRP2	2.121221241	0.0000653	up
1267	A_32_P300427	APCDD1L	2.120911369	7.23E-08	up
1268	A_33_P3293009	NKAIN4	2.12059532	0.0002225	up
1269	A_33_P3214129	LOC728061	2.120536526	0.001828875	up
1270	A_23_P160849	FCER1G	2.12020324	0.000161187	up
1271	A_33_P3235132	LOC400968	2.119787136	0.0000416	up

1272	A_23_P416212	HSPB9	2.119759366	0.024482048	up
1273	A_33_P3284311	LOC728660	2.119342859	0.0000624	up
1274	A_33_P3217704	KIAA1539	2.119127953	0.0000578	up
1275	A_23_P203475	PRKCDBP	2.117461395	0.00000117	up
1276	A_23_P43800	BOP1	2.116671423	0.0000129	up
1277	A_23_P49279	C16orf87	2.115617772	0.0000296	up
1278	A_33_P3219641	CEP78	2.115544012	0.00000226	up
1279	A_33_P3284646	FBXL6	2.114271525	0.0000247	up
1280	A_24_P369232	CCDC3	2.114239284	0.0000543	up
1281	A_24_P169234	ZAP70	2.113993587	0.000336979	up
1282	A_24_P175909	MARS	2.11367808	0.00000027	up
1283	A_24_P42389	OTUD6A	2.112832014	0.015147514	up
1284	A_23_P302060	IFNE	2.112418576	0.0000786	up
1285	A_33_P3402010	LOC10050814	2.112202762	0.0000167	up
1286	A_33_P3410206	C16orf7	2.112088031	0.000514973	up
1287	A_23_P15146	IL32	2.111311821	0.00000352	up
1288	A_32_P15512	C1orf194	2.110437834	0.0000274	up
1289	A_33_P3297020	PSORS1C3	2.110350163	0.02253017	up
1290	A_33_P3249046	CLDN2	2.110284241	0.0000171	up
1291	A_32_P59678	C7orf46	2.110222807	0.000913858	up
1292	A_33_P3378284	COX6B2	2.110109939	0.006634606	up
1293	A_33_P3266132	GRHL3	2.109439192	0.003190708	up
1294	A_33_P3386467	RPL23AP7	2.109342936	0.000013	up
1295	A_33_P3370364	PRLHR	2.108306572	0.000873385	up
1296	A_23_P7099	AGA	2.10818952	0.0000273	up
1297	A_23_P120863	GAL3ST1	2.107481792	0.0000803	up
1298	A_32_P81768	TMEM167A	2.106798981	5.95E-08	up
1299	A_24_P188377	CD55	2.106724019	0.00000359	up
1300	A_33_P3210379	SCGB3A1	2.105980386	0.000250266	up
1301	A_23_P356330	PPPDE2	2.105574127	0.0000181	up
1302	A_33_P3415440	MAP3K2	2.105240907	0.0000216	up
1303	A_23_P346006	CCPG1	2.104218956	0.000000353	up
1304	A_23_P257231	AGXT2L1	2.104091971	0.032370375	up
1305	A_23_P128372	FKBP4	2.103698081	0.0000466	up
1306	A_33_P3362153	LOC388564	2.102566358	0.002638741	up
1307	A_23_P28246	SLC23A3	2.102332849	0.000684406	up
1308	A_23_P47579	NLRP14	2.102210785	0.000954818	up
1309	A_33_P3363425	FRMD3	2.102001939	0.014183922	up
1310	A_23_P13364	NUCB2	2.101520845	0.00000274	up
1311	A_24_P286569	LANCL2	2.101262886	0.000431319	up
1312	A_24_P359091	FAM95B1	2.099108394	0.000072	up
1313	A_33_P3249259	TGM6	2.098570601	0.000110016	up
1314	A_33_P3342260	GCK	2.097862322	0.012743353	up
1315	A_23_P105028	ATL3	2.097160096	0.000116374	up
1316	A_33_P3225066	DNAJC12	2.09693833	0.00000448	up
1317	A_24_P150874	GNA13	2.096762805	0.0000042	up
1318	A_33_P3373185	LOC399744	2.096549365	0.001920813	up
1319	A_23_P500130	KANK1	2.095430493	0.00000694	up
1320	A_23_P382506	OR1F2P	2.095319142	0.0000127	up
1321	A_23_P30315	TRIM7	2.093989682	0.0000484	up
1322	A_33_P3390207	TMEM155	2.092972903	0.000039	up

1323	A_32_P154053	ATG9B	2.092322154	0.000557215	up
1324	A_24_P379750	MXD1	2.091982089	0.000341033	up
1325	A_33_P3210622	ASB13	2.091761307	0.0000453	up
1326	A_33_P3312676	MYT1	2.091214428	0.0000167	up
1327	A_23_P158725	SLC16A3	2.091033488	0.0000166	up
1328	A_33_P3313401	CYCS	2.089908964	0.00000533	up
1329	A_23_P131846	SNAI1	2.089510488	0.0000496	up
1330	A_33_P3238280	ESYT3	2.087758733	0.000873682	up
1331	A_32_P126229	FLJ23152	2.08726431	0.000423845	up
1332	A_33_P3219010	PPA2	2.086193821	0.0000188	up
1333	A_33_P3386686	LOC10013287	2.08607255	0.0000105	up
1334	A_23_P403081	C5orf34	2.084732339	0.0000795	up
1335	A_33_P3247534	LOC389834	2.084602532	0.000264421	up
1336	A_33_P3235546	RIMS3	2.084489348	0.000612194	up
1337	A_33_P3632937	LOC10013126	2.084212676	0.00000947	up
1338	A_23_P132226	TPST2	2.08263743	0.000223938	up
1339	A_23_P31453	STEAP1	2.082059888	0.000265402	up
1340	A_33_P3641714	C19orf66	2.082019624	0.002614233	up
1341	A_24_P80776	LOC221710	2.080656538	0.000125707	up
1342	A_33_P3236591	RLTPR	2.079647002	0.001380903	up
1343	A_33_P3413305	SH3BP5L	2.07949392	0.0000208	up
1344	A_23_P92202	GMPPB	2.079416518	0.002174882	up
1345	A_23_P430792	C17orf66	2.079284976	0.000912879	up
1346	A_23_P121480	CD200	2.078238363	0.000649007	up
1347	A_33_P3396200	LRCH4	2.075392204	0.000150883	up
1348	A_23_P325562	SLC1A7	2.07515102	0.002151403	up
1349	A_23_P74943	KCNH1	2.074144874	0.0000335	up
1350	A_23_P386942	DIRAS1	2.073572274	0.0000152	up
1351	A_32_P137966	DEPDC4	2.073167381	0.000432902	up
1352	A_23_P210330	HSPC159	2.072445026	0.0000328	up
1353	A_33_P3271121	SH3BP4	2.071023131	0.0000237	up
1354	A_23_P26223	ASL	2.070784369	0.000106393	up
1355	A_23_P257131	PEX13	2.070726477	0.000016	up
1356	A_23_P142631	FKBP1B	2.069687711	1.47E-08	up
1357	A_23_P54736	GNG13	2.069187909	0.0000352	up
1358	A_24_P879740	MAP1B	2.067528667	0.0000296	up
1359	A_23_P112162	DGAT1	2.066788603	0.0000106	up
1360	A_24_P655268	LOC729082	2.066495086	0.0000378	up
1361	A_23_P254271	TUBB6	2.065792478	0.00000443	up
1362	A_23_P350698	ANKFN1	2.065774579	0.0000119	up
1363	A_33_P3872301	SNAR-C3	2.065269615	0.00000372	up
1364	A_32_P116206	RELL1	2.064133908	0.00014045	up
1365	A_33_P3354646	PNLIPRP1	2.063945678	0.041605099	up
1366	A_23_P94494	TLE4	2.062475526	0.00000769	up
1367	A_33_P3370449	GOT1L1	2.062250138	0.00030565	up
1368	A_33_P3367106	C1orf152	2.062059555	0.000775545	up
1369	A_23_P40354	MAPRE1	2.06190448	0.00000938	up
1370	A_32_P148710	CFL1	2.061573884	0.009868488	up
1371	A_33_P3233125	PSD	2.061457426	0.00038596	up
1372	A_23_P67661	COX7A1	2.060897519	0.00000674	up
1373	A_23_P428729	ZMYM6	2.05929279	0.000110871	up

1374	A_33_P3215948	MPZL2	2.058482048	0.017875664	up
1375	A_33_P3351259	LOC10013056	2.057630834	0.001089469	up
1376	A_33_P3329419	DNM1	2.056896783	0.016484535	up
1377	A_23_P500464	COL2A1	2.056241906	0.001630176	up
1378	A_23_P43317	PRDM12	2.056208887	0.000363175	up
1379	A_23_P303210	IKBIP	2.056086794	0.0000352	up
1380	A_23_P55251	ITGA3	2.055667029	0.007511495	up
1381	A_33_P3259253	CD28	2.053712596	0.041588132	up
1382	A_33_P3360565	ZNF474	2.053444564	0.00000583	up
1383	A_32_P34920	FOXD1	2.052119863	0.00000716	up
1384	A_32_P167239	AFAP1L1	2.050808241	0.0000481	up
1385	A_23_P102890	MRPS6	2.050329863	0.00000272	up
1386	A_23_P110212	ACSL1	2.049641133	0.0000503	up
1387	A_23_P64019	MTMR2	2.048362425	0.00000273	up
1388	A_23_P137814	ATP6V0B	2.048200573	0.0000219	up
1389	A_33_P3589819	LOC10050763	2.047441172	0.000151157	up
1390	A_33_P3365134	PPP1R13B	2.046923048	0.00000606	up
1391	A_24_P289383	CHD7	2.046074486	0.001490989	up
1392	A_23_P10785	VTI1A	2.045527595	0.00000814	up
1393	A_24_P322709	SNTA1	2.045433263	0.017356242	up
1394	A_33_P3273552	KRT83	2.045318473	0.008784722	up
1395	A_23_P357374	PTH2	2.045111262	0.00000271	up
1396	A_23_P166269	FAM3B	2.044538174	0.000377956	up
1397	A_33_P3283122	WWC2	2.044408744	0.0000845	up
1398	A_33_P3469673	LOC284576	2.0437059	0.03819097	up
1399	A_33_P3474250	LOC283270	2.042853386	0.000354123	up
1400	A_24_P15043	KLHL18	2.041845208	0.0000115	up
1401	A_33_P3538688	HIST2H4A	2.041511225	0.000185668	up
1402	A_23_P54846	HERPUD1	2.041087692	0.00000494	up
1403	A_23_P207537	DUSP14	2.040738886	0.00000198	up
1404	A_23_P141770	NAPG	2.039246251	0.00000199	up
1405	A_23_P206359	CDH1	2.039238477	0.00000264	up
1406	A_33_P3379922	PROC	2.038682532	0.00017601	up
1407	A_23_P62907	ATF6	2.038166154	0.0000373	up
1408	A_23_P130488	ERCC2	2.038150896	0.000751984	up
1409	A_23_P62115	TIMP1	2.036163389	7.11E-08	up
1410	A_24_P402438	TGFB2	2.035481346	0.0000191	up
1411	A_23_P393727	NRP2	2.033913983	0.000315894	up
1412	A_24_P252497	TRIB1	2.03328155	0.00010543	up
1413	A_24_P93206	TMEM179	2.03304667	0.011548754	up
1414	A_33_P3526458	LOC338579	2.032861791	0.001679891	up
1415	A_23_P334798	LRRC2	2.032351583	0.0000108	up
1416	A_33_P3275835	TOR2A	2.032260018	0.0000776	up
1417	A_24_P192994	FADS1	2.031737709	0.00000299	up
1418	A_33_P3270317	SLC18A3	2.031442693	0.046245605	up
1419	A_24_P911288	HPX-2	2.031326294	0.000300827	up
1420	A_33_P3350643	LMX1B	2.030842044	0.000355042	up
1421	A_23_P60227	CCIN	2.0305161	0.00000329	up
1422	A_23_P36562	ITGA5	2.030202076	0.004884296	up
1423	A_32_P84009	CMTM4	2.02976682	0.0000923	up
1424	A_33_P3270451	TXNDC5	2.029611314	0.000027	up

1425	A_32_P62276	LOC10050720(	2.029486861	0.02166201	up
1426	A_23_P202565	SHOC2	2.029370528	0.000125509	up
1427	A_23_P139143	STX3	2.028913651	0.000097	up
1428	A_23_P116902	ART4	2.028776351	0.002268405	up
1429	A_23_P204947	GJB2	2.02873482	0.00150187	up
1430	A_33_P3325467	C1orf212	2.028653777	0.000246018	up
1431	A_24_P921366	CALD1	2.028309907	0.00000358	up
1432	A_33_P3353170	CLTC	2.02818605	0.00000157	up
1433	A_33_P3269423	C13orf35	2.02758866	0.000142307	up
1434	A_23_P251151	NELL1	2.026730131	0.000772872	up
1435	A_33_P3223923	PDIA3	2.025931694	0.0000357	up
1436	A_24_P169073	FAM131C	2.0254119	0.001137091	up
1437	A_33_P3375413	LOC10013171!	2.025382278	0.000597374	up
1438	A_23_P23356	RRP15	2.025365338	0.0000314	up
1439	A_32_P226186	KIAA1549	2.025176058	0.000114453	up
1440	A_23_P983	PRDX6	2.025076862	0.0000271	up
1441	A_24_P264909	NDFIP2	2.025067832	0.00000202	up
1442	A_33_P3260100	CCDC167	2.024599668	0.0000037	up
1443	A_24_P364296	STX2	2.023687699	0.00000382	up
1444	A_33_P3330125	DIABLO	2.022774038	0.002179574	up
1445	A_33_P3544887	TTC28	2.021713175	0.019861203	up
1446	A_33_P3369631	EIF3J	2.021646192	0.0000314	up
1447	A_23_P109026	KCNK15	2.021380616	0.000489734	up
1448	A_24_P141736	METAP2	2.0213596	0.000105388	up
1449	A_33_P3356255	ANXA11	2.020395406	0.021745908	up
1450	A_24_P374427	ZDHHC21	2.019657747	0.000000912	up
1451	A_33_P3368301	BOLA3	2.018828465	0.0000782	up
1452	A_33_P3240996	LOC10013424(	2.017801607	0.000267478	up
1453	A_33_P3238215	COBLL1	2.017683192	0.000202935	up
1454	A_32_P44568	LDHA	2.017162439	0.0000402	up
1455	A_23_P60210	RLN1	2.017137831	0.009213974	up
1456	A_23_P53763	C13orf18	2.016967447	0.000984902	up
1457	A_33_P3327663	SUSD4	2.016388782	0.000590375	up
1458	A_33_P3323048	SLC10A7	2.016261367	0.0000771	up
1459	A_24_P294832	PTP4A1	2.015292621	0.0000456	up
1460	A_33_P3260134	NTRK3	2.015207179	0.002994341	up
1461	A_33_P3422888	CLEC16A	2.014890122	0.00000705	up
1462	A_23_P75630	APOA5	2.014785612	0.000590809	up
1463	A_24_P256155	NKX1-2	2.014539602	0.0000593	up
1464	A_23_P51679	MEF2D	2.013906912	0.000172287	up
1465	A_23_P149649	SDHB	2.013449284	0.00000357	up
1466	A_23_P144916	GFPT2	2.012673285	0.00000999	up
1467	A_33_P3260969	PRAP1	2.012368018	0.0000783	up
1468	A_32_P62963	KRT16P2	2.01177208	0.017063964	up
1469	A_23_P128201	NCKAP1L	2.007790072	0.0000181	up
1470	A_33_P3328251	LOC10028801	2.007705041	0.048182803	up
1471	A_33_P3210343	ETV6	2.007397977	0.000000149	up
1472	A_33_P3214943	SPOCK2	2.007192382	0.0000108	up
1473	A_24_P108451	GPI	2.007032066	0.0000457	up
1474	A_23_P360240	MYEOV	2.006731596	0.032498456	up
1475	A_23_P47116	RASSF7	2.006132645	0.033488359	up

1476	A_23_P68669	CHODL	2.005298492	0.000255085	up
1477	A_33_P3251896	APBB2	2.00425212	0.00000167	up
1478	A_23_P27528	CYP2A7	2.002830041	0.000490996	up
1479	A_33_P3229002	C17orf50	2.001937499	0.049786975	up
1480	A_23_P200222	LRP8	2.001325737	0.000544384	up
1481	A_33_P3360972	EXOC3L4	2.000861999	0.0000778	up
1482	A_33_P3254756	LOC550112	2.000598969	0.000329906	up
1483	A_33_P3247624	REP15	2.000399986	0.0000158	up
1484	A_33_P3222892	SLC10A3	15.3200684	0.0000162	up
1485	A_32_P48466	LCA5L	11.65759407	0.0006526	up
1486	A_33_P3422654	LOC10013398	10.99706223	0.002281103	up
1487	A_33_P3402868	GRIN2D	9.887128847	0.0000248	up
1488	A_23_P10206	HAS2	9.661669704	0.000241089	up
1489	A_33_P3260373	ISY1-RAB43	9.653374619	0.00000978	up
1490	A_32_P225345	C11orf88	9.200954289	0.001725937	up
1491	A_23_P390704	STK38	8.112231369	0.001930079	up
1492	A_33_P3760937	LOC497256	7.755348073	0.0000592	up
1493	A_23_P50052	C18orf12	7.137624474	0.002293227	up
1494	A_23_P29365	RYBP	6.734244802	0.0000702	up
1495	A_24_P307808	KCTD16	6.27486636	0.0000723	up
1496	A_24_P225604	DNAJC10	6.266268116	0.000000512	up
1497	A_33_P3229452	FLJ44477	6.002900826	0.0000261	up
1498	A_23_P8906	LRP12	5.768868988	0.00000605	up
1499	A_23_P127128	DNAJC1	5.707981531	0.000144426	up
1500	A_32_P133840	TMCC2	5.663606504	0.003274473	up
1501	A_33_P3410454	GTF2IRD2B	5.548840883	0.008859007	up
1502	A_33_P3338166	NUAK2	5.359887674	0.001533241	up
1503	A_23_P319640	LOC151534	5.313741944	0.00027124	up
1504	A_23_P66739	SLC13A5	5.299191143	0.000614457	up
1505	A_23_P11279	ALG13	5.275033783	0.0000443	up
1506	A_23_P171117	MORC4	5.265659864	0.000000438	up
1507	A_33_P3357247	USP36	5.168034138	0.0000461	up
1508	A_33_P3245489	ADAMTSL5	5.09112669	0.000734087	up
1509	A_33_P3390868	SYNPO2	4.999025894	0.0000716	up
1510	A_33_P3355599	PCDHB7	4.925893717	0.000247638	up
1511	A_23_P102694	DEFB129	4.881420381	0.000588147	up
1512	A_23_P36226	SLC25A33	4.838286831	0.000141002	up
1513	A_33_P3341970	NEGR1	4.631285406	0.001163025	up
1514	A_24_P203953	LOC439951	4.433715707	0.000332714	up
1515	A_23_P58763	PELO	4.367473855	0.0000684	up
1516	A_24_P400172	LOC10013069	4.361439165	0.001279419	up
1517	A_24_P322771	TFF1	4.327851708	0.000374023	up
1518	A_24_P355493	LHPP	4.320318978	0.00000704	up
1519	A_33_P3234490	BOLA2B	4.232007155	0.000408078	up
1520	A_33_P3247252	LOC729549	4.186852808	0.006023574	up
1521	A_23_P28590	FASTKD1	4.120286527	0.0000483	up
1522	A_33_P3353263	CYB5RL	3.91182591	0.00000147	up
1523	A_33_P3223182	TPTE2P3	3.84427533	0.000181142	up
1524	A_24_P250227	NR1D1	3.832732648	0.004957389	up
1525	A_23_P204702	TMBIM6	3.818858328	0.0000973	up
1526	A_23_P366394	ZAK	3.783089829	0.000322088	up

1527	A_33_P3317589	GFRA4	3.747194682	0.044473465	up
1528	A_33_P3317253	PTER	3.715679674	0.000002	up
1529	A_33_P3244141	NDEL1	3.675262256	0.0000159	up
1530	A_32_P215318	ACACA	3.659644895	0.013232305	up
1531	A_33_P3214466	MESP1	3.600097636	0.00029431	up
1532	A_23_P83976	CEP112	3.597688636	0.00034635	up
1533	A_23_P30283	FAM174A	3.595996625	0.00000489	up
1534	A_23_P139919	CHST11	3.567115921	0.00000676	up
1535	A_33_P3358938	CLOCK	3.476527175	0.000000126	up
1536	A_24_P319942	SSR3	3.449104086	0.000392001	up
1537	A_23_P154875	BACE2	3.436726394	0.0000263	up
1538	A_24_P211709	XPNPEP2	3.433579815	0.000707695	up
1539	A_23_P307310	ACAN	3.352733938	0.000248613	up
1540	A_23_P78108	ALDOC	3.34122306	0.00000604	up
1541	A_24_P23995	RNF187	3.338680024	0.0000312	up
1542	A_33_P3335386	FAM83G	3.331182435	0.0000737	up
1543	A_23_P333951	DNAH14	3.290590499	0.0000154	up
1544	A_24_P11061	CSAG1	3.285020712	0.000182073	up
1545	A_23_P111995	LOXL2	3.282683972	0.00000302	up
1546	A_33_P3223495	FRY	3.26820361	0.0000894	up
1547	A_23_P404091	GRPEL2	3.25256187	0.0000554	up
1548	A_23_P65518	DACT1	3.172910367	0.0000151	up
1549	A_24_P252364	NRCAM	3.169352953	0.000046	up
1550	A_33_P3333992	LOC10013249	3.139096399	0.006083723	up
1551	A_24_P365515	FOXA2	3.121455936	0.001253567	up
1552	A_33_P3285911	KIAA1919	3.117042588	0.000116704	up
1553	A_33_P3802116	LOC157860	3.0966128	0.000688193	up
1554	A_33_P3218120	LOC402036	3.089953798	0.000144762	up
1555	A_23_P425750	ARMC6	3.028270333	0.014583663	up
1556	A_23_P165668	SLC35F5	2.974000864	0.0000358	up
1557	A_23_P153524	C19orf73	2.96491662	0.0000286	up
1558	A_32_P75284	KATNAL1	2.959513833	0.0000978	up
1559	A_33_P3377514	HOXA1	2.937494872	0.00000857	up
1560	A_32_P191441	C10orf90	2.935723103	0.003295066	up
1561	A_24_P721699	A1BG-AS1	2.922335196	0.00000288	up
1562	A_33_P3279124	FAM21C	2.917606125	0.005102555	up
1563	A_23_P112798	CRIP2	2.913050543	0.028916468	up
1564	A_33_P3268310	LIMS3L	2.879048049	0.000412694	up
1565	A_33_P3407925	VMP1	2.872357192	0.0000811	up
1566	A_33_P3216292	TBC1D2	2.867169575	0.000588778	up
1567	A_23_P19182	REEP2	2.854286329	0.0000486	up
1568	A_33_P3249768	DGKK	2.847848369	0.000934838	up
1569	A_23_P417415	ACOT11	2.847351233	0.00000219	up
1570	A_24_P74559	CYTH4	2.824234012	0.0000854	up
1571	A_23_P126727	NOL9	2.785972467	0.00046031	up
1572	A_23_P41114	CSTA	2.748773881	0.0000296	up
1573	A_33_P3262575	BAIAP2L1	2.730297577	0.000066	up
1574	A_23_P70307	SMOC2	2.716489242	0.001893036	up
1575	A_23_P68219	TTN	2.703400766	0.004576022	up
1576	A_23_P385081	SCAMP2	2.691511644	0.0000505	up
1577	A_33_P3236858	TGFB1I1	2.685005419	0.0000178	up

1578	A_24_P358328	TPI1P2	2.672501393	0.0000076	up
1579	A_33_P3344991	TBC1D3	2.659052939	0.0000441	up
1580	A_33_P3344574	SFTPA2	2.656708977	0.00000828	up
1581	A_33_P3216438	SPATA21	2.65607245	0.002502028	up
1582	A_23_P148273	MAGT1	2.655241408	0.0000014	up
1583	A_23_P385063	DNAJB6	2.620031215	0.000193019	up
1584	A_23_P209183	GLT25D1	2.60627262	0.000602844	up
1585	A_23_P209320	PER2	2.599036389	0.00000825	up
1586	A_33_P3415698	TMIGD2	2.596641359	0.00000224	up
1587	A_33_P3394405	LOC727721	2.588410318	0.00000486	up
1588	A_23_P6771	LMCD1	2.580921628	0.0000843	up
1589	A_23_P35205	RCAN3	2.551513603	0.00000946	up
1590	A_23_P26976	CHAD	2.544690739	0.000593717	up
1591	A_33_P3255964	CYMP	2.542598503	0.011528754	up
1592	A_23_P71790	MAMDC4	2.541648162	0.0000911	up
1593	A_23_P147918	S100A16	2.536129182	0.0000147	up
1594	A_33_P3365117	AKR1C1	2.533233974	0.0000555	up
1595	A_33_P3240258	RPN2	2.532710008	0.00000119	up
1596	A_32_P192970	ALDH4A1	2.52736652	0.000000589	up
1597	A_33_P3391796	NOG	2.515281379	0.002417001	up
1598	A_23_P115223	HAX1	2.506572448	0.0000597	up
1599	A_33_P3261433	LOC10013147[illegible]	2.485294184	0.000777441	up
1600	A_33_P3390357	SPIRE1	2.470287345	0.0000813	up
1601	A_33_P3326553	TMEM82	2.458331433	0.000920559	up
1602	A_33_P3233871	F12	2.456070149	0.000000381	up
1603	A_23_P132956	UCHL1	2.413472408	0.000927503	up
1604	A_33_P3248213	CSNK1A1P1	2.409730488	0.000486425	up
1605	A_23_P76622	DCT	2.407243363	0.002297527	up
1606	A_23_P128613	KDELC1	2.405188557	0.000039	up
1607	A_33_P3661631	PCBD2	2.38489624	0.000155658	up
1608	A_33_P3370555	GALC	2.383787554	0.000071	up
1609	A_33_P3378360	PRELID1	2.376926732	0.0000492	up
1610	A_33_P3364205	SBDSP1	2.370974259	0.0000547	up
1611	A_33_P3268686	FBXW12	2.362025926	0.000368576	up
1612	A_33_P3397486	ENTPD4	2.351133023	0.000000596	up
1613	A_24_P105564	PRKAB2	2.346691182	0.0000301	up
1614	A_33_P3333527	MSTO1	2.335146398	0.0000455	up
1615	A_24_P12136	S100Z	2.330468803	0.033097561	up
1616	A_23_P138125	FAIM3	2.328566903	0.01876136	up
1617	A_33_P3401658	PSG2	2.326515824	0.000297492	up
1618	A_23_P127175	SAR1A	2.320879575	0.0000502	up
1619	A_33_P3239087	FAM87B	2.315082597	0.000278468	up
1620	A_32_P181638	BVES	2.307532552	0.0000657	up
1621	A_33_P3374289	C10orf82	2.306101267	0.000133901	up
1622	A_33_P3414242	MOG	2.299042549	0.001131352	up
1623	A_23_P64792	KCNMB4	2.295137202	0.0000191	up
1624	A_23_P416142	DLG1	2.294473588	0.000328986	up
1625	A_23_P215517	KLHL7	2.289087695	0.00000186	up
1626	A_33_P3358312	OTUD6B	2.285919079	0.000000954	up
1627	A_23_P410965	KIAA1522	2.282982815	0.000000138	up
1628	A_24_P383762	RASGEF1C	2.270896834	0.018247718	up

1629	A_33_P3382281	C20orf94	2.25033814	0.0000982	up
1630	A_33_P3318646	CALY	2.248052456	0.000294526	up
1631	A_23_P124108	ITGAM	2.246850862	0.000593845	up
1632	A_23_P89902	RTN2	2.241711934	0.000653889	up
1633	A_23_P357929	SLC35D1	2.24000792	0.0000468	up
1634	A_24_P151692	POF1B	2.235236782	0.001225553	up
1635	A_23_P409438	IL28A	2.223265124	0.000659354	up
1636	A_33_P3222439	TCP10L2	2.219210215	0.002188778	up
1637	A_23_P4400	KRTAP4-11	2.216620153	0.02665422	up
1638	A_24_P256654	LOC401588	2.212923022	0.000266348	up
1639	A_33_P3424132	LOC158376	2.207430629	0.0000895	up
1640	A_23_P16110	OR7E24	2.207121626	0.0000321	up
1641	A_33_P3402615	SLC6A9	2.194062313	0.0000805	up
1642	A_23_P402787	FAM100A	2.189870666	0.000238856	up
1643	A_24_P167614	INTS6	2.188037807	0.0000129	up
1644	A_33_P3214481	P4HA1	2.18732081	0.000062	up
1645	A_23_P207967	CTIF	2.17602279	0.002237255	up
1646	A_23_P147786	RIMS2	2.170991684	0.0000297	up
1647	A_33_P3400152	LOC728558	2.1631143	0.006893537	up
1648	A_24_P347880	ALOXE3	2.161529711	0.000436703	up
1649	A_23_P38677	SLMO1	2.154887172	0.00000585	up
1650	A_33_P3313920	KIR2DL2	2.149333319	0.002171149	up
1651	A_23_P45751	CLCA4	2.14242287	0.000034	up
1652	A_33_P3247082	SLC6A10P	2.141549959	0.00000346	up
1653	A_23_P2181	CYB5R2	2.139509423	0.001111391	up
1654	A_33_P3401980	SPRED3	2.123347798	0.029933658	up
1655	A_33_P3298617	LOC10050899	2.123270089	0.007560348	up
1656	A_33_P3240507	KCTD12	2.119257217	0.00005	up
1657	A_33_P3384432	TFDP2	2.117855757	0.0000795	up
1658	A_23_P47226	YIF1A	2.103183895	0.000000434	up
1659	A_33_P3257030	LEPREL4	2.101532353	0.0000557	up
1660	A_33_P3235117	LOC10013328	2.099246138	0.0000645	up
1661	A_23_P212089	NFKBIZ	2.096594221	0.001222551	up
1662	A_24_P36745	CXorf38	2.094540867	0.000561612	up
1663	A_23_P307536	SH3D21	2.088364925	0.00101766	up
1664	A_23_P14165	GPR18	2.087007811	0.008525128	up
1665	A_24_P407930	SEH1L	2.081313997	0.0000106	up
1666	A_23_P167479	IL17B	2.074725302	0.006215444	up
1667	A_33_P3365524	SETD3	2.069926107	0.0000403	up
1668	A_23_P252471	PECAM1	2.067829211	0.037373549	up
1669	A_23_P145357	BAK1	2.065626384	0.0000527	up
1670	A_33_P3292844	NEUROG3	2.063503044	0.04405845	up
1671	A_23_P257649	RBP1	2.062820088	0.001098894	up
1672	A_23_P419107	TCP11L2	2.061113187	0.000190964	up
1673	A_33_P3291776	NANS	2.055258129	0.00000523	up
1674	A_33_P3222630	FBXO43	2.045708616	0.017135732	up
1675	A_32_P439662	DNAH17	2.04529319	0.003736002	up
1676	A_33_P3331882	CRTC1	2.042195619	0.000171836	up
1677	A_23_P405282	MGC45922	2.03539011	0.000837663	up
1678	A_23_P259272	WSB2	2.024028634	0.000241597	up
1679	A_33_P3340525	SYT10	2.018426053	0.000169916	up

1680	A_23_P257256	GRK6	2.0147102	0.0000379	up
1681	A_33_P3519683	ZBTB8OS	2.013890161	0.000147096	up
1682	A_33_P3423954	CBX2	2.011179245	0.0154933	up
1683	A_33_P3368830	LY9	2.010700634	0.0000837	up
1684	A_33_P3322388	SPRR2D	2.01061706	0.000184052	up
1685	A_23_P32414	MST4	2.044787232	0.000317552	up
1686	A_24_P205589	ACOT7	2.044538174	0.000804482	up
1687	A_33_P3247599	ZNF805	2.044408744	0.00000417	up
1688	A_23_P204144	KRT85	2.043982248	0.042630581	up
1689	A_33_P3290955	KIAA1875	2.0437059	0.01038533	up
1690	A_23_P17955	FBXL2	2.042853386	0.0000364	up
1691	A_33_P3522525	GRPEL1	2.041845208	0.0000852	up
1692	A_24_P37887	GPR150	2.041511225	0.000560335	up
1693	A_23_P350754	OR7E13P	2.041087692	0.0000526	up
1694	A_23_P101054	KRT34	2.040738886	0.001671144	up
1695	A_23_P6535	KLHDC7B	2.039246251	0.018541281	up
1696	A_24_P332623	CTAGE11P	2.039238477	0.000277835	up
1697	A_24_P916718	ZNF467	2.038682532	0.000297856	up
1698	A_23_P8640	GPER	2.038166154	0.000173033	up
1699	A_23_P363313	SLC16A11	2.038150896	0.001917808	up
1700	A_33_P3274696	GPR172A	2.036163389	0.00000101	up
1701	A_33_P3243337	C19orf10	2.035481346	0.0000292	up
1702	A_24_P59607	C17orf51	2.033913983	0.00000298	up
1703	A_33_P3371089	SGTB	2.03328155	0.000000605	up
1704	A_23_P313728	FAM98A	2.032351583	0.000159238	up
1705	A_23_P137391	ENO1	2.032260018	0.0000146	up
1706	A_23_P218505	LHB	2.031737709	0.0000041	up
1707	A_23_P45294	FAM199X	2.031442693	0.0000523	up
1708	A_33_P3273409	SLC35E1	2.031326294	1.45E-09	up
1709	A_23_P160537	C1orf135	2.0305161	0.0010904	up
1710	A_33_P3571120	AP3D1	2.030202076	0.000275921	up
1711	A_24_P392110	PSG8	2.02976682	0.000173581	up
1712	A_24_P24685	HMGB3P22	2.029611314	0.002024304	up
1713	A_33_P3210848	ELFN1	2.029486861	0.000684836	up
1714	A_24_P153568	MPEG1	2.029370528	0.009104548	up
1715	A_23_P115838	C10orf12	2.028913651	6.02E-08	up
1716	A_23_P78526	CEACAM19	2.028776351	0.000238449	up
1717	A_23_P97632	EPRS	2.028653777	0.0000354	up
1718	A_33_P3351617	MYO3A	2.028309907	0.003199944	up
1719	A_23_P71904	METTL11A	2.02758866	0.000144578	up
1720	A_33_P3416568	AIG1	2.026730131	0.000735527	up
1721	A_33_P3373745	BRD4	2.025931694	0.000173276	up
1722	A_23_P21673	KIAA1797	2.0254119	0.000134885	up
1723	A_23_P302038	POU4F2	2.025382278	0.001774337	up
1724	A_33_P3388938	MGC23284	2.025365338	0.000266718	up
1725	A_23_P20832	SPTAN1	2.025176058	0.005102233	up
1726	A_33_P3867461	DKFZp686O13	2.025076862	0.004615798	up
1727	A_23_P405878	C12orf54	2.025067832	0.0000516	up
1728	A_33_P3594214	OR7E104P	2.023687699	0.00000119	up
1729	A_24_P388810	SRP19	2.022774038	0.00000361	up
1730	A_33_P3385436	PLAC8L1	2.021887743	0.000412352	up

1731	A_33_P3417086	NIPAL1	2.021713175	0.028471453	up
1732	A_33_P3316770	SLCO1A2	2.021646192	0.0000795	up
1733	A_23_P54991	DYNLL2	2.021380616	0.0000281	up
1734	A_23_P98085	PTEN	2.0213596	0.0000293	up
1735	A_24_P372134	TMEM140	2.020395406	0.000216277	up
1736	A_23_P88351	ATL1	2.019657747	0.00014131	up
1737	A_23_P132915	FAM114A1	2.019233383	0.0000288	up
1738	A_23_P258814	DPH3P1	2.018828465	6.27E-09	up
1739	A_23_P147025	RAB33A	2.017801607	0.000109167	up
1740	A_23_P57379	CDC45	2.017683192	0.000000462	up
1741	A_24_P930111	SLC4A10	2.017162439	0.003940932	up
1742	A_33_P3264188	OR2D3	2.016967447	0.008348136	up
1743	A_32_P46840	LOC729680	2.016388782	0.000236912	up
1744	A_33_P3381235	LOC10012788	2.016261367	0.00000128	up
1745	A_23_P379327	FAM63B	2.015292621	0.00000195	up
1746	A_23_P82286	ZAN	2.015207179	0.001834332	up
1747	A_33_P3279379	DSTNP2	2.014890122	0.0000531	up
1748	A_33_P3226557	PRR23B	2.014785612	0.00000195	up
1749	A_23_P47857	TM7SF3	2.014539602	0.00000821	up
1750	A_23_P330616	WIPF1	2.013906912	0.005777198	up
1751	A_33_P3418091	ISLR2	2.013449284	0.035418029	up
1752	A_23_P130194	PYCR1	2.012368018	0.0000387	up
1753	A_33_P3389342	ARID5A	2.01177208	0.0000698	up
1754	A_23_P57658	HRASLS	2.010567028	0.014190637	up
1755	A_23_P88893	DEF8	2.008185676	0.00000719	up
1756	A_23_P85800	CD52	2.007790072	0.005276582	up
1757	A_33_P3232038	RBAK-LOC389	2.007705041	0.0000274	up
1758	A_24_P288685	IL13RA1	2.007397977	0.0000375	up
1759	A_23_P45955	TEKT2	2.007192382	0.039280963	up
1760	A_24_P263672	BMS1	2.007032066	0.0000355	up
1761	A_33_P3272948	C17orf107	2.006731596	0.000033	up
1762	A_33_P3341676	MEF2A	2.006132645	0.00000848	up
1763	A_33_P3294297	RNF126	2.005298492	0.000174409	up
1764	A_33_P3313215	LRIT2	2.005170711	0.0000543	up
1765	A_33_P3303031	FLJ40606	2.002830041	0.006572659	up
1766	A_33_P3285334	LOC10013041	2.002653185	0.001410547	up
1767	A_23_P77980	SLC4A1	2.001937499	0.003739186	up
1768	A_33_P3249529	PCNX	2.001325737	0.000131682	up
1769	A_33_P3257503	LOC387647	2.000861999	0.0000421	up
1770	A_23_P372925	KIAA1737	2.000598969	0.0000356	up
1771	A_23_P143981	FBLN2	2.000399986	0.000539264	up
1772	A_23_P41476	SHISA3	478.1657545	1.17E-08	down
1773	A_24_P291658	ADH1A	313.5856287	0.000000303	down
1774	A_33_P3295358	ANGPTL4	253.1984273	2.58E-08	down
1775	A_33_P3238290	FAM65C	124.8234862	9.62E-09	down
1776	A_23_P27306	COLEC12	111.8936044	4.21E-09	down
1777	A_23_P121926	SEPP1	104.2580858	0.000000494	down
1778	A_33_P3332018	FGL2	94.68161	8.82E-08	down
1779	A_24_P124349	PDGFD	77.33951971	0.000000574	down
1780	A_23_P52266	IFIT1	69.61337904	1.91E-08	down
1781	A_23_P324754	KIAA1199	68.50248092	0.000000132	down

1782	A_23_P81158	ADH1C	63.92630343	0.00000133	down
1783	A_33_P3275702	FMO2	61.46704963	0.00000119	down
1784	A_23_P347169	MTUS1	54.88092528	0.00000349	down
1785	A_24_P260101	MME	54.14487063	1.46E-09	down
1786	A_23_P434398	TXLNB	51.41363106	0.000000038	down
1787	A_23_P211680	MLC1	51.07378829	0.0000109	down
1788	A_23_P205959	ALDH1A3	49.34252594	7.27E-08	down
1789	A_23_P200780	TGFBR3	44.7127074	0.000000383	down
1790	A_23_P34345	VCAM1	40.90483661	0.000000231	down
1791	A_33_P3255404	CLDN11	37.86313596	2.32E-08	down
1792	A_23_P382705	TMTC2	35.61415445	0.000000287	down
1793	A_23_P329261	KCNJ2	34.65792156	1.04E-08	down
1794	A_23_P101960	ZFP36L2	33.31627806	0.000000184	down
1795	A_23_P163216	ATP8B4	28.71960496	4.03E-08	down
1796	A_23_P144476	SPRY1	28.57787621	0.000000341	down
1797	A_23_P202978	CASP1	26.76919593	0.00000211	down
1798	A_23_P137470	SIPA1L2	26.6040995	0.000000386	down
1799	A_23_P369899	TMEM158	26.41261063	0.000000194	down
1800	A_23_P102391	SLC40A1	25.44690571	0.000000658	down
1801	A_23_P121064	PTX3	23.20875133	9.4E-10	down
1802	A_23_P118615	ABCA8	22.5466726	0.000000664	down
1803	A_33_P3244728	LRP2	21.39329106	0.000226498	down
1804	A_23_P83134	GAS1	20.30086084	0.00000106	down
1805	A_23_P54144	BMP4	20.05115356	0.00000336	down
1806	A_23_P218858	ABI3BP	20.02211768	0.00000143	down
1807	A_33_P3221129	LRRN4CL	20.00736588	0.000000437	down
1808	A_23_P252062	PPARG	19.86048983	0.00000016	down
1809	A_23_P72117	SMPDL3A	19.73626609	7.82E-08	down
1810	A_33_P3270489	C6orf97	19.45297452	0.00000118	down
1811	A_24_P579356	ARHGAP28	19.29155368	0.0000214	down
1812	A_23_P79968	PCSK2	19.23510011	0.000054	down
1813	A_23_P69573	GUCY1A3	19.11002994	0.000000485	down
1814	A_24_P403417	PTGES	18.15683202	0.0000671	down
1815	A_24_P941167	APOL6	18.07554489	0.00019708	down
1816	A_33_P3304983	PRKAR2B	17.96364568	0.00000177	down
1817	A_33_P3283480	CTSC	17.93339424	0.000000239	down
1818	A_33_P3213822	KCNK2	17.58757333	0.000000105	down
1819	A_24_P48204	SECTM1	17.26996356	2.43E-09	down
1820	A_32_P202013	FAM196A	17.1404225	0.000000239	down
1821	A_33_P3390013	ADAMTS1	17.09310716	8.65E-09	down
1822	A_23_P74278	PDE4B	17.00161118	0.000000321	down
1823	A_23_P110253	KIT	16.761181	0.000000192	down
1824	A_23_P213857	C7	15.95513488	0.000000697	down
1825	A_23_P378416	GPM6B	15.93574873	0.000000453	down
1826	A_24_P226755	TOX	15.93281965	7.38E-08	down
1827	A_23_P73097	RGS20	15.83098262	5.56E-08	down
1828	A_23_P18447	PPARGC1A	15.82194547	0.00000799	down
1829	A_23_P33326	ADRA1B	15.6773075	2.81E-08	down
1830	A_23_P52227	GDF10	15.60030684	1.02E-08	down
1831	A_24_P208436	PDE1A	15.49572338	0.00000542	down
1832	A_23_P12082	CHI3L2	15.44222678	0.000000548	down

1833	A_23_P501831	C5orf4	15.35005231	0.00000225	down
1834	A_23_P64173	CARD16	15.29250985	0.00000939	down
1835	A_23_P362191	NCRNA00324	15.02690613	0.0000228	down
1836	A_23_P201808	PPAP2B	14.90631417	5.06E-08	down
1837	A_24_P108311	NEDD4L	14.84654573	0.00000175	down
1838	A_23_P61406	SHC3	14.82578921	0.000000136	down
1839	A_23_P8820	FABP4	14.66342011	0.0000185	down
1840	A_23_P156890	TCF21	14.33705365	0.00000516	down
1841	A_24_P133253	KITLG	14.22401817	0.000000169	down
1842	A_24_P759477	ITGB8	13.98620192	0.000000359	down
1843	A_23_P156861	RGS17	13.97781998	2.86E-08	down
1844	A_32_P46214	SLC9A9	13.97269109	1.61E-08	down
1845	A_33_P3276693	PGF	13.56258399	0.000000982	down
1846	A_33_P3276703	VGF	13.48189822	0.0000203	down
1847	A_23_P63402	GPSM2	13.46317742	0.00000345	down
1848	A_23_P53137	HBG1	13.2612972	0.00000925	down
1849	A_23_P127948	ADM	13.1887354	1.31E-12	down
1850	A_23_P131208	NR4A2	13.07055805	0.0000178	down
1851	A_23_P257583	DENND2A	13.01286898	1.08E-08	down
1852	A_24_P915692	PHLDA1	12.92617927	0.000000901	down
1853	A_24_P870620	PTN	12.66909881	0.000000213	down
1854	A_33_P3225273	QSOX1	12.3436866	0.003408547	down
1855	A_33_P3263217	LRRC4	12.31982024	0.000230021	down
1856	A_23_P431268	PLEKHA6	12.26135771	0.00000274	down
1857	A_32_P88262	SLC7A14	12.23701972	0.000000526	down
1858	A_23_P217379	COL4A6	12.13693754	0.00000202	down
1859	A_33_P3240018	PDE3B	12.11951393	0.00000191	down
1860	A_24_P200023	IL1R1	11.98511627	0.000000403	down
1861	A_23_P114740	CFH	11.94099099	0.000000339	down
1862	A_33_P3283611	IFIT3	11.93650026	0.000000945	down
1863	A_24_P928052	NRP1	11.92746323	6.7E-10	down
1864	A_33_P3212575	NNAT	11.90050178	6.33E-08	down
1865	A_23_P138352	WNT2B	11.83322901	0.000000698	down
1866	A_32_P164246	FOXQ1	11.75327765	0.00000396	down
1867	A_23_P69810	AGPAT9	11.74039058	0.00000518	down
1868	A_23_P167920	DLL1	11.59066015	0.000000149	down
1869	A_23_P404481	S1PR1	11.51552276	0.00000155	down
1870	A_24_P681011	HIPK2	11.50166512	0.0000288	down
1871	A_23_P134854	CLDN23	11.28078349	0.000000518	down
1872	A_33_P3232552	GUCY1B3	11.24843224	0.00000527	down
1873	A_23_P85682	NFIA	11.13664703	0.0000214	down
1874	A_33_P3599591	PAPPA	11.12140381	0.00000145	down
1875	A_23_P145984	TSPAN12	11.05652458	9.09E-08	down
1876	A_24_P333857	SGIP1	11.04850298	0.00000107	down
1877	A_33_P3372426	ADAMTS5	11.0218228	0.00000388	down
1878	A_23_P12884	GRK5	11.01043579	0.00000176	down
1879	A_23_P111311	AKAP12	10.98969595	0.000000417	down
1880	A_23_P82868	PLAT	10.90675984	1.37E-08	down
1881	A_33_P3352522	FLJ41484	10.89980888	0.00000538	down
1882	A_33_P3307363	LPHN2	10.83717883	0.000000196	down
1883	A_23_P216361	COL14A1	10.8033125	0.00000182	down

1884	A_24_P274814	TBXAS1	10.78201512	0.000000151	down
1885	A_24_P192805	CARD17	10.69170797	0.000000191	down
1886	A_33_P3237379	KCNC1	10.56638297	0.000202127	down
1887	A_23_P138541	AKR1C3	10.53283794	0.0000016	down
1888	A_33_P3230269	GRHL1	10.43516398	0.00000167	down
1889	A_23_P24948	KCNE3	10.30677778	0.00000344	down
1890	A_33_P3331125	SLC2A12	10.19742247	0.00000217	down
1891	A_33_P3217983	ACSL5	10.18465637	0.000000101	down
1892	A_24_P940275	FRMPD4	9.993242517	0.000358043	down
1893	A_23_P372308	RGMA	9.959651995	1.25E-08	down
1894	A_33_P3368771	C3orf47	9.891497836	0.0000725	down
1895	A_33_P3377130	MAP3K5	9.754061036	0.00000868	down
1896	A_24_P382187	IGFBP4	9.740138814	0.000599056	down
1897	A_23_P115011	ADAMTSL4	9.730913865	0.000101452	down
1898	A_24_P141214	STOM	9.667295448	0.0000009	down
1899	A_23_P500400	ABCA6	9.653970155	0.000737603	down
1900	A_24_P220485	OLFML2A	9.642273376	0.000000224	down
1901	A_23_P26386	TPPP3	9.612491607	0.000131566	down
1902	A_33_P3301940	PDE7B	9.60994384	0.00000197	down
1903	A_23_P414958	PLXNC1	9.578997652	0.000176493	down
1904	A_23_P134426	GPNMB	9.576567625	7.17E-08	down
1905	A_24_P243749	PDK4	9.545255841	0.00000498	down
1906	A_33_P3423941	IFITM1	9.510839346	4.02E-08	down
1907	A_32_P60065	F2RL2	9.444243027	0.0000105	down
1908	A_23_P113161	C1orf21	9.367180292	1.26E-08	down
1909	A_23_P420551	CIT	9.349797808	0.000000437	down
1910	A_23_P162211	MANSC1	9.331598917	0.00000261	down
1911	A_24_P171268	RASSF5	9.296880854	0.000000235	down
1912	A_32_P47157	FP588	9.283261554	0.00000344	down
1913	A_32_P128656	MID1	9.278389002	0.000000079	down
1914	A_23_P217428	ARHGAP6	9.218489863	0.00000262	down
1915	A_32_P208403	GNG2	9.210768292	0.000000268	down
1916	A_23_P76914	SIX1	9.062078574	2.13E-10	down
1917	A_33_P3317603	B4GALNT4	9.025387033	0.000370406	down
1918	A_24_P402080	MBP	8.941206804	0.000000284	down
1919	A_33_P3270514	NBLA00301	8.904038105	0.00000509	down
1920	A_24_P49260	SPTLC3	8.890008715	0.00000202	down
1921	A_33_P3226810	TNFSF10	8.888721343	0.000154268	down
1922	A_23_P385322	STAMBPL1	8.866393138	0.00020622	down
1923	A_33_P3250348	PCDH18	8.805900237	0.00000436	down
1924	A_32_P196021	FGF7	8.764786607	0.000000965	down
1925	A_23_P45324	TMEM35	8.598858573	0.0000172	down
1926	A_23_P420196	SOCS1	8.536918068	0.000000165	down
1927	A_23_P216596	SVEP1	8.493001472	0.00000325	down
1928	A_33_P3773168	LOC285768	8.486503056	0.000281316	down
1929	A_32_P37867	KIAA1644	8.478065427	0.00000125	down
1930	A_33_P3237110	GPR137C	8.430179012	0.00000172	down
1931	A_23_P11685	PLA2G4A	8.429804851	0.000000398	down
1932	A_33_P3212615	TFPI	8.398860852	0.00000134	down
1933	A_23_P77993	C1QL1	8.384797838	0.000118872	down
1934	A_23_P65307	SLITRK6	8.281252586	0.0000287	down

1935	A_23_P385067	CLIC6	8.21027588	0.000185467	down
1936	A_33_P3369058	LRRK2	8.200056404	8.93E-08	down
1937	A_23_P304897	BDKRB2	8.191747991	0.0000018	down
1938	A_33_P3423185	DPF3	8.158983559	0.0000166	down
1939	A_23_P205867	NR2E3	8.126104589	0.000000696	down
1940	A_23_P55544	CCBE1	8.086080502	0.0000027	down
1941	A_33_P3368855	GPC5	8.083938615	0.0000081	down
1942	A_24_P55496	OSR2	8.065485806	0.0000132	down
1943	A_24_P941359	FAM65B	8.035506516	0.00000565	down
1944	A_23_P417918	PENK	8.008288416	0.0000204	down
1945	A_33_P3239455	GTF2IRD2	7.939014328	0.0000455	down
1946	A_24_P321581	SLC38A4	7.938720111	0.00000382	down
1947	A_23_P140057	TNFRSF19	7.881552473	0.001037387	down
1948	A_23_P155596	FMO3	7.867317278	0.0000274	down
1949	A_24_P344961	AMOT	7.850440819	0.00000214	down
1950	A_33_P3278078	LOC10050550	7.846501609	0.0000128	down
1951	A_32_P198731	NEURL1B	7.817765262	0.000000602	down
1952	A_23_P352266	BCL2	7.812011827	8.52E-08	down
1953	A_23_P63209	HSD11B1	7.809974836	0.0000157	down
1954	A_23_P21324	TWIST2	7.783120356	0.00000154	down
1955	A_33_P3276713	HGF	7.762899624	0.0000114	down
1956	A_23_P159974	KLHL13	7.738897939	0.000000851	down
1957	A_33_P3369371	GPX3	7.714678845	0.0000584	down
1958	A_23_P58251	CPZ	7.702738599	0.0000824	down
1959	A_23_P345460	PLEKHG4	7.638651998	0.000000146	down
1960	A_23_P30163	FLJ13197	7.63567254	0.00000546	down
1961	A_23_P82503	PEG10	7.634655185	0.00000225	down
1962	A_33_P3403117	NR2F1	7.587103228	0.00000212	down
1963	A_33_P3376546	PTGFR	7.543647344	0.00000831	down
1964	A_33_P3384462	THSD4	7.536494696	1.64E-08	down
1965	A_33_P3368646	CNKSR3	7.520251965	0.00000582	down
1966	A_23_P85952	DENND2D	7.478579325	0.00000293	down
1967	A_33_P3419190	AREG	7.474971092	0.00000301	down
1968	A_33_P3281283	S1PR3	7.448837726	0.000000303	down
1969	A_23_P207367	STAT5A	7.431073128	0.00000402	down
1970	A_23_P18123	NLGN1	7.4007742	0.0000156	down
1971	A_23_P415611	NHEDC1	7.381178605	0.00000931	down
1972	A_33_P3300837	LDB2	7.375951997	0.0000376	down
1973	A_24_P158946	FGD4	7.358605197	0.0000171	down
1974	A_33_P3385266	ABCC6	7.319008055	1.69E-08	down
1975	A_24_P12065	CCNG2	7.293831139	7.55E-08	down
1976	A_23_P48936	SMAD3	7.241170898	8.47E-08	down
1977	A_23_P134601	WNT16	7.230600996	0.0000529	down
1978	A_24_P208567	IL18R1	7.217559855	0.00000544	down
1979	A_33_P3408918	SAA2	7.213378699	0.000158791	down
1980	A_24_P330263	EDNRB	7.175264428	0.00000233	down
1981	A_23_P216307	RUNX1T1	7.161470069	0.000133149	down
1982	A_23_P14986	HSD11B2	7.14191848	0.000000303	down
1983	A_23_P32404	ISG20	7.13162577	0.0000241	down
1984	A_23_P391396	EBF3	7.095295812	0.000011	down
1985	A_23_P74701	COL24A1	7.091979831	0.000000153	down

1986	A_33_P3849275	FHL1	7.086944086	0.0000016	down
1987	A_32_P108156	MIR155HG	7.049205351	0.000000514	down
1988	A_33_P3367636	C6orf221	7.047864723	0.0000177	down
1989	A_23_P29939	SNCA	7.034218585	0.000000631	down
1990	A_33_P3279590	OGN	6.975535624	0.00000601	down
1991	A_23_P400449	VAT1L	6.948360788	0.0000426	down
1992	A_33_P3393650	PLEKHA5	6.914108133	0.00041612	down
1993	A_33_P3289227	DNALI1	6.894968688	0.00000809	down
1994	A_32_P2452	TMTC1	6.879413147	0.00000316	down
1995	A_23_P103511	C1orf226	6.87350202	0.0000162	down
1996	A_33_P3213362	CASC2	6.855372691	0.006195849	down
1997	A_23_P14124	RASL11A	6.853315478	0.000000655	down
1998	A_33_P3263890	PRRX1	6.805002382	0.00000282	down
1999	A_23_P33759	DHRS3	6.736361992	7.63E-08	down
2000	A_32_P86905	KGFLP2	6.723140659	0.00000972	down
2001	A_23_P80974	TDO2	6.71997547	0.00000187	down
2002	A_33_P3412468	SLC25A27	6.712221075	0.001063188	down
2003	A_33_P3374623	ABCA7	6.665416989	0.000095	down
2004	A_23_P118065	HSD17B2	6.646964429	0.00000278	down
2005	A_33_P3383326	LPAR1	6.629415453	0.000000748	down
2006	A_24_P235338	TRPA1	6.627349178	0.00000208	down
2007	A_33_P3414591	TWIST1	6.609364678	0.0000565	down
2008	A_23_P97402	CAMK1G	6.595555775	0.00000855	down
2009	A_24_P237778	MAN1C1	6.543824393	0.0000511	down
2010	A_32_P790284	KATNAL2	6.531314442	0.00000523	down
2011	A_23_P255104	LHFPL2	6.468307057	0.000000819	down
2012	A_32_P205624	SHC2	6.455970859	0.00000282	down
2013	A_23_P156708	TNXB	6.454648651	0.00000008	down
2014	A_23_P328621	UBQLNL	6.447949896	0.000000274	down
2015	A_23_P24004	IFIT2	6.416381102	0.000000662	down
2016	A_24_P289178	C16orf74	6.404837643	0.000000573	down
2017	A_24_P229531	OBFC2A	6.402938857	0.0000155	down
2018	A_23_P16953	HTR2B	6.394270013	0.00018269	down
2019	A_23_P126908	TNFRSF14	6.382145797	0.00000754	down
2020	A_23_P86470	CH25H	6.358832029	0.00000422	down
2021	A_33_P3285868	CYGB	6.339050084	0.000000165	down
2022	A_33_P3290403	IMPA2	6.309263188	0.000109072	down
2023	A_33_P3729375	LOC285181	6.297528736	0.0000063	down
2024	A_23_P30666	TNFRSF21	6.278110115	0.00000672	down
2025	A_24_P10137	C13orf15	6.265408897	0.00000136	down
2026	A_32_P146113	C6orf81	6.254377357	0.000225312	down
2027	A_33_P3314176	FAM46C	6.19651835	0.000000161	down
2028	A_23_P163087	NID2	6.17217514	0.00000232	down
2029	A_23_P255896	C2orf89	6.150918669	0.02633848	down
2030	A_23_P112103	GSDMD	6.129313654	0.001605034	down
2031	A_33_P3424062	KCNF1	6.113186156	0.0000301	down
2032	A_23_P369701	FAM108C1	6.108280582	0.00000289	down
2033	A_24_P80500	BDH2	6.079824586	0.000000795	down
2034	A_23_P433016	FBLN1	6.070191371	0.0000198	down
2035	A_23_P76136	TSPAN11	6.031393378	0.000000527	down
2036	A_23_P82929	NOV	5.99612226	0.00000658	down

2037	A_23_P300033	PDGFRA	5.97188617	0.000000478	down
2038	A_24_P219474	MGAT5B	5.962074075	0.00000324	down
2039	A_23_P18078	RARRES1	5.958960427	0.00000141	down
2040	A_33_P3259135	D4S234E	5.949211905	0.00000513	down
2041	A_32_P112493	PKDCC	5.942608476	0.000362832	down
2042	A_23_P35725	ANO3	5.938487508	0.000000284	down
2043	A_33_P3259557	LOC440104	5.932046883	0.00000379	down
2044	A_23_P32577	DACH1	5.925817381	0.0000708	down
2045	A_23_P134835	CSGALNACT1	5.92269311	0.000000123	down
2046	A_23_P384044	CNIH3	5.911070934	0.00000275	down
2047	A_23_P61487	LRRC20	5.893781869	0.000000247	down
2048	A_24_P140569	LRRTM2	5.891748986	0.000000754	down
2049	A_23_P363778	FRZB	5.869657541	0.000000426	down
2050	A_33_P3235053	TRIM47	5.860965452	0.00000171	down
2051	A_24_P304154	AMPD3	5.86077858	0.00000235	down
2052	A_33_P3368014	HVCN1	5.859254708	0.00000332	down
2053	A_33_P3388466	BTN3A1	5.859090226	0.0000425	down
2054	A_33_P3341601	WDR86	5.854078669	0.018736901	down
2055	A_24_P68079	TRANK1	5.847560298	0.000000823	down
2056	A_23_P9293	TJP2	5.836751581	0.00000225	down
2057	A_23_P428129	CDKN1C	5.818129198	1.46E-08	down
2058	A_24_P183128	PLAC8	5.812971492	0.00000156	down
2059	A_23_P164047	MMD	5.807886796	9.68E-09	down
2060	A_24_P945113	ACVRL1	5.755933802	0.00000314	down
2061	A_23_P88626	ANPEP	5.754989648	0.00010294	down
2062	A_23_P135239	TLE1	5.748869705	0.000000339	down
2063	A_33_P3413815	LAMB2P1	5.740555965	0.0000135	down
2064	A_23_P310022	KIAA1217	5.731501092	0.0000191	down
2065	A_23_P14564	GPR65	5.729530406	0.000000823	down
2066	A_23_P69497	CLEC3B	5.724169264	0.000000979	down
2067	A_23_P360079	NCKAP5	5.695290244	0.0000183	down
2068	A_23_P428298	UNC5CL	5.689646947	0.000309219	down
2069	A_23_P326931	TTC18	5.680898395	0.000000644	down
2070	A_24_P150580	RASL12	5.676052106	0.0000321	down
2071	A_23_P115645	CELF2	5.673259805	0.00000201	down
2072	A_33_P3379061	C9orf47	5.660951513	0.0000119	down
2073	A_24_P200854	HOXA2	5.65906026	0.0000182	down
2074	A_23_P210763	JAG1	5.633453702	0.00000135	down
2075	A_23_P37375	RPS6KA5	5.602129241	0.000088	down
2076	A_23_P48771	C14orf159	5.60130246	0.000000377	down
2077	A_23_P157117	CREB5	5.59767037	0.000000123	down
2078	A_33_P3355921	LOC10013089	5.578242423	0.0000162	down
2079	A_32_P44453	INPP1	5.562243873	0.0000058	down
2080	A_23_P301521	KIAA1462	5.555070932	0.00000217	down
2081	A_33_P3347697	NOVA1	5.553979941	0.0000205	down
2082	A_33_P3338733	MITF	5.541343626	0.000102384	down
2083	A_33_P3255914	MYLIP	5.53667306	0.0000122	down
2084	A_23_P66017	PRRT2	5.535819231	0.00000335	down
2085	A_32_P4018	ROR1	5.520011095	0.00000102	down
2086	A_23_P302550	RGS18	5.506586585	0.00021292	down
2087	A_24_P238499	C18orf56	5.503350826	0.0000119	down

2088	A_23_P69109	PLSCR1	5.495147984	0.000000015	down
2089	A_23_P75786	SLC15A3	5.464508096	0.000000522	down
2090	A_32_P158966	KLRF1	5.463449156	0.000324543	down
2091	A_33_P3328863	PNMAL2	5.446923354	0.000512208	down
2092	A_33_P3333777	LOC10012938	5.430050192	0.000000987	down
2093	A_23_P316447	FGFR1	5.41320003	0.000011	down
2094	A_33_P3230478	C1S	5.412404509	0.003403987	down
2095	A_33_P3231047	CCDC121	5.402398268	0.0000846	down
2096	A_33_P3261982	PRIM2	5.38463094	0.000589201	down
2097	A_23_P57709	PCOLCE2	5.383105997	0.000000928	down
2098	A_23_P157007	TMEM176B	5.374166057	0.00000568	down
2099	A_32_P148538	LPPR4	5.374021153	0.000000206	down
2100	A_23_P44466	CCDC102B	5.372766599	0.00000492	down
2101	A_23_P30243	ERAP2	5.356716087	0.00000269	down
2102	A_33_P3420259	RN28S1	5.351961607	0.0000187	down
2103	A_23_P139704	DUSP6	5.345380573	0.00000303	down
2104	A_23_P89780	LAMA3	5.340965382	0.00000483	down
2105	A_23_P307844	PHYHD1	5.299172533	0.0000318	down
2106	A_23_P87049	SORL1	5.297605939	0.00004	down
2107	A_24_P217904	TRERF1	5.294462888	0.00000558	down
2108	A_33_P3231739	ELOVL2	5.286104333	0.00000987	down
2109	A_33_P3361422	CYP27A1	5.264918991	0.00000123	down
2110	A_33_P3363168	SSH2	5.247123209	4.93E-08	down
2111	A_23_P358709	AHRR	5.208905149	5.63E-08	down
2112	A_33_P3257513	FAT3	5.206957267	0.0000698	down
2113	A_24_P555510	PCM1	5.199732239	0.000000975	down
2114	A_33_P3363720	C1orf213	5.198610979	0.00000244	down
2115	A_33_P3367301	GJD3	5.197478073	0.00000384	down
2116	A_33_P3418025	CTSO	5.191215337	0.000000433	down
2117	A_33_P3264269	MOV10	5.164898628	0.021738824	down
2118	A_23_P216225	EGR3	5.157457401	0.000011	down
2119	A_23_P212119	GALNTL2	5.148307883	0.000058	down
2120	A_23_P202448	CXCL12	5.133925996	0.00000179	down
2121	A_24_P411561	HAVCR2	5.127646953	0.0000232	down
2122	A_23_P13753	NFE2	5.11890938	0.000021	down
2123	A_23_P49539	BAHCC1	5.115512083	0.0000529	down
2124	A_24_P926507	SLC14A1	5.109051695	0.0000693	down
2125	A_23_P143713	APOBEC3G	5.102594749	0.0000659	down
2126	A_23_P118834	TOP2A	5.088315991	0.0000505	down
2127	A_23_P97700	TXNIP	5.079682527	0.00000832	down
2128	A_33_P3266010	RIN1	5.079490051	0.000062	down
2129	A_24_P342632	AK5	5.078095171	0.0000147	down
2130	A_32_P101917	LOC400550	5.065854005	0.00000183	down
2131	A_23_P151133	TSPAN9	5.064232591	0.000337064	down
2132	A_23_P25615	SOHLH2	5.052566366	0.0000088	down
2133	A_33_P3245415	N4BP2L1	5.049311095	0.000000117	down
2134	A_23_P324327	GPRC5B	5.02887723	0.0000408	down
2135	A_24_P252078	BTN3A2	5.020861167	0.00000107	down
2136	A_23_P30254	PLK2	5.017755473	0.00000356	down
2137	A_33_P3414880	LOC339192	5.010028348	0.00103358	down
2138	A_33_P3219245	C9orf130	4.997110776	0.0000185	down

2139	A_33_P3288159	ASPM	4.991231432	0.0000165	down
2140	A_33_P3350374	C10orf58	4.98312524	0.00080712	down
2141	A_23_P94840	DYNLRB2	4.98014587	0.00000508	down
2142	A_33_P3593774	PIK3R3	4.970190675	0.000215496	down
2143	A_33_P3407324	GULP1	4.965249337	0.00000218	down
2144	A_23_P165727	MSTN	4.938042774	0.021971045	down
2145	A_33_P3305731	NINL	4.907566102	0.002620151	down
2146	A_23_P502470	IL6ST	4.894577166	0.000462112	down
2147	A_23_P416965	FAM149A	4.890201345	0.000743615	down
2148	A_23_P21207	UBA7	4.88559238	0.00003	down
2149	A_33_P3312601	AADACL4	4.878622204	0.00011512	down
2150	A_23_P414312	KNDC1	4.870752916	0.0000196	down
2151	A_23_P85082	RHOXF1	4.870585462	0.000563153	down
2152	A_23_P104563	CPT1A	4.869787096	0.000000415	down
2153	A_24_P115932	GPR44	4.863423191	0.00000221	down
2154	A_24_P658427	NFIB	4.848881959	8.34E-08	down
2155	A_23_P39131	GLTSCR2	4.839445878	0.025649046	down
2156	A_33_P3410589	FAM43A	4.831462621	5.36E-08	down
2157	A_23_P8834	EPHX2	4.825741822	0.000000316	down
2158	A_23_P252541	RAB7B	4.825190272	0.0000156	down
2159	A_32_P86763	TGM2	4.820273478	0.006454376	down
2160	A_23_P363255	CCDC68	4.815577652	0.000000861	down
2161	A_24_P406060	RNF144B	4.8151789	0.00000158	down
2162	A_23_P49816	ADAP2	4.796879947	0.0000419	down
2163	A_24_P8220	HS6ST1	4.7901583	0.000000583	down
2164	A_33_P3269218	BAI2	4.773800136	0.00000395	down
2165	A_23_P128215	SOCS2	4.769395205	0.00000848	down
2166	A_23_P66891	CDC42EP4	4.767319556	0.000011	down
2167	A_23_P374695	TEK	4.753758675	5.31E-08	down
2168	A_23_P430930	RSPO2	4.741966486	1.56E-08	down
2169	A_23_P164451	TBX2	4.734626652	0.000046	down
2170	A_23_P95930	HMGA2	4.733763619	0.00000221	down
2171	A_33_P3356361	LOC10013108	4.723456264	0.00000359	down
2172	A_23_P27229	MYO15A	4.710351211	0.00000023	down
2173	A_23_P363878	RFTN2	4.701077073	0.00000567	down
2174	A_23_P424582	EGFL8	4.699028442	0.000000607	down
2175	A_33_P3252281	EYA4	4.694707148	0.0000568	down
2176	A_33_P3306146	PLAU	4.686290645	0.002811348	down
2177	A_32_P103695	FAM92A1	4.680869898	8.03E-08	down
2178	A_23_P110196	HERC5	4.668482688	0.0000809	down
2179	A_23_P93772	HOXA5	4.668418833	0.000000165	down
2180	A_23_P139682	PZP	4.658364297	0.00000679	down
2181	A_23_P103256	CFHR3	4.650179587	0.0000181	down
2182	A_33_P3342126	PRDM8	4.646224398	0.00000369	down
2183	A_23_P252082	TMEM176A	4.641470557	0.00000907	down
2184	A_23_P150325	TMEM133	4.640242598	0.00000112	down
2185	A_23_P211207	ADARB1	4.63508994	0.000000924	down
2186	A_23_P94800	S100A4	4.619658905	0.0000219	down
2187	A_33_P3363560	TMEM51	4.613088596	0.00000857	down
2188	A_32_P169179	MSX2P1	4.594251175	0.00000377	down
2189	A_33_P3210278	SYNE2	4.588527222	0.00000116	down

2190	A_24_P658584	SASH1	4.58279473	0.00000345	down
2191	A_33_P3397073	LOC10050754	4.581998967	0.0000204	down
2192	A_23_P363316	HOXB5	4.581920203	0.000000249	down
2193	A_23_P45365	COL4A5	4.579148441	0.000000413	down
2194	A_23_P105138	CAT	4.577553243	1.22E-08	down
2195	A_23_P74088	MMP23B	4.575162545	0.000000835	down
2196	A_23_P89431	CCL2	4.562330624	0.0000103	down
2197	A_33_P3326225	SAMD12	4.559153658	0.0000152	down
2198	A_23_P254741	SOD3	4.557745916	0.00000382	down
2199	A_33_P3245183	HRH1	4.554200054	0.00000025	down
2200	A_24_P396702	CD302	4.550611739	1.05E-08	down
2201	A_23_P370588	HOXB8	4.547305611	0.000241141	down
2202	A_23_P259251	KCND2	4.544549854	0.0000143	down
2203	A_23_P200260	PCNXL2	4.544279274	0.00000216	down
2204	A_23_P358410	SRGAP3	4.53348643	0.005107523	down
2205	A_23_P26629	PYCARD	4.533215252	0.00000116	down
2206	A_23_P107247	CACNA1G	4.526935967	0.00000618	down
2207	A_24_P416997	APOL3	4.519842915	0.0000201	down
2208	A_33_P3290562	GLI3	4.516833811	0.0000216	down
2209	A_32_P167705	AGBL2	4.508765049	0.00000305	down
2210	A_33_P3281191	NID1	4.507841115	0.000000389	down
2211	A_24_P626850	H6PD	4.501078211	0.000201997	down
2212	A_23_P407614	PYDC1	4.497239864	0.0000794	down
2213	A_33_P3256828	LOC10012791	4.485832962	0.0000759	down
2214	A_24_P7143	MYO1B	4.485259946	0.00060521	down
2215	A_23_P1691	MMP1	4.479215259	0.0000122	down
2216	A_23_P415021	METTL7A	4.478395679	0.0000312	down
2217	A_24_P224488	MAPT	4.474811157	0.00000366	down
2218	A_23_P256084	ARSE	4.472859784	0.00000367	down
2219	A_23_P11800	CAMK2N1	4.465567924	0.00000361	down
2220	A_23_P60499	ZNF462	4.458676	0.000539133	down
2221	A_23_P135548	DPYD	4.456685017	0.00000373	down
2222	A_33_P3243832	ZEB2	4.440269778	0.000118358	down
2223	A_23_P45185	FIGF	4.438665632	0.00000973	down
2224	A_24_P82155	ANKDD1A	4.438392946	0.0000312	down
2225	A_24_P397255	ENPP6	4.418303021	0.000139659	down
2226	A_23_P110571	MAST4	4.415344481	0.000684713	down
2227	A_33_P3352019	SCARA3	4.410005128	0.000000272	down
2228	A_23_P3221	SQRDL	4.407893397	0.00000472	down
2229	A_33_P3371718	SAT1	4.401402573	0.00000633	down
2230	A_23_P23074	IFI44	4.397163975	0.000000552	down
2231	A_24_P6517	PLEKHG1	4.393685687	0.0000898	down
2232	A_33_P3308744	LAMA4	4.391156644	8.19E-08	down
2233	A_33_P3227691	SLC9A5	4.38629355	0.000144877	down
2234	A_23_P36658	MGST1	4.382949329	0.00000413	down
2235	A_23_P112289	TMOD1	4.379946557	0.000000533	down
2236	A_23_P134953	PLIN2	4.379691748	0.000000992	down
2237	A_24_P163237	STOX2	4.373172732	0.0000204	down
2238	A_23_P36305	ATG16L2	4.36925629	0.00000921	down
2239	A_23_P120594	ACSS1	4.36860813	0.000000593	down
2240	A_33_P3403723	LOC10013030	4.365539635	0.00000555	down

2241	A_33_P3361811	NAA16	4.357333285	0.004653367	down
2242	A_33_P3548860	SEC1	4.35042325	0.000021	down
2243	A_24_P235429	ABCA1	4.349979192	0.0000206	down
2244	A_33_P3365408	MATL2963	4.349074633	0.000330269	down
2245	A_32_P204239	CDHR3	4.344390338	0.000200535	down
2246	A_32_P117354	LIMCH1	4.342499953	0.002665501	down
2247	A_23_P18372	B3GNT5	4.341930299	0.00000917	down
2248	A_32_P358887	SLC4A4	4.33606535	0.00000558	down
2249	A_23_P69863	PCDHB5	4.32586747	0.0000116	down
2250	A_33_P3404531	ZCWPW1	4.318480075	0.000834968	down
2251	A_23_P41470	DDX60	4.314265113	0.00000122	down
2252	A_24_P896205	LOC645722	4.313873884	0.000000404	down
2253	A_32_P178499	C16orf86	4.313036425	0.0000342	down
2254	A_23_P42282	C4B	4.304595475	0.00000123	down
2255	A_33_P3280237	CNRIP1	4.302152206	0.039487038	down
2256	A_23_P158925	GPR125	4.300352731	0.00000245	down
2257	A_24_P342312	ODZ4	4.296679996	0.00000236	down
2258	A_33_P3398448	PARP10	4.277578508	0.00000453	down
2259	A_33_P3237775	NR1H3	4.272071126	0.00000747	down
2260	A_24_P182494	DUSP10	4.271904316	0.000073	down
2261	A_23_P206901	NDE1	4.271769097	0.0000134	down
2262	A_23_P363968	C1RL	4.269029109	0.000000148	down
2263	A_23_P78742	FLT3LG	4.256065833	0.002447219	down
2264	A_23_P316410	NOX1	4.245784851	0.00001	down
2265	A_23_P29096	PDE9A	4.23864106	0.0000327	down
2266	A_23_P109171	BFSP1	4.232354093	0.0000164	down
2267	A_24_P192301	SEMA3A	4.227429904	0.000000256	down
2268	A_23_P169978	ZNF608	4.2273839	3.47E-08	down
2269	A_23_P396666	TBC1D2B	4.22686099	0.0000159	down
2270	A_33_P3368139	MAP3K1	4.224177623	0.0000153	down
2271	A_23_P22352	FRMD4A	4.219385781	0.000631758	down
2272	A_23_P258493	LMNB1	4.217917274	0.000179968	down
2273	A_23_P124190	TRIM34	4.213759282	0.00111565	down
2274	A_23_P383986	CHST15	4.209290269	0.00000296	down
2275	A_24_P303454	TIAM2	4.203277385	0.0000588	down
2276	A_33_P3560679	LOC10028722	4.200032719	0.0000106	down
2277	A_23_P86021	SELENBP1	4.186248634	0.000000207	down
2278	A_23_P211957	TGFBR2	4.178755456	0.00000326	down
2279	A_23_P151870	GLCE	4.175159077	0.0000466	down
2280	A_33_P3368750	PAQR5	4.174092196	0.0000744	down
2281	A_23_P313389	UGCG	4.164574304	0.00023192	down
2282	A_32_P57810	RNF157	4.164253512	0.0000326	down
2283	A_33_P3312242	CCL25	4.157845835	0.0000557	down
2284	A_33_P3287959	RASA4	4.150678723	0.0000019	down
2285	A_23_P2705	LPAR6	4.142426214	0.00000903	down
2286	A_33_P3300747	ADHFE1	4.137517364	0.000210099	down
2287	A_33_P3388983	C5orf56	4.129928486	0.00000613	down
2288	A_23_P415652	GALNT12	4.112403142	0.00000442	down
2289	A_23_P130735	SLC6A16	4.111340898	0.0000537	down
2290	A_33_P3325349	TSPAN5	4.107803014	0.000621164	down
2291	A_23_P128744	BDKRB1	4.099120308	0.000000873	down

2292	A_23_P34744	CTSK	4.095308119	0.00000404	down
2293	A_23_P416434	PHF15	4.087340027	0.0000125	down
2294	A_33_P3413483	SORD	4.082688979	0.00000848	down
2295	A_33_P3697530	SEMA4D	4.080525336	0.0000441	down
2296	A_33_P3404601	C2	4.079045312	0.0000341	down
2297	A_24_P396375	ECE1	4.078696051	0.010783452	down
2298	A_23_P218770	RAC2	4.074117719	0.000000476	down
2299	A_33_P3281795	MGLL	4.073181683	0.000000679	down
2300	A_23_P146294	EFCAB1	4.063574375	0.002217565	down
2301	A_23_P116898	A2M	4.056844487	0.000000206	down
2302	A_33_P3274930	PMP22	4.046848947	0.000099	down
2303	A_23_P210675	SYCP2	4.045987324	0.000018	down
2304	A_32_P107002	RUNDC2A	4.044488802	0.001177547	down
2305	A_24_P85775	C1orf38	4.040172429	0.00009	down
2306	A_23_P83175	PTPLAD2	4.033631622	1.06E-09	down
2307	A_32_P157208	LOC572558	4.026024635	0.0000218	down
2308	A_24_P15898	C1orf145	4.018581292	0.000375218	down
2309	A_23_P315364	CXCL2	4.017333318	0.000000799	down
2310	A_33_P3219939	CUBN	4.014793356	0.011694014	down
2311	A_32_P16258	EXOC6B	4.002780486	0.000105864	down
2312	A_32_P177040	SPDYE5	4.000591252	0.000000472	down
2313	A_23_P71379	PSCA	3.993854108	0.0000012	down
2314	A_23_P159775	GABRE	3.9883	0.0000181	down
2315	A_23_P151297	TENC1	3.98552239	0.003197624	down
2316	A_23_P85693	GBP2	3.981792479	0.0000121	down
2317	A_24_P30923	SNN	3.979707317	0.00000327	down
2318	A_23_P167096	VEGFC	3.979261381	0.001348706	down
2319	A_24_P71938	SMAD1	3.972239655	0.000000792	down
2320	A_23_P112004	LRRC6	3.969101621	0.00000852	down
2321	A_23_P204640	NANOG	3.967590598	0.000000214	down
2322	A_23_P44505	KLF11	3.965317355	0.00000562	down
2323	A_33_P3302453	NCRNA00242	3.963306477	0.00000146	down
2324	A_32_P117170	NAPEPLD	3.96159299	0.00000125	down
2325	A_24_P194886	EHBP1	3.958319502	0.000418563	down
2326	A_23_P334883	SHANK2	3.95810678	0.00000159	down
2327	A_23_P145024	ADRB2	3.949427029	0.00000936	down
2328	A_23_P158277	TMCO4	3.939934102	0.0000134	down
2329	A_23_P35349	SVIL	3.939009783	0.000591175	down
2330	A_23_P212800	FGF5	3.932218983	0.0000863	down
2331	A_33_P3242543	MAOA	3.931697064	0.000150021	down
2332	A_24_P732099	HBBP1	3.92467149	0.000000799	down
2333	A_23_P425967	SPDYE1	3.922235516	0.000177803	down
2334	A_23_P68031	STAT4	3.917043093	0.0000206	down
2335	A_23_P109452	CHEK2	3.910242731	0.00000989	down
2336	A_23_P156445	DDX43	3.907947177	0.000382095	down
2337	A_33_P3387766	LOC284276	3.899543739	0.0000159	down
2338	A_23_P211212	COL18A1	3.898654117	0.0000251	down
2339	A_33_P3613516	LOC254057	3.895639741	0.000039	down
2340	A_23_P88331	DLGAP5	3.886408185	0.0000763	down
2341	A_33_P3293869	TIAL1	3.884347215	0.000504389	down
2342	A_33_P3403576	FCGR2A	3.88367659	0.0000152	down

2343	A_23_P117298	F7	3.882628034	0.00000188	down
2344	A_32_P129419	NARG2	3.882451314	0.000054	down
2345	A_24_P655849	SMAD9	3.881210462	0.000504263	down
2346	A_23_P18798	PCDHB9	3.877963703	0.0000289	down
2347	A_23_P29684	VILL	3.870971896	0.00000148	down
2348	A_33_P3211633	WDR3	3.8597136	0.043364145	down
2349	A_33_P3320062	PLD1	3.855957811	0.00000368	down
2350	A_33_P3261828	LOC390660	3.852825912	0.0000111	down
2351	A_24_P365807	EFNB1	3.852448934	0.000000384	down
2352	A_33_P3369567	LSP1	3.850690044	0.0000014	down
2353	A_23_P398566	NR4A3	3.850219333	0.000329304	down
2354	A_24_P313993	CAPS	3.84914975	0.018941074	down
2355	A_23_P308136	TRIM50	3.847325875	0.002622768	down
2356	A_24_P51080	FLJ42709	3.843220718	0.00000179	down
2357	A_33_P3404052	TNFAIP8	3.841908962	0.00000948	down
2358	A_33_P3343101	ARGLU1	3.839196758	0.001611203	down
2359	A_23_P77908	SLC47A2	3.830294798	0.036146	down
2360	A_24_P90097	ADD3	3.828325945	0.00000342	down
2361	A_33_P3323760	LOC153684	3.828034061	0.00000046	down
2362	A_33_P3239242	SPATA6	3.826747029	0.00000819	down
2363	A_33_P3220919	ADRBK2	3.821877843	0.0000283	down
2364	A_32_P88120	YPEL1	3.81530837	0.0000315	down
2365	A_23_P46369	RAB13	3.811229979	0.005068212	down
2366	A_23_P138680	IL15RA	3.807914713	3.35E-09	down
2367	A_33_P3293346	KCTD3	3.807557965	0.008608234	down
2368	A_24_P10233	DAPK2	3.805268797	0.000107904	down
2369	A_24_P135769	VWA5A	3.805140963	0.0000376	down
2370	A_24_P85557	GPR37	3.802781112	0.00000533	down
2371	A_33_P3305203	LOC283588	3.795288351	0.0000025	down
2372	A_23_P117852	KIAA0101	3.794180464	0.00000029	down
2373	A_33_P3392517	LBX2	3.789159142	0.000000444	down
2374	A_23_P144096	CISH	3.788322974	0.00000354	down
2375	A_32_P199429	NCAM2	3.781998174	0.000000862	down
2376	A_33_P3327587	LOC10012790	3.780991746	0.0000745	down
2377	A_23_P10121	SFRP1	3.780329096	0.00000388	down
2378	A_33_P3306159	TNRC18	3.779774064	0.0372303	down
2379	A_24_P353619	ALPL	3.77604197	0.006874385	down
2380	A_23_P80594	PLCL2	3.775649911	0.00000135	down
2381	A_23_P87560	BTG1	3.773228579	0.0000583	down
2382	A_23_P109488	PIK3IP1	3.770194886	0.002049763	down
2383	A_23_P404606	C5orf41	3.767157452	0.000139262	down
2384	A_24_P307653	SLC6A15	3.765843206	0.00000475	down
2385	A_23_P17663	MX1	3.761351153	0.000168962	down
2386	A_33_P3421183	GTF2I	3.760901356	0.000000739	down
2387	A_33_P3294509	CD44	3.754024329	0.000000288	down
2388	A_33_P3279353	AZU1	3.746237937	0.0000362	down
2389	A_23_P327777	GATS	3.744494663	0.000014	down
2390	A_33_P3504659	CASP10	3.744037366	0.000237827	down
2391	A_24_P135748	GRTP1	3.739657987	0.000129627	down
2392	A_23_P388855	KAT6B	3.738150101	0.000425234	down
2393	A_33_P3544880	LOC142937	3.73715232	0.000078	down

2394	A_33_P3311498	LOC283392	3.727380241	0.000000754	down
2395	A_23_P67569	LPPR3	3.726693232	0.00019772	down
2396	A_33_P3303212	CCDC74B	3.725128605	0.012631028	down
2397	A_33_P3396089	SLC38A6	3.723892776	0.00000782	down
2398	A_23_P137665	CHI3L1	3.718673282	0.0000291	down
2399	A_33_P3210203	SPIRE2	3.71785997	0.0000067	down
2400	A_24_P204043	ZNF318	3.712846917	0.000218737	down
2401	A_23_P67971	GALM	3.709576519	0.000000912	down
2402	A_33_P3312544	GABRB1	3.704253074	0.00000074	down
2403	A_23_P111583	CD36	3.703275464	0.000117081	down
2404	A_33_P3220475	FLJ23867	3.702621728	0.000000382	down
2405	A_24_P54174	TNFRSF1B	3.696920621	0.0000361	down
2406	A_32_P116660	RBM43	3.696460935	0.00000989	down
2407	A_23_P129246	PLEKHO2	3.695108772	0.000000301	down
2408	A_23_P169437	LCN2	3.690587	0.008408218	down
2409	A_33_P3309075	TBC1D8	3.685640101	0.0000041	down
2410	A_23_P8981	STAR	3.684493053	0.00000263	down
2411	A_23_P109034	SDC4	3.683475976	0.00000499	down
2412	A_33_P3224362	PCBP3	3.680890076	0.001125116	down
2413	A_24_P185117	RILP	3.665600854	0.000000194	down
2414	A_23_P436284	OSTBETA	3.658688948	0.0000653	down
2415	A_33_P3216933	SIK2	3.651894278	0.000033	down
2416	A_23_P391228	MANEAL	3.642667299	0.00000296	down
2417	A_23_P214026	FBN2	3.638870392	0.00000418	down
2418	A_23_P25605	KBTBD7	3.63798182	0.000964795	down
2419	A_23_P23611	AMY1C	3.632401307	0.000130944	down
2420	A_23_P205746	EML1	3.630534594	0.000000648	down
2421	A_33_P3329013	SSTR2	3.625748527	0.000446227	down
2422	A_23_P218190	CAPN3	3.625696504	0.00000328	down
2423	A_32_P194312	SDK2	3.622668513	0.000260983	down
2424	A_23_P204016	CACNB3	3.619481394	0.000163378	down
2425	A_32_P126375	NHS	3.612757701	0.0000227	down
2426	A_33_P3378514	PDE5A	3.611289302	0.000000967	down
2427	A_23_P133359	ZFP2	3.608175386	0.000000419	down
2428	A_24_P357266	GRPR	3.604276106	0.001517241	down
2429	A_23_P38346	DHX58	3.599329052	0.0000116	down
2430	A_24_P16610	ZNRF1	3.597123852	0.000000114	down
2431	A_24_P406006	LPCAT1	3.595816334	0.000208474	down
2432	A_24_P16913	ABCC4	3.593828174	0.00000055	down
2433	A_23_P17593	CDH4	3.591963857	0.00071367	down
2434	A_32_P71113	SCAI	3.591036541	0.000000094	down
2435	A_23_P212241	CHL1	3.590924449	0.000000186	down
2436	A_23_P340848	PTGIR	3.58701091	0.000804789	down
2437	A_23_P316511	HOXB3	3.583682104	0.0000439	down
2438	A_23_P128574	ENOX1	3.577298485	0.00003	down
2439	A_33_P3350094	PATL2	3.576816733	0.000114975	down
2440	A_23_P72059	VSIG10	3.571738353	0.0000365	down
2441	A_23_P250564	PRKCE	3.568284551	0.00000224	down
2442	A_23_P151820	RIN3	3.568006722	0.001283284	down
2443	A_23_P150609	IGF2	3.560632813	0.000000721	down
2444	A_23_P254254	SGSH	3.559848884	0.00000274	down

2445	A_33_P3319134	LOC10050619	3.557665735	0.0000261	down
2446	A_23_P160992	FMO4	3.553339576	0.0000811	down
2447	A_33_P3571901	DKFZP586K15	3.549789804	0.00000266	down
2448	A_33_P3412016	SEMA4B	3.540590762	0.022138343	down
2449	A_23_P111000	PSMB9	3.538667885	0.0000109	down
2450	A_23_P14769	FES	3.537373034	0.0000268	down
2451	A_33_P3375145	C9orf150	3.532407216	0.00000272	down
2452	A_23_P31124	COL21A1	3.531527506	0.000106429	down
2453	A_23_P125082	IRF2	3.52777602	0.001559294	down
2454	A_33_P3287223	DPP4	3.526988406	0.000000228	down
2455	A_24_P941912	DTX3L	3.525116246	0.0000168	down
2456	A_23_P168669	CROT	3.523087647	0.0000417	down
2457	A_23_P216023	ANGPT1	3.522278537	0.0000187	down
2458	A_23_P208143	ZNF397	3.519959021	0.0000123	down
2459	A_23_P405873	C9orf72	3.518773454	0.004930729	down
2460	A_24_P391586	OAF	3.517533102	0.00146717	down
2461	A_33_P3442605	LOC389641	3.50098529	0.000134506	down
2462	A_32_P453321	C1orf228	3.49236222	0.00000202	down
2463	A_23_P427122	ART5	3.490872907	0.0000113	down
2464	A_24_P234732	MXD4	3.490685306	0.029482975	down
2465	A_23_P153853	ECH1	3.489493151	0.0000323	down
2466	A_33_P3797403	LOC283387	3.485095202	0.0000373	down
2467	A_23_P134176	SOD2	3.484029082	0.0000239	down
2468	A_23_P66635	CCL11	3.482493194	0.000000745	down
2469	A_23_P385105	PLCD4	3.47951799	0.00000161	down
2470	A_23_P204630	NTN4	3.474309632	0.00000165	down
2471	A_32_P94001	FAM92A3	3.472794397	0.000115522	down
2472	A_23_P30294	CDO1	3.457945905	0.00000115	down
2473	A_23_P212508	TF	3.456819162	0.00000415	down
2474	A_32_P47988	CAMK2D	3.454949923	0.000119768	down
2475	A_24_P322229	RASL10B	3.451613678	0.0000198	down
2476	A_23_P42306	HLA-DMA	3.450244656	0.0000921	down
2477	A_23_P377214	HEXIM2	3.447428587	0.000025	down
2478	A_33_P3531206	PCOTH	3.445065629	0.0000417	down
2479	A_33_P3419865	LOC646719	3.438574651	0.001167205	down
2480	A_23_P301855	LSAMP	3.435905918	0.000000247	down
2481	A_24_P134356	BTBD3	3.427425562	0.0000477	down
2482	A_23_P148047	PTGER4	3.427238678	0.00000291	down
2483	A_23_P114947	RGS2	3.426566454	0.0000351	down
2484	A_33_P3342598	PRKG2	3.414197147	0.000000605	down
2485	A_23_P34093	G6PD	3.413347743	0.024429266	down
2486	A_23_P169039	SNAI2	3.41256218	0.00005	down
2487	A_23_P24077	C10orf54	3.411555453	0.00000615	down
2488	A_24_P217365	ANKRD28	3.404878143	0.00000404	down
2489	A_24_P356916	SLC13A3	3.404817962	0.00000228	down
2490	A_24_P82493	DCAKD	3.404153282	0.00000768	down
2491	A_23_P90804	MAP4K4	3.398198985	0.000000568	down
2492	A_23_P143774	MOV10L1	3.395820183	0.00000145	down
2493	A_23_P142878	ATOH8	3.393590765	0.0000164	down
2494	A_33_P3289865	PLCL1	3.392738963	0.00000651	down
2495	A_23_P404965	GNL1	3.392223751	0.00000158	down

2496	A_23_P131263	MPP4	3.391079327	0.00000537	down
2497	A_32_P216734	SPDYE3	3.390558414	0.0000981	down
2498	A_23_P59714	MGC16142	3.390145751	0.000265162	down
2499	A_23_P31064	MOXD1	3.38994688	0.00000238	down
2500	A_33_P3418833	FLRT3	3.382112853	0.0000055	down
2501	A_33_P3220470	SMAD6	3.381783182	0.000000328	down
2502	A_23_P64617	FZD4	3.377698759	0.00000539	down
2503	A_33_P3243023	ADRA1D	3.369535596	0.00000432	down
2504	A_23_P422071	B3GALT4	3.368398125	0.000144566	down
2505	A_23_P129128	TARSL2	3.366773499	0.00000793	down
2506	A_23_P88880	ZNF423	3.366722937	0.0000576	down
2507	A_23_P58506	ELL2	3.364908636	0.0000105	down
2508	A_23_P131866	AURKA	3.36445774	0.000000052	down
2509	A_32_P196263	ADAMTS9	3.362880391	0.0000192	down
2510	A_23_P93383	RGL2	3.362245727	0.000248675	down
2511	A_33_P3605529	LOC400548	3.359854055	0.048571921	down
2512	A_33_P3222977	TEKT5	3.359124731	0.000958606	down
2513	A_23_P323272	OSR1	3.351971773	0.001136832	down
2514	A_24_P42446	PURG	3.350890013	0.0000047	down
2515	A_23_P60146	PDGFRL	3.348584405	0.00000583	down
2516	A_23_P411806	SLC44A1	3.348327551	0.0000665	down
2517	A_24_P102880	NAV1	3.342120305	0.00000248	down
2518	A_23_P99253	LIN7A	3.340416277	0.000049	down
2519	A_23_P412577	ANKRD29	3.335358568	0.0000293	down
2520	A_33_P3247205	MOSC1	3.333987742	0.00000599	down
2521	A_23_P1331	COL13A1	3.333873429	0.00000338	down
2522	A_33_P3234472	LOC284751	3.333670773	0.00000118	down
2523	A_33_P3317523	STMN1	3.333242623	0.0000119	down
2524	A_33_P3404989	HIST1H3H	3.332585448	0.000024	down
2525	A_23_P204208	KLRD1	3.331788911	0.0000986	down
2526	A_23_P84576	ANTXR1	3.330639788	0.000126625	down
2527	A_33_P3316278	ABCC6P1	3.329617532	0.0000114	down
2528	A_33_P3413989	SERPING1	3.328948996	0.009420596	down
2529	A_24_P102293	SLITRK5	3.327278282	0.0000126	down
2530	A_23_P356526	TRIM5	3.326026354	0.0000013	down
2531	A_24_P23625	HS3ST3B1	3.321289928	0.0000325	down
2532	A_24_P410797	KALRN	3.320974549	3.62E-09	down
2533	A_33_P3232354	BTBD8	3.319403244	0.00000172	down
2534	A_23_P253464	FAM175A	3.319123168	0.000000987	down
2535	A_23_P2203	TAOK3	3.318719123	0.000094	down
2536	A_33_P3252695	CYTL1	3.318510487	0.0000242	down
2537	A_23_P111041	HIST1H2BI	3.317947365	0.00000624	down
2538	A_24_P943301	PEAR1	3.31679313	0.0000341	down
2539	A_23_P122976	GNAI1	3.316548599	0.0000204	down
2540	A_23_P29422	GYG1	3.31457983	0.000100408	down
2541	A_23_P53126	LMO2	3.313561279	0.0000321	down
2542	A_33_P3400763	PLIN4	3.30843339	0.00000639	down
2543	A_23_P303833	SCN4B	3.303915906	0.00000195	down
2544	A_33_P3325497	FIBIN	3.302872167	0.00000941	down
2545	A_24_P157087	CASP8	3.294257128	0.000075	down
2546	A_33_P3226542	SNORD3B-1	3.288715024	0.0000764	down

2547	A_33_P3844650	ANGPTL2	3.286652665	0.00000231	down
2548	A_23_P69383	PARP9	3.286384616	0.000150108	down
2549	A_23_P67042	MOCOS	3.285295937	0.000024	down
2550	A_33_P3393927	MTMR11	3.284582266	0.00030285	down
2551	A_23_P94754	TNFSF15	3.283823682	0.0000707	down
2552	A_23_P347777	SYCP2L	3.282745332	0.000102791	down
2553	A_32_P827528	S1PR2	3.281696149	0.000000751	down
2554	A_32_P75264	TMEM26	3.279182737	0.00000672	down
2555	A_33_P3357530	SLC12A7	3.279022194	0.0000177	down
2556	A_24_P27229	CSAD	3.277229788	0.004139465	down
2557	A_24_P153576	SHPRH	3.275500192	0.000208166	down
2558	A_33_P3364062	FLJ31356	3.274995445	0.000666997	down
2559	A_23_P383325	CCDC110	3.272950235	0.0000837	down
2560	A_23_P165414	KLHL23	3.272005107	0.00000965	down
2561	A_24_P390668	FMNL1	3.268615854	0.001609691	down
2562	A_24_P330518	CA12	3.265812959	0.00000431	down
2563	A_33_P3281686	TSC2	3.263318949	0.000985237	down
2564	A_23_P71821	PBX3	3.261287587	0.00000725	down
2565	A_32_P160561	DOK6	3.260815166	0.00000711	down
2566	A_32_P197561	EBF1	3.257422527	0.0000284	down
2567	A_32_P62997	PBK	3.254461017	0.00015048	down
2568	A_23_P303978	LOC285943	3.253331122	0.0000133	down
2569	A_23_P80503	ROBO1	3.249645399	0.0000028	down
2570	A_24_P71649	CNTN3	3.247931036	0.0000147	down
2571	A_24_P254949	PGM5	3.247870102	0.00000258	down
2572	A_23_P32233	KLF4	3.247196447	0.000000253	down
2573	A_24_P3783	HIST1H2BM	3.246966875	0.00000154	down
2574	A_23_P124946	CMYA5	3.246344712	0.002514885	down
2575	A_33_P3376214	FMO5	3.242290819	1.99E-08	down
2576	A_23_P209564	CYBRD1	3.241396483	0.0000275	down
2577	A_23_P50108	NDC80	3.235638427	0.00000379	down
2578	A_32_P141418	ARMC4	3.231948031	0.0000264	down
2579	A_33_P3350488	NUSAP1	3.231846849	0.00000267	down
2580	A_33_P3233436	PNMA6C	3.230157027	0.000665359	down
2581	A_23_P390172	RNASEL	3.229377286	0.00000969	down
2582	A_23_P29922	TLR3	3.227806437	0.00000457	down
2583	A_33_P3226775	MUC20	3.223662181	0.0000367	down
2584	A_23_P3532	LITAF	3.222905752	0.00000615	down
2585	A_33_P3393170	CAPN5	3.222164911	0.00000162	down
2586	A_23_P95231	CASC1	3.221476938	0.000426474	down
2587	A_33_P3393821	C1R	3.221091552	0.00000211	down
2588	A_23_P140290	RTN1	3.218964947	0.005617786	down
2589	A_32_P202182	SPDYE8P	3.218673415	0.0000315	down
2590	A_23_P35912	CASP4	3.216127273	0.0000535	down
2591	A_24_P942211	SLC35E2	3.212746165	0.00000627	down
2592	A_23_P1962	RARRES3	3.210184019	0.000000935	down
2593	A_33_P3257140	ROCK2	3.209702611	1.64E-08	down
2594	A_33_P3371493	TOP1	3.208879023	0.001019196	down
2595	A_23_P23947	MAP3K8	3.207952693	0.00000545	down
2596	A_23_P85598	ADCK3	3.205783654	0.0000987	down
2597	A_23_P348383	CC2D2A	3.205187303	0.0000493	down

2598	A_33_P3234809	PAX8	3.200904054	0.000130191	down
2599	A_33_P3324137	PRO0628	3.197124665	0.000761069	down
2600	A_23_P134295	NUDT1	3.195119743	0.000000997	down
2601	A_23_P6381	MN1	3.193782352	0.000775139	down
2602	A_33_P3354539	CHURC1	3.192300951	0.0000186	down
2603	A_23_P151614	PSME1	3.185920647	0.0000504	down
2604	A_23_P384329	DENND4A	3.183641233	2.03E-09	down
2605	A_23_P116235	MDK	3.181517741	0.000000735	down
2606	A_33_P3234571	MAP2K3	3.180143279	0.018689391	down
2607	A_23_P158318	ROR2	3.173407227	0.0000129	down
2608	A_23_P17471	FAM113A	3.172809788	0.000445805	down
2609	A_23_P317465	RAB8B	3.169519916	0.000000745	down
2610	A_23_P47304	CASP5	3.169337575	0.00000767	down
2611	A_23_P426636	AHNAK	3.168772311	0.014391979	down
2612	A_33_P3367247	CNTLN	3.166380355	0.00000826	down
2613	A_23_P218807	ZC3H7B	3.164634021	0.00000425	down
2614	A_24_P944049	CEP68	3.163976753	0.0000798	down
2615	A_33_P3308347	ADAMTS8	3.163897437	0.000000557	down
2616	A_23_P316612	GLIS1	3.162686011	0.00000551	down
2617	A_33_P3679941	LOC283484	3.159751643	0.000161731	down
2618	A_32_P137266	EFCAB7	3.157663273	0.00000062	down
2619	A_23_P152002	BCL2A1	3.155435937	0.00176462	down
2620	A_24_P146211	HIST1H2BD	3.152723176	2.28E-08	down
2621	A_33_P3351120	TXNRD1	3.150983792	0.0000363	down
2622	A_23_P147397	DYNC2H1	3.150048046	0.00000719	down
2623	A_23_P368740	HDAC10	3.143207176	0.007527913	down
2624	A_33_P3282840	RPS29	3.141638827	0.0000021	down
2625	A_23_P413641	PREX1	3.137329013	0.000163904	down
2626	A_24_P829183	FLJ32255	3.137045237	0.00000136	down
2627	A_23_P70670	CD83	3.136602046	0.00028147	down
2628	A_33_P3367899	FRAT1	3.134183538	0.0000294	down
2629	A_23_P132718	SEMA3B	3.132848852	0.00000463	down
2630	A_33_P3410351	GSTM2	3.132417833	0.0000939	down
2631	A_33_P3237150	BMP2	3.131203774	0.00000153	down
2632	A_24_P381136	PACSIN3	3.126289253	0.01092921	down
2633	A_33_P3209229	RAB26	3.12569859	0.000000832	down
2634	A_23_P401	CENPF	3.124993091	0.0000805	down
2635	A_33_P3220837	MAFB	3.122399492	0.000134112	down
2636	A_24_P945228	CYP4V2	3.121638191	0.00000642	down
2637	A_33_P3753757	LOC158402	3.121562821	0.001596799	down
2638	A_23_P65022	ACADS	3.118113478	0.021464401	down
2639	A_33_P3396886	C11orf52	3.117889646	0.00000385	down
2640	A_23_P163195	LRFN5	3.117241511	0.0000223	down
2641	A_33_P3359856	MZT2A	3.115172169	0.000000884	down
2642	A_33_P3345304	LOC10051019	3.113624571	0.0000476	down
2643	A_24_P388786	DNAH5	3.112454327	0.0000088	down
2644	A_33_P3645465	LOC282997	3.108822685	0.0000213	down
2645	A_24_P345846	ANTXR2	3.10494038	0.031278191	down
2646	A_33_P3297444	ABTB1	3.098020448	0.00000136	down
2647	A_23_P7144	CXCL1	3.097686548	0.000291358	down
2648	A_32_P96036	MEX3A	3.097357336	0.00000661	down

2649	A_23_P62298	NLGN3	3.085831386	0.01141575	down
2650	A_32_P34387	SPATA7	3.084286962	0.000104234	down
2651	A_24_P228875	ARHGEF6	3.083210664	0.000000179	down
2652	A_33_P3220976	EFHB	3.080508821	0.002777659	down
2653	A_24_P944640	EPB41L5	3.079466641	0.0000184	down
2654	A_23_P111804	PARP12	3.078275523	0.00000297	down
2655	A_23_P358917	CYP3A7	3.07669692	0.00000335	down
2656	A_24_P49183	EXD3	3.076677869	0.003466451	down
2657	A_23_P416894	PION	3.076070354	0.0000297	down
2658	A_32_P205241	GJA3	3.076004613	0.0000525	down
2659	A_23_P401361	PITPNM2	3.07487856	0.001331896	down
2660	A_32_P204205	SIX4	3.071831681	0.0000562	down
2661	A_33_P3349466	ANKRD12	3.070731349	8.59E-08	down
2662	A_23_P207445	MAP2K6	3.069872136	0.000025	down
2663	A_33_P3296871	ZNF33B	3.069639001	0.0000458	down
2664	A_24_P649388	C21orf63	3.068721314	0.0000165	down
2665	A_33_P3351027	PEX2	3.065650999	0.000012	down
2666	A_24_P16124	IFITM4P	3.063068577	0.0000138	down
2667	A_23_P47034	HHEX	3.061456109	0.00000616	down
2668	A_23_P98686	ATHL1	3.060532454	0.0000252	down
2669	A_24_P294851	TRIM38	3.059926218	0.00000188	down
2670	A_23_P383009	IGFBP5	3.05630813	0.00000687	down
2671	A_32_P30831	LOC10050564	3.055973006	0.000235038	down
2672	A_23_P91910	PLSCR4	3.055514513	0.0000103	down
2673	A_23_P209288	CUL3	3.054799092	0.00000141	down
2674	A_33_P3655775	LOC284630	3.052943732	0.0000037	down
2675	A_33_P3257678	HIST2H3A	3.048623238	0.000227841	down
2676	A_24_P602871	SAMD5	3.047592479	0.0000196	down
2677	A_23_P108501	EPHA4	3.038776705	0.001048274	down
2678	A_33_P3354414	AOX1	3.037139833	0.0000474	down
2679	A_33_P3288942	FAM107B	3.036933813	0.007430651	down
2680	A_23_P428842	TMEM44	3.035771421	0.0000109	down
2681	A_23_P22682	ARMCX1	3.026602058	0.000000513	down
2682	A_23_P250982	ISOC1	3.024838953	0.0000444	down
2683	A_24_P930418	RBPMS	3.023707249	0.0000129	down
2684	A_23_P100660	SERPINF1	3.023401686	9.86E-08	down
2685	A_23_P86079	LOC441869	3.022108659	0.001492955	down
2686	A_23_P252764	SMARCA2	3.021232057	0.011803175	down
2687	A_23_P137035	PIR	3.019956635	0.0000416	down
2688	A_24_P53976	GLUL	3.019864881	0.0000432	down
2689	A_23_P101407	C3	3.018693399	0.00000915	down
2690	A_23_P3681	NETO2	3.01619562	0.0000292	down
2691	A_24_P520767	LOC10050580	3.015278096	0.000229544	down
2692	A_23_P93690	MCM7	3.014033948	0.00000885	down
2693	A_23_P15402	SAT2	3.012365164	0.000000144	down
2694	A_24_P923102	PHOSPHO2-K	3.011780647	0.0000244	down
2695	A_23_P202837	CCND1	3.010814866	0.000231972	down
2696	A_33_P3327106	LOC10012990	3.010091968	0.00000729	down
2697	A_23_P120002	SP110	3.003186354	0.00000179	down
2698	A_23_P151529	C14orf132	3.003025378	0.000000191	down
2699	A_23_P309950	FAM178A	3.002178796	0.001915716	down

2700	A_24_P414376	KLF3	3.001473645	4.62E-08	down
2701	A_33_P3294459	COPG2IT1	2.999862758	0.000704082	down
2702	A_23_P213562	F2R	2.996737051	0.0000016	down
2703	A_24_P323598	ESCO2	2.994927612	0.002337954	down
2704	A_24_P156049	SLC39A6	2.993218644	0.00000292	down
2705	A_23_P167401	PCDHB11	2.990830188	0.00000391	down
2706	A_32_P180315	C9orf174	2.987637683	0.0000182	down
2707	A_23_P427148	PROCA1	2.984148851	0.00000129	down
2708	A_23_P7397	PCDHB10	2.981785889	0.000000221	down
2709	A_23_P24384	CCDC88B	2.981731945	0.00000291	down
2710	A_32_P123629	TTC39C	2.980084007	0.0000189	down
2711	A_23_P7976	HIST1H1E	2.979464656	0.00000915	down
2712	A_23_P97123	ANKRD36BP1	2.978219252	0.000366628	down
2713	A_33_P3286218	DLEU2L	2.977815424	0.00000696	down
2714	A_23_P370682	BATF2	2.97713498	0.000099	down
2715	A_24_P376422	LOC10028716	2.974766299	0.000000994	down
2716	A_23_P210210	EPAS1	2.973905903	0.00000225	down
2717	A_32_P179740	ATXN7L1	2.973402975	0.00000427	down
2718	A_33_P3317392	ADAMTS19	2.973112387	0.0000826	down
2719	A_24_P298174	CBX1	2.971694068	0.00674274	down
2720	A_23_P43415	HSD17B3	2.968279063	0.000166047	down
2721	A_23_P423331	NTNG2	2.967372893	0.0000431	down
2722	A_33_P3262191	CPNE7	2.96543346	0.000000299	down
2723	A_23_P400078	MTHFR	2.965359463	0.000000338	down
2724	A_23_P39840	VAMP5	2.960713722	0.00000226	down
2725	A_24_P191781	PARM1	2.960295375	0.0000262	down
2726	A_23_P214080	EGR1	2.953192364	0.000110519	down
2727	A_23_P86532	BICC1	2.95269328	0.0000383	down
2728	A_23_P98350	BIRC3	2.952303624	0.000016	down
2729	A_23_P366216	HIST1H2BH	2.949532556	0.000074	down
2730	A_23_P165891	TAF1B	2.948692403	0.00000383	down
2731	A_24_P350576	TNIK	2.943372225	0.00000695	down
2732	A_33_P3319870	GREM1	2.942754112	0.00000257	down
2733	A_23_P60387	NOTCH1	2.941624777	0.002021261	down
2734	A_33_P3241984	PTPN22	2.941527927	0.0000378	down
2735	A_23_P361584	TMEM154	2.939458752	0.000347063	down
2736	A_23_P50096	TYMS	2.936884101	0.00000209	down
2737	A_23_P42116	PPT2	2.935354337	0.0000301	down
2738	A_33_P3294951	TAS2R30	2.935118736	0.000512766	down
2739	A_24_P148796	MST1	2.934780288	0.000011	down
2740	A_24_P140204	PXK	2.934068731	0.0000809	down
2741	A_23_P65230	TMTC4	2.933681668	0.0000381	down
2742	A_23_P87879	CD69	2.933419429	0.003045246	down
2743	A_23_P133902	PSORS1C1	2.932631905	0.00003	down
2744	A_23_P420293	C11orf45	2.932307091	0.000000257	down
2745	A_23_P121106	HESX1	2.932086638	0.000000396	down
2746	A_23_P167997	HIST1H2BG	2.931363747	0.000000904	down
2747	A_24_P4816	GABARAPL1	2.931338687	0.0000671	down
2748	A_23_P169154	ERMP1	2.929823996	0.000000263	down
2749	A_24_P260443	THBS4	2.928190877	0.0000103	down
2750	A_33_P3311076	CYB5A	2.924456102	0.00000615	down

2751	A_33_P3871347	SNED1	2.921558816	0.00000754	down
2752	A_33_P3284919	SEMA6C	2.916948133	0.000128596	down
2753	A_33_P3271651	HLA-DPB1	2.916170152	0.000065	down
2754	A_33_P3870906	LOC10050763	2.91570609	0.002535855	down
2755	A_23_P91943	IL12A	2.913332366	0.00000129	down
2756	A_23_P422732	WDR63	2.912254829	0.0000213	down
2757	A_23_P118042	LRRC36	2.910030754	4.59E-09	down
2758	A_23_P59637	DOCK4	2.909752612	0.000320937	down
2759	A_23_P32021	FANCC	2.90940472	0.00000632	down
2760	A_23_P105409	MAP3K12	2.907134863	0.000376098	down
2761	A_23_P323094	PHC1	2.906934706	0.00000264	down
2762	A_24_P361457	ENDOV	2.905179893	0.0000112	down
2763	A_24_P362881	IFT57	2.904815902	0.000225827	down
2764	A_33_P3402056	LOC389831	2.904206759	0.00000263	down
2765	A_24_P942030	VAMP4	2.903601232	0.000101028	down
2766	A_32_P155247	FTL	2.902287286	0.000000872	down
2767	A_32_P40463	NUDT9P1	2.898294641	0.0000139	down
2768	A_23_P37127	FOXA1	2.896791202	0.0000164	down
2769	A_24_P226962	KIAA0368	2.892645777	0.00000398	down
2770	A_33_P3261054	CCDC114	2.891934146	0.000000904	down
2771	A_32_P56397	LOC731789	2.891584977	0.0000895	down
2772	A_23_P145424	KIAA1009	2.889158595	0.0000217	down
2773	A_33_P3229083	HIST1H2BK	2.888383554	2.26E-08	down
2774	A_24_P216421	DTNB	2.887525059	0.016228068	down
2775	A_23_P120883	HMOX1	2.88178459	0.000210527	down
2776	A_23_P434890	CARD10	2.880588003	0.000000141	down
2777	A_33_P3369393	NCF1	2.879383996	0.000451586	down
2778	A_33_P3308481	HYMAI	2.877449615	0.000153602	down
2779	A_24_P251534	CTDSPL	2.87595679	0.000152417	down
2780	A_23_P14673	IGDCC4	2.875113347	0.00000702	down
2781	A_33_P3369190	PAM	2.874693877	0.000000647	down
2782	A_24_P140666	N4BP3	2.874482604	0.001159039	down
2783	A_33_P3302916	BACH2	2.873733545	0.000145885	down
2784	A_24_P211565	C1QTNF6	2.872478975	0.00000158	down
2785	A_33_P3653912	FAHD2A	2.871501868	4.93E-09	down
2786	A_33_P3274084	RHBDL3	2.871214074	0.030046043	down
2787	A_24_P231057	BOD1L	2.870035536	0.007955023	down
2788	A_23_P138717	RGS10	2.869991903	0.00000721	down
2789	A_23_P119344	TEAD2	2.869258267	0.000473971	down
2790	A_23_P320739	MEF2C	2.868941532	0.000017	down
2791	A_23_P147665	OLFML1	2.867260532	0.00000381	down
2792	A_23_P40217	DOK5	2.864194701	0.000038	down
2793	A_23_P66948	FAM59A	2.859523113	0.0000423	down
2794	A_23_P315892	ST6GALNAC6	2.859085968	0.001910459	down
2795	A_32_P179676	TOB2	2.856992007	0.00000553	down
2796	A_23_P144622	GNPDA1	2.856248495	0.0000196	down
2797	A_23_P324523	IQCK	2.85570971	0.00000975	down
2798	A_33_P3255647	RPL36A-HNRN	2.84366628	0.025592786	down
2799	A_23_P119943	IGFBP2	2.836635277	0.000000288	down
2800	A_33_P3232319	LRRD1	2.836560955	0.0000025	down
2801	A_33_P3883985	LMF1	2.833872793	0.00000837	down

2802	A_24_P93309	CDNF	2.832878578	0.00000259	down
2803	A_23_P250294	ABHD5	2.831204579	0.00000386	down
2804	A_24_P205252	FAM13C	2.829090968	0.00000757	down
2805	A_23_P83579	ARNT2	2.827650214	0.000000322	down
2806	A_23_P384748	PLEKHH2	2.827011333	0.000052	down
2807	A_33_P3272209	MFSD6	2.826414064	0.0000617	down
2808	A_33_P3304963	LRRC27	2.825754048	0.003294836	down
2809	A_24_P349196	CCDC30	2.823800826	0.00000122	down
2810	A_33_P3335740	FLJ10038	2.822924675	0.0000868	down
2811	A_33_P3274129	TMEM151B	2.822895716	0.0000346	down
2812	A_23_P82975	VPS13B	2.822399154	0.023544914	down
2813	A_33_P3244863	CEP350	2.821932214	0.000695822	down
2814	A_23_P62967	DISC1	2.821676314	0.000038	down
2815	A_33_P3234015	LIMD1	2.821044323	0.00000127	down
2816	A_24_P186342	ZFP14	2.819361358	0.00000013	down
2817	A_33_P3556116	LOC145678	2.819064851	0.000197395	down
2818	A_23_P4443	TBX4	2.818784983	0.000513204	down
2819	A_23_P31896	ST3GAL1	2.816792583	0.000000116	down
2820	A_33_P3296119	C13orf38	2.814845815	0.000185553	down
2821	A_32_P104478	FGD6	2.813905736	0.0000981	down
2822	A_23_P216094	ASPH	2.812759105	0.020142373	down
2823	A_23_P320622	TTTY10	2.810787329	0.000632391	down
2824	A_23_P167983	HIST1H2AC	2.807663426	0.000051	down
2825	A_33_P3263307	RANGAP1	2.804397136	0.000000965	down
2826	A_24_P239076	IGLL1	2.803842672	0.000435035	down
2827	A_33_P3234020	IGDCC3	2.802448575	0.000101464	down
2828	A_33_P3263417	FLJ43663	2.799433287	0.000018	down
2829	A_33_P3212072	GORAB	2.797733802	0.016306904	down
2830	A_23_P415061	FAM104B	2.792123908	0.00000128	down
2831	A_32_P56392	RBMX	2.792095717	0.008389484	down
2832	A_23_P142738	TMEM178	2.789165097	0.0000876	down
2833	A_23_P214139	REV3L	2.78878974	0.0000304	down
2834	A_32_P160896	FTMT	2.788440847	0.0000572	down
2835	A_23_P205046	ANKRD10	2.787905513	0.0000249	down
2836	A_23_P349966	TMEM130	2.786396051	0.000000034	down
2837	A_23_P129425	TSNAXIP1	2.783418421	0.000468516	down
2838	A_23_P81399	SQSTM1	2.782222565	0.00040491	down
2839	A_23_P111860	RADIL	2.781898726	0.000143834	down
2840	A_33_P3260125	LDLRAD2	2.781883621	0.002851963	down
2841	A_24_P202497	TWSG1	2.781812148	0.0000342	down
2842	A_23_P34375	TCEA3	2.779113007	0.000359353	down
2843	A_33_P3329522	LRRC17	2.778751522	0.000000828	down
2844	A_24_P380311	CAMK2A	2.778615672	0.00000436	down
2845	A_23_P432573	MRGPRF	2.777905137	0.000164666	down
2846	A_23_P133236	PCDHB14	2.776840541	0.0000134	down
2847	A_33_P3396537	CDHR4	2.77683143	0.0000265	down
2848	A_32_P175715	MEIG1	2.775968185	0.00000673	down
2849	A_33_P3271657	HHIPL1	2.773514305	0.0000141	down
2850	A_23_P168443	EPHB4	2.772705583	0.037462185	down
2851	A_23_P41854	CARD6	2.770608118	0.0000647	down
2852	A_23_P48198	GLT8D2	2.77013253	0.000000987	down

2853	A_24_P365349	CACNG7	2.770005358	0.0000699	down
2854	A_23_P339818	ARRDC4	2.769697019	0.00000469	down
2855	A_32_P170444	SUB1	2.769104822	0.00000869	down
2856	A_24_P381555	SAP18	2.767503548	0.0000852	down
2857	A_33_P3303259	PARP4	2.767214158	0.002822077	down
2858	A_23_P502343	ADAM33	2.766038365	0.000108407	down
2859	A_33_P3235701	ZCCHC11	2.765232653	0.001667701	down
2860	A_33_P3274462	LOC643714	2.764753388	0.000310183	down
2861	A_23_P70785	AIM1	2.761849042	0.00014703	down
2862	A_33_P3362143	TTC28-AS1	2.76083455	0.00000621	down
2863	A_23_P8013	HIST1H2BL	2.760792896	0.000264623	down
2864	A_23_P418015	MAPRE2	2.760276263	0.0000431	down
2865	A_23_P67367	DHDH	2.758435793	0.00000513	down
2866	A_32_P415151	WDR27	2.758362501	0.00000384	down
2867	A_33_P3307735	OPHN1	2.75793961	0.000057	down
2868	A_23_P256205	ABLIM3	2.754548121	0.00000689	down
2869	A_24_P932594	ULK2	2.754319078	0.001973511	down
2870	A_24_P706340	FAM155A	2.751571212	0.0000241	down
2871	A_23_P393686	C8orf42	2.750668788	0.003253982	down
2872	A_33_P3366082	EBPL	2.750340679	0.0000202	down
2873	A_24_P277657	GMPR	2.749810372	0.00000197	down
2874	A_33_P3222917	CD276	2.747711117	0.0000418	down
2875	A_33_P3288844	IL6R	2.743865314	0.000260445	down
2876	A_23_P144348	SLIT2	2.742198739	0.000443163	down
2877	A_23_P93180	HIST1H2BC	2.740767597	0.00000105	down
2878	A_23_P257834	ALB	2.740034516	0.000831522	down
2879	A_24_P177948	DPY19L2	2.739263277	0.000151816	down
2880	A_23_P258190	AKR1B1	2.73841481	0.00000858	down
2881	A_24_P331560	STS	2.738104674	0.0000257	down
2882	A_33_P3297003	LOC647979	2.737990992	0.001492349	down
2883	A_23_P380881	ANKRD13B	2.737784326	0.003565887	down
2884	A_24_P262201	SULT1A4	2.73727301	0.000288442	down
2885	A_24_P299911	PASK	2.736810922	0.00000506	down
2886	A_23_P41765	IRF1	2.732551526	0.000163246	down
2887	A_33_P3336422	LOC729013	2.730897817	0.00000974	down
2888	A_33_P3807062	HJURP	2.730260863	0.00000855	down
2889	A_23_P38206	LSMD1	2.729950767	0.003998795	down
2890	A_24_P31929	PRRG1	2.727271511	0.00000692	down
2891	A_23_P255126	GAB3	2.726878588	0.0000111	down
2892	A_23_P59069	HIST1H2BO	2.726383797	0.0000266	down
2893	A_24_P67534	LOC644538	2.726096502	0.00000527	down
2894	A_32_P41496	LOC10013283	2.72604246	0.010513302	down
2895	A_33_P3250963	TP53TG1	2.725477481	0.0000588	down
2896	A_33_P3338011	ZNF480	2.724794573	0.000146803	down
2897	A_33_P3434927	MAGI2-IT1	2.724714998	0.000124782	down
2898	A_33_P3396444	PTGR1	2.723380001	0.000190754	down
2899	A_24_P213715	LOC90834	2.722363097	0.000278797	down
2900	A_24_P943781	C7orf58	2.718159283	0.00000548	down
2901	A_24_P312041	PLAGL1	2.717910595	0.000200099	down
2902	A_23_P258124	ZNF346	2.717717564	0.000166946	down
2903	A_33_P3309481	PTPRVP	2.717369904	0.000145172	down

2904	A_33_P3332970	CLEC2B	2.715495113	0.004008877	down
2905	A_23_P134113	C6orf192	2.714521982	0.0000374	down
2906	A_33_P3266429	SAMD13	2.713198249	0.000156961	down
2907	A_23_P345928	C12orf26	2.712149427	0.0000964	down
2908	A_23_P53884	MAB21L1	2.706517407	0.000017	down
2909	A_33_P3279049	UPF2	2.706194501	0.000952176	down
2910	A_23_P35995	CLMP	2.706139104	0.000520596	down
2911	A_23_P154688	SLC4A11	2.705404471	0.0000985	down
2912	A_23_P356554	BAG2	2.704992324	0.00000214	down
2913	A_33_P3382856	DCN	2.702613736	0.0000351	down
2914	A_32_P394951	NTN5	2.702427348	0.000000468	down
2915	A_23_P132159	USP18	2.701709454	9.28E-08	down
2916	A_23_P134714	HRSP12	2.701541167	0.00000153	down
2917	A_23_P354734	PCDHGA8	2.700419733	0.0000043	down
2918	A_33_P3237944	AP4M1	2.700080025	0.009091357	down
2919	A_33_P3351510	IL1RAPL1	2.699778597	0.00000474	down
2920	A_23_P164436	ASPA	2.696066285	0.00000322	down
2921	A_23_P104073	S100A3	2.695730425	0.0000243	down
2922	A_23_P147070	SUN1	2.690614868	0.000000146	down
2923	A_23_P168882	TP53INP1	2.690415508	0.0000374	down
2924	A_33_P3233005	MBTD1	2.690152452	0.000073	down
2925	A_33_P3306504	ISYNA1	2.689925096	0.000164484	down
2926	A_33_P3255544	RNF130	2.689449066	0.0000247	down
2927	A_23_P106773	SULT1A2	2.689440988	0.000000581	down
2928	A_23_P111054	HIST1H2BB	2.689285644	0.000221955	down
2929	A_33_P3324428	AKAP1	2.686146208	0.000696917	down
2930	A_33_P3388958	SNX32	2.685332993	0.000000636	down
2931	A_24_P468810	RBBP4	2.68226975	0.000000996	down
2932	A_33_P3243332	ARHGAP42	2.680775611	0.0000361	down
2933	A_33_P3210492	C1QTNF4	2.679114487	0.000164363	down
2934	A_32_P232559	LOC439949	2.678949898	0.003540042	down
2935	A_23_P70968	HOXA7	2.678564618	0.0000204	down
2936	A_32_P59302	HIVEP3	2.676828232	0.000395642	down
2937	A_24_P369898	MYO15B	2.676713012	0.0000642	down
2938	A_24_P220771	ANKRD17	2.676709796	0.027282766	down
2939	A_24_P122921	BCL2L11	2.673190527	0.0000256	down
2940	A_23_P129856	HIC1	2.672986714	0.00143063	down
2941	A_24_P210637	CHST14	2.669649669	0.007079888	down
2942	A_32_P46594	LOC145837	2.668562252	0.00308509	down
2943	A_23_P169197	HSDL2	2.666989848	3.89E-08	down
2944	A_23_P257763	BOC	2.664447549	0.000155197	down
2945	A_24_P475349	RAB6B	2.663314253	0.000262251	down
2946	A_33_P3404411	MIPEP	2.663004131	7.11E-08	down
2947	A_24_P181295	C14orf37	2.662912086	0.0000261	down
2948	A_33_P3368375	RANBP3	2.662340199	0.032427809	down
2949	A_33_P3334220	ACACB	2.658973072	0.0000303	down
2950	A_24_P38276	FZD1	2.656612853	0.00000177	down
2951	A_23_P113471	FAAH2	2.655697762	0.0000134	down
2952	A_23_P102611	WISP2	2.653214533	0.000287529	down
2953	A_24_P802145	IRS1	2.653194303	0.000514492	down
2954	A_33_P3423445	ZNF730	2.652659867	0.000000443	down

2955	A_24_P314179	ETS2	2.652275548	0.0000524	down
2956	A_33_P3305487	REM2	2.649352361	0.0000253	down
2957	A_32_P37592	SCARNA17	2.648940184	0.000541637	down
2958	A_23_P3956	C1QTNF1	2.648816556	0.004921824	down
2959	A_23_P256956	KIF20A	2.646577559	0.0000365	down
2960	A_33_P3462155	LOC401188	2.64627281	0.013596119	down
2961	A_24_P70117	DTWD1	2.646096483	0.0000769	down
2962	A_23_P310086	BEND6	2.645026482	0.0000731	down
2963	A_24_P230540	RNF212	2.642769928	0.000624147	down
2964	A_23_P125383	TMEFF2	2.64236543	0.000236999	down
2965	A_33_P3323928	NCRNA00263	2.642197421	0.00003	down
2966	A_23_P98645	DCHS1	2.64200586	0.000847212	down
2967	A_23_P214876	JARID2	2.641819074	0.000000113	down
2968	A_23_P317324	MECOM	2.639544521	0.000103207	down
2969	A_23_P78037	CCL7	2.637708432	0.006067014	down
2970	A_23_P42331	HMGA1	2.636813133	0.007523224	down
2971	A_33_P3347452	RPS6KA2	2.633895912	0.0000107	down
2972	A_23_P106898	ORAI3	2.63340485	0.000162529	down
2973	A_23_P72668	SDPR	2.63143088	0.0000312	down
2974	A_23_P118254	FOXF1	2.629808352	0.000000909	down
2975	A_23_P321913	DEF6	2.629670245	0.0000977	down
2976	A_33_P3330498	ALDH7A1	2.62944958	0.0000919	down
2977	A_33_P3232993	MARK2	2.629119104	0.029120889	down
2978	A_23_P423427	FAM171B	2.628453903	0.00000938	down
2979	A_23_P315345	PIN4	2.626678388	0.000178759	down
2980	A_32_P218355	C6orf132	2.622197649	0.000288529	down
2981	A_24_P941038	VSTM4	2.620828408	0.000663029	down
2982	A_24_P248185	C12orf53	2.620603944	0.00000239	down
2983	A_23_P118815	BIRC5	2.619728252	0.000000911	down
2984	A_23_P207319	MAP3K14	2.619335148	0.00000442	down
2985	A_33_P3400374	PRIC285	2.617886712	0.0000615	down
2986	A_23_P211973	NEK11	2.616854842	0.0000183	down
2987	A_33_P3715177	MEGF8	2.616430614	0.000327335	down
2988	A_33_P3218004	AKR7L	2.616174913	0.000039	down
2989	A_33_P3216227	ITGB1BP1	2.612515929	0.0000986	down
2990	A_24_P169634	MBL1P	2.611832298	0.0000103	down
2991	A_23_P118722	ASGR1	2.610338676	0.0000613	down
2992	A_24_P318593	SCRN2	2.609639336	0.011281546	down
2993	A_23_P6909	CCRL1	2.609063457	0.000263914	down
2994	A_33_P3812038	LOC340335	2.607819168	0.000312636	down
2995	A_24_P406814	FAM53B	2.606900164	0.00000429	down
2996	A_24_P297539	UBE2C	2.605631742	0.00000612	down
2997	A_32_P846696	LOC349196	2.604104181	0.000434283	down
2998	A_23_P110957	FOXF2	2.603006053	2.24E-08	down
2999	A_23_P75741	UBE2L6	2.601770424	0.0001475	down
3000	A_23_P165989	NEURL2	2.599838788	0.00000341	down
3001	A_23_P256158	ADRA2C	2.597876953	0.00114486	down
3002	A_32_P135348	TANC1	2.59776213	0.00000103	down
3003	A_33_P3234667	ZKSCAN1	2.595197797	0.000232156	down
3004	A_23_P124733	COQ2	2.594581463	0.000000974	down
3005	A_24_P397386	LIFR	2.594276647	0.0000282	down

3006	A_23_P316812	AKR7A2P1	2.593557041	0.000648975	down
3007	A_33_P3310293	PKIG	2.593434021	0.014425796	down
3008	A_33_P3357630	C12orf71	2.592076685	0.048582678	down
3009	A_23_P210726	CDC25B	2.58957977	0.000733503	down
3010	A_23_P37778	FHOD1	2.584530689	0.000536722	down
3011	A_23_P332584	KIAA1107	2.584446074	0.000019	down
3012	A_23_P76823	ADSSL1	2.583935695	0.0000844	down
3013	A_33_P3330952	ATP8A1	2.582023265	0.0000138	down
3014	A_33_P3361546	TFAP2A	2.581652998	0.000106384	down
3015	A_23_P7684	CCNJL	2.581079479	0.00000461	down
3016	A_24_P49106	TCEAL7	2.579567146	0.0000076	down
3017	A_33_P3214491	PDE4DIP	2.577828942	0.00225113	down
3018	A_24_P63537	ERAP1	2.57762162	0.000190722	down
3019	A_33_P3268716	SNX1	2.574432005	0.00000312	down
3020	A_23_P149529	TACSTD2	2.574147756	0.000819004	down
3021	A_23_P326319	C16orf45	2.574133125	0.00000787	down
3022	A_32_P8546	C6orf176	2.57266552	0.000568485	down
3023	A_33_P3272169	CLIP2	2.569676593	0.001088121	down
3024	A_23_P17855	TRIOBP	2.568386522	0.0000279	down
3025	A_23_P43273	EXT1	2.56796938	0.0000132	down
3026	A_24_P405430	TIA1	2.567774183	0.00000182	down
3027	A_23_P100141	UNKL	2.567115724	0.000357902	down
3028	A_24_P132518	IKBKB	2.566388947	0.000000762	down
3029	A_24_P119685	OBSCN	2.565980073	0.000003	down
3030	A_24_P462853	NCRNA00202	2.565388993	0.009323814	down
3031	A_23_P366983	TRHDE	2.564869043	0.010655366	down
3032	A_33_P3213374	CITED2	2.562839859	0.015229776	down
3033	A_23_P146077	ZNF395	2.562175562	0.00000124	down
3034	A_23_P154605	SULF2	2.561474448	0.0000563	down
3035	A_24_P225616	RRM2	2.559899195	0.0000687	down
3036	A_23_P214658	PBX2	2.559282433	0.000249877	down
3037	A_32_P116271	NCRNA00247	2.558836676	0.00000703	down
3038	A_23_P81392	WWC1	2.557642283	0.00000413	down
3039	A_23_P259741	SATB1	2.557071793	0.0000235	down
3040	A_23_P126757	INTS3	2.55683489	0.00000247	down
3041	A_23_P163143	ACYP1	2.556315669	0.0000575	down
3042	A_24_P350546	LOC646976	2.556168606	0.00000491	down
3043	A_32_P351968	HLA-DMB	2.556035724	0.00000639	down
3044	A_23_P121851	PCDHB15	2.55597401	0.000000495	down
3045	A_23_P42353	ETV7	2.553412107	0.0000181	down
3046	A_24_P65199	CDK10	2.552455661	0.042155544	down
3047	A_33_P3290709	EGFL6	2.551584524	0.00000265	down
3048	A_33_P3306828	ZNF806	2.551446457	0.023134716	down
3049	A_24_P58122	USP24	2.551436377	0.000101202	down
3050	A_23_P23783	MYOC	2.551013558	0.013611065	down
3051	A_23_P106002	NFKBIA	2.550643613	0.0000378	down
3052	A_33_P3328410	LOC10029185	2.549911896	0.0000149	down
3053	A_23_P36753	ALDH2	2.547624527	7.98E-08	down
3054	A_24_P7202	KIDINS220	2.547435585	0.000407652	down
3055	A_33_P3251841	DSEL	2.546910918	0.000728642	down
3056	A_24_P854492	MIAT	2.546002201	0.0000371	down

3057	A_23_P312999	C4orf22	2.545582342	0.0000151	down
3058	A_32_P25050	RDH10	2.545191132	0.000000238	down
3059	A_23_P20852	AUH	2.544583734	0.0000228	down
3060	A_23_P380371	IPMK	2.544069235	0.006317174	down
3061	A_23_P66432	TTYH2	2.542168632	0.0000266	down
3062	A_33_P3221064	LTBP4	2.542080118	0.00000307	down
3063	A_33_P3357979	CTBP1	2.540758054	0.019003891	down
3064	A_24_P30557	TBX5	2.540647693	0.0000482	down
3065	A_24_P161018	PARP14	2.539322736	0.001559259	down
3066	A_23_P160025	IFI16	2.538224187	0.000000177	down
3067	A_33_P3323298	JUN	2.536341781	0.0000346	down
3068	A_33_P3381870	EPB49	2.535488094	0.0000149	down
3069	A_24_P264207	PTMA	2.535158591	0.0000806	down
3070	A_23_P93311	DDR1	2.533775729	0.002959958	down
3071	A_23_P14975	C16orf48	2.532700821	0.000481696	down
3072	A_23_P68851	KREMEN1	2.531991099	0.000000261	down
3073	A_24_P287473	SAV1	2.531332459	1.11E-08	down
3074	A_23_P4662	BCL3	2.531287425	0.00000953	down
3075	A_23_P146325	ASAP1-IT1	2.530715505	0.00568355	down
3076	A_23_P219084	ZNF3	2.530337804	8.47E-09	down
3077	A_33_P3280157	SNORD116-19	2.530221641	0.000968487	down
3078	A_24_P208909	TRIM2	2.529586664	0.002121252	down
3079	A_23_P94533	CTSL1	2.527937859	0.0000785	down
3080	A_33_P3440636	LOC286071	2.527834888	0.0000649	down
3081	A_33_P3376873	LOC10050970[illegible]	2.527283193	0.0000367	down
3082	A_23_P366682	C19orf20	2.52641516	0.028045568	down
3083	A_32_P118568	RFPL1-AS1	2.525550927	0.000066	down
3084	A_23_P68155	IFIH1	2.524891511	0.0000142	down
3085	A_32_P221991	RALGPS1	2.521785383	0.00000532	down
3086	A_33_P3270657	FAM111B	2.521268037	0.0000892	down
3087	A_23_P128808	GPR132	2.520361482	0.00000216	down
3088	A_23_P302207	ZNF853	2.519766414	0.040952239	down
3089	A_33_P3403053	LOC441242	2.518079619	0.000599365	down
3090	A_24_P922252	SPATA5L1	2.517651567	0.001093766	down
3091	A_23_P120048	BAZ2B	2.517116517	0.000013	down
3092	A_33_P3319401	ARHGAP21	2.516815685	0.00000203	down
3093	A_23_P105144	SCUBE2	2.514591005	0.0000509	down
3094	A_23_P18684	CLGN	2.51395298	0.0000017	down
3095	A_33_P3405384	C8orf45	2.513664258	0.002012015	down
3096	A_23_P112452	GGTA1P	2.513250487	0.0000412	down
3097	A_23_P68807	LOC10050987[illegible]	2.512420129	0.000151059	down
3098	A_32_P29118	SEMA3D	2.507740385	0.00000385	down
3099	A_33_P3406004	YBEY	2.504780184	0.01046039	down
3100	A_23_P113613	CDCP1	2.504433955	0.000262714	down
3101	A_32_P172141	CDON	2.501912093	0.0000644	down
3102	A_24_P236235	FLRT2	2.501902671	0.00045972	down
3103	A_24_P256380	WLS	2.501328721	0.0000848	down
3104	A_33_P3319815	CXorf28	2.49964803	0.00030885	down
3105	A_33_P3497352	GRIA4	2.497361955	0.0000389	down
3106	A_23_P24433	CTSF	2.496644369	0.00000466	down
3107	A_24_P54485	CCDC115	2.496186567	0.003429123	down

3108	A_33_P3398840	ZBED1	2.495882009	0.018521591	down
3109	A_24_P734953	TRNP1	2.495862863	0.000512766	down
3110	A_33_P3353030	UCN	2.493541706	0.0000604	down
3111	A_33_P3251492	LOC10013366	2.493242424	0.00008	down
3112	A_33_P3361584	LOC10013093	2.492904415	0.002004451	down
3113	A_33_P3366484	HIBCH	2.492485019	0.000416684	down
3114	A_23_P357860	SEC62	2.491890199	0.000074	down
3115	A_23_P17190	KBTBD10	2.491518351	0.0000158	down
3116	A_23_P67864	ADCY3	2.49077177	0.0000089	down
3117	A_23_P142849	RND3	2.48891478	0.0000751	down
3118	A_24_P360529	PDE7A	2.48688995	0.000271877	down
3119	A_23_P142872	TCF7L1	2.484279793	0.0000058	down
3120	A_23_P138507	CDK1	2.483082737	0.0000639	down
3121	A_32_P39003	LOC10013305	2.481854146	0.003144229	down
3122	A_23_P61180	PLCXD1	2.477425487	0.00000275	down
3123	A_33_P3321050	PHACTR2	2.476096086	0.000765003	down
3124	A_32_P133916	BNC2	2.47509174	4.12E-08	down
3125	A_23_P145965	TPST1	2.475043989	0.00000784	down
3126	A_23_P337262	APCDD1	2.47297302	0.000000209	down
3127	A_23_P24157	PYROXD2	2.472069836	0.00000574	down
3128	A_23_P254801	PLCG1	2.471935729	0.006349425	down
3129	A_23_P131801	SGK2	2.471818934	0.004031797	down
3130	A_23_P96641	PRPS2	2.470772251	0.006356672	down
3131	A_24_P262407	THRA	2.469899489	0.015119187	down
3132	A_24_P253827	AP2B1	2.46982245	0.000855707	down
3133	A_24_P456490	C1orf204	2.469438546	0.001782033	down
3134	A_33_P3296858	ZNF37BP	2.466674241	0.000247101	down
3135	A_33_P3270019	NCOA2	2.466536323	0.000133283	down
3136	A_23_P325690	ANKRD35	2.465930262	0.0000118	down
3137	A_23_P217564	ACSL4	2.465457414	0.00000167	down
3138	A_23_P65174	PHF11	2.464765397	0.0000392	down
3139	A_33_P3317576	DHFRL1	2.464466209	0.00030101	down
3140	A_33_P3403937	LOC10012939	2.462520611	0.001332546	down
3141	A_24_P88801	NPHP1	2.462422524	0.000202136	down
3142	A_24_P849801	RPL22	2.460511626	0.0000269	down
3143	A_33_P3283984	AFG3L1P	2.45903687	0.049995957	down
3144	A_23_P251421	CDCA7	2.457532791	0.0000107	down
3145	A_23_P75800	RAB3IL1	2.456382108	0.000000295	down
3146	A_24_P335092	SAA1	2.455661546	0.003634457	down
3147	A_23_P88033	FGF14	2.455427683	0.000136261	down
3148	A_33_P3269636	SBSN	2.455340884	0.000000121	down
3149	A_23_P148879	ATP1A2	2.454294998	0.0000124	down
3150	A_23_P61580	NSUN6	2.454234323	0.00000395	down
3151	A_33_P3232277	CCNG1	2.453724033	0.001286106	down
3152	A_33_P3257643	C17orf104	2.453038596	0.039005199	down
3153	A_23_P88559	LIPC	2.453035875	0.00000223	down
3154	A_32_P180958	PCYOX1	2.449416311	0.00000856	down
3155	A_24_P408047	PLEKHA4	2.44858283	0.0000211	down
3156	A_24_P710730	LOC10017093	2.448386525	0.000721879	down
3157	A_32_P36235	IER2	2.446769165	0.00000212	down
3158	A_23_P106973	9-Sep	2.445821866	0.000000305	down

3159	A_23_P324989	RECQL5	2.445696529	0.008229885	down
3160	A_33_P3258782	AP1S2	2.443767443	0.0000458	down
3161	A_33_P3389261	TMEM194B	2.443305055	0.000101879	down
3162	A_32_P36143	LOC729088	2.442656955	0.00000647	down
3163	A_33_P3775848	CLIC2	2.442171755	0.000000937	down
3164	A_23_P170608	TSPYL2	2.440996117	0.000889603	down
3165	A_33_P3337277	LOC10012984(	2.43931206	0.0000169	down
3166	A_33_P3326545	LRP5L	2.438749032	0.0000281	down
3167	A_23_P93032	ZBED3	2.436883587	0.00000309	down
3168	A_33_P3397795	C14orf135	2.430549754	0.001262936	down
3169	A_24_P678418	FLJ45244	2.428144029	0.000644775	down
3170	A_23_P161563	RAB38	2.42752104	0.00000169	down
3171	A_24_P876408	C11orf95	2.427390135	0.00000715	down
3172	A_24_P873659	MALAT1	2.425619629	0.007073799	down
3173	A_33_P3240328	PITX1	2.425427406	0.000000434	down
3174	A_23_P66608	KAT2A	2.425394904	0.00000134	down
3175	A_23_P129118	PDCD7	2.424071635	0.000012	down
3176	A_23_P202004	PRTFDC1	2.423285411	0.000120046	down
3177	A_24_P287043	IFITM2	2.422461102	0.000000623	down
3178	A_23_P93641	AKR1B10	2.422445822	0.00000269	down
3179	A_23_P31945	IL33	2.421874096	0.0000248	down
3180	A_33_P3383233	NDRG2	2.421657775	0.00044192	down
3181	A_23_P167030	PTH1R	2.421329526	0.0000402	down
3182	A_33_P3232173	PSPC1	2.420866405	0.0000114	down
3183	A_24_P924591	VEZF1	2.420415116	0.0000031	down
3184	A_23_P501547	ADCY6	2.419659988	0.020665175	down
3185	A_24_P379693	DNPEP	2.419159401	0.000615308	down
3186	A_24_P58054	SLC9A8	2.418305205	0.00000678	down
3187	A_23_P211064	GCFC1	2.418020094	0.000632521	down
3188	A_32_P85676	STK32B	2.417074599	0.0000066	down
3189	A_24_P190168	TMEM97	2.415249673	0.0000338	down
3190	A_24_P110141	DNM1P46	2.414828333	0.00008	down
3191	A_33_P3244424	SNRPE	2.414454874	0.000516279	down
3192	A_24_P250765	PRKG1	2.414046501	0.0000784	down
3193	A_23_P64879	KCNJ8	2.413554939	0.00000925	down
3194	A_33_P3296479	APP	2.412829155	0.046312907	down
3195	A_23_P127475	CCS	2.411483045	0.0000044	down
3196	A_33_P3333507	SENP7	2.410890845	0.000000943	down
3197	A_33_P3335624	PSMD10	2.410742122	0.001227538	down
3198	A_23_P215744	CTTNBP2	2.410693106	0.00000597	down
3199	A_23_P417383	ASPRV1	2.40969402	0.0000141	down
3200	A_23_P327370	PGPEP1	2.409650371	0.000292858	down
3201	A_33_P3383955	DDB2	2.409532343	0.000673882	down
3202	A_33_P3214785	LEPROT	2.409520485	0.0000161	down
3203	A_33_P3325502	ARHGAP29	2.408216783	0.000107542	down
3204	A_32_P189204	GAS2L3	2.405828717	0.004562778	down
3205	A_33_P3350343	MLL	2.403820106	0.000437225	down
3206	A_33_P3314531	LOC646853	2.402947786	0.000872193	down
3207	A_33_P3713357	ALCAM	2.400625457	0.00000659	down
3208	A_24_P743802	ZNF618	2.399480353	0.000113878	down
3209	A_33_P3211634	PPIL6	2.39929386	0.000214507	down

3210	A_24_P100742	ADD1	2.39922041	0.026876508	down
3211	A_33_P3234697	LXN	2.399139976	0.00000242	down
3212	A_33_P3306153	KIAA1841	2.398426563	0.000106779	down
3213	A_23_P255331	C4orf49	2.398370151	0.0000936	down
3214	A_32_P110016	LOC727869	2.397206958	0.009246075	down
3215	A_23_P416751	ZNF610	2.396933693	0.0000209	down
3216	A_32_P145010	LOC729683	2.396359185	0.00000128	down
3217	A_23_P43580	CNTRL	2.39579821	0.0000444	down
3218	A_23_P11081	AKAP4	2.395612226	0.012180379	down
3219	A_33_P3576317	LOC340178	2.395080921	0.044792202	down
3220	A_33_P3299882	UQCRB	2.395008872	0.000000169	down
3221	A_23_P408271	HSD17B11	2.39498065	0.000103424	down
3222	A_23_P69100	ARHGEF26	2.394879056	0.000288095	down
3223	A_33_P3257182	MYBPC2	2.39431107	0.000248543	down
3224	A_33_P3236906	NCRNA00271	2.39397198	0.015686138	down
3225	A_23_P162120	NUMA1	2.393619223	0.027493996	down
3226	A_24_P46911	KCNS2	2.392126914	0.000000612	down
3227	A_33_P3287631	CTSB	2.391239002	0.00000365	down
3228	A_23_P6223	SCAF4	2.389956456	0.002056716	down
3229	A_33_P3396344	LOC728613	2.388970049	0.00039013	down
3230	A_23_P71537	CSPP1	2.387467896	0.000000369	down
3231	A_24_P334640	PAQR8	2.387429448	0.00000338	down
3232	A_23_P96041	TMEM164	2.386389166	0.0000662	down
3233	A_23_P79794	TGIF2	2.385389571	0.0000336	down
3234	A_24_P32085	MOBKL2B	2.38074142	0.000446978	down
3235	A_24_P388528	ST6GAL1	2.37971329	0.000247055	down
3236	A_33_P3220911	BST2	2.378562773	0.001002842	down
3237	A_24_P374382	TOP1P2	2.378386204	0.000608026	down
3238	A_33_P3821494	LOC284939	2.377469061	0.002394123	down
3239	A_33_P3350575	OC90	2.377427973	0.019772918	down
3240	A_33_P3269359	SPPL3	2.376429056	0.0000097	down
3241	A_23_P369994	DCLK1	2.375618929	0.024516765	down
3242	A_33_P3298440	LOC729324	2.374461394	0.000000793	down
3243	A_23_P164387	SMARCE1	2.373207642	0.00000163	down
3244	A_23_P109881	ITIH4	2.37306201	0.00000466	down
3245	A_24_P84008	LOC10050902	2.372897583	0.00000741	down
3246	A_23_P122579	DAXX	2.372666778	0.000122015	down
3247	A_33_P3330079	LOC10012824	2.372277696	0.011320188	down
3248	A_24_P84822	GUSBP1	2.372249907	0.001295521	down
3249	A_23_P113111	AR	2.37157764	0.003387591	down
3250	A_23_P162449	SRGAP1	2.370911809	0.000196388	down
3251	A_23_P50368	OSCAR	2.370416653	0.000000365	down
3252	A_24_P350759	SLC1A2	2.369818275	0.00000965	down
3253	A_33_P3299279	C5orf39	2.368508474	0.0000776	down
3254	A_23_P13929	NRIP2	2.368463819	0.000618217	down
3255	A_33_P3334630	PLP1	2.367828569	0.000672735	down
3256	A_24_P62530	RHOU	2.366258027	0.0000081	down
3257	A_24_P55148	HIST1H2BJ	2.365666493	0.00000297	down
3258	A_23_P9415	ACO1	2.365371848	0.00000256	down
3259	A_33_P3353816	ITGA4	2.363831172	0.0000102	down
3260	A_33_P3384322	LOC10013200	2.362443784	0.003104991	down

3261	A_33_P3362611	ELTD1	2.362430684	0.0000121	down
3262	A_23_P328034	C20orf96	2.361709798	0.00411596	down
3263	A_24_P139901	GYPC	2.360867105	0.001226651	down
3264	A_24_P203298	IQUB	2.358931186	0.0004079	down
3265	A_23_P258698	MANBA	2.3584982	0.0000291	down
3266	A_23_P153767	AKAP8L	2.35787423	0.014808789	down
3267	A_33_P3306024	SIGLEC16	2.357107135	0.0000558	down
3268	A_23_P357760	ARSD	2.357099238	0.0000242	down
3269	A_24_P394368	WDR19	2.356232658	0.0000041	down
3270	A_23_P217015	SET	2.355182734	0.0000286	down
3271	A_33_P3236881	C1orf15-NBL1	2.354507525	0.0000016	down
3272	A_32_P34444	FHOD3	2.354106626	0.00000349	down
3273	A_23_P204696	CDKN1B	2.353540099	0.002320555	down
3274	A_23_P28434	VAMP8	2.350547986	0.0003057	down
3275	A_32_P16854	ANAPC13	2.349761504	0.000119269	down
3276	A_23_P311740	CUL9	2.349733272	0.0000485	down
3277	A_24_P174503	AMT	2.349450437	0.002810878	down
3278	A_23_P363936	HSPA4L	2.348563986	0.00000605	down
3279	A_33_P3324755	LOC10012878[illegible]	2.347557074	0.000708489	down
3280	A_23_P59045	HIST1H2AE	2.346221412	0.000026	down
3281	A_23_P387630	STARD8	2.345153409	0.000191963	down
3282	A_33_P3404097	PGM5P2	2.345085733	0.002509936	down
3283	A_33_P3695899	FLJ31104	2.345001372	0.003378995	down
3284	A_33_P3246997	LUC7L3	2.344775123	0.0001962	down
3285	A_24_P346431	TNS3	2.34353607	0.0000259	down
3286	A_23_P140725	IFT140	2.343440773	0.000394385	down
3287	A_23_P355439	HIST1H2AA	2.342753774	0.000237718	down
3288	A_32_P78491	ETV1	2.342683137	0.0000458	down
3289	A_23_P416395	STC2	2.342189275	0.000000183	down
3290	A_24_P32887	BRD3	2.341489822	0.005103481	down
3291	A_33_P3321836	PLXNB2	2.34082108	0.030118541	down
3292	A_23_P145606	CHRM2	2.340705288	0.000312612	down
3293	A_23_P86330	IER5	2.33970234	0.000000115	down
3294	A_23_P18692	ADH5	2.339180192	0.000000317	down
3295	A_23_P14174	TNFSF13B	2.338094055	0.000072	down
3296	A_33_P3400828	NXNL2	2.336525906	0.001054871	down
3297	A_32_P161913	C1orf70	2.336483151	0.0000415	down
3298	A_23_P254594	GNRH1	2.336473487	0.00023048	down
3299	A_33_P3263538	NEAT1	2.336253243	0.00178986	down
3300	A_33_P3344204	ZDHHC11	2.335879361	0.000158305	down
3301	A_24_P130936	DDX3Y	2.335822531	0.01937449	down
3302	A_24_P712562	C17orf67	2.333723596	0.000102331	down
3303	A_33_P3395605	TMEM119	2.332655623	0.0000183	down
3304	A_23_P118842	KRTAP1-5	2.332553277	0.00039843	down
3305	A_33_P3382860	RND2	2.331352571	0.003456995	down
3306	A_23_P155857	NUDT6	2.33099353	0.00000123	down
3307	A_23_P19816	RNF32	2.330087663	0.000595325	down
3308	A_23_P64898	KLRG1	2.329456032	0.000119156	down
3309	A_33_P3407529	PRRT4	2.329085821	0.0000842	down
3310	A_33_P3248227	PVT1	2.327862212	0.0000436	down
3311	A_32_P192615	TAF1	2.327121441	3.82E-08	down

3312	A_33_P3780123	FLJ33065	2.326999122	0.000284008	down
3313	A_33_P3295056	PTPRCAP	2.326513244	0.003847673	down
3314	A_23_P41599	PCDHB8	2.326512062	0.0000572	down
3315	A_33_P3792328	LSM3	2.326263786	0.000210595	down
3316	A_23_P411851	C14orf49	2.326220842	0.011776867	down
3317	A_23_P102331	SCN7A	2.32571073	0.000137651	down
3318	A_33_P3363271	CACNB2	2.325546037	0.0000528	down
3319	A_33_P3294861	FLJ40712	2.325469901	0.00644486	down
3320	A_23_P75071	KIF20B	2.324948136	0.0000337	down
3321	A_23_P84922	HDAC8	2.324273754	0.002353589	down
3322	A_24_P230176	CCDC137	2.32394759	0.042916152	down
3323	A_23_P77228	CRTC3	2.323891158	0.000511951	down
3324	A_33_P3264662	CYP27C1	2.323707588	0.000811485	down
3325	A_33_P3214199	ZNF532	2.323674945	6.04E-08	down
3326	A_23_P398530	WHSC1	2.32299578	0.001021271	down
3327	A_23_P60166	DEPTOR	2.322165023	0.00000584	down
3328	A_23_P59410	KIF25	2.321719743	0.028377738	down
3329	A_32_P79483	LOC283481	2.321065281	0.000121345	down
3330	A_33_P3230548	KIF14	2.320893249	0.0000225	down
3331	A_33_P3314659	SPEF2	2.320819678	0.0000364	down
3332	A_33_P3378051	SESTD1	2.320739353	0.0000643	down
3333	A_23_P333640	PAPLN	2.319304645	0.0000785	down
3334	A_23_P384056	CCDC14	2.318157625	0.00000361	down
3335	A_24_P481375	LOC10013156	2.317919827	0.002534274	down
3336	A_23_P210176	ITGA6	2.31565014	0.0000206	down
3337	A_23_P335452	ZCCHC24	2.314111961	0.0000256	down
3338	A_24_P292964	CDK5RAP3	2.312465486	0.005105167	down
3339	A_23_P215787	HBP1	2.312159624	0.0000359	down
3340	A_23_P28090	STX10	2.311940603	0.01414903	down
3341	A_24_P173124	FLCN	2.310763428	0.001172997	down
3342	A_24_P296508	SLC43A2	2.31028473	0.000447639	down
3343	A_23_P308673	TAOK2	2.308288154	0.000273545	down
3344	A_23_P41455	TRPC3	2.306594553	0.000137439	down
3345	A_23_P206310	KIAA0513	2.305194261	0.00000345	down
3346	A_33_P3579984	LOC285147	2.304845426	0.0000104	down
3347	A_23_P104323	MGMT	2.303616669	0.0000742	down
3348	A_23_P154585	SNX21	2.302692871	0.0000426	down
3349	A_23_P1912	ZP1	2.302230739	0.00000725	down
3350	A_24_P943472	NR1D2	2.301910912	0.0000323	down
3351	A_24_P114249	GALNT3	2.300172034	0.00000342	down
3352	A_23_P403424	JMJD7-PLA2G	2.299516155	0.00000747	down
3353	A_32_P192474	PRRT1	2.29868535	0.0000231	down
3354	A_24_P22050	RAB20	2.298506214	0.01287018	down
3355	A_23_P143662	PISD	2.298070143	0.002416262	down
3356	A_23_P141092	TFAP4	2.297995384	0.0124232	down
3357	A_33_P3303372	PARD3	2.295013012	0.000171092	down
3358	A_33_P3291614	LOC10013303	2.293999643	0.008404704	down
3359	A_23_P66137	SOX8	2.293582285	0.000328335	down
3360	A_32_P32413	SETBP1	2.292562926	0.0000201	down
3361	A_24_P356130	MAP2K5	2.292092076	0.004768089	down
3362	A_33_P3304293	STAG2	2.290541285	0.035423954	down

3363	A_23_P122443	HIST1H1C	2.290475027	0.0000307	down
3364	A_23_P103756	OVGP1	2.289790435	0.00000384	down
3365	A_33_P3264200	EGFEM1P	2.289033325	0.0000916	down
3366	A_23_P304682	EMP2	2.287233517	0.00000642	down
3367	A_23_P408232	MGC23270	2.287053741	0.001057946	down
3368	A_23_P8754	AASS	2.286994929	0.000155581	down
3369	A_33_P3267651	DZIP3	2.28655972	0.026888608	down
3370	A_23_P356004	KCNIP3	2.286012459	0.000000441	down
3371	A_23_P73114	PROS1	2.285566824	0.0000385	down
3372	A_24_P497464	FLJ37644	2.285266631	0.00020394	down
3373	A_33_P3387696	TMBIM4	2.285133313	0.0000232	down
3374	A_23_P45025	MAPK10	2.284741587	0.000280185	down
3375	A_23_P26854	ARHGAP44	2.283780295	0.0000469	down
3376	A_32_P190769	C1QTNF7	2.283027546	0.0000853	down
3377	A_23_P106835	BBS2	2.281456268	0.000033	down
3378	A_23_P108294	PPAP2C	2.280800615	0.00000597	down
3379	A_23_P76109	RILPL2	2.280694695	0.0000447	down
3380	A_23_P361544	CES4A	2.279421828	0.000246748	down
3381	A_24_P247106	AK3	2.279313971	0.00000482	down
3382	A_23_P84872	SECISBP2	2.279098062	0.00000632	down
3383	A_33_P3228102	NPHP3	2.276983441	0.001840158	down
3384	A_23_P342709	FBXO15	2.276175661	0.000098	down
3385	A_23_P130158	WNT3	2.275961521	0.000982215	down
3386	A_33_P3228435	FXYD1	2.275582671	0.0000366	down
3387	A_23_P134384	PHF14	2.274734235	0.0000428	down
3388	A_23_P171255	IGBP1	2.274502993	0.00000498	down
3389	A_23_P419714	BTBD11	2.273902927	0.0000958	down
3390	A_33_P3893191	DBIL5P2	2.273396146	0.000624152	down
3391	A_23_P48339	IFT88	2.272637631	0.000000435	down
3392	A_33_P3365870	BSDC1	2.272198645	0.047151111	down
3393	A_23_P115919	PHYH	2.271538357	0.00000109	down
3394	A_23_P215790	EGFR	2.271122199	0.0000866	down
3395	A_33_P3271105	PABPC4	2.271003873	0.024930997	down
3396	A_32_P79434	PTPRN2	2.270155256	0.002198269	down
3397	A_24_P113131	BZRAP1	2.270053344	0.00011187	down
3398	A_24_P184305	BBS1	2.269341928	0.0000037	down
3399	A_23_P134744	RNF122	2.269278275	0.000043	down
3400	A_24_P588897	SLCO3A1	2.268494403	0.000013	down
3401	A_23_P91283	CASS4	2.268377786	0.0000142	down
3402	A_24_P16378	HGSNAT	2.268242728	0.007174396	down
3403	A_32_P202125	LOC10012898	2.267185128	0.000445403	down
3404	A_33_P3272105	FLJ38576	2.267153908	0.038706218	down
3405	A_33_P3345708	CREB3L4	2.265818132	0.003900506	down
3406	A_24_P202139	METTL9	2.265581411	0.0000436	down
3407	A_23_P168771	CCDC146	2.265456831	0.000523634	down
3408	A_33_P3289406	LOC91450	2.263766516	0.0000148	down
3409	A_33_P3405459	C20orf195	2.262630024	0.00000148	down
3410	A_23_P53015	TUT1	2.26174576	0.032392799	down
3411	A_23_P216679	CDC14B	2.261090599	0.00000721	down
3412	A_23_P156907	SOBP	2.260439022	0.0000326	down
3413	A_23_P125078	SLC26A11	2.259947879	8.64E-08	down

3414	A_23_P36985	PCDH8	2.259032459	0.000293349	down
3415	A_33_P3334292	PPIAL4G	2.258905107	0.001850306	down
3416	A_32_P507710	PI4KAP2	2.258899366	0.01975668	down
3417	A_23_P361820	ATG2A	2.258846392	0.030322693	down
3418	A_24_P145019	LOC10012985[illegible]	2.258774997	0.000912668	down
3419	A_24_P145316	DTNBP1	2.258652669	0.000189515	down
3420	A_24_P49190	C17orf58	2.25837751	0.00000129	down
3421	A_23_P217899	CCNL2	2.25805741	0.0000149	down
3422	A_33_P3245412	RG9MTD3	2.257421625	0.000012	down
3423	A_33_P3315060	ZC3H11A	2.256186925	0.001246323	down
3424	A_23_P26810	TP53	2.255433631	0.000317294	down
3425	A_24_P168416	PRDX2	2.254959205	0.000702317	down
3426	A_32_P170749	STAG3L2	2.254771129	0.003283706	down
3427	A_24_P289139	SH3KBP1	2.252301036	0.046612854	down
3428	A_23_P252808	WBP1	2.251165046	0.000389121	down
3429	A_23_P155765	HMGB2	2.249073278	0.0000104	down
3430	A_33_P3361771	TPR	2.248917649	0.000589748	down
3431	A_23_P92928	C6	2.247952316	0.005185467	down
3432	A_23_P20427	RHOBTB2	2.245952111	0.001345472	down
3433	A_23_P153941	CAMKMT	2.245638547	0.0000338	down
3434	A_33_P3347971	TPD52	2.245273927	0.000025	down
3435	A_23_P54953	SAP30BP	2.245087489	0.00000769	down
3436	A_33_P3218699	MDH1B	2.244289261	0.001001557	down
3437	A_23_P99063	LUM	2.242692257	0.0000371	down
3438	A_32_P52609	LPIN1	2.241941551	0.0000419	down
3439	A_23_P419213	KIAA1407	2.239429631	0.0000334	down
3440	A_23_P87545	IFITM3	2.237973572	0.000000105	down
3441	A_23_P146849	APBA2	2.237733142	0.000361832	down
3442	A_33_P3676515	C21orf122	2.237163245	0.00005	down
3443	A_24_P180242	STIM2	2.236336625	0.000774188	down
3444	A_33_P3417195	C17orf82	2.236173663	0.00000315	down
3445	A_33_P3337415	GNAL	2.234696022	0.000122928	down
3446	A_23_P209032	ZNF302	2.234206703	0.000130296	down
3447	A_33_P3218905	FAM13AOS	2.234078945	0.0000332	down
3448	A_23_P394917	SRCAP	2.233782728	0.035065437	down
3449	A_24_P126628	TMEM194A	2.233501928	0.00047809	down
3450	A_23_P99942	LRRK1	2.23206339	0.0000119	down
3451	A_24_P390833	MPPE1	2.231148697	0.000182429	down
3452	A_23_P45917	CKS1B	2.230016936	0.000254928	down
3453	A_23_P149121	DIRAS3	2.229783543	0.0000629	down
3454	A_23_P62901	BTG2	2.229441483	0.0000117	down
3455	A_33_P3262475	SERP2	2.229175651	0.02144399	down
3456	A_33_P3387831	CENPM	2.228004788	0.000000196	down
3457	A_33_P3386765	ABHD14A	2.227809181	0.0000242	down
3458	A_23_P164179	TOB1	2.227583482	0.000865305	down
3459	A_23_P84189	PITPNC1	2.226910793	0.0000168	down
3460	A_23_P19243	AKIRIN2-AS1	2.226719603	0.0000154	down
3461	A_24_P344516	ZNF702P	2.226317778	0.000203001	down
3462	A_23_P166566	CCDC48	2.225771513	0.001016431	down
3463	A_23_P88904	NTHL1	2.224413503	0.000165169	down
3464	A_23_P78782	CA11	2.224310716	0.002881801	down

3465	A_24_P411749	GPR126	2.224132648	0.0000626	down
3466	A_32_P49867	LOC10050705	2.22391174	0.006664976	down
3467	A_23_P419503	LRRC4B	2.223707861	0.00133067	down
3468	A_23_P150189	MRE11A	2.223181241	0.000003	down
3469	A_24_P36847	DHX9	2.222413601	0.003320374	down
3470	A_24_P410086	SSBP4	2.22190659	0.001120815	down
3471	A_33_P3540725	FLJ25917	2.221533915	0.000163005	down
3472	A_33_P3685572	LOC157562	2.219886374	0.0000268	down
3473	A_33_P3732466	DKFZp547G18	2.219252671	0.000549284	down
3474	A_23_P205697	DLST	2.219143302	0.000107895	down
3475	A_33_P3288189	RHOBTB3	2.218884132	2.04E-08	down
3476	A_33_P3233608	SPANXB2	2.218372596	0.010846285	down
3477	A_32_P77098	TMEM200B	2.217788414	0.000503318	down
3478	A_33_P3288824	H2AFB3	2.217336969	0.0002471	down
3479	A_23_P351757	PLCD3	2.217173342	0.035489155	down
3480	A_24_P5305	SPG7	2.217096399	0.029204878	down
3481	A_23_P142776	EIF3F	2.216482031	0.000696003	down
3482	A_23_P126706	ANGPTL1	2.215594251	0.000064	down
3483	A_33_P3323742	PNRC2	2.213305093	0.00000657	down
3484	A_33_P3262927	PCBP4	2.212897508	0.043471913	down
3485	A_24_P82106	MMP14	2.21252159	0.032157735	down
3486	A_23_P155969	PLK4	2.212000993	0.0000158	down
3487	A_23_P41344	EREG	2.211764682	0.000990327	down
3488	A_33_P3415551	GPAT2	2.211490482	0.006225476	down
3489	A_23_P206661	NQO1	2.210924612	0.00032064	down
3490	A_33_P3854030	MBD5	2.209584851	0.000000823	down
3491	A_23_P33984	TMEM27	2.20956106	0.0000153	down
3492	A_23_P41789	SLC27A6	2.209404899	0.001604798	down
3493	A_24_P109214	APOC1	2.203870113	0.000119756	down
3494	A_23_P105619	TMEM116	2.203485394	0.000481427	down
3495	A_33_P3298159	PTGDS	2.202246306	0.00000315	down
3496	A_23_P388993	ZC3H12C	2.201170146	0.000217446	down
3497	A_33_P3293336	GFRA1	2.200466386	0.000000205	down
3498	A_33_P3360675	PRKAR1B	2.199492477	0.000124369	down
3499	A_24_P56363	CAB39L	2.198598497	0.000107869	down
3500	A_23_P65741	DIS3L	2.198068987	6.14E-08	down
3501	A_24_P98914	PFKM	2.198029629	0.0000229	down
3502	A_23_P142322	CIRBP	2.197776986	0.0000221	down
3503	A_24_P132950	NAP1L4	2.196471581	0.00000751	down
3504	A_33_P3646133	NFATC2IP	2.196000932	0.000161957	down
3505	A_33_P3265494	C6orf138	2.195941315	0.0000836	down
3506	A_23_P121499	WFS1	2.195820311	0.00000488	down
3507	A_23_P369666	ZMYND8	2.194489296	0.0000546	down
3508	A_23_P114689	ASAP3	2.19419843	0.00000371	down
3509	A_23_P338325	ELK3	2.192474151	0.000000717	down
3510	A_23_P302005	STON1	2.191685311	0.0000355	down
3511	A_23_P45389	RAB9A	2.191548085	0.0000232	down
3512	A_23_P423197	RXRA	2.190532066	0.000104246	down
3513	A_33_P3313929	CCR6	2.190396936	0.016050906	down
3514	A_33_P3789693	MGC24103	2.190257259	0.001043339	down
3515	A_24_P110780	C1orf118	2.1902523	0.000000947	down

3516	A_33_P3209537	EBF4	2.190172244	0.000000768	down
3517	A_23_P80902	KIF15	2.189398649	0.00289227	down
3518	A_24_P114255	MBOAT2	2.18895101	0.0000389	down
3519	A_33_P3236340	LOC10013313	2.188646314	0.015123048	down
3520	A_24_P942017	LOC10013235	2.188266173	0.000112641	down
3521	A_32_P22078	RPS9	2.185594361	0.000066	down
3522	A_33_P3361388	MYCBPAP	2.18383451	0.002048648	down
3523	A_33_P3239849	GPX1	2.183376103	0.00000573	down
3524	A_23_P110492	6-Mar	2.182926669	0.000266677	down
3525	A_24_P236522	CD2BP2	2.181296168	0.011870474	down
3526	A_33_P3255209	LOC151162	2.180758984	0.0000017	down
3527	A_33_P3423556	DPP6	2.180572009	0.008606983	down
3528	A_23_P387184	NHSL1	2.179640344	0.0000255	down
3529	A_23_P68899	TXN2	2.179178437	0.000190396	down
3530	A_23_P88602	MEIS2	2.178098955	0.0000364	down
3531	A_23_P409626	HNRNPUL2	2.178008373	0.015886336	down
3532	A_23_P97283	PAQR6	2.177711992	0.00000021	down
3533	A_23_P53557	LTBR	2.177626709	0.030790458	down
3534	A_23_P385861	CDCA2	2.177255223	0.000131436	down
3535	A_23_P355455	TBC1D5	2.176945062	0.0000434	down
3536	A_23_P68505	C20orf177	2.176917147	0.000107783	down
3537	A_23_P254842	HDHD1	2.176032846	0.0000125	down
3538	A_24_P105391	ERMAP	2.17555225	0.000072	down
3539	A_23_P421423	TNFAIP2	2.174900399	0.0000458	down
3540	A_23_P393645	ADAMTS13	2.173550326	0.000000042	down
3541	A_23_P26154	PLIN1	2.173256561	0.002070265	down
3542	A_23_P380928	ARPC4-TTLL3	2.173105676	0.0000207	down
3543	A_23_P407992	CRAMP1L	2.172882105	0.010104196	down
3544	A_23_P161297	OGDHL	2.172532361	0.005156712	down
3545	A_33_P3250083	NFATC4	2.172527843	0.0000549	down
3546	A_23_P39799	LOXL3	2.1720149	0.000000467	down
3547	A_23_P201939	PPM1J	2.171935661	0.023119917	down
3548	A_23_P51187	PRKCZ	2.171591286	0.0000921	down
3549	A_24_P38347	DPYSL2	2.171247217	0.00000333	down
3550	A_33_P3321801	LOC10027221	2.170948597	0.007165303	down
3551	A_33_P3212188	LRP3	2.170256656	0.0000372	down
3552	A_24_P80204	MALL	2.170252745	0.000446269	down
3553	A_33_P3338360	SCARNA13	2.169392854	0.000417388	down
3554	A_23_P207058	SOCS3	2.169129721	0.000299096	down
3555	A_33_P3244112	UOX	2.168520227	0.000449506	down
3556	A_23_P67829	UXS1	2.168136869	0.00000336	down
3557	A_33_P3374878	FAT4	2.167814785	0.0000222	down
3558	A_23_P105002	ROM1	2.167129951	0.00000507	down
3559	A_23_P103968	AKR7A3	2.166851572	0.0000548	down
3560	A_24_P183664	TRIL	2.166778629	0.000598255	down
3561	A_24_P144773	RNF145	2.166363044	0.006617105	down
3562	A_33_P3275846	CACNA1A	2.166297925	0.0000823	down
3563	A_23_P253484	AADAT	2.165885035	0.0000354	down
3564	A_33_P3381781	RPRD2	2.164962296	0.00000321	down
3565	A_23_P309381	HIST2H2AA4	2.164321519	0.000170501	down
3566	A_33_P3340385	ZNF43	2.163449832	0.000107845	down

3567	A_33_P3416366	CHAF1A	2.163185721	0.005723215	down
3568	A_23_P202696	KBTBD4	2.162691173	0.008749903	down
3569	A_24_P337334	DCTD	2.162669437	0.0000803	down
3570	A_23_P128073	SMARCC2	2.161720997	0.000242602	down
3571	A_33_P3247392	TPTE	2.161678893	0.00066398	down
3572	A_33_P3282556	TMEM204	2.160807671	0.00000765	down
3573	A_23_P151662	MAX	2.160369022	0.00000267	down
3574	A_23_P389141	SLFNL1	2.159923226	0.000305966	down
3575	A_33_P3344911	PTPRQ	2.15941286	0.0000417	down
3576	A_23_P359043	AKAP2	2.159300454	0.000624989	down
3577	A_23_P410224	C6orf52	2.159159369	0.0000136	down
3578	A_24_P268160	DRAM2	2.158230071	0.0000457	down
3579	A_24_P408736	GALNT5	2.156205974	0.00000182	down
3580	A_23_P350107	TRIM56	2.155707594	0.000152593	down
3581	A_33_P3474859	LOC203274	2.155651561	0.000000127	down
3582	A_33_P3386219	DCLRE1C	2.155582033	0.00038592	down
3583	A_24_P345002	NUDT11	2.154679316	0.0000278	down
3584	A_24_P400044	NUDT10	2.154309106	0.001425793	down
3585	A_24_P941922	CNOT4	2.153870732	0.000531095	down
3586	A_23_P130974	KIAA1683	2.153170652	0.000018	down
3587	A_24_P366777	NOTCH2NL	2.152983107	0.006132131	down
3588	A_23_P204052	PCBP2	2.151815424	0.009930067	down
3589	A_24_P218001	ZNF273	2.150389899	0.0000167	down
3590	A_33_P3351249	CXCL16	2.149915613	4.6E-09	down
3591	A_23_P67391	KPTN	2.149887349	0.0000217	down
3592	A_33_P3312039	RAD23B	2.149150577	0.005234706	down
3593	A_23_P133807	TAF8	2.149089054	0.0015384	down
3594	A_24_P136161	HNRNPCL1	2.148460868	0.004203063	down
3595	A_23_P369456	SYS1	2.147645686	0.00000344	down
3596	A_23_P57417	MMP11	2.1474745	0.000424384	down
3597	A_24_P497186	IRF2BP2	2.147383702	0.001815473	down
3598	A_33_P3628409	PKI55	2.146704083	0.000117445	down
3599	A_24_P416370	HOXB4	2.145409929	0.0000258	down
3600	A_33_P3329098	WTAP	2.145076501	0.0000331	down
3601	A_33_P3824237	LOC439990	2.144102337	0.00000698	down
3602	A_33_P3244122	HAAO	2.141642984	0.0000602	down
3603	A_23_P166775	IL17RC	2.139812816	0.02012005	down
3604	A_33_P3636590	SUCLG2	2.139678195	0.000000106	down
3605	A_24_P191971	SAP30L	2.138340105	0.000159762	down
3606	A_24_P211558	PATZ1	2.137911451	0.004171733	down
3607	A_23_P501538	HOXA3	2.137820169	0.00019186	down
3608	A_33_P3310552	FMNL2	2.136906918	0.000313017	down
3609	A_33_P3630129	FLJ46875	2.136685936	0.000310941	down
3610	A_24_P148450	UBE2E3	2.136599988	0.000096	down
3611	A_23_P71752	ZFAND5	2.135384204	0.00000163	down
3612	A_33_P3220663	STAC3	2.135036598	0.000777161	down
3613	A_24_P262321	FAM18B2-CDF	2.134302696	0.023774877	down
3614	A_33_P3436646	LOC151657	2.133477506	0.004714878	down
3615	A_23_P399797	SMAD5-AS1	2.132990441	0.000132149	down
3616	A_33_P3372788	NBPF15	2.131779321	0.025210474	down
3617	A_23_P254768	HCG4	2.131025269	0.0000629	down

3618	A_33_P3372699	SEC14L4	2.131008627	0.00027015	down
3619	A_32_P797019	NPEPL1	2.129638808	0.003042315	down
3620	A_23_P145114	GCLC	2.12906476	0.00000835	down
3621	A_33_P3640690	ZEB1	2.126838764	0.0000248	down
3622	A_32_P46981	HSBP1L1	2.125880251	0.002066738	down
3623	A_33_P3210880	ATP7A	2.125671263	0.0000559	down
3624	A_33_P3388006	SPICE1	2.125502516	0.000151882	down
3625	A_33_P3284077	NUP133	2.125491908	0.016735389	down
3626	A_23_P3602	NUDT7	2.124663694	0.000233839	down
3627	A_23_P52676	CATSPER1	2.12431317	0.001175128	down
3628	A_23_P107166	ACBD4	2.123884776	0.004432568	down
3629	A_23_P47904	CCDC65	2.123130475	0.002467164	down
3630	A_33_P3370812	TBC1D1	2.123076172	0.0000932	down
3631	A_24_P148836	KLHDC8B	2.122962175	0.020829008	down
3632	A_23_P156970	MEST	2.121896077	0.00000216	down
3633	A_33_P3259373	PAR1	2.121503512	0.044315073	down
3634	A_24_P219053	KIAA1704	2.120506394	0.0000307	down
3635	A_23_P87742	IFFO1	2.120135786	0.00000657	down
3636	A_23_P116614	ME3	2.119614889	0.0000906	down
3637	A_32_P525524	ITPRIPL1	2.119448092	0.003011191	down
3638	A_23_P258418	TNIP2	2.117640072	0.044806109	down
3639	A_23_P17914	PNPLA3	2.117636011	0.002040054	down
3640	A_33_P3335865	WDR35	2.115891036	0.0000838	down
3641	A_33_P3389653	PDE4D	2.114552676	0.0000205	down
3642	A_23_P24135	TACR2	2.114179689	0.008575549	down
3643	A_23_P207245	TEKT3	2.114155168	0.000331064	down
3644	A_33_P3383029	MXI1	2.113429274	0.000042	down
3645	A_23_P24586	ACCS	2.113413941	0.0000102	down
3646	A_23_P253200	RPL15	2.113038665	0.0000389	down
3647	A_33_P3244096	CASK	2.112430973	0.001063779	down
3648	A_33_P3405424	IL4I1	2.112214425	0.000691823	down
3649	A_24_P13475	SPATA13	2.11199585	0.00000876	down
3650	A_23_P250302	CCR3	2.111775395	0.039534189	down
3651	A_33_P3245006	DAK	2.111198162	0.0000121	down
3652	A_24_P257348	ARL6IP5	2.110527557	0.000269488	down
3653	A_32_P19539	TMEM56	2.110207498	0.0000356	down
3654	A_23_P215060	PODXL	2.109810904	0.00000266	down
3655	A_33_P3330608	PRAM1	2.109497191	0.000361754	down
3656	A_23_P126623	PGD	2.108547224	0.0000135	down
3657	A_23_P100420	ZCCHC14	2.107745482	0.00000908	down
3658	A_24_P54178	TMED5	2.107112487	0.0000634	down
3659	A_23_P15394	CD68	2.106549135	0.000771135	down
3660	A_24_P521994	KLHL24	2.105812035	0.000000226	down
3661	A_24_P381604	ITM2B	2.103988521	0.0000541	down
3662	A_24_P120115	CFLAR	2.103878757	0.000110173	down
3663	A_24_P414719	NFYA	2.103862132	0.000531222	down
3664	A_33_P3530868	LOC255654	2.102774921	0.004249864	down
3665	A_23_P132910	RBM47	2.101817491	0.0000531	down
3666	A_32_P195401	FAM117B	2.101200743	0.00000186	down
3667	A_32_P83465	NBPF10	2.099790411	0.001170006	down
3668	A_33_P3346073	SRL	2.098947284	0.003652293	down

3669	A_33_P3406636	ZFP62	2.098925655	0.008603565	down
3670	A_33_P3328736	CCDC23	2.098736434	0.000112751	down
3671	A_33_P3256500	ATXN2	2.097662146	0.00000284	down
3672	A_33_P3379091	SYNGR1	2.097624343	0.0000258	down
3673	A_24_P237927	FAM102B	2.097028158	0.00517497	down
3674	A_23_P149992	PDLIM1	2.097010376	0.000786375	down
3675	A_24_P391574	LDLRAD3	2.095816684	0.007954039	down
3676	A_23_P500799	CASP6	2.09564377	0.00000418	down
3677	A_33_P3452003	LOC143286	2.095412628	0.001036194	down
3678	A_23_P58869	GSTM2P1	2.095320594	0.001152571	down
3679	A_23_P69908	GLRX	2.094681167	0.0000361	down
3680	A_24_P911676	SOX4	2.09432475	0.000367256	down
3681	A_23_P250735	CBX7	2.093781168	0.0000452	down
3682	A_23_P130376	FAM38B	2.092252107	0.000612642	down
3683	A_24_P394940	CYP2E1	2.092040769	0.00003	down
3684	A_33_P3242829	PKN2	2.091635218	0.000247019	down
3685	A_23_P120125	COLEC11	2.090577848	0.0000694	down
3686	A_33_P3380311	ATRX	2.090387979	0.0000671	down
3687	A_24_P317827	TMEM8B	2.089365659	0.00000045	down
3688	A_33_P3317211	MECP2	2.085979144	0.037719743	down
3689	A_23_P218784	DDX17	2.085917357	0.001454147	down
3690	A_23_P4190	ACSF2	2.08474414	0.000786053	down
3691	A_33_P3214027	SPECC1L	2.084392882	0.043492713	down
3692	A_23_P392476	AKD1	2.084232854	0.001876844	down
3693	A_23_P144326	ASB5	2.083401413	0.0000258	down
3694	A_24_P590560	RRN3	2.083358091	0.0000138	down
3695	A_33_P3251640	LOC284440	2.083302398	0.0000449	down
3696	A_23_P320862	C9orf43	2.082938341	0.000140967	down
3697	A_23_P79496	STON1-GTF2/	2.0826753	0.00000248	down
3698	A_32_P90483	STXBP4	2.082513335	0.005729421	down
3699	A_33_P3429242	LOC339988	2.082419702	0.000138164	down
3700	A_32_P138617	KIAA2018	2.081964063	0.00000877	down
3701	A_33_P3256914	ITFG3	2.081654155	0.018103802	down
3702	A_23_P5342	LRP1B	2.081562293	0.00145057	down
3703	A_23_P327156	LOC645431	2.080890716	0.000171297	down
3704	A_23_P148475	KIF4A	2.080863312	0.000819985	down
3705	A_33_P3239287	CHD3	2.080523379	0.000000685	down
3706	A_23_P210948	UQCC	2.080349708	0.003600928	down
3707	A_23_P117037	LETMD1	2.080308275	0.000204012	down
3708	A_33_P3316539	SLC7A2	2.080073009	0.0000124	down
3709	A_23_P213375	PCDHB2	2.079470954	0.000103692	down
3710	A_32_P539599	FLJ40852	2.079187213	0.000177126	down
3711	A_24_P784765	CD59	2.078984497	0.0000532	down
3712	A_23_P141447	RDM1	2.078663025	0.00344197	down
3713	A_23_P35916	ATM	2.077877591	0.00000366	down
3714	A_33_P3242973	IGF2BP2	2.077503489	0.03269437	down
3715	A_23_P11744	WASH1	2.07703952	0.000048	down
3716	A_33_P3338335	MIDN	2.076658996	0.00000633	down
3717	A_23_P331928	CD109	2.076445971	0.00000824	down
3718	A_24_P305938	RAB9BP1	2.076222894	0.00000792	down
3719	A_23_P19352	CNPY3	2.075874894	0.031311204	down

3720	A_33_P3735158	LOC286272	2.075501632	0.0000238	down
3721	A_32_P58614	KIAA1377	2.075110506	0.00000413	down
3722	A_33_P3250383	CNP	2.073839628	0.0000655	down
3723	A_32_P72110	PVR	2.073837855	0.0000261	down
3724	A_33_P3762913	LOC10021654(	2.073352093	0.0000852	down
3725	A_33_P3783235	LOC286052	2.073289627	0.000118864	down
3726	A_23_P372638	CHD9	2.072900354	0.00470017	down
3727	A_33_P3245278	PTPRG	2.072811704	0.000266727	down
3728	A_23_P313632	FUT8	2.072173544	0.000478938	down
3729	A_32_P167076	CAPN14	2.071406451	0.006542919	down
3730	A_23_P90099	TMEM205	2.069021065	0.0000104	down
3731	A_23_P335239	GAB1	2.06899874	0.0000554	down
3732	A_33_P3347193	VSTM2A	2.068958824	0.0000803	down
3733	A_33_P3242663	VASH2	2.068360225	0.0000218	down
3734	A_33_P3257518	FLJ22447	2.067215415	0.000537182	down
3735	A_32_P6172	LOC10012882:	2.066698447	0.0000033	down
3736	A_32_P225659	UTS2D	2.066399596	0.000105955	down
3737	A_23_P210608	ZNF217	2.065662656	0.000108283	down
3738	A_23_P4074	WDR16	2.065398457	0.005399679	down
3739	A_23_P66719	DHRS13	2.065188257	0.012680167	down
3740	A_23_P87150	LPXN	2.064993156	0.0000548	down
3741	A_24_P291133	UHMK1	2.064937525	0.000524331	down
3742	A_23_P39910	COA5	2.064863385	0.0000575	down
3743	A_33_P3259960	LOC10012801!	2.064104912	0.002353272	down
3744	A_23_P202219	CALHM2	2.063339661	3.63E-08	down
3745	A_23_P40588	HSCB	2.063314871	0.0000032	down
3746	A_23_P163820	ZNF629	2.062863222	0.003682852	down
3747	A_23_P259955	GDF5	2.062623018	0.000115735	down
3748	A_33_P3422659	GDAP1	2.062024489	0.001692688	down
3749	A_33_P3278068	ELF1	2.061658053	0.002527235	down
3750	A_23_P97932	MSRB2	2.060384607	0.0000681	down
3751	A_23_P92082	TKT	2.060244177	0.000000954	down
3752	A_33_P3290338	PARP1	2.059517569	0.002828228	down
3753	A_23_P145074	PNRC1	2.057209422	0.000000626	down
3754	A_24_P260639	HIST1H1D	2.056888609	0.0000112	down
3755	A_24_P73075	TTC12	2.056586852	0.000620797	down
3756	A_23_P251051	NF2	2.056525556	0.0000646	down
3757	A_32_P129527	C6orf70	2.055138562	0.00000224	down
3758	A_24_P181120	PFDN5	2.054802404	0.000244509	down
3759	A_23_P252740	DSCC1	2.054791247	0.000000196	down
3760	A_23_P77048	SLC25A29	2.054399372	0.000180392	down
3761	A_24_P11965	MRFAP1	2.05412266	0.01801523	down
3762	A_23_P319895	SETD1B	2.054036521	0.002815039	down
3763	A_33_P3390027	EXOC7	2.053727354	0.000643655	down
3764	A_24_P94054	STK4	2.053537842	0.00090466	down
3765	A_23_P216622	FKTN	2.053527831	0.0001997	down
3766	A_33_P3375613	C8orf44-SGK3	2.052696498	0.000046	down
3767	A_24_P45367	NIPAL3	2.052093785	0.00000994	down
3768	A_33_P3415430	HSPA1B	2.051920733	0.0000913	down
3769	A_33_P3407469	MYH7B	2.051399106	0.000415157	down
3770	A_24_P75920	CCDC159	2.051343889	0.000610352	down

3771	A_33_P3341494	ZNF658	2.051024227	0.0000556	down
3772	A_23_P254081	LIAS	2.050554043	0.00000288	down
3773	A_23_P218817	CPT1B	2.049716431	0.0000469	down
3774	A_23_P312610	DNAJC4	2.048662644	0.017499566	down
3775	A_33_P3317628	PKP3	2.048108673	0.0000467	down
3776	A_23_P77437	PRMT7	2.04779136	0.021114626	down
3777	A_32_P486620	IGSF22	2.047622645	0.04082469	down
3778	A_24_P143686	KIAA0146	2.047115969	0.00000245	down
3779	A_23_P138139	OMA1	2.045742175	0.000144345	down
3780	A_32_P175301	DENND3	2.045688291	0.0000318	down
3781	A_33_P3389113	TRAF3IP2-AS1	2.045447394	0.0000162	down
3782	A_33_P3312246	PHIP	2.044454563	0.0000791	down
3783	A_24_P400997	SMCHD1	2.044001611	0.013647544	down
3784	A_33_P3279059	RIPK1	2.04398272	0.000162125	down
3785	A_33_P3403107	PRR19	2.04342501	0.00006	down
3786	A_23_P110879	TRAF3IP2	2.043384501	0.003329926	down
3787	A_23_P338505	C19orf40	2.043144442	0.004495405	down
3788	A_23_P23575	SLC39A1	2.042738221	0.008283002	down
3789	A_33_P3284808	C10orf112	2.042467563	0.002523423	down
3790	A_33_P3273584	SCARNA2	2.042136592	0.013414539	down
3791	A_23_P49842	UNC119	2.040315136	0.031585829	down
3792	A_23_P367676	SIN3A	2.039696736	0.001647466	down
3793	A_33_P3228023	SCPEP1	2.039178593	0.005580238	down
3794	A_33_P3722568	PPP2R3B-AS1	2.039081633	0.000404519	down
3795	A_23_P147450	SPG21	2.038035714	0.000125988	down
3796	A_23_P1056	GPR89B	2.037771564	0.000827804	down
3797	A_33_P3247803	RBM12B	2.037374226	0.000018	down
3798	A_23_P119562	CFD	2.036340288	0.000116543	down
3799	A_24_P107291	PPP2R1B	2.035094094	0.000132402	down
3800	A_23_P9289	RFX3	2.033754164	0.000123826	down
3801	A_24_P102053	OCLN	2.033602581	0.0000118	down
3802	A_24_P267592	SAMHD1	2.033526324	0.000263492	down
3803	A_23_P61674	CLK4	2.033252423	0.00000969	down
3804	A_33_P3387566	C17orf88	2.033074525	0.043110118	down
3805	A_23_P97606	GSTM5	2.032067042	0.0000195	down
3806	A_23_P205623	DDHD1	2.031975208	0.000000632	down
3807	A_33_P3211864	ARMCX4	2.031212953	0.000343107	down
3808	A_23_P21485	PID1	2.031087182	0.000267867	down
3809	A_32_P91042	LOC10012903	2.031063952	0.0000538	down
3810	A_24_P555170	LOC10050567	2.03076514	0.001331715	down
3811	A_33_P3393801	PDZK1IP1	2.030669987	0.01783742	down
3812	A_24_P349965	TCF19	2.029787877	0.000143866	down
3813	A_23_P20494	NDRG1	2.029451693	0.00000951	down
3814	A_24_P179816	SLC27A3	2.029356977	0.000000232	down
3815	A_33_P3332487	FANK1	2.028878071	0.0000349	down
3816	A_23_P368484	C17orf76	2.028599266	0.0000117	down
3817	A_23_P25348	ACAD10	2.027737499	0.030339458	down
3818	A_24_P227831	ABCC1	2.02758866	0.000978557	down
3819	A_23_P36700	TAPBPL	2.02747904	0.000271603	down
3820	A_33_P3359368	DHRS4L1	2.027124878	0.001391435	down
3821	A_23_P92754	FGFR4	2.026958428	0.001391476	down

3822	A_24_P291826	SYTL3	2.026364161	0.0000835	down
3823	A_33_P3348061	CABP7	2.026056116	0.0000373	down
3824	A_23_P257478	CYP21A2	2.026004109	0.0000401	down
3825	A_24_P143138	FGD1	2.025982436	0.01542201	down
3826	A_23_P329890	TMEM136	2.025668364	0.00021176	down
3827	A_33_P3320197	FAM150B	2.025623902	0.000405552	down
3828	A_33_P3316323	RBMS1	2.025620626	0.000110262	down
3829	A_24_P68019	ZNF551	2.025498945	0.000395407	down
3830	A_24_P702813	XPR1	2.025478119	0.0000411	down
3831	A_23_P102607	CHD6	2.025309137	0.00024154	down
3832	A_33_P3355071	MLLT10	2.025015335	0.0000578	down
3833	A_33_P3286724	PLD2	2.024776264	0.000254025	down
3834	A_23_P218358	FBXW10	2.024428514	0.000097	down
3835	A_23_P70566	FKBPL	2.024424772	0.002412449	down
3836	A_24_P396753	TRIB2	2.024383565	0.000104803	down
3837	A_24_P231104	LEPR	2.0243403	0.0000513	down
3838	A_23_P370989	MCM4	2.024255878	0.0000991	down
3839	A_33_P3347055	LOC10013096	2.021484769	0.001662884	down
3840	A_23_P322845	PPAPDC1B	2.021286978	0.0000862	down
3841	A_24_P82142	TCF12	2.019170634	0.000262953	down
3842	A_23_P122615	PNISR	2.019043276	0.00000165	down
3843	A_23_P319859	EYA2	2.018672257	0.000183953	down
3844	A_23_P428184	HIST1H2AD	2.018461636	0.0000573	down
3845	A_23_P116743	LOC338799	2.017342907	4.46E-08	down
3846	A_32_P129894	MEGF9	2.016801831	0.000106846	down
3847	A_33_P3299314	RPL28	2.01664387	0.0000579	down
3848	A_33_P3319880	EVC2	2.01652366	0.02504029	down
3849	A_23_P147109	C9orf167	2.016301897	0.000520507	down
3850	A_33_P3413114	ADAMTSL1	2.015775538	0.000017	down
3851	A_23_P4353	WSB1	2.015694035	0.005271622	down
3852	A_24_P98555	FAM45A	2.015267011	0.000000722	down
3853	A_33_P3357163	PPIEL	2.014923316	0.000224065	down
3854	A_24_P173823	PBX1	2.014587312	0.00000619	down
3855	A_33_P3335735	LOC10012897	2.014517912	0.000033	down
3856	A_23_P323166	SRRM2	2.01394786	0.00000922	down
3857	A_33_P3290443	SCARNA9	2.013247023	0.016253697	down
3858	A_23_P109907	ILDR1	2.012802566	0.0000127	down
3859	A_24_P160680	CCDC40	2.012437809	0.000201781	down
3860	A_24_P169148	HMGB1	2.01237011	0.000193207	down
3861	A_24_P612446	C6orf89	2.011901489	0.039270938	down
3862	A_33_P3239112	PTCH1	2.011496276	0.000140621	down
3863	A_33_P3236698	ANKRD19P	2.011248251	0.0000232	down
3864	A_33_P3414362	USP32	2.011160983	0.000383975	down
3865	A_23_P17103	TSGA10	2.010777383	0.000752101	down
3866	A_23_P350719	PRSS30P	2.010643493	0.000106239	down
3867	A_33_P3299834	POLR2E	2.010140533	0.00000487	down
3868	A_32_P181297	ST7-AS1	2.010107976	0.00000261	down
3869	A_23_P360542	C18orf2	2.010006918	0.009150844	down
3870	A_23_P214222	MARCKS	2.009992335	0.00000106	down
3871	A_23_P166135	XRN2	2.0099654	0.0000592	down
3872	A_23_P39185	RDH13	2.009816379	0.02458483	down

3873	A_23_P143569	DGCR6L	2.009558162	0.011838055	down
3874	A_33_P3380405	CYTH1	2.008865025	0.033317377	down
3875	A_33_P3263392	LOC729696	2.008778046	0.000228671	down
3876	A_24_P317762	LY6E	2.006807637	0.044163039	down
3877	A_24_P158718	DTX4	2.005897842	0.0000183	down
3878	A_24_P921155	C3orf17	2.005647867	0.0000357	down
3879	A_23_P92994	MFF	2.005129386	0.000000489	down
3880	A_24_P935682	LOC10013154	2.004575376	0.002555702	down
3881	A_23_P106737	LUC7L	2.003931925	0.016014526	down
3882	A_24_P316305	AQR	2.003669186	0.000300344	down
3883	A_33_P3383422	DHRS4	2.003400649	0.004894112	down
3884	A_23_P254025	ZFP37	2.003202452	0.0000488	down
3885	A_32_P149060	C21orf71	2.003068974	0.000157207	down
3886	A_33_P3829391	LOC641510	2.002812271	0.000137203	down
3887	A_23_P40880	CMTM8	2.002273381	0.00000217	down
3888	A_23_P130149	ENO3	2.001314639	0.000107755	down
3889	A_33_P3212102	SLC10A6	2.00121	0.028739605	down
3890	A_33_P3347161	TBL1X	2.000970873	0.000224527	down
3891	A_24_P926400	GGA1	2.000931899	0.00000657	down
3892	A_23_P37441	B2M	2.000704362	0.000000495	down
3893	A_23_P365719	TAPBP	2.000681018	0.000581966	down
3894	A_33_P3238636	C2orf77	2.000660956	0.000843278	down

Supplementary Table S3 Five overlapping genes between top 50 up-regulated genes in silica-exposed NR8383 cells and top 50 up-regulated genes in TGF-β-stimulated WI-38 cells

No.	Gene Name	Fold change in NR8383 cells	Fold change in WI-38 cells
1	IER3	9.50	23.37
2	MLLT11	6.63	21.76
3	TAK1	45.51	21.65
4	IL6	60.16	21.11
5	NACC2	5.28	17.46

Supplementary Table S4 Clinical features of pneumoconiosis patients and control individuals

No.	Age	Gender	Working history	Pathological Diagnosis	ILO Classification	Other lung diseases	Smoking
1	54	Male	Drill and construction	Pneumoconiosis, Stage II	Large Parenchymal Opacities (A)	No	No
2	50	Male	Drill and construction	Pneumoconiosis, Stage III	Large Parenchymal Opacities (B)	No	Stopped in 2002
3	46	Male	Drill and construction	Pneumoconiosis, Stage II	Large Parenchymal Opacities (A)	No	Stopped in 2004
4	52	Male	Construction	Pneumoconiosis, Stage I	Small Parenchymal Opacities (t)	No	No
5	48	Male	Drill and construction	Pneumoconiosis, Stage II	Large Parenchymal Opacities (A)	No	Stopped in 2010
6	61	Male	Coal worker	Pneumoconiosis, Stage III	Large Parenchymal Opacities (B)	No	No
7	55	Male	Drill	Pneumoconiosis, Stage II	Large Parenchymal Opacities (A)	No	No
8	49	Male	Construction	Pneumoconiosis, Stage I	Small Parenchymal Opacities (u)	No	No
9	57	Male	Construction and drill	Pneumoconiosis, Stage II	Large Parenchymal Opacities (B)	No	No
10	48	Male	N.A.	Pulmonary contusion	N.A.	No	No
11	37	Male	N.A.	Hemothorax by trauma	N.A.	No	No
12	52	Male	N.A.	Pneumothorax by trauma	N.A.	No	No
13	43	Male	N.A.	Pneumothorax by trauma	N.A.	No	No
14	53	Male	N.A.	Atelectasis	N.A.	No	No
15	55	Male	N.A.	Pulmonary contusion	N.A.	No	No

ILO Classification: International Labour Organization (ILO) International Classification of Radiographs of Pneumoconioses.

Supplementary Table S5 Top 30 small molecules selected from TAK1-based molecular docking

Rank	Molecules	Affinity	LipinskiScore	LeadScore	LipinskiScore2	LeadScore2
1	ZINC13542876	-8.3	7	5	6	6
2	ZINC01561462	-8.2	7	5	6	6
3	ZINC29589879	-8	7	5	6	6
4	ZINC13125795	-8	7	5	6	6
5	ZINC05647206	-8	7	5	6	6
6	ZINC00041118	-7.9	7	5	6	6
7	ZINC13152217	-7.8	7	5	6	6
8	ZINC03258337	-7.8	7	5	6	6
9	ZINC29589876	-7.7	7	5	6	6
10	ZINC01688628	-7.7	7	5	6	6
11	ZINC13130016	-7.7	7	5	6	6
12	ZINC04934182	-7.5	7	5	6	6
13	ZINC01697110	-7.5	7	5	6	6
14	ZINC01676213	-7.4	7	5	6	6
15	ZINC01729467	-7.4	7	5	6	6
16	ZINC02042811	-7.4	7	5	6	6
17	ZINC00006787	-7.3	7	5	6	6
18	ZINC00563913	-7.3	7	5	6	6
19	ZINC05392913	-7.3	7	5	6	6
20	ZINC01653222	-7.2	7	5	6	6
21	ZINC01724376	-7.2	7	5	6	6
22	ZINC00076793	-7.2	7	5	6	6
23	ZINC18117772	-7.2	7	5	6	6
24	ZINC01754899	-7.2	7	5	6	6
25	ZINC29589877	-7.2	7	5	6	6
26	ZINC00387464	-7.2	7	5	6	6
27	ZINC29589797	-7.2	7	5	6	6
28	ZINC04715161	-7.2	7	5	6	6

29	ZINC03954626	-7.1	7	5	6	6
30	ZINC18847035	-7.1	7	5	6	6

Supplementary Table S6 Primers used in real-time PCR

No.	Genes	Forward primer	Reverse primer
1	IER3	CAGCCGCAGGGTTCTCTAC	GATCTGGCAGAAGACGATGGT
2	MLLT11	GGACCCTGTGAGTAGCCAGTA	CAGCTCCGACAGATCCAGT
3	TAK1	ATTGTAGAGCTTCGGCAGTTATC	CTGTAAACACCAACTCATTGCG
4	IL6	ACTCACCTCTTCAGAACGAATTG	CCATCTTTGGAAGGTTCAGGTTG
5	NACC2	CGGCACCGACCTCATGTTC	CTTCATGCTTTACTCGGGTCAG
6	GAPDH	GGAGCGAGATCCCTCCAAAAT	GGCTGTTGTCATACTTCTCATGG
